# Potential benefits of music-based therapies on rehabilitation outcomes in people with multiple sclerosis: a systematic review

**DOI:** 10.1136/bmjopen-2026-116420

**Published:** 2026-07-22

**Authors:** Nicky Ahadi, Jeeyeon Kim, Daniel Whibley, Maxwell S Barnish

**Affiliations:** 1 University of Exeter Medical School, University of Exeter, Exeter, UK; 2 Department of Physical Medicine and Rehabilitation, University of Michigan, Ann Arbor, Michigan, USA; 3 Center for History, Humanities, Arts, Social Sciences, and Ethics in Medicine, University of Michigan, Ann Arbor, Michigan, USA; 4 Peninsula Technology Assessment Group (PenTAG), School of Public Health and Sport Sciences, University of Exeter Medical School, University of Exeter, Exeter, UK

**Keywords:** Multiple sclerosis, COMPLEMENTARY MEDICINE, PUBLIC HEALTH

## Abstract

**Objectives:**

To assess the evidence for a benefit of music-based therapies (MBTs) on rehabilitation outcomes in adults with multiple sclerosis (MS).

**Setting:**

Scholarly literature (published in English) from any country. This systematic review received no specific funding and was registered on PROSPERO as CRD420251083656.

**Data sources:**

Six bibliographic databases: AMED (Ebsco), APA PsycINFO (Ovid), CINAHL (Ebsco), Cochrane CENTRAL, EMBASE (Ovid) and MEDLINE (Ovid), plus supplementary searches and citation chasing (search date July 2025).

**Primary and secondary outcome measures:**

Eligible studies used a clinical trial or controlled quantitative observational study design to assess the benefit of MBTs on rehabilitation outcomes in adults with MS. Risk of bias was assessed using Critical Appraisal Skills Programme checklists. Data were synthesised using narrative synthesis.

**Results:**

23 studies were included (955 participants, of whom 880 people had MS and 691 received MBTs). The most frequently assessed outcome was motor function (16 studies), followed by quality of life (12 studies). Evidence suggests potential benefits for MBTs, particularly rhythmic auditory stimulation and music-cued motor imagery, on gait and mobility outcomes, including walking speed, walking distance, cadence, stride length and gait symmetry. Findings for other outcomes were less clear.

**Conclusions:**

MBTs may benefit people with MS, with the most consistent evidence observed for gait and mobility outcomes. However, heterogeneity in intervention type, study design and outcome measurement limits the strength of conclusions. Higher-quality, adequately powered trials with standardised outcomes and direct comparisons between MBTs are needed to clarify effectiveness and inform clinical implementation.

**PROSPERO registration number:**

CRD420251083656.

**STRENGTHS AND LIMITATIONS OF THIS STUDY**

- Systematic review methods minimised subjectivity and bias.
- A structured outcome set was used.
- An independent dual review was conducted.
- Patient and public involvement was not conducted.
- Only English-language studies could be included.

## Introduction

### Multiple sclerosis

Multiple sclerosis (MS) is a chronic inflammatory and neurodegenerative disease of the central nervous system and a leading cause of neurological disability in young adults.^1 2^ It affects more than 2.8 million people worldwide and its prevalence has increased substantially in many regions, with the highest rates reported in North America, western Europe and Australasia.^1–3^ This rise is attributed to a combination of greater life expectancy, population growth and better access to diagnostic services.^2 3^ Common manifestations include optic neuritis, sensory disturbance, limb weakness and gait ataxia as any site in the central nervous system can be affected.^1–3^ MS is usually described as having three main types with Relapsing Remitting MS (RRMS) being the most common, affecting around 85%–90% of people with MS. Primary progressive MS accounts for about 10%–15% and progresses steadily without relapses. Over time, many with RRMS develop secondary progressive MS, where disability gradually increases.^1^

### Treatment options for MS

Disease-modifying therapies can reduce relapse frequency and delay disability progression, yet they do not fully address the multifactorial physical, cognitive and emotional consequences of living with a chronic, unpredictable condition. MS places a heavy burden on health systems and carries high economic costs.^2 3^ As symptoms often persist despite medication, there is increasing interest in non-pharmacological approaches to support holistic rehabilitation and improve quality of life (QoL).^3 4^ These approaches include arts-based interventions, which have been shown to be promising therapeutic approaches in anxiety and depression,^5^ palliative care,^6^ stroke^7^ and Parkinson’s disease (PD).^8–10^ Within this broad arts sphere, one focal point of therapeutic interest is the use of music for therapeutic benefit.

### The concept of music-based therapies

A range of different approaches have been taken to conceptualising music interventions for therapeutic benefit. One established concept is music therapy (MT). The American Music Therapy Association defines MT as ‘the clinical and evidence-based use of music interventions to accomplish individualised goals within a therapeutic relationship by a credentialed professional who has completed an approved music therapy programme’.^11^ However, definitions of MT vary greatly internationally, making the concept challenging to interpret in the international literature. The UK, USA and Canada take a highly formalised approach to MT using protected, clinically based legal definitions, requiring specific MT qualifications and registration with a statutory body.^11–13^ Meanwhile, the German Music Therapy Society focuses on the ‘targeted use of music’ to restore, maintain and promote ‘mental, physical and cognitive health’,^14^ the Norwegian approach focuses on ‘community music therapy’, combining individual clinical work with social, community-based approaches,^15 16^ and the Chinese approach incorporates theories from traditional Chinese medicine, such as the ‘five tones’ model, alongside western MT approaches.^17^ Areas of between-country variation include whether statutory registration is required, whether the focus is on active or receptive MT approaches, and the extent to which MT is clinically focused and conducted individually or socially focused and conducted in groups. Therefore, MT does not have the same meaning in different countries—interventions that would be described as MT in some countries would not be permitted to be described as MT in other countries.^14^ Pragmatic definitions of MT have been used by some researchers^5 8 9^ to overcome this lack of a universal definition of MT, however, the misalignment of these definitions with those used by statutory bodies may be problematic.

Noting these issues defining MT in a consistent and meaningful way for international research, broader concepts have been proposed that include interventions that may be beneficial but need not be delivered by a certified music therapist. The broadest alternative conceptualisation of the use of music for health benefit is ‘musical care’ (MC)—a concept developed by Spiro and Sanfilippo.^18^ MC is defined as ‘the role of music—music listening as well as music-making—in supporting any aspect of people’s developmental or health needs: for example, physical and mental health, cognitive and behavioural development and interpersonal relationships’.^18^ The authors state^18^ that MC encompasses MT but goes beyond MT and includes a wide range of musically based interventions. The concept of MC considers the role of music in its broadest sense and does not only consider structured interventions, nor does it only consider interventions that are therapeutic—MC includes unstructured activities that include music and interventions where music is not the primary therapeutic modality.^18^ This may be considered a limitation of the MC concept.

Therefore, the concept of music-based therapies (MBTs) has been proposed, as well as the alternative term music-based interventions (MBIs). As these terms are synonymous, we shall use the term MBTs consistently. The concept of MBTs has been used in several previous systematic reviews in neurological conditions.^19–21^ Lin *et al*^19^ delineate their review in the full text as assessing MBTs, although the term MT is used in the article title. However, a common limitation in previous reviews using the MBTs concept across different health conditions has been a lack of an explicit definition of MBTs. The key distinction between MBTs and MT is that while MBTs are designed to be therapeutic and often to enhance general well-being or provide short-term symptom relief, they need not be clinical interventions delivered by a certified music therapist.^22^ Based on the work of Raglio,^22^ we propose the following explicit operational definition of MBTs for the purposes of our work: ‘structured musical activities designed to enhance well-being, reduce stress and improve QoL, without necessarily focusing on clinical diagnosis, assessment or long-term psychotherapy’. While professional regulation and licensing remain important for clinical governance, our conceptualisation prioritises the active components of MBTs over jurisdiction-specific labels. Ultimately, understanding which components produce therapeutic effects is most important for advancing the evidence base. Our theoretical and conceptual justification for aggregating diverse music-based activities under the concept of MBTs lies in their shared complexity as interventions with potentially multiple ‘active ingredients’ that promote health, well-being and neurophysiological change.^23^ MBTs are mid-range in their scope—broader than MT but narrower than MC, as MBTs do not include activities that are not therapeutic or do not comprise structured interventions. MT falls within the stated definition of MBTs, but not all aspects of MC are included under MBTs.

### Evidence for MBTs in MS

We have identified six previous systematic,^20 24–28^ scoping or comprehensive reviews on MBTs for MS, in addition to a broad non-systematic literature review on ‘cognitive re-education’ for QoL and psychological well-being in MS.^29^ Table 1 summarises the scope, methodological approaches, interventions, outcomes and included studies of these reviews. Due to clinical, methodological and statistical heterogeneity between studies, no reviews have been able to include a meta-analysis. An early comprehensive review^26^ assessed MT in relation to a non-specified set of outcomes and found very limited evidence, with the only full-text peer-reviewed papers besides case reports or case series being two pilot studies.^30 31^ Systematic and scoping reviews have typically been limited to specific outcome domains: (1) gait and mobility^25 28^ and (2) psychological outcomes.^24^ Gardener *et al*^24^ conducted a systematic review on MT for psychological outcomes in a broader range of conditions and included only one pilot randomised controlled trial (RCT)^32^ on MS. Tan *et al*^28^ conducted a recent scoping review of 15 studies on music-based and rhythmic auditory interventions for walking in MS. They focused exclusively on gait and mobility outcomes and reported high acceptability and consistent short-term improvements in walking speed and quality. Kong *et al*^25^ similarly undertook a systematic review of 12 clinical trials of MT for gait disorders in MS, again concentrating on mobility outcomes and finding that music-based rhythmic cueing improved gait performance. Together, these reviews support the role of music-based approaches for gait rehabilitation but do not explore wider motor, cognitive or psychosocial domains. Lopes and Keppers^20^ provided the first MS-specific systematic review of MBTs as a rehabilitative approach. Across 10 clinical trials, they grouped interventions into rhythmic auditory strategies, instrument playing, dance-based strategies and neurological MT. They found that MBTs were superior to conventional therapy or no intervention for several motor and non-motor outcomes, including gait, fatigue, balance, hand dexterity, emotional status and pain, although evidence for memory and mental fatigability was less clear. The search period ended in 2020 and interventions and outcome measures were highly heterogeneous, with several protocols primarily dance-based rather than primarily music focused.^20^ Phillips *et al*^27^ extended the scope of the literature by comparing active and receptive music interventions across 15 studies in people with MS, with outcomes including anxiety, depression, QoL, cognition, gait, balance, fatigue and hand dexterity. Most interventions were receptive, often involving music listening during walking. While the review showed encouraging effects across motor and non-motor domains, its focus on engagement type and the limited number of studies mean it does not fully represent the breadth of MBTs for MS rehabilitation.^27^

**Table 1:** Previous systematic, scoping and comprehensive reviews

Authors	Method	Search date	PROSPERO	Interventions	Outcomes	Included studies
Gardener *et al*^24^	Systematic review (without meta-analysis)	January 2025 (limited to literature since 2015)	Not registered, no prepublished protocol	MT	Psychological outcomes	Wider range of neurological conditions including MS. For MS: 1 pilot RCT (Impellizeri *et al*).^32^
Kong *et al*^25^	Systematic review (without meta-analysis)	October 2022	CRD 42022365668	MT	Gait and mobility outcomes	2 case series. 7 RCTs (Conklyn *et al*^46^; Seebacher *et al*^56^; Seebacher *et al*^49^; Seebacher *et al*^59^; Seebacher *et al*^50^; Shahraki *et al*^52^; Young *et al*).^45^ 3 non-RCTs (Maggio *et al*^54^; Ng *et al*^71^; Van Geel *et al*).^72^
Lopes *et al*^20^	Systematic review (without meta-analysis)	July 2020	CRD 42020143080	MBTs	Balance, cognition, dexterity, fatigue, gait, pain, QoL	10 clinical trials (Aldridge *et al*^62^; Conklyn *et al*^46^; Gatti *et al*^47^; Van Geel *et al*^72^; Impellizzeri *et al*^32^; Moore *et al*^48^; Seebacher *et al*^50^; Seebacher *et al*^49^; Thaut *et al*^61^;* Young *et al*).^45^
Ostermann *et al*^26^	Comprehensive review	Not stated	Not registered (review predates PROSPERO)	MT	Not specified	8 conference abstracts, chapters or non-peer-reviewed sources. 2 case reports or case series. 2 pilot studies (Schmid and Aldridge 2004^30^; Wiens *et al*).^19^
Phillips *et al*^27^	Systematic review (without meta-analysis)	June 2024	CRD 42022338291	Music interventions (active or receptive)	Anxiety, balance, cognition, depression, dexterity, fatigue, gait, QoL	10 RCTs (Basturk *et al*^73^; Conklyn *et al*^46^; Gatti *et al*^47^; Impellizzeri *et al*^32^; Moore *et al*^48^; Seebacher *et al*^49^; Seebacher *et al*^50^; Thaut *et al*^60^; Thaut *et al*^61^; Young *et al*).^45^ 5 quasi-experimental studies (Maggio *et al*^54^; Moumdjian *et al*^58^; Schmid and Aldridge^30^; Van Geel *et al*).^72^
Tan *et al*^28^	Scoping review	April 2025	Not registered (but protocol uploaded to Open Science Framework)	Music interventions	Gait and mobility	8 RCTs (Baram and Miller^74^; Conklyn *et al*^46^; Helmlinger *et al*^53^; Seebacher *et al*^51^; Seebacher *et al*^50^; Seebacher *et al*^59^; Seebacher *et al*^49^; Seebacher *et al*, 2015^55^; Shahraki *et al*, 2017).^42^† 3 non-randomised experimental studies (Maggio *et al*^54^; Moumdjian *et al*^75^; Naderi *et al*).^55^ One cross-sectional study (Goetschalckx *et al*).^57^ Two case-control studies (Moumdjian *et al*,^58^ Moumdjian *et al*).^76^ 2 case studies.

All included studies from these previous reviews were included in our review with the following exceptions: (1) case studies, case reports, case series, conference abstracts, chapters and non-peer-reviewed sources were not eligible publication types for our review; (2) Baştürk *et al*^73^; Ng *et al*^71^ and Van Geel *et al*^72^ were both dance intervention studies which were not eligible for our review because the intervention was not primarily music-based; Baram and Miller^74^ is about auditory feedback control but makes no mention of music and is therefore not eligible for our review; Moumdjian *et al*^75^ ^76^ were excluded because they examined movement synchronisation to music or metronome without assessing rehabilitation or clinical outcomes in people with MS. We have checked (May 2026) and the Baştürk *et al*^73^ ‘online ahead of print’ paper from 2024 has not yet been published in its final version.

*The citation for Thaut *et al*^61^ was incorrect in the bibliography of the cited review but appears to refer to this study.

†The review states eight RCTs but provides nine citations—it would appear that two papers are being referred to as parts of the same study, but this is not made clear.

MBTs, music-based therapies; MS, multiple sclerosis; MT, music therapy; QoL, quality of life; RCT, randomised controlled trial.

### Rationale

Existing systematic reviews are fragmented and limited in scope. Available reviews either focus narrowly on gait and walking-related outcomes,^25 28^ only include a small number of heterogeneous studies,^20 27^ or provide findings from multiple neurological conditions without MS-specific conclusions.^24^ Reviews of related performing arts modalities, for example, dance, highlight potential motor and psychological benefits for people with MS, yet these interventions do not isolate the therapeutic contribution of the musical component.^4^ There is no recent systematic review of the potential benefit of MBTs across a broad range of rehabilitation outcomes relevant to people with MS. No existing review provides a comprehensive synthesis of the impact of MBTs on motor, cognitive, psychological or QoL domains, nor evaluates the diversity of interventions used.

Considering specifically reviews published in 2025, here is a clear statement of how they differ from our systematic review and the advance to knowledge our work offers:

- Gardener *et al*^24^: This systematic review has a specific focus on MT and psychological outcomes; our review considers MBTs and mobility, cognitive function and QoL outcomes, therefore, offering broader intervention and outcome measure coverage.
- Phillips *et al*^27^: This systematic review considers ‘music interventions’, mode of engagement (active vs receptive interventions) and ‘balance’, ‘gait’, ‘anxiety’, ‘depression’ and ‘fatigue’. Our review explicitly uses the MBTs framework to define eligibility, includes mobility and psychological outcomes, and includes comparison of MBTs and control arms.
- Tan *et al*^28^: This is a scoping review, while our review is a systematic review. Systematic reviews are considered a higher level of evidence compared with scoping reviews and offer a structured assessment of risk of bias.^33^ This review considers ‘music interventions’ but does not use an explicitly defined framework such as MBTs, while our review explicitly uses the MBTs framework to define eligibility. This review limits its outcomes to gait and mobility, while our review also considers cognitive function, psychological well-being and QoL.

Therefore, our review provides a clear advance in knowledge in terms of coverage of interventions and outcomes. We provide the first systematic review to provide an assessment of the potential benefit of music in MS using the MBTs framework to ensure appropriately broad intervention coverage and assessment of mobility, cognitive function, psychological outcomes and QoL outcomes.

### Aims

The aim of our work was to provide a systematic review of the evidence for a benefit of MBTs on rehabilitation outcomes in adults with MS. In particular, the primary review question was:

- What is the impact of MBTs on rehabilitation outcomes in people with MS?

Secondary review questions were:

- What types of MBTs have been studied for their rehabilitation potential for people with MS?
- What specific rehabilitation domains (eg, motor, cognitive, psychological and QoL) show the greatest response to MBTs in people with MS?

## Methods

### Design

A systematic review was conducted following the Preferred Reporting Items for Systematic Reviews and Meta-Analyses (PRISMA) 2020 guidelines.^34^ Completed PRISMA and PRISMA for Abstracts checklists are provided in online supplemental files 1 and 2. Reporting of the narrative synthesis was compliant with the Synthesis without meta-analysis (SWiM) guidelines.^35^ This systematic review was registered on the PROSPERO database (CRD420251083656). One protocol amendment was made—to use risk of bias tools from the same suite for all study designs.

10.1136/bmjopen-2026-116420.supp1 Supplementary data

### Data sources

AMED (Ebsco), APA PsycINFO (Ovid), CINAHL (Ebsco), Cochrane-CENTRAL, EMBASE (Ovid) and MEDLINE (Ovid) were searched from inception until July 2025. Supplementary searches were conducted on Google Scholar and through forward and backward citation chasing on studies identified for full-text review up to November 2025. Searches were designed to retrieve articles on MS and MBTs (strategies for all databases are shown in online supplemental file 3) and were designed by NA and MSB and conducted by the lead author NA. MSB conducted supplementary searches and citation chasing.

### Inclusion criteria

Screening was initially conducted based on the title and abstract. Potentially relevant articles were screened at the full-text stage to determine inclusion (online supplemental file 4) or exclusion (online supplemental file 5) in the systematic review. Screening was initially conducted by NA based on titles and abstracts, followed by full text screening of potentially eligible studies. A second reviewer (MSB) independently screened all records to verify consistency, and any disagreements were resolved through discussion.

Eligibility criteria are shown in box 1. The outcomes of interest were rehabilitation-related outcomes: functional recovery, motor function, gait and balance, cognitive function, psychological well-being and QoL. Studies with and without control arms were eligible. MBTs were defined using the operational definition proposed in the Introduction: ‘structured musical activities designed to enhance well-being, reduce stress and improve QoL, without necessarily focusing on clinical diagnosis, assessment, or long-term psychotherapy’. As MT falls within this definition of MBTs, studies on MT were eligible for inclusion. There were no specific requirements for what control arms should involve. Therefore, control arms could include alternative populations, such as healthy controls or people with another medical condition rather than MS, in addition to alternative interventions. While no limits were set on the types of control arms that were eligible, based on previous systematic reviews on the performing arts in neurological settings^8 9^ and knowledge of the field, it was anticipated that control arms may include exercise, physiotherapy, usual care (or no intervention), conventional cognitive rehabilitation and other MBTs. Screening was conducted using Rayyan, which uses artificial intelligence, machine learning and natural language processing to make systematic reviewing more efficient by learning from user decisions to predict article relevance and highlight key PICO terms. All screening was done manually by the two human reviewers.

Box 1 **Inclusion criteria for the systematic review**

Eligible studies assessed:

- Population: People with a diagnosis of multiple sclerosis (MS)
- Intervention: Studies involving music-based therapies (MBTs) or music interventions delivered for the purpose of rehabilitation in people with MS. Interventions could include:
  - Active music-making (eg, instrument playing).
  - Passive listening to music (prerecorded or live).
  - Rhythmic auditory stimulation.
  - Structured music therapy delivered by a trained therapist.
  - Group or individual sessions of MBTs.
- Control arms: Studies with and without control arms were eligible. There were no specific requirements for what control arms should involve.
- Outcomes: Studies that report rehabilitation-related outcomes: functional recovery, motor function, gait and balance, cognitive function, psychological well-being, quality of life (QoL).
- Other: Studies available in full-text and published in the English language involving human participants aged 18 and over with a confirmed diagnosis of MS. Studies conducted in any healthcare, community or rehabilitation setting, provided they meet the control arm and outcome criteria outlined above.

Given the emergent evidence base, we included studies that provided data that were informative for the listed outcome domains but did not cover the full breadth of the domain. For example, the MSFC scale covers cognition but is not exclusively on cognition but was considered relevant. Furthermore, studies addressing pain, general fatigue and QoL subscales rather than a formal QoL scale that covers the full breadth of QoL were considered relevant.

### Data extraction

Information extracted is shown in box 2. All data extraction processes were conducted by NA, and MSB independently reviewed all records to verify consistency. Any disagreements were resolved through discussion. No automation tools were used. The appendix provides additional information on study characteristics (online supplemental file 6), interventions (online supplemental file 7), controls (online supplemental file 8) and narrative results (online supplemental file 9).

Box 2 **Data extracted**

The following information was extracted for each included study:

- Bibliographic details (authors, year and citation).
- Country of study.
- Study design.
- Participants (sample size, sex profile, mean age or age range, multiple sclerosis type, disease duration, Expanded Disability Status Scale score, use of walking aids, left/right-handed—as this variable is often considered in neuropsychological studies).
- Inclusion criteria.
- Outcomes.
- Content of intervention.
- Professional background of intervention leader—as work in Parkinson’s disease^8^ has shown large differences in leader background and expertise within and between studies.
- Location of intervention (eg, health centre or home).
- Frequency and duration of intervention.
- Content of control arm.
- Professional background of leader of intervention delivered to the control arm.
- Location of control arm (eg, health centre or home).
- Frequency and duration of control arm.
- Study results for narrative synthesis for all eligible reported outcomes.

### Risk of bias assessment

Risk of bias and methodological quality were assessed using the relevant Critical Appraisal Skills Programme (CASP) checklists for RCTs, cohort studies, case control studies and cross-sectional studies.^36^ The descriptive, qualitative approach to CASP was used, whereby we tabulated and described the patterns of bias both within and between studies but did not use a numerical scoring system or assign overall ‘high’, ‘moderate’ and ‘low’ quality labels to studies. Avoidance of the use of numerical composite scores for risk of bias is in line with recommendations from the Agency for Healthcare Research and Quality^37^ in the USA. Quality appraisal was conducted independently by NA, with MSB reviewing all studies to ensure consistency. Disagreements were resolved through discussion. No automation tools were used. The appendix provides additional information on risk of bias checklists used (online supplemental file 10).

### Narrative synthesis

A thematic narrative synthesis was used to analyse all studies that met the inclusion criteria. Guidance recommends^38^ that the decision whether to undertake a meta-analysis should be pre-specified based on clear a priori justification and conceptual understanding rather than decided post hoc based on formal evaluation of identified studies. In all previous systematic reviews in the field, meta-analysis was not considered appropriate due to substantial methodological heterogeneity across eligible studies. These sources of heterogeneity included: (1) study design (including RCTs and observational studies), (2) differences in type and delivery of MBTs (including active and receptive MBTs), (3) differences in disciplinary backgrounds of intervention leaders (including music therapists, other health professionals and professional performing artists), (4) differences in session length, frequency and duration and (5) differences in outcome measurement (both conceptual differences and differences in terms of what assessment tools and scales were used). In previous systematic reviews in the field, these factors were considered to preclude the forming of meaningful meta-analysis sets comprising sufficient studies assessing the same combination of intervention, control and outcome. This precedent from previous systematic reviews was the key factor in our a priori decision that conducting meta-analysis was not feasible. Additionally, we conducted a qualitative post hoc assessment of whether these issues were also present in the evidence base for our systematic review to confirm the appropriateness of our a priori decision. This showed that the same issues with conducting meta-analysis were present in the evidence base we identified as in the previous systematic reviews, supporting our decision to conduct exclusively narrative synthesis.

To conduct narrative synthesis, initially the narrative study results extracted during data extraction were reviewed to identify thematic commonalities and patterns in the data. This process identified four key outcome themes in the data: (1) motor function, (2) cognitive function, (3) psychological outcomes and (4) QoL. The narrative synthesis was initially stratified by outcome using these four outcome domains. All studies that addressed a particular outcome domain were included in the synthesis for that outcome. Within each outcome domain, the synthesis was further stratified by the type of MBTs assessed. This could include rhythmic auditory stimulation (RAS), receptive music listening, active music making and structured MT interventions. However, the types of MBTs were not imposed on the synthesis a priori to allow these to emerge thematically from the data within each outcome domain. As a third level of stratification, within each type of MBTs for each outcome domain, evidence was stratified by interventional and observational study designs, also noting the evidence specifically available from RCTs. Our approach to narrative synthesis enabled comparison across rehabilitation domains and facilitated evaluation of how different MBTs contribute to rehabilitation outcomes in people with MS. For all outcomes, the primary standardised metric for narrative synthesis—based on the SWiM guidelines^35^—was direction of findings to provide an effective overall synthesis of the patterns of findings, while noting considerable differences in how outcomes were measured and analysed across studies. The potential of small-study bias was assessed using an effect direction plot.^39^ The potential of outcome reporting bias was assessed by comparing outcomes in published protocols or clinical trials registries with the outcomes in the journal publications. Heterogeneity could not be assessed quantitatively due to the differences in outcome assessment that precluded meta-analysis. However, differences in study designs, study characteristics, interventions and comparators were profiled in online supplemental files 6–8 and reflected on at appropriate points in the results and discussion. The textual write-up of the narrative synthesis was supplemented by a detailed results table (online supplemental file 9). The table was ordered alphabetically, while the textual write-up was structured according to the stratification factors stated above (outcome, type of MBTs and study design). The synthesis was initially conducted by one author (NA) and then reviewed, checked and revised by a second author (MSB).

### Clinical significance

The approach to assessing clinical significance was prespecified. Searches were conducted to identify minimally clinically important differences (MCIDs)^40^ for each of the key thematic areas in the narrative synthesis: (1) motor function, (2) cognitive function, (3) psychological outcomes and (4) QoL. There was heterogeneity in the outcome measures used to assess these outcomes across studies. In assessing MCIDs,^41^ it is considered best practice to evaluate only outcomes used in multiple studies (typically three or more studies) to ensure validity, stability and consensus. Therefore, we conducted MCID assessment on outcomes used on at least three studies included in our review. Furthermore, only outcomes that are on measurement scales amenable to MCID assessment and for which a published MCID can be identified could be included.^42^ The distinction between within-group and between-group differences was considered in the reporting and in interpretation of MCID results. The MCIDs identified and applied are shown in table 2.

**Table 2:** Minimally clinically important differences (MCIDs) identified

Outcome domain	Measure	Number of studies	MCID
Motor	Timed 25-foot walk test	6	MCID of a 17.2% improvement in test performance has been proposed based on trial data,^77^ which accords well to previous clinical proposals of a 20% improvement
Cognitive	N/A	N/A	No pivotal measures used in at least three studies identified
Psychological	Beck depression	3	No MS-specific MCID identified, however noting clinical significance depends on baseline depression severity, a population-agnostic MCID of a 17.5% reduction from baseline has been proposed^78^
Psychological	HADS anxiety	5	No MS-specific MCID identified, however an MCID of 1.70 points^79^ has been found for HADS told in cardiovascular disease and 1.60 points^80^ in chronic obstructive pulmonary disease. Halving for the anxiety subscale alone gives an MCID of 0.80–0.85
QoL	MS Impact Scale-29	3	No overall MCID identified,^81^ although an MCID of 8 points has been proposed for the physical subscale^82^

MS, multiple sclerosis; N/A, not available; QoL, quality of life.

### Certainty assessment

We conducted certainty assessment using Grading of Recommendations Assessment, Development and Evaluation (GRADE) for each outcome domain supported by the narrative synthesis. As GRADE methods are currently being updated, we used the updated GRADE Book^43^ for chapters that had been updated and the 2013 GRADE Handbook^44^ for the remaining chapters, as recommended in the GRADE BOOK.^43^ We refer to both books below as ‘the GRADE guidance’. We describe the approach concisely below. In the GRADE guidance, evidence from RCTs initially starts as high certainty evidence, while evidence from non-randomised and observational studies starts as low certainty evidence. For RCTs, the risk of systematic or random errors may reduce confidence in the findings. Four GRADE domains assess the risk of systematic errors: (1) study limitations (risk of bias), (2) inconsistency, (3) indirectness and (4) dissemination bias (publication bias). As noted in the limitations section of the discussion, it was not possible to formally assess publication bias in this systematic review. One GRADE domain evaluates the risk of random error: (1) imprecision. An RCT is downgraded one level from high certainty for every domain for which there are serious concerns, or by two levels if there are very serious concerns. Non-randomised and observational studies may be upgraded from low certainty evidence where the risk of confounding variables is relatively low (eg, due to large effect sizes or dose-response gradients) or where the presence of a confounding variable strengthens the inference of effect. Detailed operationalisation criteria are found in the GRADE guidance. We followed the GRADE guidance that the starting point for an outcome domain shall be set as high if the body of evidence consists primarily of RCTs and low if it does not consist primarily of RCTs. The evidence is profiled in table 3 along with explanations for the GRADE scoring decisions—the presentation of GRADE results is consistent with Barnish *et al*,^8^ although we include this information in the main manuscript rather than the supplemental material for clarity. It was determined that the different forms of MBTs in the evidence base did not form sufficiently clear and distinct categories to conduct separate GRADE assessments by type of MBTs.

**Table 3:** GRADE scoring and explanation

Outcome domain	GRADE	Explanation
Motor	Low	16 studies, of which 10 RCTs (including feasibility RCTs but excluding secondary analysis of RCT data). Starting GRADE High as most of the evidence is randomised (as per GRADE guidance). Downgrade to moderate because of heterogeneity. Downgrade to low because the set of studies included evidence considered to be low quality.
Cognitive	Low	8 studies, of which 5 RCTs. Starting GRADE High as most of the evidence is randomised (as per GRADE guidance). Downgrade to moderate because of heterogeneity. Downgrade to low because the set of studies included evidence considered to be low quality.
Psychological	Low	7 studies, of which 4 RCTs. Starting GRADE High as most of the evidence is randomised (as per GRADE guidance). Downgrade to moderate because of heterogeneity. Downgrade to low because the set of studies included evidence considered to be low quality.
QoL	Low	12 studies, of which 8 RCTs. Starting GRADE High as most of the evidence is randomised (as per GRADE guidance). Downgrade to moderate because of heterogeneity. Downgrade to low because the set of studies included evidence considered to be low quality.

GRADE, Grading of Recommendations Assessment, Development and Evaluation; QoL, quality of life; RCT, randomised controlled trial.

### Patient and public involvement

Patient and public involvement could not be conducted for this systematic review assessing a broad range of MBTs due to a lack of funding. The corresponding author will respond to any reputable media enquiries.

## Results

### Search results

**Figure 1:**
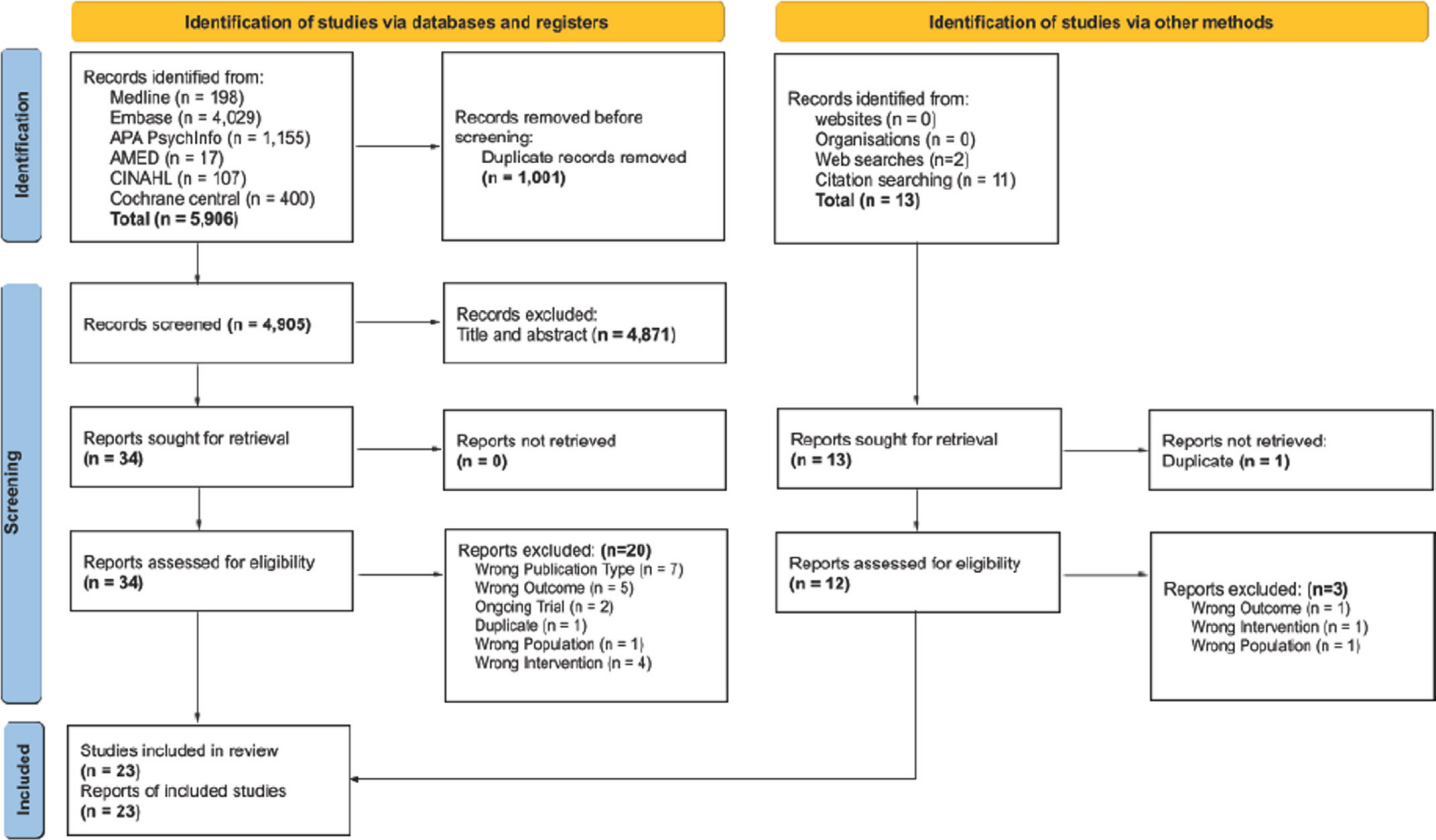
Preferred Reporting Items for Systematic Reviews and Meta-Analyses (PRISMA) 2020 flow diagram. *Consider, if feasible to do so, reporting the number of records identified from each database or register searched (rather than the total number across all databases/registers). **If automation tools were used, indicate how many records were excluded by a human and how many were excluded by automation tools. Source: Page *et al*.^34^ This work is licensed under CC BY 4.0. To view a copy of this license, visit https://creativecommons.org/licenses/by/4.0/

Database searches returned a total of 5906 records (AMED 17, PsycINFO/APA PsycINFO 1155, EMBASE 4029 and MEDLINE 198, CINAHL 107, Cochrane central 400), plus 13 from supplementary searches. A total of 4918 records proceeded to title and abstract screening (including one duplicate). 47 unique records (including one duplicate) were assessed at full-text screening; 23 studies were included in the systematic review (figure 1).

### Study profile

Of the 23 included studies, there were 20 interventional studies (14 RCTs, 2 feasibility RCTs, 2 quasi-experimental controlled feasibility studies, 1 secondary analysis of an RCT and one matched controlled pilot study) and three observational studies (one observational cross-sectional comparative study, one case–control study, and one matched control study, details in online supplemental file 6). Six included studies (26%) described their approach as MT or the session leader as a music therapist (details in online supplemental file 7). Among the remaining 17 studies, 6 used gait training or motor imagery, 5 used rhythmic auditory stimulation, three used sung word list tasks, 1 used instrument playing, 1 used listening to music and 1 used music to movement (M2M).^45^ Three studies (13%) had no control arm. A further four studies (17%) had no control arm comprising people with MS—two of these had a ‘healthy’ control arm who received the MBTs intervention and two had a ‘healthy’ control arm who received no intervention. 16 studies (70%) had a control group with MS; in seven studies the control was no intervention, in three studies it was learning spoken word lists, in three studies it was a ‘placebo’ or sham intervention (playing a keyboard that was switched off, lying on top of a monochord that is not being played, or doing the same exercises without rhythmic auditory stimulation), in two studies it was conventional cognitive rehabilitation, and in one study it was receptive music activities—listening to music, discussing music and learning about music and composers (details in online supplemental file 8). No studies compared MBTs to movement interventions (eg, exercise or physiotherapy).

The included studies comprised 955 participants, of whom 880 had MS and 75 did not. Across all studies, 691 participants received MBT and 264 did not receive MBT and were therefore classified as control participants. Of the 75 participants without MS, 58 received MBT and 17 did not receive MBT. Therefore, the MBT and control totals include both participants with MS and participants without MS, depending on the study design. Sex was incompletely reported; where data were available, 666 participants (75.0%) were female and 222 (25.0%) were male. Reported mean ages across studies generally ranged from the mid-40s to early 50s, with overall participant ages spanning approximately 29 to 68 years. Disease duration was inconsistently reported, but where available typically ranged from approximately 7 to 18 years since diagnosis, with similar durations between intervention and control groups within individual studies. Baseline disability levels were most commonly reported using the Expanded Disability Status Scale (EDSS). Across studies reporting EDSS, participants generally had mild to moderate disability, with median or mean scores most frequently ranging between 2.0 and 4.5. No studies directly compared different MBI types with each other. MBTs assessed covered both active and passive forms of MT (instrumental and vocal) as well as other MBTs such as musically and rhythmically based cueing, and rhythmic auditory stimulation. No studies assessed choral singing or community singing groups. Further details, including on the types of MBTs assessed, are provided in online supplemental file 10.

### Narrative synthesis

As there are 23 included studies, an overview of the evidence landscape is provided in table 4. Several factors appeared to influence intervention effectiveness. Positive motor outcomes were most consistently reported in studies using rhythmic auditory cueing, RAS, and music-cued motor imagery. Benefits were generally observed in walking-related outcomes; however, effects on fatigue, QoL, and psychological outcomes were more variable. In terms of contextual moderators, variations in intervention type (active vs passive), cueing method, intervention duration, baseline disability level, and sample size may have contributed to differences in outcomes across studies.

**Table 4:** Distribution of included studies according to rehabilitation outcome domains

First author	Motor outcomes	Cognitive outcomes	Psychological outcomes	QoL outcomes
Aldridge *et al*^62^	–	Global cognitive function	Depression, anxiety, self-acceptance	QoL, functional disability, fatigue
Conklyn *et al*^46^	Gait and mobility; muscle strength; spasticity and movement related disability	–	–	Pain; functional disability
Gatti *et al*^47^	Upper limb function	–	–	–
Goetschalckx *et al*^57^	Balance and stability; coordination and synchronisation	–	–	–
Helminger *et al*^53^	Gait and mobility	–	–	–
Impellizzeri *et al*^32^	–	Global cognitive function	Mood and emotional processing	MS specific QoL
Kuhlmann *et al*^63^	–	–	Anxiety; depression; body perception	Fatigue; pain
Maggio *et al*^54^	Gait and mobility; balance and stability	–	Depression; emotional components	MS specific QoL
Moore *et al*^48^	–	Learning and memory; executive function and attention	–	–
Moumdjian *et al*^58^	Gait and mobility; coordination and synchronisation	Cognitive fatigue	–	Fatigue
Naderi *et al*^55^	Gait and mobility	–	–	–
Schmid and Aldridge^30^	Spasticity and movement related disability	Cognitive function	Depression; anxiety; self-acceptance	QoL; functional disability
Seebacher *et al*^56^	Gait and mobility	–	–	Fatigue
Seebacher *et al*^49^	Gait and mobility	–	–	QoL; fatigue
Seebacher *et al*^59^	Gait and mobility	–	–	QoL; fatigue
Seebacher *et al*^50^	Gait and mobility; coordination and synchronisation	–	–	QoL; fatigue
Seebacher *et al*^51^	Gait and mobility	Global cognitive function	Anxiety; depression; suicidality	QoL; fatigue
Seighalani *et al*^64^	–	–	Depression; anxiety; self esteem	–
Shahraki *et al*^52^	Gait and mobility	–	–	–
Thaut *et al*^60^	–	Learning and memory	–	–
Thaut *et al*^61^	–	Learning and memory; neural synchronisation	–	–
Wiens *et al*^31^	Muscle strength (respiratory)	–	–	–
Young *et al*^45^	Gait and mobility	–	–	Fatigue; pain

MS, multiple sclerosis; QoL, quality of life.

#### Motor outcomes

Motor outcomes were assessed in 16 studies (70%)—profiled in online supplemental files 4–6. 14 studies were interventional—of which 9 were RCTs,^31 45–52^ and 5 were other interventional designs (secondary analysis of RCT, quasi-experimental controlled feasibility study and feasibility RCT)^49 53–56^—and two were observational studies.^57 58^

One study^31^ (an RCT) looked at active MT and showed that numerically people with MS receiving active MT improved in terms of the strength of their respiratory muscles, while those in the passive music engagement group showed a numerical decline, although the between-group difference did not reach statistical significance. Active music making (keyboard playing)—not led by a music therapist—was associated with a statistically significant improvement (p=0.003, Cohen’s d=1.66, large effect) in hand function compared with playing with the keyboard switched off in one study^47^ (an RCT). However, there was no between-group difference in hand dexterity or hand strength. M2M—which is a specific type^45^ of active music intervention that is not led by a music therapist—showed a statistically significant difference compared with no active music intervention or yoga on two mobility outcomes (timed Up and Go, p=0.01, Cohen’s d=0.7, moderate effect; 6 min walk test (p=0.05, Cohen’s d=0.6, moderate effect) in one study^45^ (an RCT), although no statistically significant difference was found for five times sit-to-stand.

There were three RCTs^49–51^ on motor imagery, all conducted by the same research group. All music-based groups showed statistically significant improvements in walking in all three studies, but there were no statistically significant differences between the different music-based groups. One study^51^ showed synchronisation improved more with music and verbal motor imagery and music-cued motor imagery and worsened with just motor imagery. While one study^51^ showed that physical fatigue only showed a statistically significant improvement after music and verbally cued motor imagery, another study^49^ showed that this was found only for music-cued imagery. Studies using other interventional designs partly corroborated these findings—showing improved walking speed and distance after all music interventions^56 59^ and reduced physical fatigue after music and verbally-cued motor imagery,^59^ although one study only showed improvements in walking tests for those classed as ‘responders’, while response was associated with changes in brain activation patterns.^53^ There were no observational studies for this intervention-outcome combination.

There were two RCTs^46 52^ that assessed rhythmic auditory stimulation in relation to motor outcomes, both showing improvements in at some gait parameters. In one study, significant improvements were observed in double-support time, while additional parameters including velocity, cadence, stride length and step length consistently favoured the intervention group and were associated with moderate effect sizes despite not reaching statistical significance.^46^ There was one study^55^ using a different intervention design, which corroborated the findings from RCTs in terms of improvements in gait parameters versus no rhythmic stimulation. There was one observational study,^57^ which showed an effect of frequency of rhythmic auditory stimulation on interlimb coordination (0.75 Hz was worse than either 1.00 Hz or 1.50 Hz) with higher cognitive processing speed and attention scores were associated with better coordination.

#### Cognitive outcomes

Cognitive outcomes were assessed in eight studies (35%)—profiled in online supplemental files 4–6. Seven studies were interventional—of which five were RCTs^32 48 51 60 61^ and two were other interventional designs^30 62^—and one was an observational study.^58^

Specifically using neurologic MT methods, one study^32^ (an RCT) showed statistically significant improvements in cognitive function (p<0.001), long-term retrieval (p=0.007) and delayed recall (p=0.001) in the MT group. One study^54^ (using a different interventional design) on passive music listening (not led by a music therapist) showed statistically significant within-group effects in the music group on cognitive function (p<0.001) and long-term retrieval (p<0.007). There was no statistically significant difference versus conventional cognitive rehabilitation. It should be noted that conventional cognitive rehabilitation was only administered in the control arm for this study.

One study^58^ (observational) assessed rhythmic auditory stimulation and found that people with MS perceived statistically significantly lower cognitive fatigue (p<0.001) while listening to music.

#### Psychological outcomes

Psychological outcomes were assessed in seven studies (30%)—profiled in online supplemental files 4–6. Six studies were interventional—of which four were RCTs^32 51 63 64^ and two were other interventional designs^54 62^—and one was an observational study.^30^

There was one RCT^64^ that assessed active MT and showed that—from pretest to post-test to follow-up, MT statistically significantly reduced depression and anxiety and improved self-esteem. One study^62^ using a different interventional design did not find statistically significant differences versus control but did show medium effect sizes for improvements in self-esteem (Cohen’s d=0.54), depression (Cohen’s d=0.64) and anxiety (Cohen’s d=0.63). One observational study reported significant within-group improvements in depression (p=0.036), anxiety (p=0.013) and self-acceptance (p=0.012) following MT; however, no significant differences were observed compared with the control group.^30^ One study^63^ (an RCT) assessed passive MT (listening to a monochord while lying on it) and showed statistically significant benefits for MT versus a silent condition lying on the monochord while it is not being played on psychosocial fatigue (p=0.029) and stronger immediate subjective effects (p<0.001), although the numerical improvement in anxiety outcomes did not reach statistical significance (p=0.109). One study^54^ (using a different interventional design) showed statistically significant within-group improvements for passive music listening (not led by a music therapist) on motivation, emotional awareness and depression. There were no statistically significant differences versus conventional cognitive rehabilitation, which was only administered in the control arm.

#### QoL outcomes

QoL-related outcomes were assessed in 12 studies (52%)—profiled in online supplemental files 4–6. 10 studies were interventional—of which six were RCTs^32 45 49–51 63^ and four were other interventional designs^54 56 59 62^—and two were observational.^30 58^

One RCT found that adding neurologic MT to conventional cognitive rehabilitation was associated with greater improvement in the mental health component of the MSQoL-54 (p<0.001) than conventional cognitive rehabilitation alone.^32^ One interventional study^62^ (using a different interventional design) and one observational study^30^ of active MT reported no significant improvement in QoL compared with a matched control group. Evidence for QoL and well-being outcomes was mixed. Studies assessing QoL directly reported variable findings, with significant improvements observed following passive music listening^63^ and music-assisted gait training,^54^ whereas active MT studies found no significant improvements in overall QoL.^30 62^ Studies assessing related outcomes such as fatigue and pain interference also produced mixed findings, reporting a trend towards reduced fatigue but no significant effect on pain interference.^45^

Three RCTs assessed motor imagery.^49–51^ In two RCTs,^49 50^ QoL showed a statistically significant improvement only after music and verbally cued motor imagery. A smaller feasibility study^59^ using a different interventional design provided preliminary evidence to support a benefit on QoL.

#### Summary of synthesis and risk of bias

Overall, current evidence suggests that MBTs may provide benefits across motor, cognitive, psychological and QoL domains in people with MS. However, heterogeneity in intervention type, outcome measures, control arms and study design limits the strength of conclusions that can be drawn, and several outcomes were assessed in only a small number of studies. The risk of bias profile as well as the potential impact of risk of bias on the outcomes of the narrative synthesis are presented in online supplemental file 10. Potential outcome reporting bias was not evaluable in 13 studies (57%) as no protocol was available. In five studies (22%), all outcomes from the protocol were reported in the journal article and in one further study all primary outcomes were reported. In two studies (9%), primary outcomes from the protocol were not reported in the journal article, while in two further studies (9%), the primary outcomes from the protocol were reported in the journal article but were switched to secondary outcomes. Further details can be found in online supplemental file 11. The effect direction plot (online supplemental file 12) shows that all studies were classed as small or medium (as there were no studies with a sample size more than 300); however, there was no evidence that the results of studies differed materially dependent on sample size.

#### Clinical significance

Available evidence consistently supported a clinically significant benefit of MBTs on depressive symptoms as assessed by the Beck depression scale. However, less consistent evidence of a clinically significant benefit was found for motor function assessed by the timed 25-foot walk test and QoL assessed by the MS Impact Scale-29 physical subscale. Meanwhile, the only evaluable study for anxiety assessed by HADS-anxiety subscale did not find evidence of a clinically significant benefit. Further details can be found in online supplemental file 13.

#### Certainty assessment

Certainty of evidence was assessed as Low for all outcome domains using the GRADE schemata, reflecting potential issues around heterogeneity and the presence of studies where multiple CASP domains were assessed at potential high risk of bias, although most included studies for each outcome domain were RCTs. All studies assessed within-group rather than between-group differences, although for some studies information was available for more than one group.

## Discussion

### Summary

This systematic review synthesised evidence on MBTs for rehabilitation outcomes in people with MS, identifying 23 eligible studies published between 1999 and 2025. The value of a systematic review comes in the number of studies integrated in the same systematic review rather than how many studies had featured in any previous systematic review.^65^ No previous systematic review had included more than 12^26 27^ of the 23 studies (52%) that we identified. Indeed, we double the amount of evidence included in the most recent related review by Phillips *et al*^27^ By doubling the amount of evidence on MBTs in MS that has been integrated together compared with any previous systematic review, we offer a clear advance in knowledge. Furthermore, the present review adds an assessment of clinical significance and certainty of findings. Across the 23 studies, outcomes could be mapped to four rehabilitation domains, with motor outcomes most frequently assessed (16 studies), followed by QoL and related outcomes (12 studies), cognitive outcomes (8 studies), and psychological outcomes (7 studies). The most evidence among motor outcomes was found for gait and mobility, particularly where interventions used rhythmic auditory stimulation and music cued motor imagery, with improvements reported in walking speed, walking distance, cadence, stride length and gait symmetry. Cognitive findings were mixed, with clearer benefits in music assisted learning paradigms (eg, sung vs spoken word learning), while broader cognitive rehabilitation comparisons suggested similar rather than superior benefits relative to conventional rehabilitation. Psychological outcomes showed potential improvements in depression, anxiety, self-acceptance and self-esteem, but between group effects were not uniform. QoL and fatigue were frequently co-assessed, with several trials reporting improvements in fatigue and MS-specific QoL measures, though findings were heterogeneous. Importantly, this review incorporated studies that have been less emphasised in prior summaries and were necessary to include for a complete synthesis. Challenges were identified in terms of certainty of evidence as assessed by GRADE and reaching clinical significance thresholds across the sample for some outcomes. These may reflect the emerging nature of the evidence base for MBTs in MS, the arts in health more generally, and the methodological challenges associated therewith.

### Interpretation of findings

This review extends and updates the MS rehabilitation literature on MBTs by both increasing the quantity of included evidence and the scope of interventions and outcomes compared with prior syntheses. A key interpretive issue is the distinction between active and receptive MBTs. The evidence base remains weighted toward receptive approaches, particularly music listening during walking or cueing based strategies. Phillips *et al*^27^ concluded that most included interventions were receptive, with fewer active interventions, and highlighted gait parameters and fatigue as the most common outcomes. The findings in the present review corroborate that pattern using a much larger evidence base but also suggest that the active vs receptive categorisation favoured by Phillips *et al*^27^ may be too simplistic for MS rehabilitation. Phillips *et al*^27^ searched from 2002 to June 2024 and included 15 studies,^27^ three of which did not meet the inclusion criteria for our review as the interventions were not MBTs (see table 1 for details). Furthermore, Phillips *et al*^27^ (due to their 2002 search date limit) excluded key earlier work^19^ as well as not identifying studies published since their June 2024 search date. The inclusion of Kuhlmann *et al*^63^ in our review provides additional randomised evidence on receptive vibration-based therapy effects on fatigue and pain perception, while Helmlinger *et al*^53^ contributes neuroimaging correlated outcomes that are not represented in reviews with earlier search cut-offs. While group singing-based interventions were shown to be common in PD,^8^ no evidence on these types of MBTs was identified in our review on MS. Across outcomes, evidence from our review suggests the greatest benefit for gait and mobility, with more heterogeneous findings for cognition and psychological outcomes. This likely reflects differences in intervention intent and dose, diversity in control arms and inconsistent outcome measures. While there were multiple studies by the same authors,^49–51 53 56 59^ they were separate studies, and except for one pair of studies that had a small overlap in sample (3%), it did not appear that there were overlapping participants (online supplemental file 11). However, comparability of findings across studies may be limited by differences in control arms across studies, meaning that MBTs are not always being compared with interventions of equivalent effectiveness. A further limitation of the evidence base was that the follow-up assessment typically took place shortly after the intervention, meaning we lack information about long-term benefit. This is a common limitation with clinical trials and could be addressed through observational registry studies.^66^ As sample sizes were typically small, there is a possibility of findings being affected by small-sample bias. Furthermore, arts interventions are difficult to blind and therefore blinding is seldom conducted in research on the arts in health.^5 8 9^ Therefore, the possibility of performance bias due to non-blinded interventions should be considered. Overall, based on the available evidence, MBTs appeared promising across multiple rehabilitation domains in MS, but the evidence remains constrained by intervention heterogeneity, limited head-to-head comparisons of types of MBTs and variable methodological reporting, which together limit the certainty of any single conclusion.

### Strengths and limitations

This systematic review has several strengths. We used a comprehensive search strategy across multiple databases, followed PRISMA reporting standards^34^ and applied predefined eligibility criteria. The final search was conducted in July 2025, within 6 months of original manuscript submission (January 2026), which strengthens the currency of the evidence base.^67^ The review adhered to PRISMA^34^ reporting standards and applied predefined eligibility criteria, reducing the risk of selection bias. Data extraction was conducted using standardised forms, and risk of bias was assessed using design-appropriate CASP tools,^36^ supporting consistency across heterogeneous study designs. The review also incorporated assessments of clinical significance and certainty of evidence, allowing interpretation beyond statistical significance alone.

However, limitations should be acknowledged. First, meta-analysis was not feasible because of substantial heterogeneity in study design, intervention type, comparator condition, intervention dose and outcome measurement. As a result, the synthesis relied on narrative methods, which are less precise than quantitative pooling. Second, many included studies had small samples and were exploratory or pilot in nature, limiting the statistical power. Third, although many studies were randomised, blinding of participants and intervention providers was generally not possible because of the nature of MBTs, increasing the potential for performance bias. Also, participant characteristics such as race and ethnicity were not consistently reported, limiting the assessment of generalisability. Finally, only studies published in English were eligible for inclusion; however, no studies were excluded on grounds of language of publication, as no studies published in languages other than English that would otherwise be eligible for inclusion were identified. This may reflect limitations^68^ in indexing of non-English language journals in major bibliographic databases. It is also noted^69^ that subscription coverage of non-English language journals in university libraries in English-speaking countries is limited, which would limit access to full texts of non-English language articles if they had been eligible for inclusion. It was not possible to assess publication bias as the data for most outcomes were not amenable to such aggregation—for many of the same reasons as meta-analysis could not be conducted, including differences in sampling frames and differences in how outcomes were assessed. The groupings in the narrative synthesis were broad and may not reflect granular differences in outcome and intervention types—this was necessary because of the relatively low (n=23) number of total studies.

### Implications for research and practice

Future research should prioritise addressing the methodological limitations identified in this review. Larger, adequately powered RCTs are needed, with clearer reporting^70^ of allocation concealment, participant flow and effect size precision. Greater standardisation of outcome measures, particularly for gait, fatigue, cognition and QoL, would enhance comparability and enable future quantitative synthesis. Reporting of clinically meaningful change, alongside statistical significance, should be prioritised to support translation into practice. There is a clear need for studies that directly compare different MBTs, including active versus receptive therapies. The relative lack of research on singing-based interventions in MS represents an evidence gap, which contrasts with evidence in PD, where singing is a frequently studied therapeutic modality.^8^ Future studies should also expand beyond gait-focused outcomes to include communication, social participation and patient-prioritised outcomes. From a clinical perspective, the current evidence base supports cautious integration of MBTs, particularly rhythmic cueing and music-cued motor imagery, within MS rehabilitation programmes. These interventions appear feasible in both facility and home-based settings and may complement conventional physiotherapy and cognitive rehabilitation. However, given heterogeneity and residual risk of bias, MBTs should currently be considered an adjunct rather than a replacement for established rehabilitation approaches.

## Conclusions

This systematic review synthesised evidence from 23 studies examining the effects of MBTs on motor, cognitive, psychological, and QoL outcomes in people with MS. The narrative synthesis suggests that MBTs, particularly rhythmic auditory stimulation and music-cued motor imagery, may provide benefits for gait and mobility, with more variable evidence for cognitive, psychological, and QoL outcomes. Evidence for cognitive benefit was found most in music-assisted learning paradigms, while psychological and fatigue-related outcomes showed mixed but promising signals.

This review extends prior rehabilitation-focused syntheses by including a broader range of intervention types, outcome domains, and recent studies, while maintaining methodological consistency through exclusive use of CASP risk of bias tools. Nonetheless, heterogeneity in interventions, outcomes, and study design limits the strength of conclusions. Overall, MBTs appear to be a promising adjunct in MS rehabilitation, but higher-quality, standardised research is required to clarify optimal intervention models and establish clinical effectiveness.

## Supplementary Material

Reviewer comments

Author's manuscript

## Data Availability

All data relevant to the study are included in the article or uploaded as supplementary information. All data relevant to the study are included in the article or uploaded as supplementary information. The presented work is a systematic review. All relevant information is provided in the manuscript and appendices. This includes the data extraction form completed with the data from all included studies. No meta-analyses were conducted, so there is no analytical code.

## References

[1] Portaccio E, Magyari M, Havrdova EK, et al. Multiple sclerosis: emerging epidemiological trends and redefining the clinical course. Lancet Reg Health Eur 2024;44:100977. 10.1016/j.lanepe.2024.100977 39444703 PMC11496978

[2] GBD 2016 Multiple Sclerosis Collaborators. Global, regional, and national burden of multiple sclerosis 1990–2016: a systematic analysis for the Global Burden of Disease Study 2016. Lancet Neurol 2016;18:269–85. 10.1016/S1474-4422(18)30443-5 PMC6372756 30679040

[3] Jakimovski D, Bittner S, Zivadinov R, et al. Multiple sclerosis. Lancet 2024;403:183–202. 10.1016/S0140-6736(23)01473-3 37949093

[4] Davis E, Webster A, Whiteside B, et al. Dance for Multiple Sclerosis: A Systematic Review. Int J MS Care 2023;25:176–85. 10.7224/1537-2073.2022-088 37469335 PMC10353690

[5] Barnish MS, Nelson-Horne RV. Group-based active artistic interventions for adults with primary anxiety and depression: a systematic review. BMJ Open 2023;13:e069310. 10.1136/bmjopen-2022-069310 PMC10335485 37380205

[6] Pérez-Eizaguirre M, Vergara-Moragues E. Music Therapy Interventions in Palliative Care: A Systematic Review. J Palliat Care 2021;36:194–205. 10.1177/0825859720957803 32928042

[7] Liu Q, Li W, Yin Y, et al. The effect of music therapy on language recovery in patients with aphasia after stroke: a systematic review and meta-analysis. Neurol Sci 2022;43:863–72. 10.1007/s10072-021-05743-9 34816318

[8] Barnish MS, Reynolds SE, Nelson-Horne RV. Active group-based performing arts interventions in Parkinson’s disease: an updated systematic review and meta-analysis. BMJ Open 2025;15:e089920. 10.1136/bmjopen-2024-089920 PMC11987092 40204323

[9] Barnish MS, Barran SM. A systematic review of active group-based dance, singing, music therapy and theatrical interventions for quality of life, functional communication, speech, motor function and cognitive status in people with Parkinson’s disease. BMC Neurol 2020;20:371. 10.1186/s12883-020-01938-3 33038925 PMC7547481

[10] Barnish J, Atkinson RA, Barran SM, et al. Potential Benefit of Singing for People with Parkinson’s Disease: A Systematic Review. J Parkinsons Dis 2016;6:473–84. 10.3233/JPD-160837 27258698

[11] American Music Therapy Association. What is music therapy? Available: https://www.musictherapy.org/about/musictherapy/ [Accessed 08 May 2026].

[12] British Association for Music Therapy. What is a music therapist? Available: https://www.bamt.org/music-therapy/what-is-a-music-therapist [Accessed 08 May 2026].

[13] Canadian Association of Music Therapists. About music therapy. Available: https://www.musictherapy.ca/about-camt-music-therapy/about-music-therapy/ [Accessed 08 May 2026].

[14] Mainka S, Weymann E. Music therapy newly defined. AIJMT 2025;17:812–24. 10.56883/aijmt.2025.631

[15] Skånland MS. Music therapy and social recovery in flexible assertive community treatment. Nord J Music Ther 2023;32:290–306. 10.1080/08098131.2022.2116593

[16] Nebelung I, Kruger V. Norway: country report on professional recognition of music therapy. Approaches 2015;7:171–2. 10.56883/aijmt.2015.410

[17] Xiao H, Chen C. The study of traditional Chinese medicine five-element music therapy for depression: a bibliometric analysis. Digital Chinese Medicine 2025;8:558–70. 10.1016/j.dcmed.2025.12.010

[18] Spiro N, Sanfilippo KRM, eds. Collaborative insights: interdisciplinary perspectives on musical care throughout the life course. Oxford: Oxford University Press, 2022.

[19] Lin T-H, Liao Y-C, Tam K-W, et al. Effects of music therapy on cognition, quality of life, and neuropsychiatric symptoms of patients with dementia: A systematic review and meta-analysis of randomized controlled trials. Psychiatry Res 2023;329:115498. 10.1016/j.psychres.2023.115498 37783097

[20] Lopes J, Keppers II. Music-based therapy in rehabilitation of people with multiple sclerosis: a systematic review of clinical trials. Arq Neuropsiquiatr 2021;79:527–35. 10.1590/0004-282X-ANP-2020-0374 34320057 PMC9394567

[21] Stegemann T, Geretsegger M, Phan Quoc E, et al. Music Therapy and Other Music-Based Interventions in Pediatric Health Care: An Overview. Medicines (Basel) 2019;6:25. 10.3390/medicines6010025 30769834 PMC6473587

[22] Raglio A. A novel music-based therapeutic approach: the Therapeutic Music Listening. Front Hum Neurosci 2023;17:1204593. 10.3389/fnhum.2023.1204593 37520927 PMC10375023

[23] Wolff L, Quan Y, Perry G, et al. Music Engagement as a Source of Cognitive Reserve. Am J Alzheimers Dis Other Demen 2023;38:15333175231214833. 10.1177/15333175231214833 37993973 PMC10666690

[24] Gardener SH, Mukaetova-Ladinska EB, Perera NA. The Effect of Music Therapy on Psychological Outcomes for Neurological Conditions: A Systematic Review. Medicina (B Aires) 2025;61:1611. 10.3390/medicina61091611 PMC12471332 41011002

[25] Kong L, Zhang X, Meng L, et al. Effects of music therapy intervention on gait disorders in persons with multiple sclerosis: A systematic review of clinical trials. Mult Scler Relat Disord 2023;73:104629. 10.1016/j.msard.2023.104629 36963169

[26] Ostermann T, Schmid W. Music therapy in the treatment of multiple sclerosis: a comprehensive literature review. Expert Rev Neurother 2006;6:469–77. 10.1586/14737175.6.4.469 16623646

[27] Phillips CS, Kim J, Ganesh A, et al. Impact of Active Versus Receptive Music Interventions on Psychosocial and Neurological Outcomes in People with Multiple Sclerosis: A Systematic Review. J Integr Complement Med 2025;31:889–903. 10.1089/jicm.2024.0588 40468842

[28] Tan Y, Chen X, Wang L, et al. Music interventions for gait and mobility in multiple sclerosis: a scoping review. Mult Scler Relat Disord 2025;101:106563. 10.1016/j.msard.2025.106563 40554209

[29] Manocchio N, Moriano C, D’Amato A, et al. Beyond Cognition: Cognitive Re-Education’s Impact on Quality of Life and Psychological Well-Being in People with Multiple Sclerosis-A Narrative Review. NeuroSci 2025;6:64. 10.3390/neurosci6030064 40700129 PMC12286144

[30] Schmid W, Aldridge D. Active music therapy in the treatment of multiple sclerosis patients: a matched control study. J Music Ther 2004;41:225–40. 10.1093/jmt/41.3.225 15327343

[31] Wiens ME, Reimer MA, Guyn HL. Music therapy as a treatment method for improving respiratory muscle strength in patients with advanced multiple sclerosis: a pilot study. Rehabil Nurs 1999;24:74–80. 10.1002/j.2048-7940.1999.tb01840.x 10410058

[32] Impellizzeri F, Leonardi S, Latella D, et al. An integrative cognitive rehabilitation using neurologic music therapy in multiple sclerosis: A pilot study. Medicine (Baltimore) 2020;99:e18866. 10.1097/MD.0000000000018866 31977888 PMC7004652

[33] Munn Z, Peters MDJ, Stern C, et al. Systematic review or scoping review? Guidance for authors when choosing between a systematic or scoping review approach. BMC Med Res Methodol 2018;18:143. 10.1186/s12874-018-0611-x 30453902 PMC6245623

[34] Page MJ, McKenzie JE, Bossuyt PM, et al. The PRISMA 2020 statement: an updated guideline for reporting systematic reviews. BMJ 2021;372:n71. 10.1136/bmj.n71 33782057 PMC8005924

[35] Campbell M, McKenzie JE, Sowden A, et al. Synthesis without meta-analysis (SWiM) in systematic reviews: reporting guideline. BMJ 2020;368:l6890. 10.1136/bmj.l6890 31948937 PMC7190266

[36] Critical Appraisal Skills Programme. CASP checklists. Available: https://casp-uk.net/casp-tools-checklists/ [Accessed 12 Dec 2025].

[37] Viswanathan M, Ansari NT, Berkman ND, et al. Assessing the risk of bias of individual studies in systematic reviews of health care interventions. In: Methods guide for effectiveness and comparative effectiveness reviews. Rockville (MD): Agency for Healthcare Research and Quality, 2008. 22479713

[38] Johnson BT, Hennessy EA. Systematic reviews and meta-analyses in the health sciences: Best practice methods for research syntheses. Soc Sci Med 2019;233:237–51. 10.1016/j.socscimed.2019.05.035 31233957 PMC8594904

[39] Boon MH, Thomson H. The effect direction plot revisited: Application of the 2019 Cochrane Handbook guidance on alternative synthesis methods. Res Synth Methods 2021;12:29–33. 10.1002/jrsm.1458 32979023 PMC7821279

[40] Jaeschke R, Singer J, Guyatt GH. Measurement of health status. Ascertaining the minimal clinically important difference. Control Clin Trials 1989;10:407–15. 10.1016/0197-2456(89)90005-6 2691207

[41] Copay AG, Subach BR, Glassman SD, et al. Understanding the minimum clinically important difference (MCID) of patient-reported outcome measures. Spine J 2007;7:333–43. 10.1016/j.spinee.2007.01.008 17448732

[42] Engel L, Beaton DE, Touma Z. Minimal clinically important difference: a review of outcome measure score interpretation. Rheum Dis Clin 2018;44:177–88. 10.1016/j.rdc.2018.01.011 29622290

[43] The GRADE Working Group. The GRADE book. Available: https://book.gradepro.org/about [Accessed 10 May 2026].

[44] Schunemann H, Brozek J, Guyatt G, eds. GRADE handbook for grading quality of evidence and strength of recommendations. The GRADE Working Group, 2013.

[45] Young H-J, Mehta TS, Herman C, et al. The Effects of M2M and Adapted Yoga on Physical and Psychosocial Outcomes in People With Multiple Sclerosis. Arch Phys Med Rehabil 2019;100:391–400. 10.1016/j.apmr.2018.06.032 30092206 PMC9105798

[46] Conklyn D, Stough D, Novak E, et al. A home-based walking program using rhythmic auditory stimulation improves gait performance in patients with multiple sclerosis: a pilot study. Neurorehabil Neural Repair 2010;24:835–42. 10.1177/1545968310372139 20643882

[47] Gatti R, Tettamanti A, Lambiase S, et al. Improving hand functional use in subjects with multiple sclerosis using a musical keyboard: a randomized controlled trial. Physiother Res Int 2015;20:100–7. 10.1002/pri.1600 25045035

[48] Moore KS, Peterson DA, O’Shea G, et al. The effectiveness of music as a mnemonic device on recognition memory for people with multiple sclerosis. J Music Ther 2008;45:307–29. 10.1093/jmt/45.3.307 18959453

[49] Seebacher B, Kuisma R, Glynn A, et al. The effect of rhythmic-cued motor imagery on walking, fatigue and quality of life in people with multiple sclerosis: A randomised controlled trial. Mult Scler 2017;23:286–96. 10.1177/1352458516644058 27055804

[50] Seebacher B, Kuisma R, Glynn A, et al. Effects and mechanisms of differently cued and non-cued motor imagery in people with multiple sclerosis: A randomised controlled trial. Mult Scler 2019;25:1593–604. 10.1177/1352458518795332 30106328

[51] Seebacher B, Helmlinger B, Pinter D, et al. Actual and Imagined Music-Cued Gait Training in People with Multiple Sclerosis: A Double-Blind Randomized Parallel Multicenter Trial. Neurorehabil Neural Repair 2024;38:555–69. 10.1177/15459683241260724 38873806 PMC11308272

[52] Shahraki M, Sohrabi M, Taheri Torbati HR, et al. Effect of rhythmic auditory stimulation on gait kinematic parameters of patients with multiple sclerosis. J Med Life 2017;10:33–7. 28255373 PMC5304368

[53] Helmlinger B, Seebacher B, Ropele S, et al. Effects of rhythmic-cued gait training on gait-like task related brain activation in people with multiple sclerosis. J Neurol Sci 2025;471:123426. 10.1016/j.jns.2025.123426 39965306

[54] Maggio MG, Tripoli D, Porcari B, et al. How may patients with MS benefit from using music assisted therapy? A case-control feasability study investigating motor outcomes and beyond. Mult Scler Relat Disord 2021;48:102713. 10.1016/j.msard.2020.102713 33387863

[55] Naderi S, Sadeghi H, Amirseyfaddini M. The effects of rhythmic auditory stimulation with different tempos on spatio-temporal parameters and gait symmetry in patients with multiple sclerosis: a quasi-experimental study. J Res Rehabil Sci 2022;18:92–9. 10.48305/jrrs.2023.41771.1041

[56] Seebacher B, Kuisma R, Glynn A, et al. Rhythmic cued motor imagery and walking in people with multiple sclerosis: a randomised controlled feasibility study. Pilot Feasibility Stud 2015;1:25. 10.1186/s40814-015-0021-3 27965804 PMC5154106

[57] Goetschalckx M, Van Geel F, Meesen R, et al. Rhythmic interlimb coordination of the lower limbs in multiple sclerosis during auditory pacing to three different frequencies. Gait Posture 2021;86:334–40. 10.1016/j.gaitpost.2021.04.001 33845379

[58] Moumdjian L, Moens B, Maes P-J, et al. Continuous 12 min walking to music, metronomes and in silence: Auditory-motor coupling and its effects on perceived fatigue, motivation and gait in persons with multiple sclerosis. Mult Scler Relat Disord 2019;35:92–9. 10.1016/j.msard.2019.07.014 31357124

[59] Seebacher B, Kuisma R, Glynn A, et al. Exploring cued and non-cued motor imagery interventions in people with multiple sclerosis: a randomised feasibility trial and reliability study. Arch Physiother 2018;8:6. 10.1186/s40945-018-0045-0 29507773 PMC5833073

[60] Thaut MH, Peterson DA, Sena KM, et al. Musical structure facilitates verbal learning in multiple sclerosis. Music Percept 2008;25:325–30. 10.1525/mp.2008.25.4.325

[61] Thaut MH, Peterson DA, McIntosh GC, et al. Music mnemonics aid Verbal Memory and Induce Learning - Related Brain Plasticity in Multiple Sclerosis. Front Hum Neurosci 2014;8:395. 10.3389/fnhum.2014.00395 24982626 PMC4056382

[62] Aldridge D, Schmid W, Kaeder M, et al. Functionality or aesthetics? A pilot study of music therapy in the treatment of multiple sclerosis patients. Complement Ther Med 2005;13:25–33. 10.1016/j.ctim.2005.01.004 15907675

[63] Kuhlmann J, Ebner K, Zimmer A, et al. Music therapy with a monochord in multiple sclerosis (“MUTIMS”): A randomized, controlled, rater-blinded trial Mult Scler J Exp Transl Clin 2025;11:20552173251352712. 10.1177/20552173251352712 40655476 PMC12246520

[64] Seighalani MZ, Ghahari S, Zarbakhsh M. The effectiveness of music therapy on depression, anxiety and self-esteem of patients with multiple sclerosis. Adv Environ Biol 2014;8:253–60.

[65] Ioannidis JPA. The Mass Production of Redundant, Misleading, and Conflicted Systematic Reviews and Meta‐analyses. Milbank Quarterly 2016;94:485–514. 10.1111/1468-0009.12210 27620683 PMC5020151

[66] Barnish MS, Turner S. The value of pragmatic and observational studies in health care and public health. Pragmat Obs Res 2017;8:49–55. 10.2147/POR.S137701 28546780 PMC5438073

[67] Higgins JPT, Thomas J, Chandler J, et al. Cochrane handbook for systematic reviews of interventions version 6.5 (updated August 2024). Available: https://www.cochrane.org/handbook [Accessed 06 Jan 2026].

[68] Aali G, Shokraneh F. No limitations to language, date, publication type, and publication status in search step of systematic reviews. J Clin Epidemiol 2021;133:165–7. 10.1016/j.jclinepi.2021.02.002 33571633

[69] Absher LU, Desilets MR. English as the Scholarly Language: Diversity, Equity, and Inclusion Implications for Academic Reference and Instruction Librarians. Libr Q 2024;94:296–315. 10.1086/730464

[70] Robb SL, Story KM, Harman E, et al. Reporting Guidelines for Music-based Interventions checklist: explanation and elaboration guide. Front Psychol 2025;16:1552659. 10.3389/fpsyg.2025.1552659 40567875 PMC12188939

[71] Ng A, Bunyan S, Suh J, et al. Ballroom dance for persons with multiple sclerosis: a pilot feasibility study. Disabil Rehabil 2020;42:1115–21. 10.1080/09638288.2018.1516817 30638081

[72] Van Geel F, Van Asch P, Veldkamp R, et al. Effects of a 10-week multimodal dance and art intervention program leading to a public performance in persons with multiple sclerosis - A controlled pilot-trial. Mult Scler Relat Disord 2020;44:102256. 10.1016/j.msard.2020.102256 32570178

[73] Baştürk S, Ekici G, Kırteke F, et al. Therapeutic effects of line dancing in people with multiple sclerosis: an evaluator-blinded, randomized controlled study. Arts Health 2026;18:74–86. 10.1080/17533015.2024.2325425 38466080

[74] Baram Y, Miller A. Auditory feedback control for improvement of gait in patients with Multiple Sclerosis. J Neurol Sci 2007;254:90–4. 10.1016/j.jns.2007.01.003 17316692

[75] Moumdjian L, Maes P-J, Dalla Bella S, et al. Detrended fluctuation analysis of gait dynamics when entraining to music and metronomes at different tempi in persons with multiple sclerosis. Sci Rep 2020;10:12934. 10.1038/s41598-020-69667-8 32737347 PMC7395137

[76] Moumdjian L, Moens B, Maes P-J, et al. Walking to Music and Metronome at Various Tempi in Persons With Multiple Sclerosis: A Basis for Rehabilitation. Neurorehabil Neural Repair 2019;33:464–75. 10.1177/1545968319847962 31079541

[77] Coleman CI, Sobieraj DM, Marinucci LN. Minimally important clinical difference of the Timed 25-Foot Walk Test: results from a randomized controlled trial in patients with multiple sclerosis. Curr Med Res Opin 2012;28:49–56. 10.1185/03007995.2011.639752 22073939

[78] Button KS, Kounali D, Thomas L, et al. Minimal clinically important difference on the Beck Depression Inventory--II according to the patient’s perspective. Psychol Med 2015;45:3269–79. 10.1017/S0033291715001270 26165748 PMC4611356

[79] Lemay KR, Tulloch HE, Pipe AL, et al. Establishing the Minimal Clinically Important Difference for the Hospital Anxiety and Depression Scale in Patients With Cardiovascular Disease. J Cardiopulm Rehabil Prev 2019;39:E6–11. 10.1097/HCR.0000000000000379 30489438

[80] Puhan MA, Frey M, Büchi S, et al. The minimal important difference of the hospital anxiety and depression scale in patients with chronic obstructive pulmonary disease. Health Qual Life Outcomes 2008;6:46. 10.1186/1477-7525-6-46 18597689 PMC2459149

[81] Canadian Agency for Drugs and Technologies in Health. Clinical review report: daclizumab (Zinbryta). Ottawa (ON) CADTH; 2017. 30561964

[82] Costelloe L, O’Rourke K, Kearney H, et al. The patient knows best: significant change in the physical component of the Multiple Sclerosis Impact Scale (MSIS-29 physical). J Neurol Neurosurg Psychiatry 2007;78:841–4. 10.1136/jnnp.2006.105759 17332049 PMC2117755

