# Supplementary material for: Potential benefits of music-based therapies on rehabilitation outcomes in people with multiple sclerosis: a systematic review
Source: BMJ Open. 2026 Jul 22;16(7):e116420. doi: 10.1136/bmjopen-2026-116420 (PMC13410710; doi:10.1136/bmjopen-2026-116420)
Supplement: Supplementary data [file bmjopen-16-7-s001.pdf]

Ahadi et al. Potential benefits of music-based therapies on rehabilitation outcomes in people with multiple sclerosis: a systematic review. Supplementary files. R1.

Supplemental file 1. Completed PRISMA 2020 checklist

Section and Topic	Item #	Checklist item	Location where item is reported
**TITLE**			
Title	1	Identify the report as a systematic review.	1
**ABSTRACT**			
Abstract	2	See the PRISMA 2020 for Abstracts checklist.	2
**INTRODUCTION**			
Rationale	3	Describe the rationale for the review in the context of existing knowledge.	5-6
Objectives	4	Provide an explicit statement of the objective(s) or question(s) the review addresses.	6
**METHODS**			
Eligibility criteria	5	Specify the inclusion and exclusion criteria for the review and how studies were grouped for the syntheses.	6-8
Information sources	6	Specify all databases, registers, websites, organisations, reference lists and other sources searched or consulted to identify studies. Specify the date when each source was last searched or consulted.	6, Supp file 3
Search strategy	7	Present the full search strategies for all databases, registers and websites, including any filters and limits used.	Supp file 3
Selection process	8	Specify the methods used to decide whether a study met the inclusion criteria of the review, including how many reviewers screened each record and each report retrieved, whether they worked independently, and if applicable, details of automation tools used in the process.	6-7
Data collection process	9	Specify the methods used to collect data from reports, including how many reviewers collected data from each report, whether they worked independently, any processes for obtaining or confirming data from study investigators, and if applicable, details of automation tools used in the process.	7
Data items	10a	List and define all outcomes for which data were sought. Specify whether all results that were compatible with each outcome domain in each study were sought (e.g. for all measures, time points, analyses), and if not, the methods used to decide which results to collect.	7, Table 3
	10b	List and define all other variables for which data were sought (e.g. participant and intervention characteristics, funding sources). Describe any assumptions made about any missing or unclear information.	7, Table 3
Study risk of bias assessment	11	Specify the methods used to assess risk of bias in the included studies, including details of the tool(s) used, how many reviewers assessed each study and whether they worked independently, and if applicable, details of automation tools used in the process.	7
Effect measures	12	Specify for each outcome the effect measure(s) (e.g. risk ratio, mean difference) used in the synthesis or presentation of results.	N/A – no meta-analysis

Section and Topic	Item #	Checklist item	Location where item is reported
Synthesis methods	13a	Describe the processes used to decide which studies were eligible for each synthesis (e.g. tabulating the study intervention characteristics and comparing against the planned groups for each synthesis (item #5)).	6-8
	13b	Describe any methods required to prepare the data for presentation or synthesis, such as handling of missing summary statistics, or data conversions.	N/A – no meta-analysis
	13c	Describe any methods used to tabulate or visually display results of individual studies and syntheses.	N/A – no meta-analysis
	13d	Describe any methods used to synthesize results and provide a rationale for the choice(s). If meta-analysis was performed, describe the model(s), method(s) to identify the presence and extent of statistical heterogeneity, and software package(s) used.	7-8
	13e	Describe any methods used to explore possible causes of heterogeneity among study results (e.g. subgroup analysis, meta-regression).	N/A – no meta-analysis
	13f	Describe any sensitivity analyses conducted to assess robustness of the synthesized results.	N/A – no meta-analysis
Reporting bias assessment	14	Describe any methods used to assess risk of bias due to missing results in a synthesis (arising from reporting biases).	N/A – no meta-analysis
Certainty assessment	15	Describe any methods used to assess certainty (or confidence) in the body of evidence for an outcome.	9
**RESULTS**			
Study selection	16a	Describe the results of the search and selection process, from the number of records identified in the search to the number of studies included in the review, ideally using a flow diagram.	Fig 1
	16b	Cite studies that might appear to meet the inclusion criteria, but which were excluded, and explain why they were excluded.	Supp file 5
Study characteristics	17	Cite each included study and present its characteristics.	Supp file 6
Risk of bias in studies	18	Present assessments of risk of bias for each included study.	Supp file 11

**Section and Topic**	**Item #**	**Checklist item**	**Location where item is reported**
Results of individual studies	19	For all outcomes, present, for each study: (a) summary statistics for each group (where appropriate) and (b) an effect estimate and its precision (e.g. confidence/credible interval), ideally using structured tables or plots.	No meta-analysis, relevant info in Supp file 9
Results of syntheses	20a	For each synthesis, briefly summarise the characteristics and risk of bias among contributing studies.	9-10, Suppl file 10
	20b	Present results of all statistical syntheses conducted. If meta-analysis was done, present for each the summary estimate and its precision (e.g. confidence/credible interval) and measures of statistical heterogeneity. If comparing groups, describe the direction of the effect.	N/A – no meta-analysis
	20c	Present results of all investigations of possible causes of heterogeneity among study results.	N/A – no meta-analysis
	20d	Present results of all sensitivity analyses conducted to assess the robustness of the synthesized results.	N/A – no meta-analysis
Reporting biases	21	Present assessments of risk of bias due to missing results (arising from reporting biases) for each synthesis assessed.	N/A – no meta-analysis
Certainty of evidence	22	Present assessments of certainty (or confidence) in the body of evidence for each outcome assessed.	13, Table 5
**DISCUSSION**			
Discussion	23a	Provide a general interpretation of the results in the context of other evidence.	14-15
	23b	Discuss any limitations of the evidence included in the review.	14-16
	23c	Discuss any limitations of the review processes used.	15-16
	23d	Discuss implications of the results for practice, policy, and future research.	16
**OTHER INFORMATION**			
Registration and protocol	24a	Provide registration information for the review, including register name and registration number, or state that the review was not registered.	6
	24b	Indicate where the review protocol can be accessed, or state that a protocol was not prepared.	6
	24c	Describe and explain any amendments to information provided at registration or in the protocol.	6

Section and Topic	Item #	Checklist item	Location where item is reported
Support	25	Describe sources of financial or non-financial support for the review, and the role of the funders or sponsors in the review.	17
Competing interests	26	Declare any competing interests of review authors.	17
Availability of data, code and other materials	27	Report which of the following are publicly available and where they can be found: template data collection forms; data extracted from included studies; data used for all analyses; analytic code; any other materials used in the review.	17

From: Page MJ, McKenzie JE, Bossuyt PM, Boutron I, Hoffmann TC, Mulrow CD, et al. The PRISMA 2020 statement: an updated guideline for reporting systematic reviews. BMJ 2021;372:n71. doi: 10.1136/bmj.n71. This work is licensed under CC BY 4.0. To view a copy of this license, visit https://creativecommons.org/licenses/by/4.0/

Synthesis Without Meta-analysis (SWiM) reporting items

The citation for the Synthesis Without Meta-analysis explanation and elaboration article is: Campbell M, McKenzie JE, Sowden A, Katikireddi SV, Brennan SE, Ellis S, Hartmann-Boyce J, Ryan R, Shepperd S, Thomas J, Welch V, Thomson H. Synthesis without meta-analysis (SWiM) in systematic reviews: reporting guideline BMJ 2020;368:l6890 http://dx.doi.org/10.1136/bmj.l6890

**SWiM is intended to complement and be used as an extension to PRISMA**			
**SWiM reporting item**	**Item description**	**Page in manuscript where item is reported**	**Other***
Methods			
**1** Grouping studies for synthesis	1a) Provide a description of, and rationale for, the groups used in the synthesis (e.g., groupings of populations, interventions, outcomes, study design)	8	
	1b) Detail and provide rationale for any changes made subsequent to the protocol in the groups used in the synthesis	N/A (no changes)	
**2** Describe the standardised metric and transformation methods used	Describe the standardised metric for each outcome. Explain why the metric(s) was chosen, and describe any methods used to transform the intervention effects, as reported in the study, to the standardised metric, citing any methodological guidance consulted	8	

**3** Describe the synthesis methods	Describe and justify the methods used to synthesise the effects for each outcome when it was not possible to undertake a meta-analysis of effect estimates	8	
**4** Criteria used to prioritise results for summary and synthesis	Where applicable, provide the criteria used, with supporting justification, to select the particular studies, or a particular study, for the main synthesis or to draw conclusions from the synthesis (e.g., based on study design, risk of bias assessments, directness in relation to the review question)	8	
**SWiM reporting item**	**Item description**	**Page in manuscript where item is reported**	**Other***
**5** Investigation of heterogeneity in reported effects	State the method(s) used to examine heterogeneity in reported effects when it was not possible to undertake a meta-analysis of effect estimates and its extensions to investigate heterogeneity	8	
**6** Certainty of evidence	Describe the methods used to assess certainty of the synthesis findings	8	
**7** Data presentation methods	Describe the graphical and tabular methods used to present the effects (e.g., tables, forest plots, harvest plots).<br>Specify key study characteristics (e.g., study design, risk of bias) used to order the studies, in the text and any tables or graphs, clearly referencing the studies included	8	
Results			
**8** Reporting results	For each comparison and outcome, provide a description of the synthesised findings, and the certainty of the findings. Describe the result in language that is consistent with the question the synthesis addresses, and indicate which studies contribute to the synthesis	10-13	
Discussion			
**9** Limitations of the synthesis	Report the limitations of the synthesis methods used and/or the groupings used in the synthesis, and how these affect the conclusions that can be drawn in relation to the original review question	16	

PRISMA=Preferred Reporting Items for Systematic Reviews and Meta-Analyses.
*If the information is not provided in the systematic review, give details of where this information is available (e.g., protocol, other published papers (provide citation details), or website (provide the URL)).

Supplemental file 2. Completed PRISMA for Abstracts 2020 checklist

Section and Topic	Item #	Checklist item	Reported (Yes/No)
**TITLE**			
Title	1	Identify the report as a systematic review.	Yes
**BACKGROUND**			
Objectives	2	Provide an explicit statement of the main objective(s) or question(s) the review addresses.	Yes
**METHODS**			
Eligibility criteria	3	Specify the inclusion and exclusion criteria for the review.	Yes
Information sources	4	Specify the information sources (e.g. databases, registers) used to identify studies and the date when each was last searched.	Yes
Risk of bias	5	Specify the methods used to assess risk of bias in the included studies.	Yes
Synthesis of results	6	Specify the methods used to present and synthesise results.	Yes
**RESULTS**			
Included studies	7	Give the total number of included studies and participants and summarise relevant characteristics of studies.	Yes
Synthesis of results	8	Present results for main outcomes, preferably indicating the number of included studies and participants for each. If meta-analysis was done, report the summary estimate and confidence/credible interval. If comparing groups, indicate the direction of the effect (i.e. which group is favoured).	Yes
**DISCUSSION**			
Limitations of evidence	9	Provide a brief summary of the limitations of the evidence included in the review (e.g. study risk of bias, inconsistency and imprecision).	Yes
Interpretation	10	Provide a general interpretation of the results and important implications.	Yes
**OTHER**			
Funding	11	Specify the primary source of funding for the review.	Yes
Registration	12	Provide the register name and registration number.	Yes

Supplemental file 3. Detailed search strategies

Medline – via OVID

Search date: Wednesday 16th July 2025

1. exp Multiple Sclerosis/

2. multiple sclerosis.mp.

3. MS.mp.

4. 1 or 2 or 3

5. exp Music Therapy/

6. music therap.mp.*

7. (music-based adj3 therap*).mp.

8. Music-based therapy.mp.

9. music intervention

10. music interventions.mp.

11. musical intervention.mp.

12. musical interventions.mp.

13. rhythmic auditory stimulation.mp.

14. sound therap.mp.*

15. singing therapy.mp.*

16. auditory stimulation.mp.*

17. 5 or 6 or 7 or 8 or 9 or 10 or 11 or 12 or 13 or 14 or 15

18. 4 and 17

19. limit 14 to English language

Results: 198

Search History (19)

View Saved

#	Searches	Results	Runtime	Type	Actions	Annotations
1	exp Multiple Sclerosis/	75635	0.62	Advanced	Display Results More	
2	Multiple Sclerosis.mp.	109818	0.98	Advanced	Display Results More	
3	MS.mp.	478942	0.48	Advanced	Display Results More	
4	1 or 2 or 3	531625	0.09	Advanced	Display Results More	
5	exp Music Therapy/	4964	0.09	Advanced	Display Results More	
6	music therap*.mp.	6740	2.61	Advanced	Display Results More	
7	(music-based adj3 therap*).mp. [mp=title, book title, abstract, original title, name of substance word, subject heading word, floating sub-heading word, keyword heading word, organism supplementary concept word, protocol supplementary concept word, rare disease supplementary concept word, unique identifier, synonyms, population supplementary concept word, anatomy supplementary concept word]	103	2.42	Advanced	Display Results More	
8	music-based therapy.mp.	27	0.64	Advanced	Display Results More	
9	music intervention.mp.	700	0.34	Advanced	Display Results More	
10	music interventions.mp.	456	0.32	Advanced	Display Results More	
11	musical intervention.mp.	86	0.28	Advanced	Display Results More	
12	musical interventions.mp.	68	0.27	Advanced	Display Results More	
13	rhythmic auditory stimulation.mp.	215	0.52	Advanced	Display Results More	
14	sound therap*.mp.	458	2.16	Advanced	Display Results More	
15	singing therapy*.mp.	22	1.16	Advanced	Display Results More	
16	auditory stimulation*.mp.	2366	0.63	Advanced	Display Results More	
17	5 or 6 or 7 or 8 or 9 or 10 or 11 or 12 or 13 or 14 or 15 or 16	9799	0.02	Advanced	Display Results More	
18	4 and 17	211	0.05	Advanced	Display Results More	
19	limit 18 to english language	198	0.02	Advanced	Display Results More	

Save Remove Combine with: AND OR Contract

Embase via Ovid

Search date: Wednesday 16th July 2025

1. *exp Multiple Sclerosis/*
2. *multiple sclerosis.mp.*
3. *MS.mp.*
4. *1 or 2 or 3*
5. *exp Music Therapy/*
6. *music therap*.mp.*
7. music-based therap*.mp.

8. music intervention,mp.
9. music interventions.mp.
10. musical intervention.mp.
11. musical interventions.mp.
12. *rhythmic auditory stimulation.mp.*
13. *sound therap*.mp.*
14. *singing therap*.mp.*
15. *auditory stimulation*.mp.*
16. *5 or 6 or 7 or 8 or 9 or 10 or 11 or 12 or 13 0r 14 or 15*
17. *4 and 16*
18. *limit 17 to English language*

results: 4029

# ▲	Searches	Results	Runtime	Type	Actions	Annotations
1	exp multiple sclerosis/	176930	0.07	Advanced	Display Results More	
2	multiple sclerosis.mp.	193474	0.77	Advanced	Display Results More	
3	MS.mp.	664062	0.18	Advanced	Display Results More	
4	1 or 2 or 3	754065	0.15	Advanced	Display Results More	
5	exp music therapy/	11246	0.08	Advanced	Display Results More	
6	music therap*.mp.	11935	4.36	Advanced	Display Results More	
7	music-based therap*.mp.	61	4.43	Advanced	Display Results More	
8	music intervention.mp.	1086	0.23	Advanced	Display Results More	
9	music interventions.mp.	595	0.15	Advanced	Display Results More	
10	musical intervention.mp.	143	0.22	Advanced	Display Results More	
11	musical interventions.mp.	98	0.16	Advanced	Display Results More	
12	rhythmic auditory stimulation.mp.	338	0.65	Advanced	Display Results More	
13	sound therap*.mp.	625	4.48	Advanced	Display Results More	
14	singing therap*.mp.	36	4.21	Advanced	Display Results More	
15	auditory stimulation*.mp.	43773	0.43	Advanced	Display Results More	
16	5 or 6 or 7 or 8 or 9 or 10 or 11 or 12 or 13 or 14 or 15	56404	0.02	Advanced	Display Results More	
17	4 and 16	4168	0.07	Advanced	Display Results More	
18	limit 17 to english language	4029	0.02	Advanced	Display Results More	

Save Remove Combine with: AND OR Contract

APA PsycInfo via Ovid

Search date: Wednesday 16th July 2025

1. *exp Multiple Sclerosis/*
2. *multiple sclerosis.mp.*
3. *MS.mp.*
4. *1 or 2 or 3*
5. *exp Music Therapy/*
6. *music therap*.mp.*
7. music-based therap*.mp.
8. music intervention.mp.
9. music interventions.mp.
10. musical intervention.mp.
11. musical interventions.mp.
12. *rhythmic auditory stimulation.mp.*
13. *sound therap*.mp.*
14. *singing therap*.mp.*
15. *auditory stimulation*.mp.*
16. *5 or 6 or 7 or 8 or 9 or 10 or 11 or 12 or 13 0r 14 or 15*
17. *4 and 16*
18. *limit 17 to English language*

results: 1155

#	Searches	Results	Runtime	Type	Actions	Annotations
1	exp Multiple Sclerosis/	15247	0.14	Advanced	Display Results More	
2	Multiple Sclerosis.mp.	19196	1.06	Advanced	Display Results More	
3	MS.mp.	37720	0.20	Advanced	Display Results More	
4	1 or 2 or 3	44700	0.01	Advanced	Display Results More	
5	exp Music Therapy/	6337	0.13	Advanced	Display Results More	
6	Music therap*.mp.	7695	0.57	Advanced	Display Results More	
7	music-based therap*.mp.	27	1.03	Advanced	Display Results More	
8	music intervention.mp.	353	0.29	Advanced	Display Results More	
9	music interventions.mp.	304	0.24	Advanced	Display Results More	
10	musical intervention.mp.	59	0.29	Advanced	Display Results More	
11	musical interventions.mp.	76	0.23	Advanced	Display Results More	
12	rhythmic auditory stimulation.mp.	83	0.58	Advanced	Display Results More	
13	sound therap*.mp.	167	0.68	Advanced	Display Results More	
14	singing therap*.mp.	13	0.73	Advanced	Display Results More	
15	auditory stimulation*.mp.	24980	0.60	Advanced	Display Results More	
16	5 or 6 or 7 or 8 or 9 or 10 or 11 or 12 or 13 or 14 or 15	32845	0.01	Advanced	Display Results More	
17	4 and 16	1175	0.01	Advanced	Display Results More	
18	limit 17 to english language	1155	0.13	Advanced	Display Results More	

Save Remove Combine with: AND OR Contract

AMED via EBSCOhost

Search date: Wednesday 16th July 2025

"multiple sclerosis" OR "MS"

AND

"music therap*" OR "music-based therap*" OR "musical intervention" OR "musical interventions" OR “music intervention” OR “music interventions” OR "rhythmic auditory stimulation*" OR "sound therap*" OR "singing therap*" OR "auditory stimulation*"

Limit = English language

Results: 17

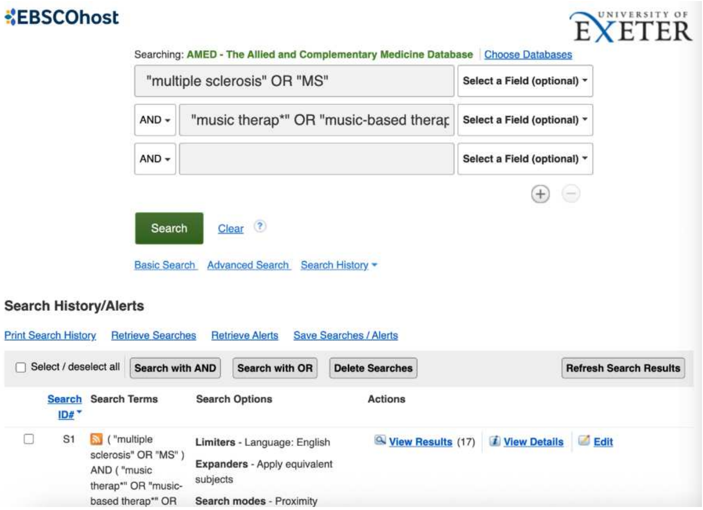

<u>CINAHL Ultimate via EBSCOhost</u>

Search date: Wednesday 16th July 2025

"multiple sclerosis" OR "MS"

AND

"music therap*" OR "music-based therap*" OR "musical intervention" OR "musical interventions" OR “music intervention” OR “music interventions” OR "rhythmic auditory stimulation*" OR "sound therap*" OR "singing therap*" OR "auditory stimulation*"

Limit = English language

Results: 107

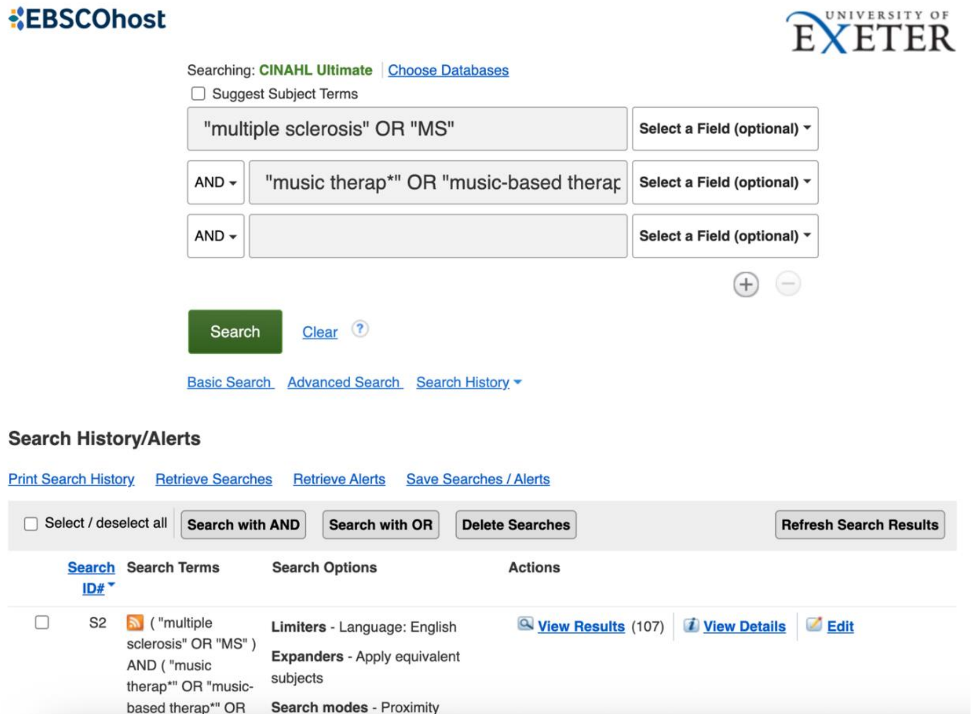

COCHRANE-Central

Search date: Wednesday 16th July 2025

"multiple sclerosis" OR "MS"

AND

(music therapy) OR (music-based therapy) OR (musical intervention) OR (musical interventions) OR (music intervention) OR (music interventions) OR (rhythmic auditory stimulation) OR (sound therapy) OR (singing therapy) OR (auditory stimulation)

Results: 400 trials (Trials = 392 are English and 8 non-English)

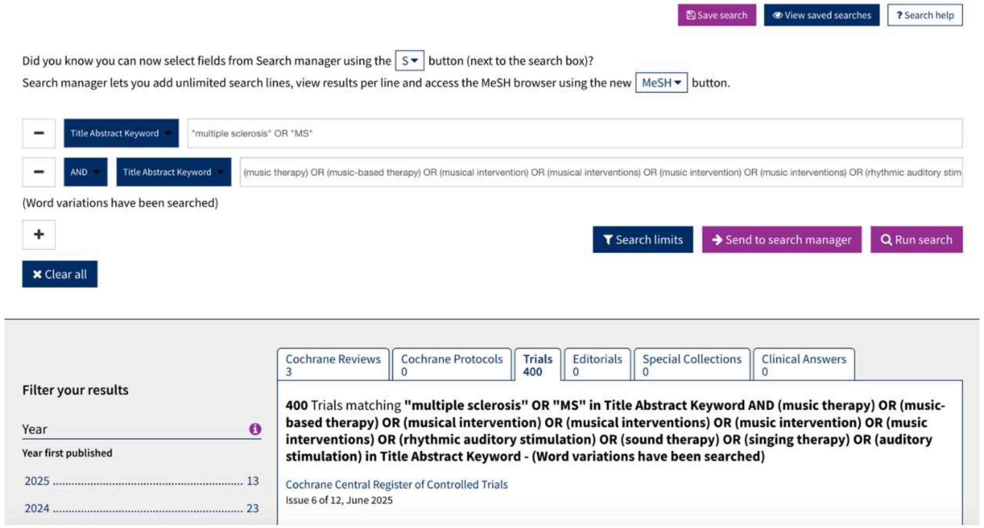

Save search
View saved searches
? Search help
Did you know you can now select fields from Search manager using the S button (next to the search box)?
Search manager lets you add unlimited search lines, view results per line and access the MeSH browser using the new MeSH button.
Title Abstract Keyword
"multiple sclerosis" OR "MS"
AND
Title Abstract Keyword
(music therapy) OR (music-based therapy) OR (musical intervention) OR (musical interventions) OR (music intervention) OR (music interventions) OR (rhythmic auditory stim
(Word variations have been searched)
Search limits
Send to search manager
Run search
Clear all
Filter your results
Year
Year first published
2025 13
2024 23
Cochrane Reviews 3
Cochrane Protocols 0
Trials 400
Editorials 0
Special Collections 0
Clinical Answers 0
400 Trials matching "multiple sclerosis" OR "MS" in Title Abstract Keyword AND (music therapy) OR (music-based therapy) OR (musical intervention) OR (musical interventions) OR (music intervention) OR (music interventions) OR (rhythmic auditory stimulation) OR (sound therapy) OR (singing therapy) OR (auditory stimulation) in Title Abstract Keyword - (Word variations have been searched)
Cochrane Central Register of Controlled Trials
Issue 6 of 12, June 2025

Supplemental file 4. Full list of included publications

1. Aldridge D, Schmid W, Kaeder M, et al. Functionality or aesthetics? A pilot study of music therapy in the treatment of multiple sclerosis patients. *Complement Ther Med* 2005; 13(1): 25–33.
2. Conklyn D, Stough D, Novak E, et al. A home-based walking program using rhythmic auditory stimulation improves gait performance in patients with multiple sclerosis: a pilot study. Neurorehabil Neural Repair 2010; 24(9): 835-42.
3. Gatti R, Tettamanti A, Lambiase S, et al. Improving hand functional use in subjects with multiple sclerosis using a musical keyboard: a randomized controlled trial. Physiother Res Int 2015; 20: 100-7.
4. Goetschalckx M, Van Geel F, Meesen R, et al. Rhythmic interlimb coordination of the lower limbs in multiple sclerosis during auditory pacing to three different frequencies. Gait Posture 2021; 334-40.
5. Helminger B, Seebacher B, Ropele S, et al. Effects of rhythmic-cued gait training on gait-like task related brain activation in people with multiple sclerosis. J Neurol Sci 2025; 471: 123426.
6. Impellizzeri F, Leonardi S, Latella D, et al. An integrative cognitive rehabilitation using neurologic music therapy in multiple sclerosis: a pilot study. Medicine 2020: 99(4): e18866.
7. Kuhlmann J, Ebner K, Zimmer A, et al. Music therapy with a monochord in multiple sclerosis (“MUTIMS”): A randomized, controlled, rater-blinded trial. Mult Scler J Exp Transl Clin 2025; 11(3):20552173251352712.
8. Maggio MG, Tripoli D, Porcari B, et al. How may patients with MS benefit from using music assisted therapy? A case-control feasability study investigating motor outcomes and beyond. Mult Scler Relat Disord 2021; 48: 102713.
9. Moore KS, Peterson DA, O’Shea G, et al. The effectiveness of music as a mnemonic device on recognition memory for people with multiple sclerosis. J Music Ther 2008; 45(3): 307-29.
10. Moumdjian L, Moens B, Maes PJ, et al. Continuous 12 min walking to music, metronomes and in silence: auditory-motor coupling and its effects on perceived fatigue, motivation and gait in persons with multiple sclerosis. Mult Scler Relat Disord 2019; 35: 92-9.
11. Naderi S, Sadeghi H, Amirseyfaddini M. The effects of rhythmic auditory stimulation with different tempos on spatio-temporal parameters and gait symmetry in patients with multiple sclerosis: a quasi-experimental study. J Res Rehabil Sci 2022; 18: doi: 10.48305/jrrs.2023.41771.1041.
12. Schmid W, Aldridge D. Active music therapy in the treatment of multiple sclerosis patients: a matched control study. J Music Ther 2004; 41(3): 225-40.
13. Seebacher B, Helmlinger B, Pinter D, et al. Actual and imagined music-cued gait training in people with multiple sclerosis: a double-blind randomized parallel multicenter trial. Neurorehabil Neural Repair 2024; 38(8): 555-69.
14. Seebacher B, Kuisma R, Glynn A, et al. Effects and mechanisms of differently cued and non-cued motor imagery in people with multiple sclerosis: a randomised controlled trial. Mult Scler J 2019; 25(12): 1593-1604.
15. Seebacher B, Kuisma R, Glynn A, et al. Exploring cued and non-cued motor imagery interventions in people with multiple sclerosis: a randomised feasibility trial and reliability study. Arch Physiother 2018; 8:6.
16. Seebacher B, Kuisma R, Glynn A, et al. The effect of rhythmic-cued motor imagery on walking, fatigue and quality of life in people with multiple sclerosis: a randomised controlled trial. Mult Scler J 2017; 23(2): 286-96.
17. Seebacher B, Kuisma R, Glynn A, et al. Rhythmic cued motor imagery and walking in people with multiple sclerosis: a randomised controlled feasibility study.
    Pilot Feasibility Stud 2015; 1: 25.
18. Seighalani MZ, Ghahari S, Zarbakhsh M. The effectiveness of music therapy on depression, anxiety and self-esteem of patients with multiple sclerosis (MS). Adv Environ Biol 2014; 8(9): 253-60.

19. Shahraki M, Sohrabi M, Taheri Torbati HR, et al. Effect of rhythmic auditory stimulation on gait kinematic parameters of patients with multiple sclerosis. J Med Life 2017; 10(1): 33-7.
20. Thaut MH, Peterson DA, Sena KM, et al. Musical structure facilitates verbal learning in multiple sclerosis. *Music Percept* 2008; 25(4): 325–30.
21. Thaut MH, Peterson DA, McIntosh GC, et al. Music mnemonics aid verbal memory and induce learning – related brain plasticity in multiple sclerosis. Front Hum Neurosci 2014; 8: 395.
22. Wiens ME, Reimer MA, Guyn HL. Music therapy as a treatment method for improving respiratory muscle strength in patients with advanced multiple sclerosis: a pilot study. Rehabil Nurs 1999; 24(2): 74-80.
23. Young HJ, Mehta TS, Herman C, et al. The effects of M2M and adapted yoga on physical and psychosocial outcomes in people with multiple sclerosis. Arch Phys Med Rehabil 2019;100(3):391-400.

List of studies for each outcome domain (studies can contribute to multiple outcome domains)

<u>Motor</u>

1. Conklyn et al. 2010
2. Gatti et al. 2015
3. Goetschalckx. et al. 2021
4. Helminger. et al. 2025
5. Maggio et al. 2021
6. Moumdjian et al. 2019
7. Naderi et al 2022
8. Schmid et al, 2004
9. Seebacher et al. 2015
10. Seebacher et al. 2017
11. Seebacher et al. 2018
12. Seebacher et al. 2019
13. Seebacher et al. 2024
14. Shahraki et al, 2017
15. Wiens et al. 1999
16. Young et al. 2019

<u>Cognitive</u>

1. Aldridge et al. 2005
2. Impellizzeri et al. 2020
3. Moore et al. 2008
4. Moumdjian et al. 2019
5. Schmid et al, 2004
6. Seebacher et al. 2024
7. Thaut et al 2008

8. Thaut et al 2014

Psychological

1. Aldridge et al. 2005
2. Impellizzeri et al. 2020
3. Kuhlmann et al. 2025
4. Maggio et al. 2021
5. Schmid et al. 2004
6. Seebacher et al. 2024
7. Seighalani et al. 2014

Quality of life

1. Aldridge et al. 2005
2. Concklyn et al 2010
3. Impellizzeri et al. 2020
4. Kuhlmann et al. 2025
5. Maggio et al. 2021
6. Schmid et al. 2004
7. Seebacher et al. 2015
8. Seebacher et al. 2017
9. Seebacher et al. 2018
10. Seebacher et al. 2019
11. Seebacher et al. 2024
12. Young et al. 2019

Supplemental file 5. Full list of studies excluded at full text review with reasons

Duplicate

1. Lambiase S, Nava C, Tettamanti A, et al. Improving hand functional use in subjects with multiple sclerosis using a musical keyboard: a randomized controlled trial. Physiother Res Int 2015;20: 100-7. [This is Gatti et al, 2015, included study].
2. Moore KS, Peterson DA, O'Shea G, et al. The effectiveness of music as a mnemonic device on recognition memory for people with multiple sclerosis. *J Music Ther* 2008; 45(3): 307–29.

Intervention

3. Baram Y, Miller A. Auditory feedback control for improvement of gait in patients with multiple sclerosis. J Neurol Sci 2007; 254: 90-4.
4. Basturk S, Ekici G, Kirteke F, et al. Therapeutic effects of line dancing in people with multiple sclerosis: an evaluator-blinded, randomized controlled study. Art Health 2024; doi: 10.1080/17533015.2024.2325425.
5. Mandelbaum R, Triche EW, Fasoli SE, et al. A pilot study: examining the effects and tolerability of structured dance intervention for individuals with multiple sclerosis. *Disabil Rehabil* 2016; 38(3): 218–22
6. Trinchillo A, Caliendo D, Nicolella V, et al. Tango classes in people with Multiple Sclerosis (PwMS): Impact on motor and non-motor functions. Clin Neurol Neurosurg 2024; 243: 108380.
7. Van Geel F, Van Asch P, Veldkamp R, et al. Effects of a 10-week multimodal dance and art intervention program leading to a public performance in persons with multiple sclerosis: a controlled pilot-trial. Mult Scler Relat Disord 2020; 44: 102256.

Outcome

8. Fasching B, Mildner S, Fink F, et al. MuSic Moves-co-creating a music-supported exercise programme with and for people with multiple sclerosis: a bicentre participatory mixed methods study. BMJ Open 2024; 14(11): e091168.
9. Moumdjian L, Maes PJ, Dalla Bella S, et al. Detrended fluctuation analysis of gait dynamics when entraining to music and metronomes at different tempi in persons with multiple sclerosis. Sci Rep 2020; 10: 12934.
10. Moumdjian L, Moens B, Maes PJ, et al. Walking to music and metronome at various tempi in persons with multiple sclerosis: a basis for rehabilitation. Neurorehabil Neural Repair 2019; 33(6): 464-75.
11. Seebacher B, Helmlinger B, Hotz I, et al. Actual and imagined music-cued gait training for people with multiple sclerosis: a multicentre qualitative study. BMJ Open 2024; 14: e086555.
12. Seighalani MZ, Cheraghali Gol H, Rostami AM. The effectiveness of music therapy on depression, anxiety and self-esteem of patients with multiple sclerosis. *Adv Environ Biol* 2014; 8(10): 759–64.
13. Vanbilsen N, Moumdjian L, Kinnaert F, et al. Step to the beat: Auditory-motor coupling during walking to higher and lower tempi with music and metronomes in progressive multiple sclerosis: an observational study. Mult Scler Relat Disord 2024; 92: 106152.

Population

14. Srinithiwat P, Foocharoen C, Mahakkanukrauh A, et al. Patient perspectives and satisfaction following a short-term singing intervention in systemic sclerosis patients: a cross-sectional study. *J Med Assoc Thai* 2025; 108: S59–65.

15. Young HJ, Lai B, Wilroy J, et al. Effects of a teleexercise movement-to-music intervention on health outcomes in individuals with mobility disabilities: a randomized controlled trial. Arch Phy Med Rehabil 2025; https://doi.org/10.1016/j.apmr.2025.05.017.

Publication type

16. https://clinicaltrials.gov/study/NCT05792176. Ukulele playing to improve cognition in people with multiple sclerosis: a feasibility study [No results posted].
17. https://clinicaltrials.gov/study/NCT04019912. The novel use of treadmill plus music in MS patients gate rehabilitation (SMUSIC) [No results posted – spelling mistake ‘gate’ for ‘gait’ was made on Clinical Trials.gov].
18. https://clinicaltrials.gov/study/NCT04314076. Rhythmic auditory stimulation & gait training [No results posted].
19. https://clinicaltrials.gov/study/NCT01086371. Rhythmic auditory stimulation and walking performance in multiple sclerosis (MS) patients [No results posted].
20. https://clinicaltrials.gov/study/NCT06800144. Effect of music therapy on pain in people with multiple sclerosis [No results posted].
21. https://trialsearch.who.int/Trial2.aspx?TrialID=IRCT20230219057455N1. The effectiveness of slow and fast music on anxiety and quality of life of people with multiple sclerosis [No results posted].
22. https://trialsearch.who.int/Trial2.aspx?TrialID=DRKS00020469. Effectiveness of playing the instruments „Big Bom“and “Temple Blocks” as a music therapy approach to improve gait function among patients with multiple sclerosis [No results posted].
23. Bethoux F, Awad L, Gallagher L, et al. Safety and acceptability of closed-loop rhythmic cueing for gait training in persons with multiple sclerosis: a pilot single-blind randomized controlled trial. Mult Scler J 2023: 29(3): 645 [Abstract].
24. Pedulla L, Addeo S, Rinaldi S, Aerobic training in multiple sclerosis: a pilot study on the effects of music during group treatment. Mult Scler J 2020; *26* (Suppl 2): 54-55 [Abstract].

Supplemental file 6. Study characteristics for included studies.

Author, year	Country	Design	Participants	Inclusion criteria	Exclusion criteria	Outcomes
Aldridge et al. 2005	Germany	Matched controlled pilot study	20 people with MS (10 per arm).<br>30% male<br>Age range: 29-47	Diagnosis of multiple sclerosis and within the EDSS range used for matching	Pregnancy and mental disorders requiring medication	Primary:<br>Psychological outcomes such as Depression (Beck, HADS), Anxiety (HADS), Self-acceptance (SESA)<br>Secondary:<br>Quality of life (HAQUAMS)<br>Functional disability (EDSS)<br>Cognitive (MSFC) and motor function (MSFC)<br>Fatigue (IFSS)
Conklyn et al, 2010	USA	RCT	10 people with MS (5 per arm), 30% male, Mean age: intervention 47, control 50	Age 18 or over; medical diagnosis of MS; able to walk 30.48m without physical assistance (assistive devices were allowed); able to walk 7.62m in 8 to 60s [distances were published in imperial feet]	Treatment for MS exacerbation in past 30 days; severe comorbidity precluding participation; severe cognitive deficits precluding informed consent or preventing following study instructions	Primary: Gait parameters<br><br>Secondary:<br>Spasticity (MAS Modified Ashworth Scale); muscle strength (Manual Muscle Testing); Pain (out of 10);<br>Ambulation disability (Ambulation Index);<br>MS-related disability

						(Patient-determined Disease Steps); Treatment efficacy (Subject Global Impression, Clinician Global Impression)
Gatti et al, 2015	Italy	RCT	19 people with MS (9 intervention, 10 control), 37% male, mean age intervention 43, control 48	Adults; primary or secondary progressive MS; no relapse or medication changes in past 3 months; hand strength below normal; Nine-Hole Peg Test >18s; no experience playing a musical instrument; no cognitive or psychiatric disorders; no hearing or visual deficits	No additional criteria	Primary: Perceived hand functional use (ABILHAND)<br><br>Secondary: Hand dexterity (Nine-Hole Peg Test); Hand strength (Jamar and Pinch)
Goetschalckx et al, 2021	Belgium	Observational cross-sectional comparative study	38 people with MS (16% male, mean age 49).13 healthy controls (38% male, mean age 52). Note that the 'controls' were not a comparator group in terms of intervention – they received the same MBTs, the only difference was they did not have MS	MS diagnosis (McDonald criteria); age 18-70; able to walk independently	Other medical conditions influencing walking	Primary: Bilateral gait coordination (Phase Coordination Index)<br><br>Secondary: movement amplitude; movement frequency

Helminger et al, 2025	Austria	Secondary analysis of a randomised controlled trial (longitudinal interventional study)	48 people with MS (52% male, mean age 44). 17 health controls (53% male, mean age 41)	Age at least 18; definite MS (Thompson criteria); mild-to-moderate disability (Expanded Disability Status Scale 2-5);	Relapse or change in disease-modifying treatment or physiotherapy in past 3 months; concomitant diseases; MRI contraindications	Primary: Walking function (2-minute walk test; timed 25-foot walk)<br><br>Secondary: fMRI brain activation changes during movement tasks; correlation of brain activation changes with walking performance
Impellizzeri et al, 2020	Italy	RCT	30 people with MS (15 per arm), 63% male, mean age intervention 52, control 51	MS diagnosis (Lublin criteria); Expanded Disability Status Scale 3-7; enjoy music; no visual or hearing loss; no severe medical and psychiatric illness	No additional criteria	Cognition (Brief Repeatability Battery of Neurophysiology); quality of life (MSQoL-54); depression (Beck); emotions (Emotional Awareness Questionnaire)
Kuhlmann et al, 2025	Switzerland	RCT	57 people with MS (30 intervention, 27 control), 18% male, mean age 50	Aged at least 18; MS diagnosis (McDonald criteria); Expanded Disability Status Scale no more than 6.5	Severe psychiatric disorders; other life-threatening or severely disabling physical disorders; >2 MS relapses in past year or relapse in past 3 months; change in disease-modifying treatment or symptomatic MS medication in past 3	Primary: Anxiety (HADS)<br><br>Secondary: Depression (HADS); fatigue (self-report); pain (quantitative sensory testing); body perception (self-report)

					months; pregnancy; unable to lie supine for 15 min; severely compromised hearing (uncorrected); numbness on the back of both hands	
Maggio et al, 2021	Italy	Quasi-experimental controlled feasibility study	20 people with MS (10 per arm), 35% male	Secondary progressive MS (Lublin criteria); Expanded Disability Status Scale 3-6; enjoy music; no hearing or visual loss; no change in disease-modifying drugs in past 6 months	Age <65; other severe medical conditions or psychiatric illnesses; history of neuroplasms or other neurological conditions; moderate-severe cognitive decline (MoCA <21); neurologic music therapy in past 3 months	Motor evaluation (Berg Balance; Timed Up and Go; 10-metre walk test); neuropsychological battery (Beck depression; MSQOL-54 quality of life; Goal Attainment Scaling); usability (System Usability Scale)
Moore et al, 2008	USA	RCT	38 people with MS (20 intervention, 18 control), 21% male, mean age 53	Right-handed; non-smoker; Extended Disability Status Scale 3.5-7; at least 5 MS brain lesions on MRI; stable on immunomodulatory therapy; less than 2 exacerbations in past 12 months	Active exacerbation phase; treatment with pulse-cortical steroids in past 3 months; history of seizure disorder, traumatic brain injury or substance abuse; taking cognition-enhancing acetylecho-linesterase inhibitors	Verbal learning and memory (adapted Auditory-Visual Learning Test) – sensitivity index; response bias index

Moumdjian et al, 2019	Belgium	Case-control study	31 people with MS (26% male, mean age 53). 30 healthy controls (27% male, mean age 52). Note that only 27 people with MS and 30 healthy controls completed the experimental session. Note that the 'controls' were not a comparator group in terms of intervention – they received the same MBTs, the only difference was they did not have MS	Aged at least 18; diagnosis of MS for over 1 year; no exacerbation in past month; average comfortable walking speed 0.4-1.2 m/s	Pregnancy; hearing impairment; cognitive impairment hindering understanding of instructions	Primary: Auditory-motor coupling and synchronisation<br><br>Secondary: Perceived physical and cognitive fatigue (Modified Fatigue Impact Scale); motivation (Likert); cadence; stride length; velocity
Naderi et al, 2022	Iran	Quasi-experimental controlled feasibility study	13 people with MS (0% male, mean age 38). 14 healthy controls (0% male, mean age 38). Note that the 'controls' were not a comparator group in terms of intervention – they received the same MBTs, the only difference was they did not have MS	MS confirmed by neurologist; Expanded Disability Status Scale 3.5-5.5; no vision or hearing problems; no other active treatment; able to walk without assistive devices	Unable to perform tasks; severe attacks or relapse on day of the test	Primary: Gait (Gait Symmetry Index) – step duration symmetry; stride length symmetry<br><br>Secondary: Additional gait parameters
Schmid et al, 2004	USA	Matched control study	20 people with MS (10 per arm), 30%	Not specifically stated (only exclusion criteria)	Pregnancy; mental disorders requiring medication; previous	Depression (Beck); anxiety (HADS); self-acceptance (Social

			male, age range 29-47		experience of music therapy	and Emotional Skills Assessment); quality of life (Hamburg Quality of Life Questionnaire in MS); cognitive and functional disability (Expanded Disability Status Scale)
Seebacher et al, 2024	Austria	RCT	127 people with MS (split 43, 44, 40 across the 3 music therapy arms), 25% male, mean age per arm range 47-50	Age 18 or over; MS diagnosis (McDonald criteria); Expanded Disability Status Scale 2-5; stable disease	Cognitive impairment (MoCA <26), anxiety or depression (HADS subscales 11 or over); suicidality; relapse, starting new medication, medication adjustment or physiotherapy in past 3 months or during the study	Primary: Walking speed (timed 25-foot walk); change in walking distance (2-minute walk test)<br><br>Secondary: Cognition (MoCA); anxiety and depression (HADS); suicidality (narrative screening); fatigue (Neurological Fatigue Index-MS); quality of life (MS International Quality of Life); motor imagery (Kinesthetic and Visual Imagery Questionnaire-10)
Seebacher et al, 2019	Austria	RCT	59 people with MS (split 19, 20, 20 across the 3 music therapy arms), 20% male, mean age 45	Aged 18 or over; German speaking; clinically definite MS (McDonald criteria); mild-to-moderate disability (Expanded Disability Status Scale 1.5-4.5)	Concomitant diseases affecting interventions or walking; relapse in past 3 months or during the study; change of treatment in past 2 months or	Primary: Walking speed (timed 25-foot walk); change in walking distance (2-minute walk test)<br>Secondary: Fatigue (Modified Fatigue Impact Scale); quality

					during the study; pregnancy; clinical symptoms of depression or cognitive dysfunction	of life (MS Impact Scale-29); motor imagery (Kinesthetic and Visual Imagery Questionnaire-10); sensory motor synchronisation
Seebacher et al, 2018	Austria	Feasibility RCT	15 people with MS (split 5, 5, 5 across the 3 music arms), 13% male, mean age 52	Age 18 or over; MS (McDonald criteria); mild-to-moderate disability (Expanded Disability Status Scale 1.5-4.5); German speaking	Concomitant diseases affecting rhythmic cued motor imagery or walking; relapse in past 3 months or during the study; change of medication or start of physiotherapy in past 2 months; pregnancy; overt symptoms of depression or cognitive dysfunction	Primary: Feasibility; safety and adverse events<br><br>Secondary: Walking speed (timed 25-foot walk); walking distance (6-minute walk test); fatigue (Modified Fatigue Impact Scale); quality of life (MS Impact Scale-29); motor imagery (Kinesthetic and Visual Imagery Questionnaire-10)
Seebacher et al, 2017	Austria and UK	RCT	101 people with MS (34 for each of the 2 music arms, 33 controls), 16% male, mean age 44	Aged 18 or over; clinically definite MS (McDonald criteria); mild-to-moderate disease (Expanded Disability Status Scale 1.5-4.5)	Concomitant diseases affecting interventions or walking; relapse in past 3 months; change of medication or physiotherapy in past 2 months; pregnancy; overt symptoms of depression or cognitive dysfunction	Primary: Walking speed (timed 25-foot walk); change in walking distance (2-minute walk test)<br><br>Secondary: Walking perception (MS Walking Scale-12); fatigue (Modified Fatigue Impact Scale); quality of life

						(MS Impact Scale-29)
Seebacher et al, 2015	Austria	Feasibility RCT	30 people with MS (10 for each of the 2 music arms, 10 controls), 27% male, mean age per arm ranged 42-47	Aged 18 or over; diagnosis of MS (McDonald criteria); mild-to-moderate disability (Expanded Disability Status Scale 1.5-4.5); German speaking	Concomitant diseases affecting rhythmic cued motor imagery or walking; relapse in past 3 months or during the study; medication change in past 2 months; pregnancy; overt symptoms of depression or cognitive dysfunction	Primary: Feasibility; fatigue (Modified Fatigue Impact Scale)<br><br>Secondary: Walking speed (timed 25-foot walk); change in walking distance (2-minute walk test)
Seighalani et al, 2014	Iran	RCT	30 people with MS (15 per arm), gender and age not reported	MS diagnosis by physician; self-esteem below average (Cooper Smith <25)	Cooper Smith self-esteem 25 or above	Depression (Beck); anxiety (Beck); self-esteem (Cooper Smith)
Shahraki et al, 2017	Iran	RCT	18 people with MS (9 per arm), 22% male, mean age intervention 40, control 38	Age 18 or over; Expanded Disability Status Scale 3-6; able to walk 30.48m without assistance; lack of treatment for relapse or MS exacerbation in past 30 days; lack of cardiovascular and rheumatic diseases; lack of severe pain in lower joints; no engagement in regular physical	No additional criteria	Gait parameters

				activity in past 3 months; no hearing impairment		
Thaut et al, 2008	USA	RCT	20 people with MS (10 per arm), 10% male,<br>mean age: intervention 51.7, control 53.8	Right-handed; relapse-remitting MS; normal hearing; no history of other neurological or psychiatric conditions	No additional criteria	Verbal learning and word order memory<br>Recall of higher order word sequences (3–7 words)
Thaut et al, 2014	Germany and USA	RCT	54 people with MS (27 intervention, 27 control), 30% male, mean age intervention 50, control 53	Right-handed; relapse-remitting MS; normal hearing; no history of other neurological or psychiatric conditions; at least 5 brain lesions on MRI; stable on immunomodulatory therapy; <2 exacerbations in past 12 months	In active exacerbation phase; treated with pulse-cortical steroids or cognition-enhancing AChE inhibitors	Learning; memory; brain activity with oscillatory network synchronisation
Wiens et al, 1999	Canada	RCT	19 people with MS (9 intervention, 10 control), gender not reported, mean age 55	Residents in a long-term care centre with MS; able to tolerate 30 minutes of concentrated effort; able to follow verbal instructions; MMSE 20 or above; free from respiratory infection; able to sit in a wheelchair for at least 2 hours	No additional criteria	Strength of respiratory muscles (mouth pressure testing) – inspiratory and expiratory

Young et al, 2019	USA	RCT	81 people with MS (53 intervention across 2 arms, 28 control), 19% male, mean age per arm ranged 47-50	Aged 18-65; self-reported MS diagnosis; Patient-Determined Disease Steps 0-6; able to exercise with arms and/or legs; physician clearance	Participation in similar intervention in past 6 months; use of tobacco in past 6 months; unstable weight; cognitive impairment (MMSE <24); active pressure ulcer; contraindications to exercise (American College of Sports Medicine guidelines)	Primary: Motor evaluation (Timed Up and Go; 6-minute walk test; 5-times sit-to-stand)<br><br>Secondary: Fatigue (Patient-Reported Outcomes Measurement Information System Fatigue); pain (Pain Interference Short Form 8a.)

Supplemental file 7. Intervention profile for included studies.

Author, year	Content	Leader	Location	Duration
Aldridge et al. 2005	Nordoff Robbins approach: Active music therapy - Music-making on instruments, or singing	Therapist	Health facility	3 blocks of music therapy in single sessions over the course of the project (8—10 sessions, respectively)
Conklyn et al, 2010	Rhythmic auditory stimulation	Self	Home	2 sessions of 20 min/day for 4 weeks
Gatti et al, 2015	Playing keyboard (numbered, coloured stickers to guide)	Physiotherapist	Health facility	30 minutes a day, 5 days a week for 3 weeks
Goetschalckx et al, 2021	Rhythmic auditory stimulation with metronome	Facilitator	Health facility	1 single session (each metronome condition lasted 1 minute with a 1-minute rest in between conditions)
Helminger et al, 2025	Actual and/or imagined gait training with rhythmic-auditory cueing	Self	Home	4 sessions of 30 minutes per week for 4 weeks
Impellizzeri et al, 2020	Neurologic music therapy (plus conventional cognitive rehabilitation)	Music therapist	Health facility	3 sessions of 60 minutes per week for 8 weeks
Kuhlmann et al, 2025	Listening to a monochord being played while lying on top of it and feeling the vibration	Music therapist	Health facility	1 session of 45 minutes per week for 6 weeks
Maggio et al, 2021	Listening to music while walking on a treadmill	Physiotherapist	Health facility	3 sessions of 30 minutes per week for 8 weeks
Moore et al, 2008	Hearing word lists in song format and being asked to sing them back	Research team	Health facility	Single session (testing time approximately 60 minutes)

Moumdjian et al, 2019	Rhythmic auditory stimulation (2 groups: music and metronome)	Research team	Health facility	Single session of 12 minutes
Naderi et al, 2022	Rhythmic auditory stimulation	Facilitator	Health facility	Single session (4 walking modes, each 2 minutes walking with 30s rhythm exposure beforehand, and 3-minute rest between modes)
Schmid et al, 2004	Active music therapy (singing or instruments)	Music therapist	Health facility	3 blocks of 8-10 sessions over 1 year
Seebacher et al, 2024	3 groups: cued motor imagery; combined cued motor imagery and cued gait training; cued gait training	Physiotherapist, occupational therapist, psychologist	Home	4 sessions of 30 minutes (including short breaks) per week for 4 weeks
Seebacher et al, 2019	3 groups: music and verbally cued motor imagery; music cued motor imagery; non-cued motor imagery	Self	Home	6 sessions of 17 minutes per week for 4 weeks
Seebacher et al, 2018	3 groups: music and verbally cued motor imagery; music cued motor imagery; non-cued motor imagery	Self	Home	6 sessions of 17 minutes per week for 4 weeks
Seebacher et al, 2017	2 groups: music cued motor imagery; metronome cued motor imagery	Self	Home	6 sessions of 17 minutes per week for 4 weeks
Seebacher et al, 2015	2 groups: music cued motor imagery; metronome cued motor imagery	Self	Home	6 sessions of 17 minutes per week for 4 weeks
Seighalani et al, 2014	Active music therapy (discussion about music; making music with mouth; expressing the effects of music) and passive music therapy (listening to music)	Researcher and music therapist	Health facility	15 sessions of 30 minutes in total (6 active and 9 passive)

Shahraki et al, 2017	Rhythmic auditory stimulation with metronome	Coach	Health facility	3 sessions of 30 minutes per week for 3 weeks
Thaut et al, 2008	Ray's Auditory Verbal Learning Test – sung word lists	Research team	Health facility	Single session
Thaut et al, 2014	Ray's Auditory Verbal Learning Test – sung word lists	Research team	Health facility	Single session
Wiens et al, 1999	Music therapy: focusing on singing: relaxation and diaphragmatic breathing, intonation of syllables, and reading or singing phrases, paragraphs, and simple songs	Music therapist with instrument speciality in voice	Health facility	3 sessions of 30 minutes per week for 12 weeks
Young et al, 2019	2 groups: music to movement; adapted yoga. The yoga group does not meet the inclusion criteria as an intervention for this review and is not presented as a comparator.	Self	Fitness facility	3 sessions of 60 minutes per week for 12 weeks

Supplemental file 8. Control profile for included studies.

**Author, year**	**Synopsis of control arm**
Aldridge et al. 2005	No Intervention
Conklyn et al, 2010	No intervention
Gatti et al, 2015	Playing the keyboard with it turned off
Goetschalckx et al, 2021	Healthy control group who had the same intervention
Helminger et al, 2025	Healthy control group who had no music therapy
Impellizzeri et al, 2020	6 sessions per week for 8 weeks of conventional cognitive rehabilitation
Kuhlmann et al, 2025	Patients laid on top of a monochord and remained relaxed in a supine position in silence
Maggio et al, 2021	Conventional cognitive rehabilitation (same amount of conventional physiotherapy, stretching, muscle strength and occupational therapy as intervention group)
Moore et al, 2008	Spoken word group – learn and recall words without music
Moumdjian et al, 2019	Healthy control group who had no music therapy or metronome
Naderi et al, 2022	Healthy control group who had the same intervention
Schmid et al, 2004	No intervention
Seebacher et al, 2024	[No control arm]
Seebacher et al, 2019	[No control arm]
Seebacher et al, 2018	[No control arm]
Seebacher et al, 2017	No music therapy
Seebacher et al, 2015	No music therapy
Seighalani et al, 2014	No intervention
Shahraki et al, 2017	Exercises as per intervention group but without rhythmic auditory stimulation
Thaut et al, 2008	Spoken word list (same words as sung by intervention group)
Thaut et al, 2014	Spoken 15-word list (same words as sung by intervention group)
Wiens et al, 1999	3 sessions of 30 minutes per week for 12 weeks with a nurse – listened to music, discussed music, learnt about music and composers (but no active music therapy)

Young et al, 2019	No active music therapy or yoga

Supplementary file 9. Narrative results for included studies

**Author, year**	**Results**
Aldridge et al. 2005	There were no statistically significant differences between the music therapy group and the control group; however, medium effect sizes were observed for self-esteem (d = 0.54), depression (HAD-D d = 0.63), and anxiety (HAD-A d = 0.63). Within the music therapy group, significant improvements in self-esteem, depression, and anxiety were seen over the treatment period (U1–U4). No statistically significant improvement in quality of life was observed, although a trend towards improvement was reported (HAQUAMS p = 0.066). No differences were found for the functional and physiological values (MSFC, EDSS).
Conklyn et al, 2010	Compared to no intervention, rhythmic auditory stimulation was associated in statistically significant greater improvement in double-support time (left, p = .0176; right, p = .0247). No statistically significant between-group differences were found for the other outcome measures. However, trends with medium to high effect sizes were observed for other gait parameters including walking speed. Within-group analysis showed statistically significant improvement in cadence, stride length, step length, velocity, and normalized velocity. Participant satisfaction level with the rhythmic auditory stimulation intervention was high.
Gatti et al, 2015	Compared to playing with the keyboard turned off, playing with the keyboard turned on was associated with a statistically significant improvement in hand function (p=0.003, Cohen's d = 1.66), while both groups showed a statistically significant improvement in hand dexterity, although between-group differences were not statistically significant. There was also no statistically significant between-group difference in hand strength.
Goetschalckx et al, 2021	Reported results that people with MS performed more poorly than healthy controls were not surprising and outside the scope of the present review. Within people with MS, interlimb coordination was worse at 0.75Hz metronome frequency compared to 1.00Hz and 1.50Hz. Movement amplitude was greatest at 1.00 Hz but reduced at both 0.75Hz and 1.50HZ. Movement frequency followed the metronome tempo. Poorer coordination was statistically significantly correlated with poorer cognitive performance at 0.75Hz and 1.50Hz metronome frequencies, and with reduced walking capacity especially at the 1.50Hz metronome frequency.
Helminger et al, 2025	In people with MS, walking distance in the 2-minute walk test showed a statistically significant improvement only in those classified as responders (>5% improvement), while no statistically significant improvement was shown in the timed 25-foot walk test. Responders showed reductions in brain activation within the premotor cortex, precuneus, and middle frontal gyrus, areas linked to motor planning, spatial guidance, and attention. Across people with MS, greater decreases in brain activation in these regions showed a statistically significant correlation with greater improvements in the 2-minute walk test. There was no observed statistically significant association between walking function and motor imagery-related brain activation changes.
Impellizzeri et al, 2020	The music therapy group showed statistically significant improvements in cognitive function (p < .001), long-term retrieval (p= 0.007), delayed recall (p=0.001), and on multiple subscales of motivation, emotional awareness, depression, and quality of life. The between-group effect versus conventional cognitive rehabilitation did not show any statistically

	significant differences (either better or worse). It should be noted that conventional cognitive rehabilitation was administered to both arms.
Kuhlmann et al, 2025	Compared to the silent condition, monochord music therapy was associated with a statistically significant reduction in psychosocial fatigue (p=0.029), increased heat pain thresholds (p=0.024) and stronger immediate subjective effects (e.g. feeling balanced: p < 0.001, relaxed: p < 0.001, less pain: p < 0.001). However, there was no statistically significant between-group difference in anxiety levels (p=0.109).
Maggio et al, 2021	Participants receiving music-assisted treadmill training demonstrated significant improvements in balance (BBS, p<0.002), walking speed (10mWT, p<0.002), mobility (TUG, p<0.002), mood (BDI-II, p<0.002), physical QoL (MSQOL-PH, p<0.002), mental QoL (MSQOL-MH, p<0.001), and goal attainment (GAS, p<0.002). Compared with conventional gait training, significantly greater improvements were observed in walking speed (p=0.004), mobility (p=0.001–0.003), physical QoL (p=0.004), mental QoL (p<0.001), and goal attainment (p=0.002).
Moore et al, 2008	There were no statistically significant differences between the spoken word and sung groups for recognition accuracy, false alarms, sensitivity or response bias.
Moumdjian et al, 2019	Participants were able to synchronize to the different tempi. People without cognitive impairment showed statistically significantly higher synchronization with the metronome compared with music (p < 0.001). People with MS perceived statistically significantly less physical (p < 0.0001) and cognitive fatigue (p < 0.0001) when walking to music. People with cognitive impairment perceived lower fatigue walking to music compared with metronomes (p < 0.0001).
Naderi et al, 2022	Rhythmic auditory stimulation statistically significantly improved gait symmetry and spatiotemporal parameters in MS patients. At 90% tempo, stride length, stride width, and double support duration increased, while cadence decreased. At 110% tempo, cadence increased while stride length, stride width, and double support duration decreased, indicating improved efficiency and stability. Across all tempi (90%, 100%, 110%), gait symmetry indices improved compared to walking without rhythmic auditory stimulation.
Schmid et al, 2004	No significant between-group differences were observed in functional status (MSFC), self-acceptance, depression, anxiety, or quality of life at the end of treatment. However, medium effect sizes favoured music therapy for self-esteem (d=0.54), depression (d=0.63), and anxiety (d=0.63). Within the music therapy group, significant improvements were observed in self-acceptance (p=0.012), depression (p=0.035–0.036), and anxiety (p=0.013), while no significant changes were found in functional status or quality of life.
Seebacher et al, 2024	Music-cued motor imagery, combined motor imagery and gait training, and conventional gait training (CGT) all improved walking performance, fatigue, and several domains of health-related quality of life. However, no significant between-group differences or interaction effects were observed for quality of life outcomes. Reductions in physical, cognitive, and total fatigue were reported across all groups, with a significant between-group difference favouring CGT for cognitive fatigue (p=0.048). No significant effects were reported for anxiety, depression, or cognitive function.

Seebacher et al, 2019	All music interventions statistically significantly improved walking speed and distance. Motor outcomes, fatigue and quality of life significantly improved only after music and verbally cued motor imagery. Synchronisation improved more with music and verbal motor imagery and music motor imagery and worsened with just motor imagery.
Seebacher et al, 2018	Feasibility was demonstrated. There were no safety-related or adverse events related to the study. Improvements in all groups for walking speed and distance, with greatest improvement in the music and verbally cued motor imagery group. Improvements in fatigue, quality of life and motor imagery ability were observed. Only one participant (in the music and verbally cued motor imagery group) showed a clinically significant improvement (defined as at least 20%) in the timed 25-foot walk test. 60% of participants in the music and verbally cued motor imagery group and 40% of participants in other groups showed a clinically significant improvement (defined as at least 20%) in the 6-minute walk test. Fatigue decreased and QoL improved following music-cued and non-cued motor imagery interventions, although findings were exploratory as this is a feasibility study.
Seebacher et al, 2017	Music-cued and metronome-cued motor imagery significantly improved walking speed and walking distance compared with controls (both p<0.0001). Cognitive and total fatigue improved in both intervention groups, while physical fatigue, vitality, and several QoL domains improved only after music-cued motor imagery.
Seebacher et al, 2015	Motor imagery with instrumental music and metronome cues statistically significantly improved walking speed and distance in intervention groups compared to no music.
Seighalani et al, 2014	Compared to no intervention, music therapy significantly reduced depression and anxiety from pretest to post-test and follow-up, with effects persisting over time. Depression scores decreased markedly (39.13 to ~17), and anxiety scores halved (41.07 to ~21). Self-esteem scores improved substantially, rising from 16.00 at pretest to ~37–38 at post-test and follow-up. While differences between post-test and follow-up were minimal, all outcomes showed significant improvements compared to pretest.
Shahraki et al, 2017	Statistically significant improvements in the gait parameters stride length, time, double support time, cadence, and gait speed were found in the rhythmic auditory stimulation group compared to the control group doing the same exercises without rhythmic auditory stimulation.
Thaut et al, 2008	Music-based learning was associated with superior word order recall compared with spoken learning,(F(1,18) = 4.51, p = 0.038). Musical learning led to greater improvements in early and late learning phases, while spoken learning showed only transient gains that reduced toward the end of the task.
Thaut et al, 2014	The sung word list group showed statistically significantly more improvement in word order memory and recall (p= 0.038) and bilateral frontal alpha learning related synchronization (p= 0.05) than the spoken word group.
Wiens et al, 1999	Compared to passive music engagement, active music therapy was not shown to be associated with statistically significant improvements in the strength of respiratory muscles in people with advanced MS.
Young et al, 2019	Statistically significant improvements for music to movement versus no active music therapy or yoga were shown for in Timed Up and Go (least square mean difference [95% confidence interval] = −1.9s [−3.3 to −0.5], *P*=.01, *d*=0.7 and 6-minute walk test (41.0m [2.2-80.0], *P*=.04, *d*=0.6; controlled for Patient-Determined Disease Steps). No significant group

	differences were found in 5-times sit-to-stand, fatigue, and pain interference. Tests of music to movement versus control were post-hoc as the study also contained an active yoga intervention and the primary comparisons were made across music to movement, active yoga and control.

Supplementary file 10. Risk of bias results

Risk of bias and methodological quality were assessed using the Critical Appraisal Skills Programme (CASP) checklists, selected according to study design (randomised controlled trials, cohort studies, case control studies, and cross-sectional studies). CASP was chosen to provide a structured, domain-based assessment of internal validity, applicability, and reporting quality, rather than a single summary score. Interpretation focused on domain level patterns of bias, consistent with recommendations to avoid reliance on composite quality scores.
Across the 23 included studies, the RCT checklist was the most frequently applied, reflecting that most studies were experimental in design. Where previous reviews had inconsistently labelled some studies as non-randomised, quasi experimental, or clinical trials, the present review applied CASP checklists based on the actual methodological features reported, rather than labels used in earlier syntheses. This resulted in three intentional mismatches between study labels used in prior reviews and the CASP checklist applied here. For example, Maggio et al (2021) and Naderi et al (2022) were assessed using the RCT checklist despite being described as quasi experimental in some summaries, as both involved prospective intervention allocation and comparator groups. Conversely, Aldridge et al (2005) and Schmid et al (2004) were assessed using the cohort checklist, as allocation was matched rather than randomised. These decisions were made to ensure the most appropriate appraisal tool was applied to each study’s actual design.

Overall, most studies addressed a clearly focused research question, reported outcomes comprehensively, and used validated outcome measures. Baseline characteristics were generally comparable between intervention and control groups where applicable. However, several recurring sources of bias were identified across studies. Blinding of participants and intervention providers was rarely possible, reflecting the nature of music-based interventions. While some studies reported assessor blinding, this was inconsistently applied or insufficiently described. Allocation concealment was rarely reported and may represent either a reporting limitation or a true methodological weakness.

Sample size was a common concern. Many studies were small, exploratory, or pilot in nature, limiting statistical power and increasing the risk of type II error. Participant flow and attrition were not always clearly reported, particularly in older studies and non-randomised designs, raising potential concerns regarding selection and attrition bias. Although most studies reported statistically significant findings where present, clinical significance was rarely addressed, and confidence intervals or precision estimates were inconsistently reported.

Observational and non-randomised studies generally demonstrated appropriate measurement of exposure and outcomes, but often relied on convenience sampling, increasing the risk of selection bias. Cross sectional and case control designs were limited in their ability to account for confounding, although the case control study assessed using CASP demonstrated strong internal consistency across domains. Cohort studies scored highly overall but lacked detailed reporting of precision and effect estimates.

Several important limitations were identified that are not fully captured by CASP or other standard risk of bias tools. These include heterogeneity in intervention content, duration, and delivery personnel; variability in outcome measures across studies; frequent underrepresentation of male participants; and reliance on self-reported outcomes in several domains. In addition, some single session experimental studies lacked a comparator capable of controlling for non-specific effects such as attention, novelty, or participant expectation.

Despite these limitations, higher quality RCTs demonstrated generally good internal validity, clear outcome reporting, and appropriate analytical approaches. Studies rated as high quality were more likely to include blinded outcome assessment, clearer reporting of statistical methods, and better alignment between results and conclusions. Across study designs, conflicts of interest were not identified as a major concern, and authors typically acknowledged at least some methodological limitations.

Overall, the risk of bias profile suggests that while many studies were thoughtfully designed and conducted within the practical constraints of arts-based research, methodological limitations were common, particularly relating to blinding, sample size, and reporting transparency. These factors should be considered when interpreting the strength and consistency of the evidence base.

CASP risk of bias checklists

Part A: RCT checklist

**Study**	**Clear research question?**	**Randomised?**	**All participants accounted for?**	**Participants blinded?**	**Investigators blinded?**	**Assessors blinded?**	**Similar baselines?**
Conklyn et al, 2010	Yes	Yes	Yes	No	No	No	Yes
Gatti et al, 2015	Yes	Yes	Yes	No	No	No	Yes
Helminger et al. 2025	Yes	Yes	Yes	No	No	No	Yes
Impellizzeri et al, 2020	Yes	Yes	Yes	Yes	No	No	Yes
Kuhlmann et al, 2025	Yes	Yes	No	No	Yes	No	Yes
Maggio et al. 2021	Yes	No	Yes	No	No	No	Yes
Moore KS. et al. 2008	Yes	Yes	Yes	No	No	No	Yes
Naderi S. Et al. 2022	Yes	No	Yes	No	No	No	Yes
Seebacher et al, 2015	Yes	Yes	Yes	No	No	No	Yes
Seebacher et al, 2017	Yes	Yes	Yes	No	No	No	Yes
Seebacher et al, 2018	Yes	Yes	Yes	No	No	No	Yes
Seebacher et al, 2019	Yes	Yes	Yes	No	No	No	Yes

Seebacher et al, 2024	Yes	Yes	Yes	Yes	Yes	No	Yes
Seighalani et al, 2014	Yes	Yes	Yes	No	No	No	Yes
Shakraki et al, 2017	Yes	Yes	Yes	No	No	No	Yes
Thaut et al, 2008	Yes	yes	Yes	No	No	No	Yes
Thaut et al, 2014	Yes	Yes	Yes	No	Yes	No	Yes
Wiens et al, 1999	Yes	Yes	Yes	No	No	No	Yes
Young et al, 2019	Yes	Yes	Yes	No	No	Yes	Yes

**Study**	**Same level of care?***	**Effects of intervention reported comprehensively?**	**Precision of treatment effect reported?**	**Benefits outweigh harms and costs?**	**Applicable to our context?**	**Greater value than existing treatment?**
Conklyn et al, 2010	Yes	Yes	No	Yes	Yes	Can’t tell
Gatti et al, 2015	Yes	Yes	Yes	Yes	Yes	Can’t tell
Helminger et al. 2025	Yes	Yes	Yes	Yes	Yes	Can’t tell
Impellizzeri et al, 2020	Yes	Yes	Yes	Yes	Yes	Can’t tell
Kuhlmann et al, 2025	Yes	Yes	Yes	Yes	Yes	Can’t tell
Maggio et al. 2021	Yes	Yes	No	Yes	Yes	Can’t tell
Moore. et al. 2008	Yes	Yes	No	Yes	Yes	Can’t tell
Naderi. et al. 2022	Yes	Yes	No	Yes	Yes	Can’t tell
Seebacher et al, 2015	Yes	Yes	Yes	Yes	Yes	Can’t tell
Seebacher et al, 2017	Yes	Yes	Yes	Yes	Yes	Can’t tell

Seebacher et al, 2018	Yes	Yes	Yes	Yes	Yes	Can't tell
Seebacher et al, 2019	Yes	Yes	Yes	Yes	Yes	Can't tell
Seebacher et al, 2024	Yes	Yes	Yes	Yes	Yes	Can't tell
Seighalani et al, 2014	Yes	Yes	No	Yes	Yes	Can't tell
Shakraki et al, 2017	Yes	Yes	No	Yes	Yes	Can't tell
Thaut et al, 2008	Yes	Yes	No	Yes	Yes	Can't tell
Thaut et al, 2014	Yes	Yes	No	Yes	Yes	Can't tell
Wiens et al, 1999	Yes	Yes	No	Yes	Yes	Can't tell
Young et al, 2019	Yes	Yes	Yes	Yes	Yes	Can't tell

* = apart from intervention

Part B: Cross-sectional checklist

**Study**	**Clear research question?**	**Appropriate method?**	**Acceptable recruitment?**	**Accurate measurement to reduce bias?**	**Data collected in a way that addressed research issue?**	**Enough participants?**	**Clear results presentation?**
Goetschalckx et al, 2021	Yes	Yes	Yes	Yes	Yes	Can't tell	Yes

**Study**	**Rigorous analysis?**	**Clear statement of findings?**	**Applicable to local population?**	**How valuable is the research?**
Goetschalckx et al, 2021	Yes	Yes	Yes	Can't tell

Part C: Case control checklist

Study	Clearly focused issue?	Appropriate method?	Acceptable recruitment?	Acceptable control selection?	Exposure accurately measured to minimise bias?	Groups similar apart from exposure?	Confounding taken into account in design and analysis?
Moumdjian et al, 2019	Yes	Yes	Yes	Yes	Yes	Yes	Yes

Study	Large treatment effect?	Precise estimate of treatment effect?	Do you believe the results?	Applicable to our context?	Results fit with other available evidence?
Moumdjian et al, 2019	Can't tell	Can't tell	Yes	Yes	Yes

Part D: Cohort checklist

Study	Clearly focused issue?	Acceptable recruitment?	Exposure accurately measured to minimise bias?	Outcome accurately measured to minimise bias?	All important confounders identified?	Confounding taken into account in design and analysis?	Follow-up complete enough?
Aldridge et al. 2005	Yes	Yes	Yes	Yes	Yes	Yes	Yes
Schmid et al, 2004	Yes	Yes	Yes	Yes	Yes	Yes	Yes

Study	Clear results presentation?	How precise are the results?	Do you believe the results?	Applicable to local population?	Results fit with other available evidence?	Clear implications for practice?
Aldridge et al. 2005	Yes	Can't tell	Yes	Yes	Yes	Can't tell
Schmid et al, 2004	Can't tell	No	Yes	Yes	Yes	Can't tell

Supplemental file 11: Assessment of reporting bias

Study	Protocol available?	Protocol outcomes not reported in journal
Aldridge et al (2005)	No	Not evaluable
Conklyn et al (2010)	No	Not evaluable
Gatti et al (2015)	No	Not evaluable
Goetschalckx et al (2021)	NCT04142853 and NCT03938558	Primary outcomes from the protocol not reported in journal: Stroop test, Nine-hole peg test, Fatigability index hand, motor fatigability, trait motor fatigue*
Helminger et al (2025)	DRKS00023978 (German trials registry) **	All primary outcomes from the protocol were reported in the journal – some secondary clinical and psychological outcomes were not
Impellizzeri et al (2020)	No	Not evaluable
Kuhlmann et al (2025)	DRKS00024549	All protocol outcomes reported in journal
Maggio et al (2021)	No	Not evaluable
Moore et al (2008)	No	Not evaluable
Moumdjian et al (2019)	NCT03281330	All protocol outcomes reported in journal
Naderi et al (2022)	No	Not evaluable
Schmid et al (2004)	No	Not evaluable
Seebacher et al (2015)	ISRCTN67054113	Primary outcomes from protocol (T25FW + 6MWT) switched to secondary outcomes in the journal, one of the secondary outcomes from the protocol (MFIS) switched to primary outcome in the journal*
Seebacher et al (2017)	ISRCTN67054113	All protocol outcomes reported in journal
Seebacher et al (2018)	ISRCTN92351899***	Some of the primary outcomes from the protocol (T25FW and 6MWT) switched to secondary outcomes in the journal
Seebacher et al (2019)	ISRCTN92351899***	All protocol outcomes reported in journal
Seebacher et al (2024)	DRKS00023978**	All protocol outcomes reported in journal
Seighalani et al (2014)	No	Not evaluable
Shahraki et al (2017)	No	Not evaluable
Thaut et al (2008)	No	Not evaluable
Thaut et al (2014)	No	Not evaluable
Wiens et al (1999)	No	Not evaluable
Young et al (2019)	NCT02533882	Primary outcomes from the protocol not reported in journal: VO2 fitness, pain, fatigue, loneliness, grip strength, balance tests*

All protocols were published prospectively rather than retrospectively, * there were also secondary outcomes from the protocol not reported in the journal (however where primary outcomes from the protocol are not reported in the journal we focus on listing these in the table), ** while these studies share a protocol registration number, they are separate studies with different aims and outcomes (total sample size across the two studies = 147, the sample sizes for the two separate studies are 121 and 31 considering only included participants, suggesting an overlap of 5 participants - 3% of the total sample size across the two studies), *** while these two studies share a protocol registration number, they are separate studies and have no overlap in participants (total sample size across the two studies = 75, the sample sizes for the two separate studies are 15 and 60).

Supplemental file 12. Effect direction plot

Study	Study Design	Motor outcomes	Cognitive outcomes	Psychological outcomes	Quality of life outcomes
Aldridge D. et al. 2005	Matched controlled pilot study		◄►	▲	◄►
Conklyn D. et al. 2010	RCT	▲			◄►
Gatti R. Et al. 2015	RCT	▲			
Goetschalckx M. et al. 2021	Observational cross-sectional comparative study	◄►			
Helminger B. et al. 2025	RCT	◄►			
Impellizzeri et al, 2020	RCT		▲	▲	▲
Kuhlmann J et al. 2025	RCT			▲	▲
Maggio et al. 2021	Quasi-experimental controlled feasibility study	▲	▲	▲	▲
Moore KS. et al. 2008	RCT		◄►		
Moumdjian L. et al. 2019	Observational case control study	▲			▲
Naderi S. Et al. 2022	Quasi-experimental controlled study with repeated measures	▲			
Schmid et al. 2014	Matched control study	◄►	◄►	▲	◄►
Seebacher B. et al. 2015	RCT	▲			▲
Seebacher B. et al. 2017	RCT	▲			▲
Seebacher B. et al. 2018	RCT	▲			▲

Seebacher B. et al. 2019	RCT	▲			▲
Seebacher B. et al. 2024	RCT	▲	◄►	◄►	▲
Seighalani M. et al. 2014	RCT			▲	
Shahraki M et al, 2017	RCT	▲			
Thaut et al. 2008	RCT		▲		
Thaut M. et al. 2014	RCT		▲		
Wiens M E et al. 1999	RCT	◄►			
Young HJ. Et al. 2019	RCT	▲			◄►

Legend:
Study design: RCT: Randomised Controlled Trial. Effect direction: upward arrow ▲= positive health impact, downward arrow ▼= negative health impact, sideways arrow ◄►= no change/mixed effects/conflicting findings. Sample size: Final sample size (individuals) in intervention group Large arrow ▲ >300; medium arrow ▲ 50-300; small arrow ▲ <50. Following Boon and Thomson[82]

Supplemental file 13. Evaluation of minimally clinically important differences

Outcome domain	Measure	MCID	Findings
Motor	Timed 25-foot walk test	17.2% improvement[76]	• Helminger et al[49] – no clinically significant difference in participants receiving actual and/or imagined gait training with rhythmic-auditory cueing from baseline to follow-up. **Within-group**<br>• Seebacher et al[55] – clinically significant difference in participants receiving music cued motor imagery (17.3%) and metronome cued motor imagery (20.0%), but not controls, from baseline to follow-up. **Within-group**<br>• Seebacher et al[56] – no clinically significant difference in any group from baseline to follow-up. **Within-group**<br>• Seebacher et al[57] – no group-level clinically significant difference in any group from baseline to follow-up. One out of five participants in the music- and verbally cued motor imagery group had a clinically significant difference and no participants in other groups. **Within-group**<br>• Seebacher et al [58] – no group-level clinically significant difference in any group from baseline to follow-up. 21.1% of participants in the in the music- and verbally cued motor imagery group, 15.0% in the music-cued motor imagery and none in the motor imagery group had a clinically significant difference (defined as >=20% by the authors of this study). **Within-group**<br>• Seebacher et al[59] – no clinically significant difference in any group from baseline to the final follow-up (week 13). **Within group**<br><br>One study out of six found evidence of a clinically significant difference. All assessed within-group rather than between-group differences, although for some studies information was available for more than one group.

Psychological	Beck depression	17.5% reduction[77]	• Impellizeri et al[32] – clinically significant difference in experimental group (46.4%) but not the control group from baseline to follow-up.<br>**Within-group**<br>• Maggio et al[51] – clinically significant difference in experimental group (56.2%) but not the control group from baseline to follow-up.<br>**Within-group**<br>• Schmid et al[30] - numerical descriptive results not presented, therefore not evaluable<br>Two studies out of two (that were evaluable) found evidence of a clinically significant difference. Both assessed within-group rather than between-group differences, although information was available for more than one group.
Psychological	HADS anxiety	0.80 points (for subscale)[79]	• Kuhlmann et al[50] – no clinically significant difference from baseline to follow-up for the experimental group, although a clinically significant improvement (1.5 points) was found for the control group.<br>**Within-group**<br>• Schmid et al[30] – numerical descriptive results not presented, therefore not evaluable<br>• Seebacher et al[59] – secondary outcome but data not presented, therefore not evaluable<br>No studies out of one (that were evaluable) found evidence of a clinically significant difference. This study assessed within-group rather than between-group differences, although information was available for more than one group.
QoL	MS Impact Scale-29 (MSIS-29)	8 points for physical subscale[81]	• Seebacher et al[58] – clinically significant difference in the music- and verbally-cued motor imagery group (15 points), but not the music-cued motor imagery or motor imagery groups. 79% of participants in the music- and verbally cued motor imagery group, 50% of participants in the music-cued motor imagery group, and 35% of participants in the motor imagery group had a clinically

			significant difference, defined as 7.5 points by the authors of this study. **Within-group** • Seebacher et al[57] – no clinically significant difference in any group from baseline to follow-up (although the music-cued motor imagery group met the more lenient threshold of 7.5 points). **Within-group** • Seebacher et al[56] – no clinically significant difference in any group from baseline to follow-up. 50% of participants in the music- and verbally cued motor imagery group, 41.2% in the music-cued motor imagery group, and 21.2% of those in the motor imagery group had a clinically significant difference, defined as 7.5 points by the authors of this study. **Within-group** <br>One study out of three found evidence of a clinically significant difference. All assessed within-group rather than between-group differences, although information was available for more than one group.

Cognitive MCIDs could not be evaluated, as explained in Table 2.
