# Supplementary material for: Potential benefits of music-based therapies on rehabilitation outcomes in people with multiple sclerosis: a systematic review
Source: BMJ Open. 2026 Jul 22;16(7):e116420. doi: 10.1136/bmjopen-2026-116420 (PMC13410710; doi:10.1136/bmjopen-2026-116420)
Supplement: Reviewer comments [file bmjopen-2026-116420.reviewer_comments.pdf]

PEER REVIEW HISTORY

BMJ Open publishes all reviews undertaken for accepted manuscripts. Reviewers are asked to complete a checklist review form and are provided with free text boxes to elaborate on their assessment. These free text comments are reproduced below.

ARTICLE DETAILS

Title (Provisional)

Potential benefits of music-based therapies on rehabilitation outcomes in people with multiple sclerosis: a systematic review

Authors

Ahadi, Nicky; Kim, Jeeyeon; Whibley, Daniel; Barnish, Maxwell S

VERSION 1 - REVIEW

**Reviewer**	**1**
**Name**	**Yazdan Panah, Mohammad**
**Affiliation**	**Isfahan University of Medical Sciences**
**Date**	**12-Feb-2026**
**COI**	**None**

Dear Editor,

Thank you for the opportunity to review the manuscript entitled “Potential benefits of music-based therapies on rehabilitation outcomes in people with multiple sclerosis: a systematic review.” Although the topic is potentially relevant to the field of neurorehabilitation, I have substantial concerns regarding the methodological rigour, analytical framework, and overall contribution of this review. In its current form, the manuscript does not meet the standards expected for publication in a high-impact general medical journal. My detailed comments are provided below.

1. The manuscript does not demonstrate a sufficiently clear or methodologically robust advancement beyond existing systematic reviews on this topic.

2. The conceptual framing of “music-based therapies” is overly broad and inconsistently applied, resulting in conceptual heterogeneity that undermines the interpretability of the findings.

3. The review lacks analytical depth and remains largely descriptive despite claiming structured narrative synthesis and GRADE-based certainty assessment.

4. The use of CASP checklists as quantitative scoring instruments is inappropriate, as CASP tools are not designed to generate numerical quality scores or percentage-based thresholds.

5. The application of arbitrary percentage cut-offs to classify studies as low, medium, or high-quality lacks methodological justification and introduces subjectivity.

6. The decision to aggregate randomized trials, feasibility trials, quasi-experimental studies, and observational designs within the same synthesis without stratified or sensitivity analyses significantly weakens internal validity.

7. The GRADE assessment is methodologically flawed; starting at “High” certainty for entire outcome domains solely because the majority of studies were labelled RCTs fails to account for small sample sizes, pilot designs, risk of bias, and indirectness.

8. The manuscript does not transparently describe how downgrading decisions were operationalized within GRADE, nor does it provide structured evidence profiles.

9. The approach to evaluating minimally clinically important differences is selective and inconsistently applied, and the choice of “pivotal” outcomes appears post hoc rather than prespecified.

10. Clinical significance is assessed inconsistently across studies, with unclear distinction between within-group change and between-group comparisons.

11. The justification for not performing any quantitative synthesis is insufficient; heterogeneity is asserted rather than formally evaluated.

12. No structured assessment of publication bias or small-study effects is undertaken, despite the predominance of small pilot studies.

13. Dual screening and data extraction were performed on only 50% of records, which is below accepted methodological standards for systematic reviews in major journals.

14. The review does not clearly address potential overlap of study populations across multiple publications.

15. The restriction to English-language studies introduces potential language bias that is neither quantified nor critically examined.

16. The intervention category “music-based therapies” encompasses highly diverse modalities, including rhythmic cueing, motor imagery, receptive listening, and structured therapy, without a clear theoretical or mechanistic framework to justify aggregation.

17. The narrative synthesis does not move beyond listing statistically significant findings and lacks exploration of effect magnitude, consistency patterns, or contextual moderators.

18. Conclusions regarding the relative consistency of motor outcomes appear overstated given that certainty of evidence is rated as low across all domains.

19. The manuscript does not adequately distinguish short-term post-intervention outcomes from sustained or clinically meaningful long-term effects.

20. The interpretation occasionally extends beyond the data presented, particularly when drawing parallels to other neurological conditions.

21. The discussion does not critically interrogate the risk that positive findings may be driven by small-sample bias and performance bias inherent in non-blinded interventions.

22. Terminology is inconsistent throughout the manuscript, alternating between "music-based therapy," "music-based therapies," and "music interventions."

27. The manuscript does not convincingly demonstrate that it changes current understanding or clinical practice beyond prior reviews published in 2023–2025.

28. The added value of including two additional recent studies is not sufficient to justify publication in its current form.

29. Methodological weaknesses substantially limit confidence in the conclusions and reduce the manuscript's suitability for a general medical readership.

**Reviewer**	**2**
**Name**	**Meza, Cecilia**
**Affiliation**	**Sunnybrook Health Sciences Centre**
**Date**	**02-Apr-2026**
**COI**	**None**

Thursday, April 2, 2026

The manuscript addresses an important and timely topic and provides a valuable synthesis of the emerging literature on music-based therapies in multiple sclerosis. The scope is appropriately broad, and the integration of motor, cognitive, psychological, and quality of life outcomes represents a notable strength. Overall, the paper has the potential to make a meaningful contribution to the rehabilitation literature. However, several methodological and reporting issues require clarification and, in some cases, revision. My comments are outlined below.

1. There appears to be a discrepancy in the reported sample sizes that requires clarification. The manuscript reports a total of 938 participants, of whom 863 have multiple sclerosis, with 575 allocated to the intervention group and 295 to control conditions. However, the sum of intervention and control participants (575 + 295 = 870) does not align with the reported number of participants with MS (863). This inconsistency raises concerns about data accuracy. Please clarify whether non-MS participants are included in the totals and whether any participants were excluded from group allocation counts.

2. There is a conceptual inconsistency regarding the distinction between music-based therapies (MBT) and music therapy. While the manuscript defines MBT broadly as any music-based intervention and music therapy as interventions delivered by licensed therapists, this

distinction is not consistently maintained throughout the review. Studies labeled as “music therapy” appear to be included without clear justification or classification. I recommend providing a clear operational definition used for study inclusion and explicitly stating how studies categorized as “music therapy” were incorporated under the MBT framework.

3. The approach to risk of bias assessment raises methodological concerns. The manuscript applies CASP checklists with a numeric scoring system (0–2 per item) and percentage-based thresholds to classify study quality. However, CASP tools are not designed to be used as quantitative scoring instruments, and this transformation is not widely validated. This approach may introduce bias and reduce methodological rigor. I recommend either providing a strong justification for this scoring method with appropriate references or revising the analysis to present a qualitative appraisal consistent with standard CASP use.

4. The interpretation of findings occasionally overstates the strength of the evidence. For example, phrases such as “music-based therapies were consistently associated with improvements” may be too strong given the acknowledged heterogeneity, small sample sizes, and overall low certainty of evidence based on GRADE. I suggest adopting more cautious language, such as “evidence suggests potential benefits” or “findings were generally supportive but heterogeneous,” to better align with the underlying evidence.

5. There are minor but important issues related to clarity and accuracy. For example, the term “preceded” should be corrected to “proceeded” in the description of the screening process. Additionally, the manuscript would benefit from careful proofreading to ensure consistency in terminology and formatting.

6. Key methodological details are missing or insufficiently reported. Specifically, while both randomized controlled trials and observational studies are included, the manuscript does not clearly state how many studies fall into each category. Providing a clear breakdown of study designs would improve transparency and aid interpretation.

7. The description of comparator conditions lacks clarity. The term “control conditions” is used broadly but not defined in sufficient detail. It would be helpful to specify whether these include usual care, no intervention, or active comparators, as this has important implications for interpreting the results.

8. There is some redundancy in the manuscript. I believe this may be as mistake, the data availability statement appears to be repeated verbatim, “All data relevant to the study are included in the article” which should be corrected.

Thanks.

VERSION 1 - AUTHOR RESPONSE

Reviewer: 1 Dr. Mohammad Yazdan Panah, Isfahan University of Medical Sciences

Comment 1: Thank you for the opportunity to review the manuscript entitled “Potential benefits of music-based therapies on rehabilitation outcomes in people with multiple sclerosis: a systematic review.” Although the topic is potentially relevant to the field of neurorehabilitation, I have substantial concerns regarding the methodological rigour, analytical framework, and overall contribution of this review. In its current form, the manuscript does not meet the standards expected for publication in a high-impact general medical journal. My detailed comments are provided below.

Response 1: Thank you for your time reviewing our manuscript in detail. We appreciate your helpful and constructive suggestions and have used them to improve our manuscript through this revision to address your concerns.

Thank you for noting that the topic is potentially relevant to the topic of neurorehabilitation. We have improved our reporting in this revision to provide a more thorough and detailed account of our work. We have also revised our work – including the risk of bias assessment – to address your concerns about the methodological rigour and analytical framework of our work.

To address your concerns about the overall contribution of the review, we have added text to the Introduction section demonstrating more clearly and comprehensively the rationale for our work, especially emphasising the added value we offer in the context of the recent related reviews. We note the positive comments of Reviewer #2 regarding the rationale for our work and the unique contribution we make, and we have taken the opportunity in this revision to ensure this is reflected clearly in the manuscript. Please note that the BMJ Open editorial criteria require that the work is methodologically robust, offers an advance (however small) in scientific knowledge, and is well reported, but do not consider the impact of the work.

Comment #2: The manuscript does not demonstrate a sufficiently clear or methodologically robust advancement beyond existing systematic reviews on this topic.

Response #2: By improving our reporting in this revision and making methodological revisions following your helpful suggestions, we now demonstrate that our work is methodologically robust and well reported. Furthermore, we now show more clearly how our review asks a different research question than previous reviews and thereby offers a clear advance in scientific knowledge.

Comment #3: The conceptual framing of “music-based therapies” is overly broad and inconsistently applied, resulting in conceptual heterogeneity that undermines the interpretability of the findings.

Response #3: Thank you for your observation regarding the conceptual framing. Firstly, we have gone through the manuscript to ensure that our concept of “music-based therapies” (MBTs) is applied consistently throughout. We have added text to the Introduction to more clearly introduce and define the concept of MBTs and contrast

them with the narrower concept of ‘music therapy’ and the broader concept of ‘musical care’. Thereby, we demonstrate the value of the concept of MBTs and provide a clear definition based on previous literature that has used this concept. Furthermore, in our revision, we have ensured that we describe the nature of interventions in the included studies precisely within the main text of our manuscript, to ensure that the findings are interpretable and as useful as possible.

Comment #4: The review lacks analytical depth and remains largely descriptive despite claiming structured narrative synthesis and GRADE-based certainty assessment.

Response #4: Thank you for this comment. We agree that the recommended word limit resulted in the results section of the main manuscript reading rather descriptive and superficial with a lot of important detail consigned to the supplemental material. As described earlier in this response, we have extended the length of the manuscript in this revision to address the Reviewers’ and Editor’s comments thoroughly and ensure that reporting of the narrative synthesis is in accordance with the SWiM guidelines. We have revised the manuscript to add more detail to the main manuscript to improve the analytical depth and ensure it does not read as largely descriptive.

Comment #5: The use of CASP checklists as quantitative scoring instruments is inappropriate, as CASP tools are not designed to generate numerical quality scores or percentage-based thresholds.

Response #5: While we initially used a quantitative method for the CASP checklists following a peer-reviewed published method (Badakshan et al, 2025), we have now revised our CASP approach to use the descriptive qualitative approach without numerical scores or percentage-based thresholds following your recommendation. We have also implemented this revision in the detailed tables in the supplemental information.

Comment #6: The application of arbitrary percentage cut-offs to classify studies as low, medium, or high-quality lacks methodological justification and introduces subjectivity.

Response #6: As noted above, we have revised our CASP approach and therefore no longer use these percentage thresholds.

Comment #7: The decision to aggregate randomized trials, feasibility trials, quasi-experimental studies, and observational designs within the same synthesis without stratified or sensitivity analyses significantly weakens internal validity.

Response #7: Thank you for this observation. We have revised the narrative synthesis to offer stratification to strengthen internal validity following your recommendation and ensure compliance with the SWiM reporting guidelines for narrative synthesis.

Comment #8: The GRADE assessment is methodologically flawed; starting at “High” certainty for entire outcome domains solely because the majority of studies were

labelled RCTs fails to account for small sample sizes, pilot designs, risk of bias, and indirectness.

Response #8: We have thoroughly reviewed the GRADE Book (which has been the official publication of the GRADE Working Group since 2024). As the GRADE Book is not yet complete, we have also reviewed the GRADE Handbook (2013) for chapters that have not yet been added to the GRADE Book. We can confirm that our approach to setting the starting GRADE follows official GRADE guidance.

Comment #9: The manuscript does not transparently describe how downgrading decisions were operationalized within GRADE, nor does it provide structured evidence profiles.

Response #9: We have added text to the manuscript to address these specific points following your recommendation.

Comment #10: The approach to evaluating minimally clinically important differences is selective and inconsistently applied, and the choice of "pivotal" outcomes appears post hoc rather than prespecified.

Response #10: We have added text to the Methods section to clarify how we evaluated minimally clinically important differences (MCIDs), to show that the approach was consistently applied, and to clarify that the choice of pivotal outcomes was prespecified, adding key references to support these decisions.

Comment #11: Clinical significance is assessed inconsistently across studies, with unclear distinction between within-group change and between-group comparisons.

Response #11: We have added text to the Methods section to clarify the approach taken to the assessment of clinical significance and ensure that the approach taken was consistent and clearly distinguished between within-group change and between-group comparisons. We have also ensured that this distinction is incorporated in the presentation of the clinical significance results.

Comment #12: The justification for not performing any quantitative synthesis is insufficient; heterogeneity is asserted rather than formally evaluated.

Response #12: We have added text to the Methods section to more clearly justify the rationale for using narrative synthesis and not performing any quantitative synthesis (meta-analysis). We have also added a citation to guidance stating that the decision whether to undertake meta-analysis should be pre-specified based on clear a priori justification and conceptual understanding rather than 'formally evaluated' post-hoc based on the results of the review.

Comment #13: No structured assessment of publication bias or small-study effects is undertaken, despite the predominance of small pilot studies.

Response #13: Thank you for highlighting this issue. Assessment of publication bias and small-study effects is typically considered in the context of meta-analysis, which is not feasible in our review. However, we consider that the points you raised are important. Therefore, we have investigated alternative ways to assess publication bias and small-study effects without meta-analysis. We have now used Effect Direction plots (to assess small-study effects). We have now also assessed potential outcome reporting bias by comparing outcomes in registered or published protocols with outcomes in journal publications. We have added relevant text to the Methods, Results and Discussion sections, as well as the supplemental materials, ensuring we provide clear justification for our approach. It was not feasible to use funnel plots to conduct a formal assessment of publication bias as the data for most outcomes were not amenable to such aggregation – for many of the same reasons as meta-analysis could not be conducted, including differences in sampling frames and differences in how outcomes were assessed. We have added this limitation to the Discussion.

Comment #14: Dual screening and data extraction were performed on only 50% of records, which is below accepted methodological standards for systematic reviews in major journals.

Response #14: While 50% is compliant with guidance for conducting systematic reviews in a streamlined manner to ensure efficient use of resources, we have increased the second review to 100% in this revision to address your concern.

Comment #15: The review does not clearly address potential overlap of study populations across multiple publications.

Response #15: Thank you for this observation. Overlap of study populations is more frequently considered in the context of meta-analysis since it can undermine the validity of statistical pooling. However, we agree that it is also important to consider in the context of narrative synthesis since overlapping study populations can lead to certain findings being overrepresented in the synthesis. Established systematic review guidance (such as the Cochrane Handbook) does not recommend excluding studies from the narrative synthesis based on population overlap. Where papers report the same study, these should be grouped for synthesis. However, no papers in our review do report on the same study. Instead, there are cases of different studies being conducted in the same setting. There was one pair of studies where a small overlap was noted (3% of the sample), but otherwise it did not appear that there were overlapping participants. We have added text to the Discussion section to discuss this issue.

Comment #16: The restriction to English-language studies introduces potential language bias that is neither quantified nor critically examined.

Response #16: No studies were excluded on grounds of language (see the PRISMA flow chart), as there were no eligible studies identified that were not published in English.

There was one article (Naderi et al) that was available in both English and Farsi (Persian). We included it based on the English language version. One of our authors speaks and reads fluent Farsi and has read the Farsi version and can confirm the content of the Farsi version was the same as the English version. The reason for stating English only in the protocol is due to limitations of indexing of non-English language journals in major scholarly databases and limitations in availability of non-English language journals through the subscriptions of English-speaking universities. We have added this information to the discussion.

Comment #17: The intervention category “music-based therapies” encompasses highly diverse modalities, including rhythmic cueing, motor imagery, receptive listening, and structured therapy, without a clear theoretical or mechanistic framework to justify aggregation.

Response #17: Thank you for this observation. It is true that “music-based therapies” (MBTs) is a broad category, which includes a highly diverse range of modalities. It should be noted that Reviewer #2 considered this a particular strength of our work. To address your concern, we have added text to the Introduction section to provide a clear delineation of MBTs in contrast to narrower concepts such as music therapy and to provide a clear theoretical framework to justify aggregation, citing previous work that has used the MBTs concept.

Comment #18: The narrative synthesis does not move beyond listing statistically significant findings and lacks exploration of effect magnitude, consistency patterns, or contextual moderators.

Response #18: We have revised the narrative synthesis to explore the issues you suggest in your comment to address your concern. We have added all the relevant effect sizes to the supplementary material, and provided an exploration of this issue in the main manuscript to the extent that can be done without making the manuscript so long that readability would be affected.

Comment #19: Conclusions regarding the relative consistency of motor outcomes appear overstated given that certainty of evidence is rated as low across all domains.

Response #19: Following your recommendation and the recommendation of Reviewer #2, we have revised our language on these conclusions to be more cautious.

Comment #20: The manuscript does not adequately distinguish short-term post-intervention outcomes from sustained or clinically meaningful long-term effects.

Response #20: Thank you for this suggestion. We have added text to the Discussion to examine this issue.

Comment #21: The interpretation occasionally extends beyond the data presented, particularly when drawing parallels to other neurological conditions.

Response #21: Thank you for pointing this out – we have reviewed our manuscript carefully for this revision to ensure that the interpretation does not extend beyond the data presented.

Comment #22: The discussion does not critically interrogate the risk that positive findings may be driven by small-sample bias and performance bias inherent in non-blinded interventions.

Response #22: Thank you for this helpful suggestion. We have added text to the Discussion section to address this concern, while ensuring that we keep the manuscript length readable.

Comment #23: Terminology is inconsistent throughout the manuscript, alternating between "music-based therapy," "music-based therapies," and "music interventions."

Response #23: Thank you for this helpful observation – we have standardised our terminology to 'music-based therapies', which we abbreviate as MBTs.

Comment #24: The manuscript does not convincingly demonstrate that it changes current understanding or clinical practice beyond prior reviews published in 2023–2025.

Response #24: We have strengthened the presentation of the rationale for our work in the Introduction section. We have added text to the Discussion section to clearly demonstrate how our work offers an advance in scientific understanding of the field beyond the previous reviews published in 2023-2025. Impact of the work on clinical practice is not part of the BMJ Open editorial criteria.

Comment #25: The added value of including two additional recent studies is not sufficient to justify publication in its current form.

Response #25: We thank you for your observation and apologise that you misunderstood due to our poor phrasing. We said that there were two studies that had not featured in any previous systematic review. The value of a systematic review comes through the integration of findings from the set of identified studies. Therefore, what matters is how many of the identified studies have been integrated together in the same previous systematic review, rather than how many have been included separately in any of previous systematic review. No single previous review had included more than 12 of the 23 studies (52%) we identified. Therefore, we double the amount of evidence that has been integrated together compared to any previous systematic review in the field. This demonstrates a clear advance in knowledge. Also, the BMJ Open editorial criteria do not require the advance in knowledge to be large and do not consider the impact of the work. We have revised the text at the start of the Discussion section to focus on the pertinent information and make our contribution to knowledge clearer.

Comment #26: Methodological weaknesses substantially limit confidence in the conclusions and reduce the manuscript's suitability for a general medical readership.

Response #26: We have improved our reporting and made methodological revisions following your helpful comments to address your concerns about the robustness of our work and confidence that readers can have in our conclusions. Broad appeal to a general medical readership is not part of the BMJ Open editorial criteria.

Reviewer: 2 Dr. Cecilia Meza, Sunnybrook Health Sciences Centre

Comment #1: The manuscript addresses an important and timely topic and provides a valuable synthesis of the emerging literature on music-based therapies in multiple sclerosis.

Response #1: Thank you for your positive comments regarding our work, especially how our manuscript addressed an important and timely topic and provides a valuable synthesis of the emerging literature on music-based therapies (MBTs) in MS.

Comment #2: The scope is appropriately broad, and the integration of motor, cognitive, psychological, and quality of life outcomes represents a notable strength.

Response #2: Thank you for your appreciation of the rationale for our work and for noting the strengths of our work, including the appropriately broad scope and the integration of motor, cognitive, psychological, and quality of life outcomes. Indeed, this is a key part of what makes our review different from previous reviews, providing the basis for the unique contribution our work shall make to the literature. In response to Reviewer #1's suggestions, we have added text to the Introduction to make the rationale and unique contribution of our work clearer.

Comment #3: Overall, the paper has the potential to make a meaningful contribution to the rehabilitation literature.

Response #3: Thank you for appreciating our paper's potential to make a meaningful contribution to the rehabilitation literature.

Comment #4: However, several methodological and reporting issues require clarification and, in some cases, revision. My comments are outlined below.

Response #4: Thank you for your careful review of our work. We appreciate that there were some methodological and reporting issues in the original manuscript, and have addressed them in this revision, as detailed in response to your specific comments below.

Comment #5: There appears to be a discrepancy in the reported sample sizes that requires clarification. The manuscript reports a total of 938 participants, of whom 863 have multiple sclerosis, with 575 allocated to the intervention group and 295 to control conditions. However, the sum of intervention and control participants (575 + 295 = 870) does not align with the reported number of participants with MS (863). This inconsistency raises concerns about data accuracy. Please clarify whether non-MS

participants are included in the totals and whether any participants were excluded from group allocation counts.

Response #5: Thank you for highlighting this discrepancy. We have rechecked the extracted data and identified inaccuracies in the previously reported total sample size and group allocations. The corrected total is 955 participants, of whom 880 had MS and 75 did not. Across all studies, 691 participants received MBT and 264 did not receive MBT and were classified as controls. The control group included both participants with MS and, in some studies, participants without MS. Specifically, of the 75 participants without MS, 58 received MBT and 17 did not. We have revised the manuscript to clarify that group allocation was based on receipt of MBT rather than MS status. In supplemental file 6, we have corrected an error in the total sample size for Helminger et al (2025), added information on the per arm sample size for Thaut et al (2014) and clarified that the 'control' groups in Goetschalckx et al (2021), Moumdjian et al (2019), and Naderi et al (2022) were not comparator groups in terms of intervention. They received the same MBTs, the only difference was that they did not have MS. We have also done another check of the accuracy of all data in our review as requested.

Comment #6: There is a conceptual inconsistency regarding the distinction between music-based therapies (MBTs) and music therapy. While the manuscript defines MBTs broadly as any music-based intervention and music therapy as interventions delivered by licensed therapists, this distinction is not consistently maintained throughout the review. Studies labeled as "music therapy" appear to be included without clear justification or classification. I recommend providing a clear operational definition used for study inclusion and explicitly stating how studies categorized as "music therapy" were incorporated under the MBTs framework.

Response #6: Thank you for your observation. It is important that our terminology is clear. Therefore, we have added text to the Introduction to more clearly differentiate music therapy and music-based therapies (MBTs). We have also, following your valuable suggestion, added text to the Methods providing a clear operational definition of MBTs as part of the inclusion criteria. We have also explained how music therapy falls within the scope of MBTs and therefore why music therapy studies are included. As recommended, we have added text to the study profile in the Results section explicitly stating how many music therapy studies were included in our review.

Comment #7: The approach to risk of bias assessment raises methodological concerns. The manuscript applies CASP checklists with a numeric scoring system (0–2 per item) and percentage-based thresholds to classify study quality. However, CASP tools are not designed to be used as quantitative scoring instruments, and this transformation is not widely validated. This approach may introduce bias and reduce methodological rigor. I recommend either providing a strong justification for this scoring method with

appropriate references or revising the analysis to present a qualitative appraisal consistent with standard CASP use.

Response #7: Thank you for your helpful suggestion. Our write-up of the risk of bias in Supplementary file 11 was focused on the qualitative approach to CASP that is more familiar to readers. We have now removed the quantitative CASP scoring from our manuscript.

Comment #8: The interpretation of findings occasionally overstates the strength of the evidence. For example, phrases such as “music-based therapies were consistently associated with improvements” may be too strong given the acknowledged heterogeneity, small sample sizes, and overall low certainty of evidence based on GRADE. I suggest adopting more cautious language, such as “evidence suggests potential benefits” or “findings were generally supportive but heterogeneous,” to better align with the underlying evidence.

Response #8: We do not wish to overstate the strength of the evidence. Therefore, we have revised the phrasing to adopt more cautious language, following your suggestion.

Comment #9: There are minor but important issues related to clarity and accuracy. For example, the term “preceded” should be corrected to “proceeded” in the description of the screening process.

Response #9: We have reviewed the manuscript carefully to address any minor issues, such as the typographical error you mention.

Comment #10: Additionally, the manuscript would benefit from careful proofreading to ensure consistency in terminology and formatting.

Response #10: We have proofread the revision carefully to ensure consistency in terminology and formatting.

Comment #11: Key methodological details are missing or insufficiently reported. Specifically, while both randomized controlled trials and observational studies are included, the manuscript does not clearly state how many studies fall into each category. Providing a clear breakdown of study designs would improve transparency and aid interpretation.

Response #11: As suggested, we have carefully gone through our manuscript to ensure that key methodological details are well reported. Furthermore, we now provide a clear breakdown of how many studies fall into each study design category.

Comment #12: The description of comparator conditions lacks clarity. The term “control conditions” is used broadly but not defined in sufficient detail. It would be helpful to specify whether these include usual care, no intervention, or active comparators, as this has important implications for interpreting the results.

Response #12: Thank you for this useful suggestion. We have revised the Methods section to ensure that we provide clarity on the eligible comparator conditions. We have also revised the study profile in the Results section to provide more detail on the comparators in including studies, since we agree that the comparators in included studies have important implications for interpreting the results. Additionally, we have revised the Discussion section to ensure that this point is considered in the interpretation of our review findings.

Comment #13: There is some redundancy in the manuscript. I believe this may be as mistake, the data availability statement appears to be repeated verbatim, "All data relevant to the study are included in the article" which should be corrected.

Response #13: We have looked through the manuscript, fixed any typographical errors, and removed any redundant or repeated text. This includes removing the verbatim repetition of "All data relevant to the study are included in the article" in the Declarations section that you noted. Anything that is required by the journal style guide has been retained. We have also addressed redundancy of text in the supplemental materials, where some sections repeated what was in the main manuscript or did not make a substantial addition. The supplemental Discussion (Supplemental file 12) largely repeated what was in the Discussion of the main manuscript in a slightly more verbose way, so has been removed. Similarly, Supplemental file 10 has been shortened to focus on risk of bias results and merged with the previous Supplemental file 11 which contained the risk of bias tables. The original Supplemental file 10 largely repeated the study profile from the main manuscript and then presented a more detailed narrative synthesis, which has now been edited and integrated into the main manuscript as requested by the Editor and Reviewer #1. An assessment of reporting bias has been added as Supplemental file 11.

Comment #14: Thanks.

Response #14: Thank you for your time reviewing our manuscript, and your helpful and constructive comments, which have helped improved our manuscript.

VERSION 2 - REVIEW

**Reviewer**	**2**
**Name**	**Meza, Cecilia**
**Affiliation**	**Sunnybrook Health Sciences Centre**
**Date**	**08-Jun-2026**
**COI**	

The manuscript has been considerably strengthened and is now much clearer and more methodologically transparent. I have identified only the following minor editorial corrections:

1. On page 6 of 122, “fatigue’..” should be corrected by removing the additional period.
2. On page 11 of 122, the sentence describing the observational studies appears to be missing a closing parenthesis: “(one observational cross-sectional comparative study, one case-control study, and one matched control study (details in Supplemental file 6).”
3. On page 11 of 122, “Baseline disability levels were most reported…” would read more clearly as “Baseline disability levels were most commonly reported…”
4. On page 13 of 122, a full stop should be added after the sentence ending in “overall QoL.”
5. On page 14 of 122, “The effect direction plot … show” should be changed to “The effect direction plot … shows.”
6. On page 15 of 122, “using much larger evidence base” should be revised to “using a much larger evidence base.”
7. On page 16 of 122, “The final search conducted in July 2025” should be changed to “The final search was conducted in July 2025.”
8. On page 16 of 122, “Participant characteristic such as race and ethnicity…” should be corrected to “Participant characteristics such as race and ethnicity…”

The authors may also wish to clarify the statement that the July 2025 search was conducted within six months of manuscript submission in January 2026, as the revised proof lists a submission date of 5 June 2026. If January 2026 refers to the original submission date, this should be stated explicitly.

These are minor matters that do not affect the scientific content or conclusions of the review. I recommend acceptance following minor revision.

VERSION 2 - AUTHOR RESPONSE

Reviewer: Cecilia Meza

Comment 1: The authors have made substantial revisions and have satisfactorily addressed the concerns raised in my previous review. In particular, they have clarified the participant totals, provided a clearer operational definition of music-based therapies, revised the risk-of-bias assessment, added a detailed breakdown of study designs and comparator conditions, and adopted more cautious language when interpreting the findings.

Response 1: Thank you for your positive comment

Comment 2: The manuscript has been considerably strengthened and is now much clearer and more methodologically transparent. I have identified only the following minor editorial corrections:

1. On page 6 of 122, “fatigue’..” should be corrected by removing the additional period.
2. On page 11 of 122, the sentence describing the observational studies appears to be missing a closing parenthesis: “(one observational cross-sectional comparative study, one case-control study, and one matched control study (details in Supplemental file 6).”
3. On page 11 of 122, “Baseline disability levels were most reported…” would read more

clearly as “Baseline disability levels were most commonly reported…”

4. On page 13 of 122, a full stop should be added after the sentence ending in “overall QoL.”
5. On page 14 of 122, “The effect direction plot … show” should be changed to “The effect direction plot … shows.”
6. On page 15 of 122, “using much larger evidence base” should be revised to “using a much larger evidence base.”
7. On page 16 of 122, “The final search conducted in July 2025” should be changed to “The final search was conducted in July 2025.”
8. On page 16 of 122, “Participant characteristic such as race and ethnicity…” should be corrected to “Participant characteristics such as race and ethnicity…”

Response 2: Thank you for your positive comment – we have made all these corrections as requested. In correction 2, rather than adding another closing parenthesis, we removed an erroneous parenthesis and replaced it with a comma.

Comment 3: The authors may also wish to clarify the statement that the July 2025 search was conducted within six months of manuscript submission in January 2026, as the revised proof lists a submission date of 5 June 2026. If January 2026 refers to the original submission date, this should be stated explicitly.

Response 3: We have added the word ‘original’ to clarify as requested.

Comment 4: These are minor matters that do not affect the scientific content or conclusions of the review. I recommend acceptance following minor revision.

Response 4: Thank you for your positive comment
