# Supplementary material for: Potential benefits of music-based therapies on rehabilitation outcomes in people with multiple sclerosis: a systematic review
Source: BMJ Open. 2026 Jul 22;16(7):e116420. doi: 10.1136/bmjopen-2026-116420 (PMC13410710; doi:10.1136/bmjopen-2026-116420)
Supplement: Author's manuscript [file bmjopen-2026-116420.draft_revisions.pdf]

BMJ Open is committed to open peer review. As part of this commitment we make the peer review history of every article we publish publicly available.

When an article is published we post the peer reviewers' comments and the authors' responses online. We also post the versions of the paper that were used during peer review. These are the versions that the peer review comments apply to.

The versions of the paper that follow are the versions that were submitted during the peer review process. They are not the versions of record or the final published versions. They should not be cited or distributed as the published version of this manuscript.

BMJ Open is an open access journal and the full, final, typeset and author-corrected version of record of the manuscript is available on our site with no access controls, subscription charges or pay-per-view fees (http://bmjopen.bmj.com).

If you have any questions on BMJ Open's open peer review process please email info.bmjopen@bmj.com

BMJ Open

Potential benefits of music-based therapies on rehabilitation outcomes in people with multiple sclerosis: a systematic review

Journal:	*BMJ Open*
Manuscript ID	bmjopen-2026-116420
Article Type:	Original research
Date Submitted by the Author:	06-Jan-2026

Complete List of Authors:	Ahadi, Nicky ; University of Exeter, University of Exeter Medical School<br>Kim, Jeeyeon ; University of Michigan, Department of Physical Medicine and Rehabilitation<br>Barnish, Maxwell; University of Exeter, School of Public Health and Sport Sciences Medical School<br>Whibley, Daniel; University of Aberdeen, Epidemiology Group; University of Michigan,

Keywords:	Multiple sclerosis < NEUROLOGY, COMPLEMENTARY MEDICINE, PUBLIC HEALTH

SCHOLARONE MANUSCRIPTS

I, the Submitting Author has the right to grant and does grant on behalf of all authors of the Work (as defined in the below author licence), an exclusive licence and/or a non-exclusive licence for contributions from authors who are: i) UK Crown employees; ii) where BMJ has agreed a CC-BY licence shall apply, and/or iii) in accordance with the terms applicable for US Federal Government officers or employees acting as part of their official duties; on a worldwide, perpetual, irrevocable, royalty-free basis to BMJ Publishing Group Ltd ("BMJ") its licensees and where the relevant Journal is co-owned by BMJ to the co-owners of the Journal, to publish the Work in this journal and any other BMJ products and to exploit all rights, as set out in our licence.

The Submitting Author accepts and understands that any supply made under these terms is made by BMJ to the Submitting Author unless you are acting as an employee on behalf of your employer or a postgraduate student of an affiliated institution which is paying any applicable article publishing charge ("APC") for Open Access articles. Where the Submitting Author wishes to make the Work available on an Open Access basis (and intends to pay the relevant APC), the terms of reuse of such Open Access shall be governed by a Creative Commons licence – details of these licences and which Creative Commons licence will apply to this Work are set out in our licence referred to above.

Other than as permitted in any relevant BMJ Author's Self Archiving Policies, I confirm this Work has not been accepted for publication elsewhere, is not being considered for publication elsewhere and does not duplicate material already published. I confirm all authors consent to publication of this Work and authorise the granting of this licence.

For peer review only

Potential benefits of music-based therapies on rehabilitation outcomes in people with multiple sclerosis: a systematic review

Nicky Ahadi[1], Jeeyeon Kim[2], Daniel Whibley[2,3], Maxwell S. Barnish[4]

1 University of Exeter Medical School, University of Exeter, Exeter, UK

2 Department of Physical Medicine and Rehabilitation, University of Michigan, Ann Arbor, USA

3 Center for History, Humanities, Arts, Social Sciences, and Ethics in Medicine, University of Michigan, Ann Arbor, USA

4 Peninsula Technology Assessment Group (PenTAG), School of Public Health and Sport Sciences, University of Exeter Medical School, University of Exeter, Exeter, UK

Correspondence to: Dr Maxwell S. Barnish; m.s.barnish@exeter.ac.uk

Keywords: Multiple sclerosis; Music therapy; Music-based therapy

3960 words

For peer review only

ABSTRACT

Objectives. To assess the evidence for a benefit of music-based therapies on rehabilitation outcomes in adults with multiple sclerosis (MS).

Setting. Scholarly literature (published in English) from any country. This systematic review received no specific funding and was registered on PROSPERO as CRD420251083656.

Data sources. Six bibliographic databases: AMED (Ebsco), APA PsycINFO (Ovid), CINAHL (Ebsco), Cochrane CENTRAL, EMBASE (Ovid) and MEDLINE (Ovid), plus supplementary searches and citation chasing (search date July 2025).

Primary and secondary outcome measures. Eligible studies used a clinical trial or controlled quantitative observational study design to assess the benefit of music-based therapies (MBTs) on rehabilitation outcomes in adults with MS. Risk of bias was assessed using CASP checklists. Data were synthesised using narrative synthesis.

Results. 23 studies were included (938 participants, of whom 863 people had MS and 575 received MBTs). The most frequently assessed outcome was motor function (16 studies), followed by quality of life (12 studies). Music-based therapies, particularly rhythmic auditory stimulation and music-cued motor imagery, were consistently associated with improvements in gait and mobility outcomes, including walking speed, walking distance, cadence, stride length, and gait symmetry; findings for other outcomes were less consistent.

Conclusions. Music-based therapies may benefit people with MS, with the most consistent evidence observed for gait and mobility outcomes. However, heterogeneity in intervention type, study design, and outcome measurement limits the strength of conclusions. Higher-quality, adequately powered trials with standardised outcomes and direct comparisons between music-based intervention approaches are needed to clarify effectiveness and inform clinical implementation.

STRENGTHS AND LIMITATIONS OF THIS STUDY

- Systematic review methods minimised subjectivity and bias.
- A structured outcome set was used.
- An independent dual review was conducted.
- It was not possible to conduct patient and public involvement.
- Only English-language studies could be included.

For peer review only

INTRODUCTION

Multiple sclerosis

Multiple sclerosis (MS) is a chronic inflammatory and neurodegenerative disease of the central nervous system and a leading cause of neurological disability in young adults.[1,2] It affects more than 2.8 million people worldwide and its prevalence has increased substantially in many regions, with the highest rates reported in North America, western Europe and Australasia.[1-3] This rise is attributed to a combination of greater life expectancy, population growth, and better access to diagnostic services.[2,3] Common manifestations include optic neuritis, sensory disturbance, limb weakness and gait ataxia as any site in the central nervous system can be affected.[1-3] MS is usually described as having three main types with Relapsing Remitting MS (RRMS) being the most common, affecting around 85-90% of patients. Primary progressive MS (PPMS) accounts for about 10-15% and progresses steadily without relapses. Over time, many with RRMS develop secondary progressive MS (SPMS), where disability gradually increases.[1]

Treatment options for MS

Disease modifying therapies can reduce relapse frequency and delay disability progression, yet they do not fully address the multifactorial physical, cognitive, and emotional consequences of living with a chronic, unpredictable condition. MS places a heavy burden on health systems and carries high economic costs.[2,3] As symptoms often persist despite medication, there is increasing interest in non-pharmacological approaches to support holistic rehabilitation and improve quality of life (QoL).[3,4] These approaches include arts-based interventions, which have been shown to be promising therapeutic approaches in anxiety and depression[5], palliative care[6], stroke[7] and Parkinson's disease[8-10]. Within this broad arts sphere, one focal point of therapeutic interest is the use of music for therapeutic benefit.

Evidence for Music-Based Therapies in MS

Music-based therapies (MBT) refers to a broad set of interventions and therapeutic approaches using music as a therapeutic medium regardless of the professional background of the therapist or the specific music-based intervention used, contrasting with music therapy, which is a more restricted set of interventions delivered by licensed music therapists, according to the norms and regulations of their health system. We have identified six previous systematic[11-16], scoping or comprehensive reviews on music-based therapies (MBT) for MS, in addition to a broad non-systematic literature review on 'cognitive re-education' for QoL and psychological wellbeing in MS.[17] Table 1 summarises the scope, methodological approaches, interventions, outcomes, and included studies of these reviews. Due to clinical, methodological and statistical heterogeneity between studies, no reviews have been able to include a meta-analysis. An early comprehensive review[14] assessed music therapy in relation to a non-specified set of outcomes and found very limited evidence, with the only full-text peer-reviewed papers besides case reports or case series being two pilot studies[18,19]. Systematic and scoping reviews have typically been limited to specific outcome domains: i) gait and mobility[12,16] and ii) psychological outcomes.[11] Gardener et al[11] conducted a systematic review music therapy for psychological outcomes in a broader range of conditions and included only one pilot RCT[20] on MS. Tan et al[16] conducted a recent scoping review of 15 studies on music based and rhythmic auditory interventions for walking in MS. They focused exclusively on gait and mobility outcomes and reported high acceptability and consistent short-term improvements in walking speed and quality. Kong et al, 2023[12] similarly undertook a systematic review of 12 clinical trials of music therapy for gait disorders in MS, again concentrating on mobility outcomes and finding that music based rhythmic cueing improved

For peer review only

gait performance. Together, these reviews support the role of music-based approaches for gait rehabilitation but do not explore wider motor, cognitive, or psychosocial domains. Lopes et al, 2021[13] provided the first MS specific systematic review of MBT as a rehabilitative approach. Across 10 clinical trials, they grouped interventions into rhythmic auditory strategies, instrument playing, dance-based strategies and neurological music therapy. They found that MBT was superior to conventional therapy or no intervention for several motor and non-motor outcomes, including gait, fatigue, balance, hand dexterity, emotional status and pain, although evidence for memory and mental fatigability was less clear. The search period ended in 2020 and interventions and outcome measures were highly heterogeneous, with several protocols primarily dance-based rather than primarily music focused.[13] Phillips et al, 2025[15] extended the scope of the literature by comparing active and receptive music interventions across 15 studies in people with MS, with outcomes including anxiety, depression, QoL, cognition, gait, balance, fatigue, and hand dexterity. Most interventions were receptive, often involving music listening during walking. While the review showed encouraging effects across motor and non-motor domains, its focus on engagement type and the limited number of studies mean it does not fully represent the breadth of MBT for MS rehabilitation.[15]

Aims and rationale

The aim of our work was to provide a systematic review of the evidence for a benefit of MBT on rehabilitation outcomes in adults with multiple sclerosis. In particular, the primary review question was:

- What is the impact of music-based therapies on rehabilitation outcomes in people with MS?

Secondary review questions were:

- What types of music-based interventions have been studied for their rehabilitation potential for people with MS?
- What specific rehabilitation domains (for example motor, cognitive, psychological, and quality of life) show the greatest response to music-based interventions in people with MS?

Although preliminary research suggests that MBT may offer benefits for motor, cognitive and psychosocial outcomes in people with MS, existing evidence syntheses are fragmented and limited in scope. Available reviews either focus narrowly on gait and walking-related outcomes,[12,16] only include a small number of heterogeneous studies,[13,15] or provide findings from multiple neurological conditions without MS-specific conclusions.[11] Reviews of related performing arts modalities, e.g. dance, highlight potential motor and psychological benefits for people with MS, yet these interventions do not isolate the therapeutic contribution of the musical component.[4] Taken together, these gaps mean that the rehabilitative potential of MBT in MS remains unclear. The key rationale for this review is therefore the absence of a contemporary systematic evaluation of MBT across the full range of rehabilitation outcomes relevant to patients with MS. No existing review provides a comprehensive synthesis of the impact of MBT on motor, cognitive, psychological or QoL domains, nor evaluates the diversity of interventions used.

METHODS

Design

For peer review only

A systematic review was conducted following the Preferred Reporting Items for Systematic Reviews and Meta-Analyses (PRISMA) 2020 guidelines.[21] Completed PRISMA and PRISMA for Abstracts checklists are provided (Supplemental files 1 and 2). This systematic review was registered on the PROSPERO database (CRD420251083656). One protocol amendment was made – to use risk of bias tools from the same suite for all study designs.

Data sources

AMED (Ebsco), APA PsycINFO (Ovid), CINAHL (Ebsco), Cochrane-CENTRAL, EMBASE (Ovid) and MEDLINE (Ovid) were searched from inception until July 2025. Supplementary searches were conducted on Google Scholar and through forward and backward citation chasing on studies identified for full-text review up to November 2025. Searches were designed to retrieve articles on MS and MBT (strategies for all databases are shown in online supplemental file 3) and were designed by NA and MSB and conducted by the lead author NA. MSB conducted supplementary searches and citation chasing.

Inclusion criteria

Screening was initially conducted based on the title and abstract. Potentially relevant articles were screened at the full-text stage to determine inclusion (online supplemental file 4) or exclusion (online supplemental file 5) in the systematic review. Screening was initially conducted by NA based on titles and abstracts, followed by full text screening of potentially eligible studies. A second reviewer (MSB) independently screened a random sample of 50% of records to verify consistency, and any disagreements were resolved through discussion. Eligibility criteria are shown in Table 2. Screening was conducted using Rayyan, which uses artificial intelligence, machine learning, and natural language processing to make systematic reviewing more efficient by learning from user decisions to predict article relevance and highlight key PICO terms. All screening was done manually by the two human reviewers.

Data extraction

Information extracted is shown in Table 3. All data extraction processes were conducted by NA, and MSB independently reviewed a random sample of 50% of records to verify consistency. Any disagreements were resolved through discussion. No automation tools were used. The appendix provides additional information on study characteristics (online supplemental file 6), interventions (online supplemental file 7), controls (online supplemental file 8) and narrative results (online supplemental file 9).

Risk of bias assessment

Risk of bias and methodological quality were assessed using the relevant Critical Appraisal Skills Programme (CASP) checklists for randomised controlled trials, cohort studies, case control studies and cross-sectional studies[22]. Each CASP item was scored as 2 for "yes", 1 for "can't tell" and 0 for "no".[23] Because the total possible score differed across study designs (RCT: 26, cohort: 28, case control: 22, cross sectional: 22), quality ratings were assigned using percentage-based thresholds: low quality (<60 % of the maximum score), medium quality (60–79%) and high quality (≥80 %). Given the higher methodological expectations for RCTs, they were classified using study design specific cut-offs as per Badakhshan et al,[23] while percentage-based thresholds were applied to observational designs. Quality appraisal was conducted independently by NA, with MSB reviewing a random sample of 50% of studies to ensure consistency. Disagreements were resolved through discussion. No automation tools were used. The appendix provides additional information on risk of bias checklists used (supplemental file 10).

For peer review only

Narrative synthesis

A thematic narrative synthesis was used to analyse all studies that met the inclusion criteria. Meta-analysis was not appropriate due to substantial methodological heterogeneity across eligible studies, as per previous reviews in this field. Heterogeneity was observed in study design, type and delivery of MBT, intervention duration and intensity, and outcome measures, which prevented meaningful quantitative pooling of results in a meta-analysis. Synthesis was initially organised by four rehabilitation outcome domains: (1) motor outcomes, (2) cognitive outcomes, (3) psychological outcomes and (4) QoL. Within each outcome domain, findings were further synthesised according to the type of MBT used, such as rhythmic auditory stimulation, receptive music listening, active music making and structured music therapy interventions. This approach enabled comparison across rehabilitation domains and facilitated evaluation of how different MBT contribute to rehabilitation outcomes in people with MS.

Clinical significance

We searched for MCIDs for outcome measures for motor, cognitive, psychological, and quality of life outcome domains. Diversity of measures used across studies, which was a factor in precluding meta-analysis, was also a limitation in conducting assessment using MCIDs. Only measures that were considered pivotal by the reviewers and used in at least three studies were included. The MCIDs identified and applied are shown in Table 4.

Certainty assessment

We conducted certainty assessment using GRADE[24] for each outcome domain supported by the narrative synthesis. Implementation in a narrative synthesis context followed Barnish et al.[8] Scoring explanations are provided in Table 5. It was determined that the different forms of MBT in the evidence base did not form sufficiently clear and distinct categories to conduct separate GRADE assessments by type of MBT.

Patient and public involvement

Patient and public involvement could not be conducted for this systematic review assessing a broad range of MBT due to a lack of funding. The corresponding author will respond to any reputable media enquiries.

RESULTS

Search results

Database searches returned a total of 5906 records (AMED 17, PsycINFO/APA PsycINFO 1155, EMBASE 4029 and MEDLINE 198, CINAHL 107, Cochrane central 400), plus 13 from supplementary searches. A total of 4918 records preceded to title and abstract screening (including one duplicate). 47 unique records (including one duplicate) were assessed at full-text screening; 23 studies were included in the systematic review (Figure 1).

Study profile

The included studies comprised a total of 938 participants, of whom 863 had MS and 75 did not. All participants who received MBT had MS. Across the studies, 575 participants received MBT and 295 participants were allocated to control conditions. Sex was incompletely reported; where data were available, 666 participants (75.0%) were female and 222 (25.0%) were male. Reported mean ages across studies generally ranged from the mid 40s to early 50s, with overall participant ages spanning approximately 29 to 68 years. Disease duration was inconsistently reported, but where available typically ranged from approximately 7 to 18 years

For peer review only

since diagnosis, with similar durations between intervention and control groups within individual studies. Baseline disability levels were most reported using the Expanded Disability Status Scale (EDSS). Across studies reporting EDSS, participants generally had mild to moderate disability, with median or mean scores most frequently ranging between 2.0 and 4.5. No studies directly compared different music-based intervention types with each other. MBTs assessed covered both active and passive forms of music therapy (instrumental and vocal) as well as other MBTs such as musically and rhythmically based cueing, and rhythmic auditory stimulation. No studies assessed choral singing or community singing groups. Further details, including on the types of MBTs assessed, are provided in Supplemental file 10.

Narrative synthesis

As 23 studies were included in this systematic review,[18-20,25-44] a summary of the narrative synthesis is presented here, with further details provided in supplementary file 10. An overview of the evidence landscape of the 23 included studies across the four outcome domains is presented in Table 6.

Motor outcomes

Motor outcomes were the most frequently assessed, reported in 16 of the 23 included studies[19,26-29,31-39,41,44] (list in Supplemental file 4). Across these studies, MBTs were consistently associated with improvements in gait and mobility outcomes, including walking speed, walking distance, and spatiotemporal gait parameters.[26-29,31,33-39,41,44] Rhythmic auditory stimulation (RAS) and music cued motor imagery were associated with statistically significant improvements in gait symmetry, cadence, stride length, and walking efficiency in eight controlled trials[26,34-39,41]. Improvements were also observed in balance, coordination, and synchronisation outcomes, with some studies reporting associations between improved motor coordination and cognitive performance or walking capacity.[28,31,33,34] Upper limb function[27] and respiratory muscle strength[19] outcomes were less frequently assessed, with mixed findings.

Cognitive outcomes

Eight studies assessed cognitive outcomes, including global cognitive function, learning and memory, and executive function or attention[20,25,31,32,33,39,42,43](list in Supplemental file 4). MBTs were associated with statistically significant improvements in verbal learning, word order memory, and delayed recall in several studies, particularly when learning was paired with musical structure rather than spoken words.[42,43] However, when MBT was compared to conventional cognitive rehabilitation, no statistically significant between-group differences were observed, suggesting comparable rather than superior effects.[20,31]

Psychological outcomes

Seven studies evaluated psychological outcomes, including symptoms of depression and anxiety, self-acceptance or self-esteem, mood, and emotional processing[18,20,25,30,31,39,40] (list in Supplemental file 4). Several studies reported significant improvements in depression, anxiety, and self-acceptance following MBTs, with some effects persisting at follow-up.[18,25,40] However, between-group differences compared to control conditions were not consistently observed, and findings varied according to study design, comparator, and outcome measure.[18,20,25,31,39]

Quality of life outcomes

QoL-related outcomes were assessed in 12 studies, frequently alongside fatigue and functional disability measures[18,20,25,30,31,35-39,44] (list in Supplemental file 4). Improvements in

For peer review only

QoL and reductions in fatigue were reported in multiple studies, particularly following music cued motor imagery and rhythmic auditory interventions[35-39]. Fatigue was one of the most assessed non motor outcomes, with both physical and cognitive fatigue showing improvement after some interventions, although findings were not uniform across studies.[25,30,33,35-39,44]

Summary of synthesis and risk of bias

Overall, current evidence suggests that MBT may provide benefits across motor, cognitive, psychological, and quality of life domains in people with MS. However, heterogeneity in intervention type, outcome measures, comparator conditions, and study design limits the strength of conclusions that can be drawn, and several outcomes were assessed in only a small number of studies. The risk of bias profile as well as the potential impact of risk of bias on the outcomes of the narrative synthesis are presented in online supplemental file 11.

Clinical significance

Available evidence consistently supported a clinically significant benefit of MBTs on depressive symptoms as assessed by the Beck depression scale. However, less consistent evidence of a clinically significant benefit was found for motor function assessed by the timed 25-foot walk test and quality of life assessed by the MSIS-29 physical subscale. Meanwhile, the only evaluable study for anxiety assessed by HADS-anxiety subscale did not find evidence of a clinically significant benefit. Further details can be found in Table 7.

Certainty assessment

Certainty of evidence was assessed as Low for all outcome domains using the GRADE schemata, reflecting potential issues around heterogeneity and the presence of studies assessed at potential high risk of bias, although most included studies for each outcome domain were RCTs.

DISCUSSION

Summary

This systematic review synthesised evidence on MBT for rehabilitation outcomes in people with MS, identifying 23 eligible studies published between 1999 and 2025, of which two[30,40] had not been included in any previous review on this topic. Furthermore, no single previous review[14,15] had included more than 12 of the 23 studies (52%) we identified. Furthermore, the present review adds an assessment of clinical significance and certainty of findings. Across the 23 studies, outcomes could be mapped to four rehabilitation domains, with motor outcomes most frequently assessed (16 studies), followed by quality of life and related outcomes (12 studies), cognitive outcomes (8 studies), and psychological outcomes (7 studies). Motor findings were most consistent for gait and mobility, particularly where interventions used rhythmic auditory stimulation and music cued motor imagery, with improvements reported in walking speed, walking distance, cadence, stride length, and gait symmetry. Cognitive findings were mixed, with clearer benefits in music assisted learning paradigms (e.g. sung versus spoken word learning), while broader cognitive rehabilitation comparisons suggested similar rather than superior benefits relative to conventional rehabilitation. Psychological outcomes showed potential improvements in depression, anxiety, self-acceptance and self-esteem, but between group effects were not uniform. Quality of life and fatigue were frequently co-assessed, with several trials reporting improvements in fatigue and MS-specific quality of life measures, though findings were heterogeneous. Importantly, this review incorporated studies that have been less emphasised in prior summaries and were necessary to include for a complete synthesis. Challenges were identified in terms of certainty of evidence as assessed by GRADE and reaching clinical significance thresholds across the sample for some outcomes. These may reflect the emerging nature of the evidence base for

For peer review only

MBTs in MS, the arts in health more generally, and the methodological challenges associated therewith.

Interpretation of findings

This review extends and updates the MS rehabilitation literature on MBT by both increasing the quantity of included evidence and the scope of interventions and outcomes compared with prior syntheses. A key interpretive issue is the distinction between active and receptive MBT. The evidence base remains weighted toward receptive approaches, particularly music listening during walking or cueing based strategies. Phillips et al[14] Similarly concluded that most included interventions were receptive, with fewer active interventions, and highlighted gait parameters and fatigue as the most common outcomes. The findings in the present review corroborate that pattern but also suggest that the active versus receptive categorisation may be too simplistic for MS rehabilitation.[15] This review also differs from Phillips et al[14] in scope and included studies. Phillips et al searched from 2002 to June 2024 and included 15 studies.[15] By design, that excludes earlier work such as the 1999 singing and respiratory intervention trial,[19] and it does not capture more recent studies. The inclusion here of Kuhlmann et al[30] provides additional randomised evidence on receptive vibration-based therapy effects on fatigue and pain perception,[30] while Helmlinger et al[29] contributes neuroimaging correlated outcomes that are not represented in reviews with earlier cut-offs. [29] It is notable that, compared with Parkinson's disease, (PD)[8] there is a relative lack of research on singing based interventions in MS. Across outcomes, the pattern of benefit appears strongest and most consistent for gait and mobility, with more heterogeneous findings for cognition and psychological outcomes. This likely reflects differences in intervention intent and dose, diversity in comparators, and inconsistent outcome measures. Overall, MBTs appear promising across multiple rehabilitation domains in MS, but the evidence remains constrained by intervention heterogeneity, limited head-to-head comparisons of MBT types, and variable methodological reporting, which together limit the certainty of any single conclusion.

Strengths and limitations

This systematic review has several strengths. A comprehensive and transparent search strategy was employed across multiple databases, with the final search conducted in July 2025, within six months of manuscript submission, in line with best practice guidance for systematic reviews, which states that searches should be within 12 months of the date of submission and ideally six months.[45] The review adhered to PRISMA[21] reporting standards and applied predefined eligibility criteria, reducing the risk of selection bias. Data extraction was undertaken using standardised forms, which helped ensure consistency in capturing study characteristics, intervention details, and outcomes across heterogeneous study designs. A key methodological strength is the exclusive use of CASP risk of bias tools,[22] selected according to study design. All assessments were drawn from the same CASP suite, ensuring conceptual consistency across randomised controlled trials, cohort studies, case-control studies, and cross-sectional studies. This approach avoided methodological fragmentation that can arise when multiple unrelated appraisal tools are combined. Where inconsistencies existed in how previous systematic reviews labelled study designs, studies were appraised based on their actual methodological features rather than historical labels, ensuring the most appropriate CASP checklist[22] was applied. This strengthened the internal coherence of the risk of bias assessment and improved transparency. However, limitations should be acknowledged. Although many included studies were randomised, blinding of participants and intervention providers was not possible due to the nature of MBTs, increasing the potential for performance bias. Sample sizes were frequently small, and several studies were exploratory or pilot in nature, limiting the statistical power. Reporting of participant flow, allocation concealment, and precision estimates was inconsistent, particularly in older studies and non-randomised designs. Furthermore, race and ethnicity of participants is not consistently reported – more clearly profiled and diverse samples could be beneficial in future studies.

For peer review only

Implications for research and practice

Future research should prioritise addressing the methodological limitations identified in this review. Larger, adequately powered randomised controlled trials are needed, with clearer reporting[46] of allocation concealment, participant flow, and effect size precision. Greater standardisation of outcome measures, particularly for gait, fatigue, cognition, and quality of life, would enhance comparability and enable future quantitative synthesis. Reporting of clinically meaningful change, alongside statistical significance, should be prioritised to support translation into practice. There is a clear need for studies that directly compare different MBTs, including active versus receptive therapies. The relative lack of research on singing-based interventions in MS represents a notable gap, particularly given the evidence base for therapeutic singing in Parkinson's disease.[8] Future studies should also expand beyond gait-focused outcomes to include communication, social participation, and patient-prioritised outcomes. From a clinical perspective, the current evidence base supports cautious integration of MBTs, particularly rhythmic cueing and music-cued motor imagery, within MS rehabilitation programmes. These interventions appear feasible in both facility and home-based settings and may complement conventional physiotherapy and cognitive rehabilitation. However, given heterogeneity and residual risk of bias, MBTs should currently be considered an adjunct rather than a replacement for established rehabilitation approaches.

Conclusion

This systematic review synthesised evidence from 23 studies examining the effects of music-based therapy on motor, cognitive, psychological, and quality of life outcomes in people with multiple sclerosis. The narrative synthesis suggests that music-based interventions, particularly rhythmic auditory stimulation and music-cued motor imagery, may provide benefits for gait and mobility, with more variable evidence for cognitive, psychological, and quality of life outcomes. Evidence for cognitive benefit was strongest in music-assisted learning paradigms, while psychological and fatigue-related outcomes showed mixed but promising signals.

This review extends prior rehabilitation-focused syntheses by including a broader range of intervention types, outcome domains, and recent studies, while maintaining methodological consistency through exclusive use of CASP risk of bias tools. Nonetheless, heterogeneity in interventions, outcomes, and study design limits the strength of conclusions. Overall, MBTs appear to be a promising adjunct in MS rehabilitation, but higher-quality, standardised research is required to clarify optimal intervention models and establish clinical effectiveness. Additional discussion can be found in Supplemental file 12.

DECLARATIONS

Contributors. MSB provided some initial suggestions of topic areas in which he would be willing to supervise a student project on the Royal Society for Medicine systematic review programme for medical students. NA suggested a specific topic for this review which, through discussion, NA and MSB refined to form the protocol for this systematic review. NA designed the database searches, refined them through discussion with MSB, and ran these searches. MSB designed and conducted the supplementary searches and citation chasing. Screening, data extraction and risk of bias assessment were conducted by NA and MSB. NA and MSB conducted the narrative synthesis and interpreted the findings. MSB conducted the GRADE analysis. NA wrote the first draft of the manuscript, which was initially revised by MSB prior to circulation to all authors. MSB wrote the supplemental materials. DW and JK provided valuable contributions to interpreting the findings and revised the manuscript for substantive intellectual content. All authors read the final version of the manuscript prior to submission and take responsibility for their own work as appropriate. NA is a medical

For peer review only

student and her work on this project was supervised by MSB (Senior Research Fellow). JK is a postdoctoral fellow and her work on this project was supervised by DW (Assistant Professor and centre Co-Director). NA was the project manager and MSB was the project director. MSB is the guarantor and takes overall responsibility for the work.

Acknowledgements. This work was done under the auspices of the Royal Society of Medicine systematic review programme for medical students. NA was a co-ordinator of the programme as well as a participant. MSB supervised NA on this programme. The programme did not provide any specific funding for this work. DW is Assistant Professor in Physical Medicine and Rehabilitation and Co-Director (Arts & Medicine), Center for History, Humanities, Arts, Social Sciences & Ethics in Medicine, University of Michigan, Ann Arbor, USA. MSB previously received expenses but not payment for arts promotion activities, however these ceased in 2010 and consequently no longer represent a competing interest.

Funding. The authors have not declared a specific grant for this research from any funding agency in the public, commercial or not-for-profit sectors.

Competing interests. The authors declare no competing interests.

Patient and public involvement. Patients and/or the public were not involved in the design, or conduct, or reporting, or dissemination plans of this research.

Patient consent for publication. Not applicable.

Ethics approval. Not applicable.

Conference presentation. This work has not been published or submitted to any other journal. A conference abstract has been accepted an oral presentation at the 2nd International Meeting of Arts Prescribing in HealthCare, Olympus Riviera, Greece, April 2026.

Data availability statement. All data relevant to the study are included in the article or uploaded as supplementary information. All data relevant to the study are included in the article or uploaded as online supplemental material. The presented work is a systematic review. All relevant information is provided in the manuscript and appendices. This includes the data extraction form completed with the data from all included studies. No meta-analyses were conducted, so there is no analytical code.

REFERENCES

1. Portaccio E, Magyari M, Havrdova EK, et al. Multiple sclerosis: emerging epidemiological trends and redefining the clinical course. *Lancet Reg Health Eur;* 2024;44: 100977.
2. GBD 2016 Multiple Sclerosis Collaborators. Global, regional, and national burden of multiple sclerosis 1990–2016: a systematic analysis for the Global Burden of Disease Study 2016. *Lancet Neurol* 2019;18(3):269-85.
3. Jakimovski D, Bittner S, Zivadinov R, et al. Multiple sclerosis. *Lancet* 2023;403 (10422): 183-202.
4. Davis E, Webster A, Whiteside B, et al. Dance for multiple sclerosis: a systematic review. *Int J MS Care* 2023; 25(4): 176-85.
5. Barnish MS, Nelson-Horne B. Group-based active artistic interventions for adults with primary anxiety and depression: a systematic review. *BMJ Open* 2023;13(6):e069310.
6. Pérez-Eizaguirre M, Vergara-Moragues E. Music therapy interventions in palliative care: a systematic review. *J Palliat Care* 2021;36(3):194–205.
7. Liu Q, Li W, Yin Y, et al. The effect of music therapy on language recovery in patients with aphasia after stroke: a systematic review and meta-analysis. *Neurol Sci* 2022;42(3):863–72.

For peer review only

8. Barnish MS, Reynolds SE, Nelson-Horne RV. Active group-based performing arts interventions in Parkinson's disease: an updated systematic review and meta-analysis. *BMJ Open* 2025;15(4):e089920.
9. Barnish MS, Barran SM. A systematic review of active group-based dance, singing, music therapy and theatrical interventions for quality of life, functional communication, speech, motor function and cognitive status in Parkinson's disease. *BMC Neurol* 2020;20(1):371.
10. Barnish J, Atkinson RAA, Barran SM, et al. Potential benefit of singing for people with Parkinson's disease: a systematic review. *J Parkinsons Dis* 2016;6(3):473–84.
11. Gardener SH, Mukaetova-Ladinska EB, Perera NA. The effect of music therapy on psychological outcomes for neurological conditions: a systematic review. *Medicina* 2025;61(9):1611.
12. Kong L, Zhang X, Meng L, et al. Effects of music therapy intervention on gait disorders in persons with multiple sclerosis: a systematic review of clinical trials. *Mult Scler Relat Disord* 2023;73:104629.
13. Lopes J, Keppers II. Music-based therapy in rehabilitation of people with multiple sclerosis: a systematic review of clinical trials. *Arq Neuropsiquiatr* 2021;79(6):527–35.
14. Ostermann T, Schmid W. Music therapy in the treatment of multiple sclerosis: a comprehensive literature review. *Expert Rev Neurother* 2006;6(4):469–77.
15. Phillips CS, Kim J, Ganesh A, et al. Impact of active versus receptive music interventions on psychosocial and neurological outcomes in people with multiple sclerosis: a systematic review. *J Integr Complement Med* 2025;31(10):889–903.
16. Tan Y, Chen X, Wang L, et al. Music interventions for gait and mobility in multiple sclerosis: a scoping review. *Mult Scler Relat Disord* 2025;101:106563.
17. Manocchio L, Moriano C, D'Amato A, et al. Beyond cognition: cognitive re-education and psychological wellbeing in people with multiple sclerosis: a narrative review. *NeuroSci* 2025; 6(3): 45.
18. Schmid W, Aldridge D. Active music therapy in the treatment of multiple sclerosis patients: a matched control study. *J Music Ther* 2004;41(3):225–40.
19. Wiens ME, Reimer MA, Guyn HL. Music therapy as a treatment method for improving respiratory muscle strength in patients with advanced multiple sclerosis: a pilot study. *Rehabil Nurs* 1999;24(2):74–80.
20. Impellizzeri F, Leonardi S, Latella D, et al. An integrative cognitive rehabilitation using neurologic music therapy in multiple sclerosis: a pilot study. *Medicine (Baltimore)* 2020;99(4):e18866.
21. Page MJ, McKenzie JE, Bossuyt PM, et al. The PRISMA 2020 statement: an updated guideline for reporting systematic reviews. *BMJ* 2021;372:n71.
22. Critical Appraisal Skills Programme. CASP checklists [online]. https://casp-uk.net/casp-tools-checklists/ (accessed 12 December 2025).
23. Badakhshan U, Zamani R, Martins T. Exploring ethnic disparities in burn injury outcomes in the UK: a systematic review. *Eur Burn J* 2025;6(3):48.
24. Prasad M. Introduction to the GRADE tool for rating certainty in evidence and recommendations. *Clin Epidemiol Glob Health* 2024;25:101484.
25. Aldridge D, Schmid W, Kaeder M, et al. Functionality or aesthetics? A pilot study of music therapy in the treatment of multiple sclerosis patients. *Complement Ther Med* 2005;13(1):25–33.
26. Conklyn D, Stough D, Novak E, et al. A home-based walking program using rhythmic auditory stimulation improves gait performance in patients with multiple sclerosis: a pilot study. *Neurorehabil Neural Repair* 2010;24(9):835–42.
27. Gatti R, Tettamanti A, Lambiase S, et al. Improving hand functional use in subjects with multiple sclerosis using a musical keyboard: a randomized controlled trial. *Physiother Res Int* 2015;20(2):100–7.
28. Goetschalckx M, Van Geel F, Meesen R, et al. Rhythmic interlimb coordination of the lower limbs in multiple sclerosis during auditory pacing to three different frequencies. *Gait Posture* 2021;86:334–40.

For peer review only

29. Helmlinger B, Seebacher B, Ropele S, et al. Effects of rhythmic-cued gait training on gait-like task related brain activation in people with multiple sclerosis. *J Neurol Sci* 2025;471:123426.
30. Kuhlmann J, Ebner K, Zimmer A, et al. Music therapy with a monochord in multiple sclerosis (“MUTIMS”): a randomized, controlled, rater-blinded trial. *Mult Scler J Exp Transl Clin* 2025;11(3):20552173251352712.
31. Maggio MG, Tripoli D, Porcari B, et al. How may patients with MS benefit from using music assisted therapy? A case-control feasibility study investigating motor outcomes and beyond. *Mult Scler Relat Disord* 2021;48:102713.
32. Moore KS, Peterson DA, O’Shea G, et al. The effectiveness of music as a mnemonic device on recognition memory for people with multiple sclerosis. *J Music Ther* 2008;45(3):307–29.
33. Moumdjian L, Moens B, Maes PJ, et al. Continuous 12 min walking to music, metronomes and in silence: auditory-motor coupling and its effects on perceived fatigue, motivation and gait in persons with multiple sclerosis. *Mult Scler Relat Disord* 2019;35:92–9.
34. Naderi S, Sadeghi H, Amirseyfaddini M. The effects of rhythmic auditory stimulation with different tempos on spatio-temporal parameters and gait symmetry in patients with multiple sclerosis: a quasi-experimental study. *J Res Rehabil Sci* 2022;18(1): 92-9.
35. Seebacher B, Kuisma R, Glynn A, et al. Rhythmic cued motor imagery and walking in people with multiple sclerosis: a randomized controlled feasibility study. *Pilot Feasibility Stud* 2015;1:25.
36. Seebacher B, Kuisma R, Glynn A, et al. The effect of rhythmic-cued motor imagery on walking, fatigue and quality of life in people with multiple sclerosis: a randomized controlled trial. *Mult Scler J* 2017;23(2):286–96.
37. Seebacher B, Kuisma R, Glynn A, et al. Exploring cued and non-cued motor imagery interventions in people with multiple sclerosis: a randomized feasibility trial and reliability study. *Arch Physiother* 2018;8:6.
38. Seebacher B, Kuisma R, Glynn A, et al. Effects and mechanisms of differently cued and non-cued motor imagery in people with multiple sclerosis: a randomised controlled trial. Mult Scler J 2019; 25(12): 1593-1604.
39. Seebacher B, Helmlinger B, Pinter D, et al. Actual and imagined music-cued gait training in people with multiple sclerosis: a double-blind randomized parallel multicenter trial. *Neurorehabil Neural Repair* 2024;38(8):555–69.
40. Seighalani MZ, Ghahari S, Zarbakhsh M. The effectiveness of music therapy on depression, anxiety and self-esteem of patients with multiple sclerosis. *Adv Environ Biol* 2014;8(9):253–60.
41. Shahraki M, Sohrabi M, Taheri Torbati HR, et al. Effect of rhythmic auditory stimulation on gait kinematic parameters of patients with multiple sclerosis. *J Med Life* 2017;10(1):33–7.
42. Thaut MH, Peterson DA, Sena KM, et al. Musical structure facilitates verbal learning in multiple sclerosis. *Music Percept* 2008;25(4):325–30.
43. Thaut MH, Peterson DA, McIntosh GC, et al. Music mnemonics aid verbal memory and induce learning-related brain plasticity in multiple sclerosis. *Front Hum Neurosci* 2014;8:395.
44. Young HJ, Mehta TS, Herman C, et al. The effects of music to movement and adapted yoga on physical and psychosocial outcomes in people with multiple sclerosis. *Arch Phys Med Rehabil* 2019;100(3):391–400.
45. Higgins JPT, Thomas J, Chandler J, et al (Eds). *Cochrane Handbook for Systematic Reviews of Interventions* version 6.5 (updated August 2024). Available at:// www.cochrane.org/handbook [Last accessed 6 January 2026].
46. Robb SL, Story KM, Harman E, et al. Reporting Guidelines for Music-based Interventions checklist: explanation and elaboration guide. Front Psychol 2025;16:1552659.

For peer review only

47. Basturk S, Ekici G, Kirteke F, et al. Therapeutic effects of line dancing in people with multiple sclerosis: an evaluator-blinded randomized controlled study. *Arts Health* 2024; doi: 10.1080/17533015.2024.2325425 [Online ahead of print].
48. Ng A, Bunyan S, Suh J, et al. Ballroom dance for persons with multiple sclerosis: a pilot feasibility study. *Disabil Rehabil* 2020;42(8):1115–21.
49. Van Geel F, Van Asch P, Veldkamp R, et al. Effects of a 10-week multimodal dance and art intervention program leading to a public performance in persons with multiple sclerosis: a controlled pilot trial. *Mult Scler Relat Disord* 2020;44:102256.
50. Baram Y, Miller A. Auditory feedback control for improvement of gait in patients with multiple sclerosis. *J Neurol Sci* 2007;254 (1-2):90–4.
51. Moumdjian L, Maes PJ, Dalla Bella S, et al. Detrended fluctuation analysis of gait dynamics when entraining to music and metronomes at different tempi in persons with multiple sclerosis. *Sci Rep* 2020;10(1):12934.
52. Moumdjian L, Moens B, Maes PJ, et al. Walking to music and metronome at various tempi in persons with multiple sclerosis: a basis for rehabilitation. *Neurorehabil Neural Repair* 2019;33(6):464–75.
53. Coleman CI, Sobieraj DM, Marinucci LN. Minimally important clinical difference of the Timed 25-Foot Walk Test: results from a randomized controlled trial in patients with multiple sclerosis. *Curr Med Res Opin*. 2012;28(1):49-56.
54. Button KS, Kounali D, Thomas L, et al. Minimal clinically important difference on the Beck Depression Inventory--II according to the patient's perspective. *Psychol Med*. 2015;45(15):3269-79.
55. Lemay KR, Tulloch HE, Pipe AL, et al. Establishing the minimal clinically important difference for the Hospital Anxiety and Depression Scale in patients with cardiovascular disease. *J Cardiopulm Rehabil Prev* 2019; 39(6): E6-11.
56. Puhan MA, Frey M, Buchi S, et al. The minimal important difference of the hospital anxiety and depression scale in patients with chronic obstructive pulmonary disease. *Health Qual Life Outcomes* 2008: 2(6):46.
57. Canadian Agency for Drugs and Technologies in Health. *Clinical review report: Daclizumab (Zinbryta*). Ottawa (ON): CADTH; 2017.
58. Costelloe L, O'Rourke K, Kearney H, et al. The patient knows best: significant change in the physical component of the Multiple Sclerosis Impact Scale (MSIS-29 physical). *J Neurol Neurosurg Psychiatry* 2007;78(8):841-4

For peer review only

TABLES AND FIGURES

Table 1. Previous systematic, scoping and comprehensive reviews

Authors	Method	Search date	PROSPERO	Interventions	Outcomes	Included studies
Gardener et al, 2025[11]	Systematic review (without meta-analysis)	January 2025 (limited to literature since 2015)	Not registered, no pre-published protocol	Music therapy	Psychological outcomes	Wider range of neurological conditions including MS. For MS:<br>1 pilot RCT (Impellizeri et al, 2020)[20].
Kong et al, 2023[12]	Systematic review (without meta-analysis)	October 2022	CRD 42022365668	Music therapy	Gait and mobility outcomes	2 case series. 7 RCTs (Conklyn et al, 2010[26]; Seebacher et al, 2015[35]; Seebacher et al, 2017[36]; Shahraki et al, 2017[41]; Seebacher et al, 2018[37]; Seebacher et al, 2019[38]; Young et al, 2019[44]).<br>3 non-RCTs (Van Geel et al, 2020[49]; Maggio et al, 2021[31]; Ng et al, 2020[48]).
Lopes et al, 2021[13]	Systematic review (without meta-analysis)	July 2020	CRD 42020143080	Music-based therapy	Balance, cognition, dexterity, fatigue, gait, pain, QoL	10 clinical trials (Aldridge et al[25], 2005; Conklyn et al, 2010[26]; Gatti et al, 2015[27]; Van Geel et al, 2020[49]; Impellizeri et al, 2020[20]; Moore et al, 2008[32]; Seebacher et al, 2019[38]; Seebacher et al, 2017[36]; Thaut et al, 2014[43]*; Young et al, 2019[44]).

For peer review only

Ostermann et al, 2006[14]	Comprehensive review	Not stated	Not registered (review pre-dates PROSPERO)	Music therapy	Not specified	8 conference abstracts, chapters or non-peer-reviewed sources.<br>2 case reports or case series.<br>2 pilot studies (Schmid et al 2004[18]; Wiens et al, 1999[19]).
Phillips et al, 2025[14]	Systematic review (without meta-analysis)	June 2024	CRD 42022338291	Music interventions (active or receptive)	Anxiety, balance, cognition, depression, dexterity, fatigue, gait, QoL	10 RCTs (Basturk et al, 2024[47]; Conklyn et al, 2010[26]; Gatti et al, 2015[27]; Impellizzeri et al, 2020[20]; Moore et al, 2008[32]; Seebacher et al, 2017[36]; Seebacher et al, 2019[38]; Thaut et al, 2008[42]; Thaut et al, 2014[43]; Young et al, 2019[44]).<br>5 quasi-experimental studies (Maggio et al, 2021[31]; Moumdjian et al, 2019[33]; Schmid et al, 2004[18]; Van Geel et al, 2020[49]).
Tan et al, 2025[15]	Scoping review	April 2025	Not registered (but protocol uploaded to Open Science Framework)	Music interventions	Gait and mobility	8 RCTs (Baram & Miller, 2007[50]; Conklyn et al, 2010[26]; Helmlinger et al, 2025[29]; Seebacher et al, 2024[39], 2019[38], 2018[37], 2017[36], 2015[34]; Shahraki et al, 2017[41])**. 3 non-randomised experimental studies (Maggio et al, 2021[31]; Moumdjian et al, 2020[51]; Naderi et al, 2022[34]).<br>One cross-sectional study (Goetschalckx et al, 2021[28]).<br>Two case-control studies

For peer review only

						(Moumdjian et al, 2019a[33],b[51]) Two case studies.

* The citation for Thaut et al (2014)[43] was incorrect in the bibliography of the cited review but appears to refer to this study. ** The review states 8 RCTs but provides 9 citations – it would appear that 2 papers are being referred to as parts of the same study, but this is not made clear. All included studies from these previous reviews were included in our review with the following exceptions: i) case studies, case reports, case series, conference abstracts, chapters and non-peer-reviewed sources were not eligible publication types for our review; ii) Basturk et al, 2024[47]; Ng et al (2020)[48], and Van Geel et al (2020)[49] were both dance intervention studies which were not eligible for our review because the intervention was not primarily music-based; Baram and Miller (2007)[50] is about auditory feedback control but makes no mention of music and is therefore not eligible for our review; Moumdjian et al. (2019b[51], 2020[52]) were excluded because they examined movement synchronisation to music or metronome without assessing rehabilitation or clinical outcomes in people with MS.

Table 2. Inclusion criteria for the systematic review

Eligible studies assessed:

- Population: People with a diagnosis of Multiple Sclerosis
- Intervention: Studies involving music-based therapy or music interventions delivered for the purpose of rehabilitation in people with multiple sclerosis. Interventions could include:
    - Active music-making (e.g., instrument playing)
    - Passive listening to music (pre-recorded or live)
    - Rhythmic auditory stimulation (RAS)
    - Structured music therapy delivered by a trained therapist
    - Group or individual sessions of music-based therapy
- Comparator/Control: Studies with and without control arms were eligible. There were no specific requirements for what control arms should involve.
- Outcomes: Studies that report rehabilitation-related outcomes: functional recovery, motor function, gait and balance, cognitive function, psychological well-being, quality of life.
- Other: Studies available in full-text and published in the English language involving human participants aged 18 and over with a confirmed diagnosis of multiple sclerosis. Studies conducted in any healthcare, community, or rehabilitation setting, provided they meet the comparator and outcome criteria outlined above.

Table 3. Data Extracted

The following information was extracted for each included study:
• Bibliogrophic details (authors, year and citation).

- Country of study.
- Study design.
- Participants (sample size, sex profile, mean age or age range, MS type, disease duration, EDSS score, use of walking aids, left/right-handed – as this variable is often considered in neuropsychological studies).
- Inclusion criteria.
- Outcomes.
- Content of intervention.
- Professional background of intervention leader – as work in PD[8] has shown large differences in leader background and expertise within and between studies.
- Location of intervention (eg, health centre or home).
- Frequency and duration of intervention.
- Content of control arm.
- Professional background of leader of intervention delivered to the control arm.
- Location of control arm (eg, health centre or home).
- Frequency and duration of control arm.
- Study results for narrative synthesis for all eligible reported outcomes.

Table 4. Minimally clinically important differences identified

Outcome domain	Measure	Number of studies	MCID
Motor	Timed 25-foot walk test	6	MCID of a 17.2% improvement in test performance has been proposed based on trial data,[53] which accords well to previous clinical proposals of a 20% improvement
Cognitive	N/A	N/A	No pivotal measures used in at least three studies identified
Psychological	Beck depression	3	No MS-specific MCID identified, however noting clinical significance depends on baseline depression severity, a population-agnostic MCID of a

For peer review only

			17.5% reduction from baseline has been proposed[54]
Psychological	HADS anxiety	5	No MS-specific MCID identified, however an MCID of 1.70 points[55] has been found for HADS told in cardiovascular disease and 1.60 points[56] in chronic obstructive pulmonary disease. Halving for the anxiety subscale alone, gives an MCID of 0.80-0.85
Quality of life	MS Impact Scale-29 (MSIS-29)	3	No overall MCID identified,[57] although an MCID of 8 points has been proposed for the physical subscale.[58]

Table 5. GRADE scoring and explanation

Outcome domain	GRADE	Explanation
Motor	Low	16 studies, of which 10 RCTs (including feasibility RCTs but excluding secondary analysis of RCT data). Starting GRADE High as the majority of the evidence is randomised. Downgrade to Moderate because of heterogeneity. Downgrade to Low because the set of studies included evidence considered to be low quality.
Cognitive	Low	8 studies, of which 5 RCTs. Starting GRADE High as the majority of the evidence is randomised. Downgrade to Moderate because of heterogeneity. Downgrade to Low because the set of studies included evidence considered to be low quality.
Psychological	Low	7 studies, of which 4 RCTs. Starting GRADE High as the majority of the evidence is randomised. Downgrade to Moderate because of heterogeneity. Downgrade to Low because the set of studies included evidence considered to be low quality.

For peer review only

Quality of life	Low	12 studies, of which 8 RCTs. Starting GRADE High as the majority of the evidence is randomised. Downgrade to Moderate because of heterogeneity. Downgrade to Low because the set of studies included evidence considered to be low quality.

Table 6. Distribution of included studies according to rehabilitation outcome domains

First Author, Year	Motor outcomes	Cognitive outcomes	Psychological outcomes	Quality of life outcomes
Aldridge et al, 2005[25]	–	Global cognitive function	Depression, anxiety, self-acceptance	QoL, functional disability, fatigue
Conklyn et al, 2010[26]	Gait and mobility; muscle strength; spasticity and movement related disability	–	–	Pain; functional disability
Gatti et al, 2015[27]	Upper limb function	–	–	–
Goetschalckx et al, 2021[28]	Balance and stability; coordination and synchronisation	–	–	–
Helminger et al, 2025[29]	Gait and mobility	–	–	–
Impellizzeri et al, 2020[20]	–	Global cognitive function	Mood and emotional processing	MS specific QoL
Kuhlmann et al, 2025[30]	–	–	Anxiety; depression; body perception	Fatigue; pain
Maggio et al, 2021[31]	Gait and mobility; balance and stability	Cognitive abilities	Depression; emotional components	MS specific QoL
Moore et al, 2008[32]	–	Learning and memory; executive function and attention	–	–
Moumdjian et al, 2019[33]	Gait and mobility; coordination and synchronisation	–	–	Fatigue

Naderi et al, 2022[34]	Gait and mobility	–	–	–
Schmid et al, 2004[18]	Spasticity and movement related disability	Cognitive function	Depression; anxiety; self-acceptance	QoL; functional disability
Seebacher et al, 2015[35]	Gait and mobility	–	–	Fatigue
Seebacher et al, 2017[36]	Gait and mobility	–	–	QoL; fatigue
Seebacher et al, 2018[37]	Gait and mobility	–	–	QoL; fatigue
Seebacher et al, 2019[38]	Gait and mobility; coordination and synchronisation	–	–	QoL; fatigue
Seebacher et al, 2024[39]	Gait and mobility	Global cognitive function	Anxiety; depression; suicidality	QoL; fatigue
Seighalani et al, 2014[40]	–	–	Depression; anxiety; self esteem	–
Shahraki et al, 2017[41]	Gait and mobility	–	–	–
Thaut et al, 2008[42]	–	Learning and memory	–	–
Thaut et al, 2014[43]	–	Learning and memory; neural synchronisation	–	–
Wiens et al, 1999[19]	Muscle strength (respiratory)	–	–	–
Young et al, 2019[44]	Gait and mobility	–	–	Fatigue; pain

For peer review only

Table 7. Evaluation of minimally clinically important differences

Outcome domain	Measure	MCID	Findings

Motor	Timed 25-foot walk test	17.2% improvement[53]	• Helminger et al[29] – no clinically significant difference in participants receiving actual and/or imagined gait training with rhythmic-auditory cueing from baseline to follow-up.<br>• Seebacher et al[35] – clinically significant difference in participants receiving music cued motor imagery (17.3%) and metronome cued motor imagery (20.0%), but not controls, from baseline to follow-up.<br>• Seebacher et al[36] – no clinically significant difference in any group from baseline to follow-up.<br>• Seebacher et al[37] – no group-level clinically significant difference in any group from baseline to follow-up. One out of five participants in the music- and verbally cued motor imagery group had a clinically significant difference and no participants in other groups.<br>• Seebacher et al[38] – no group-level clinically significant difference in any group from baseline to follow-up. 21.1% of participants in the in the music- and verbally cued motor imagery group, 15.0% in the music-cued motor imagery and none in the motor imagery group had a clinically significant difference (defined as >=20% by the authors of this study).<br>• Seebacher et al[39] – no clinically significant difference in any group from baseline to the final follow-up (week 13).<br>One study out of six found evidence of a clinically significant difference
Psychological	Beck depression	17.5% reduction[54]	• Impellizeri et al[20] – clinically significant difference in experimental group (46.4%) but

For peer review only

			not the control group from baseline to follow-up.<br>• Maggio et al[31] – clinically significant difference in experimental group (56.2%) but not the control group from baseline to follow-up.<br>• Schmid et al[18] - numerical descriptive results not presented, therefore not evaluable<br>Two studies out of two (that were evaluable) found evidence of a clinically significant difference
Psychological	HADS anxiety	0.80 points (for subscale)[56]	• Kuhlmann et al[30] – no clinically significant difference from baseline to follow-up for the experimental group, although a clinically significant improvement (1.5 points) was found for the control group.<br>• Schmid et al[18] – numerical descriptive results not presented, therefore not evaluable<br>• Seebacher et al[39] – secondary outcome but data not presented, therefore not evaluable<br>No studies out of one (that were evaluable) found evidence of a clinically significant difference
Quality of life	MS Impact Scale-29 (MSIS-29)	8 points for physical subscale[58]	• Seebacher et al[38] – clinically significant difference in the music- and verbally-cued motor imagery group (15 points), but not the music-cued motor imagery or motor imagery groups. 79% of participants in the music- and verbally cued motor imagery group, 50% of participants in the music-cued motor imagery group, and 35% of participants in the motor imagery group had a clinically significant difference, defined as 7.5 points by the authors of this study.<br>• Seebacher et al[37] – no clinically significant difference in any group from baseline to follow-up (although the music-cued motor imagery

For peer review only

			group met the more lenient threshold of 7.5 points).<br>• Seebacher et al[36] – no clinically significant difference in any group from baseline to follow-up. 50% of participants in the music- and verbally cued motor imagery group, 41.2% in the music-cued motor imagery group, and 21.2% of those in the motor imagery group had a clinically significant difference, defined as 7.5 points by the authors of this study.<br>One study out of three found evidence of a clinically significant difference

Cognitive MCIDs could not be evaluated, as explained in Table 4.

Figure 1. PRISMA 2020 flow diagram. PRISMA, Preferred Reporting Items for Systematic Reviews and Meta-Analyses

For peer review only

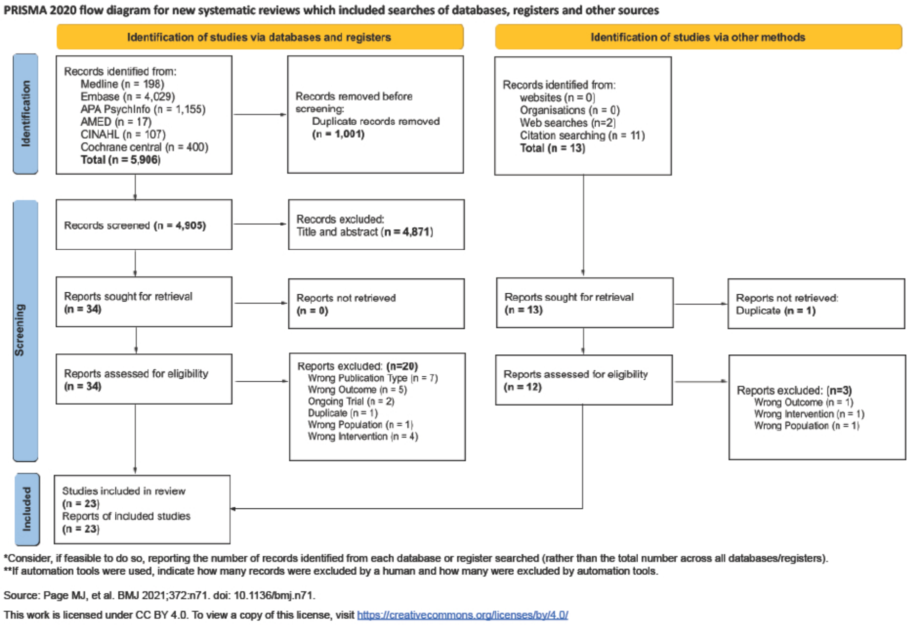

PRISMA 2020 flow diagram. PRISMA, Preferred Reporting Items for Systematic Reviews and Meta-Analyses

361x255mm (72 x 72 DPI)

Ahadi et al. Potential benefits of music-based therapies on rehabilitation outcomes in people with multiple sclerosis: a systematic review. Supplementary files.

Supplemental file 1. Completed PRISMA 2020 checklist

Section and Topic	Item #	Checklist item	Location where item is reported
**TITLE**			
Title	1	Identify the report as a systematic review.	1
**ABSTRACT**			
Abstract	2	See the PRISMA 2020 for Abstracts checklist.	2
**INTRODUCTION**			
Rationale	3	Describe the rationale for the review in the context of existing knowledge.	4
Objectives	4	Provide an explicit statement of the objective(s) or question(s) the review addresses.	4
**METHODS**			
Eligibility criteria	5	Specify the inclusion and exclusion criteria for the review and how studies were grouped for the syntheses.	5-6
Information sources	6	Specify all databases, registers, websites, organisations, reference lists and other sources searched or consulted to identify studies. Specify the date when each source was last searched or consulted.	5, Supp file 3
Search strategy	7	Present the full search strategies for all databases, registers and websites, including any filters and limits used.	Supp file 3
Selection process	8	Specify the methods used to decide whether a study met the inclusion criteria of the review, including how many reviewers screened each record and each report retrieved, whether they worked independently, and if applicable, details of automation tools used in the process.	5
Data collection process	9	Specify the methods used to collect data from reports, including how many reviewers collected data from each report, whether they worked independently, any processes for obtaining or confirming data from study investigators, and if applicable, details of automation tools used in the process.	5

For peer review only

Section and Topic	Item #	Checklist item	Location where item is reported
Data items	10a	List and define all outcomes for which data were sought. Specify whether all results that were compatible with each outcome domain in each study were sought (e.g. for all measures, time points, analyses), and if not, the methods used to decide which results to collect.	5, Table 3
	10b	List and define all other variables for which data were sought (e.g. participant and intervention characteristics, funding sources). Describe any assumptions made about any missing or unclear information.	5, Table 3
Study risk of bias assessment	11	Specify the methods used to assess risk of bias in the included studies, including details of the tool(s) used, how many reviewers assessed each study and whether they worked independently, and if applicable, details of automation tools used in the process.	5
Effect measures	12	Specify for each outcome the effect measure(s) (e.g. risk ratio, mean difference) used in the synthesis or presentation of results.	N/A – no meta-analysis
Synthesis methods	13a	Describe the processes used to decide which studies were eligible for each synthesis (e.g. tabulating the study intervention characteristics and comparing against the planned groups for each synthesis (item #5)).	5-6
	13b	Describe any methods required to prepare the data for presentation or synthesis, such as handling of missing summary statistics, or data conversions.	N/A – no meta-analysis
	13c	Describe any methods used to tabulate or visually display results of individual studies and syntheses.	N/A – no meta-analysis
	13d	Describe any methods used to synthesize results and provide a rationale for the choice(s). If meta-analysis was performed, describe the model(s), method(s) to identify the presence and extent of statistical heterogeneity, and software package(s) used.	5-6
	13e	Describe any methods used to explore possible causes of heterogeneity among study results (e.g. subgroup analysis, meta-regression).	N/A – no meta-analysis

For peer review only

Section and Topic	Item #	Checklist item	Location where item is reported
	13f	Describe any sensitivity analyses conducted to assess robustness of the synthesized results.	N/A – no meta-analysis
Reporting bias assessment	14	Describe any methods used to assess risk of bias due to missing results in a synthesis (arising from reporting biases).	N/A – no meta-analysis
Certainty assessment	15	Describe any methods used to assess certainty (or confidence) in the body of evidence for an outcome.	6
**RESULTS**			
Study selection	16a	Describe the results of the search and selection process, from the number of records identified in the search to the number of studies included in the review, ideally using a flow diagram.	Fig 1
	16b	Cite studies that might appear to meet the inclusion criteria, but which were excluded, and explain why they were excluded.	Supp file 5
Study characteristics	17	Cite each included study and present its characteristics.	Supp file 6
Risk of bias in studies	18	Present assessments of risk of bias for each included study.	Supp file 11
Results of individual studies	19	For all outcomes, present, for each study: (a) summary statistics for each group (where appropriate) and (b) an effect estimate and its precision (e.g. confidence/credible interval), ideally using structured tables or plots.	No meta-analysis, relevant info in Supp file 9
Results of syntheses	20a	For each synthesis, briefly summarise the characteristics and risk of bias among contributing studies.	7-8

For peer review only

Section and Topic	Item #	Checklist item	Location where item is reported
	20b	Present results of all statistical syntheses conducted. If meta-analysis was done, present for each the summary estimate and its precision (e.g. confidence/credible interval) and measures of statistical heterogeneity. If comparing groups, describe the direction of the effect.	N/A – no meta-analysis
	20c	Present results of all investigations of possible causes of heterogeneity among study results.	N/A – no meta-analysis
	20d	Present results of all sensitivity analyses conducted to assess the robustness of the synthesized results.	N/A – no meta-analysis
Reporting biases	21	Present assessments of risk of bias due to missing results (arising from reporting biases) for each synthesis assessed.	N/A – no meta-analysis
Certainty of evidence	22	Present assessments of certainty (or confidence) in the body of evidence for each outcome assessed.	8
**DISCUSSION**			
Discussion	23a	Provide a general interpretation of the results in the context of other evidence.	9
	23b	Discuss any limitations of the evidence included in the review.	9-10
	23c	Discuss any limitations of the review processes used.	9-10
	23d	Discuss implications of the results for practice, policy, and future research.	10
**OTHER INFORMATION**			
Registration and protocol	24a	Provide registration information for the review, including register name and registration number, or state that the review was not registered.	5
	24b	Indicate where the review protocol can be accessed, or state that a protocol was not prepared.	5
	24c	Describe and explain any amendments to information provided at registration or in the protocol.	5
Support	25	Describe sources of financial or non-financial support for the review, and the role of the funders or sponsors in the review.	11

For peer review only

Section and Topic	Item #	Checklist item	Location where item is reported
Competing interests	26	Declare any competing interests of review authors.	11
Availability of data, code and other materials	27	Report which of the following are publicly available and where they can be found: template data collection forms; data extracted from included studies; data used for all analyses; analytic code; any other materials used in the review.	11

From: Page MJ, McKenzie JE, Bossuyt PM, Boutron I, Hoffmann TC, Mulrow CD, et al. The PRISMA 2020 statement: an updated guideline for reporting systematic reviews. BMJ 2021;372:n71. doi: 10.1136/bmj.n71. This work is licensed under CC BY 4.0. To view a copy of this license, visit https://creativecommons.org/licenses/by/4.0/

Supplemental file 2. Completed PRISMA for Abstracts 2020 checklist

Section and Topic	Item #	Checklist item	Reported (Yes/No)
**TITLE**			
Title	1	Identify the report as a systematic review.	Yes
**BACKGROUND**			
Objectives	2	Provide an explicit statement of the main objective(s) or question(s) the review addresses.	Yes
**METHODS**			
Eligibility criteria	3	Specify the inclusion and exclusion criteria for the review.	Yes
Information sources	4	Specify the information sources (e.g. databases, registers) used to identify studies and the date when each was last searched.	Yes
Risk of bias	5	Specify the methods used to assess risk of bias in the included studies.	Yes
Synthesis of results	6	Specify the methods used to present and synthesise results.	Yes
**RESULTS**			
Included studies	7	Give the total number of included studies and participants and summarise relevant characteristics of studies.	Yes

Section and Topic	Item #	Checklist item	Reported (Yes/No)
Synthesis of results	8	Present results for main outcomes, preferably indicating the number of included studies and participants for each. If meta-analysis was done, report the summary estimate and confidence/credible interval. If comparing groups, indicate the direction of the effect (i.e. which group is favoured).	Yes
**DISCUSSION**			
Limitations of evidence	9	Provide a brief summary of the limitations of the evidence included in the review (e.g. study risk of bias, inconsistency and imprecision).	Yes
Interpretation	10	Provide a general interpretation of the results and important implications.	Yes
**OTHER**			
Funding	11	Specify the primary source of funding for the review.	Yes
Registration	12	Provide the register name and registration number.	Yes

Supplemental file 3. Detailed search strategies

Medline – via OVID

Search date: Wednesday 16th July 2025

1. *exp Multiple Sclerosis/*

2. *multiple sclerosis.mp.*

3. *MS.mp.*

4. *1 or 2 or 3*

5. *exp Music Therapy/*

6. *music therap*.mp.*

7. (music-based adj3 therap*).mp.

8. Music-based therapy.mp.

9. music intervention

For peer review only

10. music interventions.mp.

11. musical intervention.mp.

12. musical interventions.mp.

13. rhythmic auditory stimulation.mp.

14. sound therap.mp.*

15. singing therapy.mp.*

16. auditory stimulation.mp.*

17. 5 or 6 or 7 or 8 or 9 or 10 or 11 or 12 or 13 or 14 or 15

18. 4 and 17

19. limit 14 to English language

Results: 198

For peer review only

Search History (19) View Saved

#	Searches	Results	Runtime	Type	Actions		Annotations
1	exp Multiple Sclerosis/	75635	0.62	Advanced	Display Results	More	
2	Multiple Sclerosis.mp.	109818	0.98	Advanced	Display Results	More	
3	MS.mp.	478942	0.48	Advanced	Display Results	More	
4	1 or 2 or 3	531625	0.09	Advanced	Display Results	More	
5	exp Music Therapy/	4964	0.09	Advanced	Display Results	More	
6	music therap*.mp.	6740	2.61	Advanced	Display Results	More	
7	(music-based adj3 therap*).mp. [mp=title, book title, abstract, original title, name of substance word, subject heading word, floating sub-heading word, keyword heading word, organism supplementary concept word, protocol supplementary concept word, rare disease supplementary concept word, unique identifier, synonyms, population supplementary concept word, anatomy supplementary concept word]	103	2.42	Advanced	Display Results	More	
8	music-based therapy.mp.	27	0.64	Advanced	Display Results	More	
9	music intervention.mp.	700	0.34	Advanced	Display Results	More	
10	music interventions.mp.	456	0.32	Advanced	Display Results	More	
11	musical intervention.mp.	86	0.28	Advanced	Display Results	More	
12	musical interventions.mp.	68	0.27	Advanced	Display Results	More	
13	rhythmic auditory stimulation.mp.	215	0.52	Advanced	Display Results	More	
14	sound therap*.mp.	458	2.16	Advanced	Display Results	More	
15	singing therapy*.mp.	22	1.16	Advanced	Display Results	More	
16	auditory stimulation*.mp.	2366	0.63	Advanced	Display Results	More	
17	5 or 6 or 7 or 8 or 9 or 10 or 11 or 12 or 13 or 14 or 15 or 16	9799	0.02	Advanced	Display Results	More	
18	4 and 17	211	0.05	Advanced	Display Results	More	
19	limit 18 to english language	198	0.02	Advanced	Display Results	More	

Save Remove Combine with: AND OR Contract

Embase via Ovid

Search date: Wednesday 16th July 2025

1. *exp Multiple Sclerosis/*
2. *multiple sclerosis.mp.*
3. *MS.mp.*
4. *1 or 2 or 3*
5. *exp Music Therapy/*
6. *music therap*.mp.*
7. music-based therap*.mp.
8. music intervention,mp.

For peer review only - http://bmjopen.bmj.com/site/about/guidelines.xhtml

9. music interventions.mp.
10. musical intervention.mp.
11. *musical interventions.mp.*
12. *rhythmic auditory stimulation.mp.*
13. *sound therap*.mp.*
14. *singing therap*.mp.*
15. *auditory stimulation*.mp.*
16. *5 or 6 or 7 or 8 or 9 or 10 or 11 or 12 or 13 0r 14 or 15*
17. *4 and 16*
18. *limit 17 to English language*

results: 4029

#	Searches	Results	Runtime	Type	Actions		Annotations
1	exp multiple sclerosis/	176930	0.07	Advanced	Display Results	More	
2	multiple sclerosis.mp.	193474	0.77	Advanced	Display Results	More	
3	MS.mp.	664062	0.18	Advanced	Display Results	More	
4	1 or 2 or 3	754065	0.15	Advanced	Display Results	More	
5	exp music therapy/	11246	0.08	Advanced	Display Results	More	
6	music therap*.mp.	11935	4.36	Advanced	Display Results	More	
7	music-based therap*.mp.	61	4.43	Advanced	Display Results	More	
8	music intervention.mp.	1086	0.23	Advanced	Display Results	More	
9	music interventions.mp.	595	0.15	Advanced	Display Results	More	
10	musical intervention.mp.	143	0.22	Advanced	Display Results	More	
11	musical interventions.mp.	98	0.16	Advanced	Display Results	More	
12	rhythmic auditory stimulation.mp.	338	0.65	Advanced	Display Results	More	
13	sound therap*.mp.	625	4.48	Advanced	Display Results	More	
14	singing therap*.mp.	36	4.21	Advanced	Display Results	More	
15	auditory stimulation*.mp.	43773	0.43	Advanced	Display Results	More	
16	5 or 6 or 7 or 8 or 9 or 10 or 11 or 12 or 13 or 14 or 15	56404	0.02	Advanced	Display Results	More	
17	4 and 16	4168	0.07	Advanced	Display Results	More	
18	limit 17 to english language	4029	0.02	Advanced	Display Results	More	

Save Remove Combine with: AND OR Contract

APA PsycInfo via Ovid

For peer review only

Search date: Wednesday 16th July 2025

1. *exp Multiple Sclerosis/*
2. *multiple sclerosis.mp.*
3. *MS.mp.*
4. *1 or 2 or 3*
5. *exp Music Therapy/*
6. *music therap*.mp.*
7. music-based therap*.mp.
8. music intervention.mp.
9. music interventions.mp.
10. musical intervention.mp.
11. musical interventions.mp.
12. *rhythmic auditory stimulation.mp.*
13. *sound therap*.mp.*
14. *singing therap*.mp.*
15. *auditory stimulation*.mp.*
16. *5 or 6 or 7 or 8 or 9 or 10 or 11 or 12 or 13 0r 14 or 15*
17. *4 and 16*
18. *limit 17 to English language*

results: 1155

For peer review only

# ▲	Searches	Results	Runtime	Type	Actions		Annotations
1	exp Multiple Sclerosis/	15247	0.14	Advanced	Display Results	More	
2	Multiple Sclerosis.mp.	19196	1.06	Advanced	Display Results	More	
3	MS.mp.	37720	0.20	Advanced	Display Results	More	
4	1 or 2 or 3	44700	0.01	Advanced	Display Results	More	
5	exp Music Therapy/	6337	0.13	Advanced	Display Results	More	
6	Music therap*.mp.	7695	0.57	Advanced	Display Results	More	
7	music-based therap*.mp.	27	1.03	Advanced	Display Results	More	
8	music intervention.mp.	353	0.29	Advanced	Display Results	More	
9	music interventions.mp.	304	0.24	Advanced	Display Results	More	
10	musical intervention.mp.	59	0.29	Advanced	Display Results	More	
11	musical interventions.mp.	76	0.23	Advanced	Display Results	More	
12	rhythmic auditory stimulation.mp.	83	0.58	Advanced	Display Results	More	
13	sound therap*.mp.	167	0.68	Advanced	Display Results	More	
14	singing therap*.mp.	13	0.73	Advanced	Display Results	More	
15	auditory stimulation*.mp.	24980	0.60	Advanced	Display Results	More	
16	5 or 6 or 7 or 8 or 9 or 10 or 11 or 12 or 13 or 14 or 15	32845	0.01	Advanced	Display Results	More	
17	4 and 16	1175	0.01	Advanced	Display Results	More	
18	limit 17 to english language	1155	0.13	Advanced	Display Results	More	

Save Remove Combine with: AND OR Contract

AMED via EBSCOhost

Search date: Wednesday 16th July 2025

"multiple sclerosis" OR "MS"

AND

"music therap*" OR "music-based therap*" OR "musical intervention" OR "musical interventions" OR “music intervention” OR “music interventions” OR "rhythmic auditory stimulation*" OR "sound therap*" OR "singing therap*" OR "auditory stimulation*"

Limit = English language

Results: 17

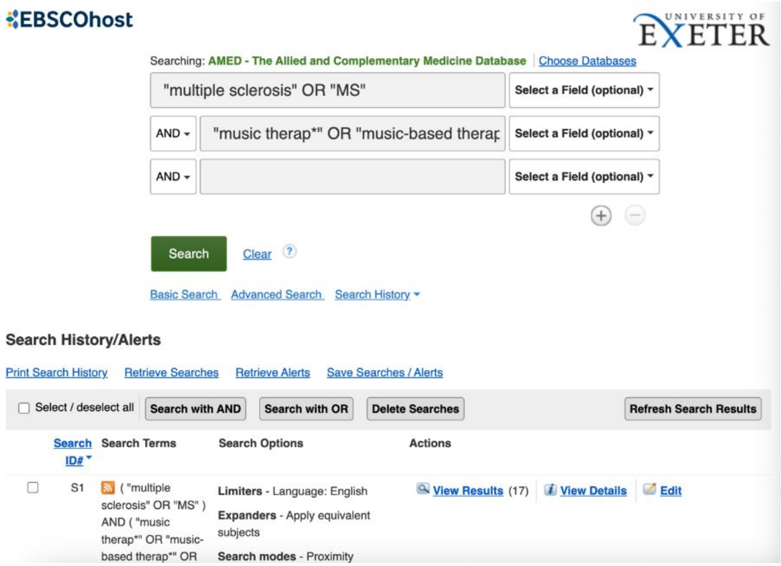

CINAHL Ultimate via EBSCOhost

Search date: Wednesday 16th July 2025

"multiple sclerosis" OR "MS"

AND

"music therap*" OR "music-based therap*" OR "musical intervention" OR "musical interventions" OR “music intervention” OR “music interventions” OR "rhythmic auditory stimulation*" OR "sound therap*" OR "singing therap*" OR "auditory stimulation*"

Limit = English language

Results: 107

For peer review only

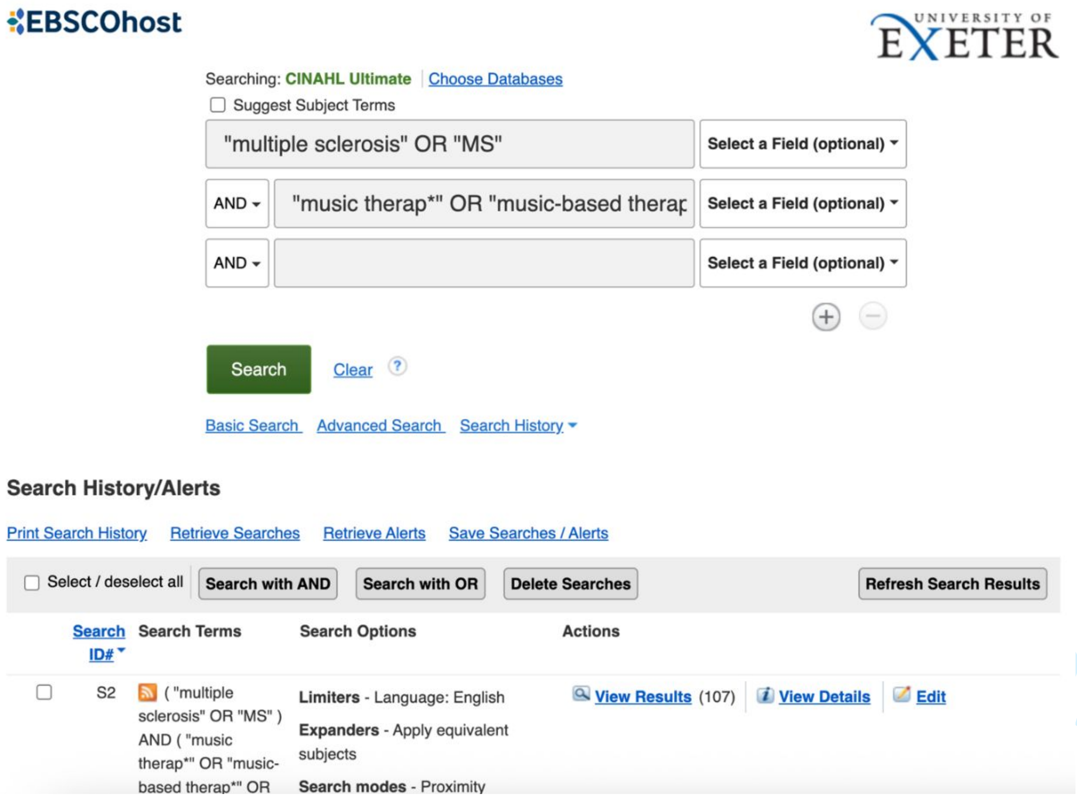

COCHRANE-Central

Search date: Wednesday 16th July 2025

"multiple sclerosis" OR "MS"

AND

(music therapy) OR (music-based therapy) OR (musical intervention) OR (musical interventions) OR (music intervention) OR (music interventions) OR (rhythmic auditory stimulation) OR (sound therapy) OR (singing therapy) OR (auditory stimulation)

Results: 400 trials (Trials = 392 are English and 8 non-English)

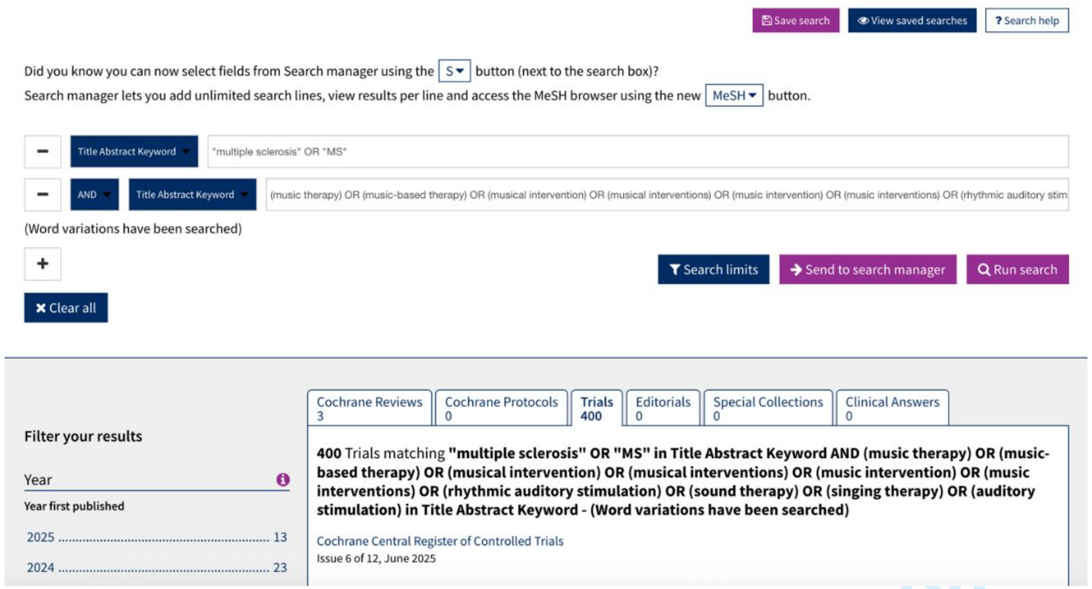

Supplemental file 4. Full list of included publications

1. Aldridge D, Schmid W, Kaeder M, et al. Functionality or aesthetics? A pilot study of music therapy in the treatment of multiple sclerosis patients. *Complement Ther Med* 2005; 13(1): 25–33.
2. Conklyn D, Stough D, Novak E, et al. A home-based walking program using rhythmic auditory stimulation improves gait performance in patients with multiple sclerosis: a pilot study. Neurorehabil Neural Repair 2010; 24(9): 835-42.
3. Gatti R, Tettamanti A, Lambiase S, et al. Improving hand functional use in subjects with multiple sclerosis using a musical keyboard: a randomized controlled trial. Physiother Res Int 2015; 20: 100-7.
4. Goetschalckx M, Van Geel F, Meesen R, et al. Rhythmic interlimb coordination of the lower limbs in multiple sclerosis during auditory pacing to three different frequencies. Gait Posture 2021; 334-40.
5. Helminger B, Seebacher B, Ropele S, et al. Effects of rhythmic-cued gait training on gait-like task related brain activation in people with multiple sclerosis. J Neurol Sci 2025; 471: 123426.
6. Impellizzeri F, Leonardi S, Latella D, et al. An integrative cognitive rehabilitation using neurologic music therapy in multiple sclerosis: a pilot study. Medicine 2020: 99(4): e18866.
7. Kuhlmann J, Ebner K, Zimmer A, et al. Music therapy with a monochord in multiple sclerosis (“MUTIMS”): A randomized, controlled, rater-blinded trial. Mult Scler J Exp Transl Clin 2025; 11(3):20552173251352712.
8. Maggio MG, Tripoli D, Porcari B, et al. How may patients with MS benefit from using music assisted therapy? A case-control feasability study investigating motor outcomes and beyond. Mult Scler Relat Disord 2021; 48: 102713.

9. Moore KS, Peterson DA, O'Shea G, et al. The effectiveness of music as a mnemonic device on recognition memory for people with multiple sclerosis. J Music Ther 2008; 45(3): 307-29.
10. Moumdjian L, Moens B, Maes PJ, et al. Continuous 12 min walking to music, metronomes and in silence: auditory-motor coupling and its effects on perceived fatigue, motivation and gait in persons with multiple sclerosis. Mult Scler Relat Disord 2019; 35: 92-9.
11. Naderi S, Sadeghi H, Amirseyfaddini M. The effects of rhythmic auditory stimulation with different tempos on spatio-temporal parameters and gait symmetry in patients with multiple sclerosis: a quasi-experimental study. J Res Rehabil Sci 2022; 18: doi: 10.48305/jrrs.2023.41771.1041.
12. Schmid W, Aldridge D. Active music therapy in the treatment of multiple sclerosis patients: a matched control study. J Music Ther 2004; 41(3): 225-40.
13. Seebacher B, Helmlinger B, Pinter D, et al. Actual and imagined music-cued gait training in people with multiple sclerosis: a double-blind randomized parallel multicenter trial. Neurorehabil Neural Repair 2024; 38(8): 555-69.
14. Seebacher B, Kuisma R, Glynn A, et al. Effects and mechanisms of differently cued and non-cued motor imagery in people with multiple sclerosis: a randomised controlled trial. Mult Scler J 2019; 25(12): 1593-1604.
15. Seebacher B, Kuisma R, Glynn A, et al. Exploring cued and non-cued motor imagery interventions in people with multiple sclerosis: a randomised feasibility trial and reliability study. Arch Physiother 2018; 8:6.
16. Seebacher B, Kuisma R, Glynn A, et al. The effect of rhythmic-cued motor imagery on walking, fatigue and quality of life in people with multiple sclerosis: a randomised controlled trial. Mult Scler J 2017; 23(2): 286-96.
17. Seebacher B, Kuisma R, Glynn A, et al. Rhythmic cued motor imagery and walking in people with multiple sclerosis: a randomised controlled feasibility study.
Pilot Feasibility Stud 2015; 1: 25.
18. Seighalani MZ, Ghahari S, Zarbakhsh M. The effectiveness of music therapy on depression, anxiety and self-esteem of patients with multiple sclerosis (MS). Adv Environ Biol 2014; 8(9): 253-60.
19. Shahraki M, Sohrabi M, Taheri Torbati HR, et al. Effect of rhythmic auditory stimulation on gait kinematic parameters of patients with multiple sclerosis. J Med Life 2017; 10(1): 33-7.
20. Thaut MH, Peterson DA, Sena KM, et al. Musical structure facilitates verbal learning in multiple sclerosis. *Music Percept* 2008; 25(4): 325–30.
21. Thaut MH, Peterson DA, McIntosh GC, et al. Music mnemonics aid verbal memory and induce learning – related brain plasticity in multiple sclerosis. Front Hum Neurosci 2014; 8: 395.
22. Wiens ME, Reimer MA, Guyn HL. Music therapy as a treatment method for improving respiratory muscle strength in patients with advanced multiple sclerosis: a pilot study. Rehabil Nurs 1999; 24(2): 74-80.
23. Young HJ, Mehta TS, Herman C, et al. The effects of M2M and adapted yoga on physical and psychosocial outcomes in people with multiple sclerosis. Arch Phys Med Rehabil 2019;100(3):391-400.

List of studies for each outcome domain (studies can contribute to multiple outcome domains)

<u>Motor</u>

1. Conklyn et al. 2010
2. Gatti et al. 2015
3. Goetschalckx. et al. 2021
4. Helminger. et al. 2025
5. Maggio et al. 2021
6. Moore et al. 2008

For peer review only

7. Moumdjian et al. 2019
8. Naderi et al 2022
9. Seebacher et al. 2015
10. Seebacher et al. 2017
11. Seebacher et al. 2018
12. Seebacher et al. 2019
13. Seebacher et al. 2024
14. Shahraki et al, 2017
15. Wiens et al. 1999
16. Young et al. 2019

<u>Cognitive</u>

1. Aldridge et al. 2005
2. Impellizzeri et al. 2020
3. Maggio et al. 2021
4. Moore et al. 2008
5. Moumdjian et al. 2019
6. Seebacher et al. 2024
7. Thaut et al 2008
8. Thaut et al 2014

<u>Psychological</u>

1. Aldridge et al. 2005
2. Impellizzeri et al. 2020
3. Kuhlmann et al. 2025
4. Maggio et al. 2021
5. Schmid et al. 2004
6. Seebacher et al. 2024
7. Seighalani et al. 2014

<u>Quality of life</u>

1. Aldridge et al. 2005
2. Impellizzeri et al. 2020
3. Kuhlmann et al. 2025
4. Maggio et al. 2021

For peer review only

5. Moumdjian. et al. 2019
6. Schmid et al. 2004
7. Seebacher et al. 2015
8. Seebacher et al. 2017
9. Seebacher et al. 2018
10. Seebacher et al. 2019
11. Seebacher et al. 2024
12. Young et al. 2019

Supplemental file 5. Full list of studies excluded at full text review with reasons

Duplicate

1. Lambiase S, Nava C, Tettamanti A, et al. Improving hand functional use in subjects with multiple sclerosis using a musical keyboard: a randomized controlled trial. Physiother Res Int 2015;20: 100-7. [This is Gatti et al, 2015, included study].
2. Moore KS, Peterson DA, O'Shea G, et al. The effectiveness of music as a mnemonic device on recognition memory for people with multiple sclerosis. *J Music Ther* 2008; 45(3): 307–29.

Intervention

3. Baram Y, Miller A. Auditory feedback control for improvement of gait in patients with multiple sclerosis. J Neurol Sci 2007; 254: 90-4.
4. Basturk S, Ekici G, Kirteke F, et al. Therapeutic effects of line dancing in people with multiple sclerosis: an evaluator-blinded, randomized controlled study. Art Health 2024; doi: 10.1080/17533015.2024.2325425.
5. Mandelbaum R, Triche EW, Fasoli SE, et al. A pilot study: examining the effects and tolerability of structured dance intervention for individuals with multiple sclerosis. *Disabil Rehabil* 2016; 38(3): 218–22
6. Trinchillo A, Caliendo D, Nicolella V, et al. Tango classes in people with Multiple Sclerosis (PwMS): Impact on motor and non-motor functions. Clin Neurol Neurosurg 2024; 243: 108380.
7. Van Geel F, Van Asch P, Veldkamp R, et al. Effects of a 10-week multimodal dance and art intervention program leading to a public performance in persons with multiple sclerosis: a controlled pilot-trial. Mult Scler Relat Disord 2020; 44: 102256.

Outcome

8. Fasching B, Mildner S, Fink F, et al. MuSic Moves-co-creating a music-supported exercise programme with and for people with multiple sclerosis: a bicentre participatory mixed methods study. BMJ Open 2024; 14(11): e091168.
9. Moumdjian L, Maes PJ, Dalla Bella S, et al. Detrended fluctuation analysis of gait dynamics when entraining to music and metronomes at different tempi in persons with multiple sclerosis. Sci Rep 2020; 10: 12934.
10. Moumdjian L, Moens B, Maes PJ, et al. Walking to music and metronome at various tempi in persons with multiple sclerosis: a basis for rehabilitation. Neurorehabil Neural Repair 2019; 33(6): 464-75.

For peer review only

11. Seebacher B, Helmlinger B, Hotz I, et al. Actual and imagined music-cued gait training for people with multiple sclerosis: a multicentre qualitative study. BMJ Open 2024; 14: e086555.
12. Seighalani MZ, Cheraghali Gol H, Rostami AM. The effectiveness of music therapy on depression, anxiety and self-esteem of patients with multiple sclerosis. *Adv Environ Biol* 2014; 8(10): 759–64.
13. Vanbilsen N, Moumdjian L, Kinnaert F, et al. Step to the beat: Auditory-motor coupling during walking to higher and lower tempi with music and metronomes in progressive multiple sclerosis: an observational study. Mult Scler Relat Disord 2024; 92: 106152.

Population

14. Srinithiwat P, Foocharoen C, Mahakkanukrauh A, et al. Patient perspectives and satisfaction following a short-term singing intervention in systemic sclerosis patients: a cross-sectional study. *J Med Assoc Thai* 2025; 108: S59–65.
15. Young HJ, Lai B, Wilroy J, et al. Effects of a teleexercise movement-to-music intervention on health outcomes in individuals with mobility disabilities: a randomized controlled trial. Arch Phy Med Rehabil 2025; https://doi.org/10.1016/j.apmr.2025.05.017.

Publication type

16. https://clinicaltrials.gov/study/NCT05792176. Ukulele playing to improve cognition in people with multiple sclerosis: a feasibility study [No results posted].
17. https://clinicaltrials.gov/study/NCT04019912. The novel use of treadmill plus music in MS patients gate rehabilitation (SMUSIC) [No results posted – spelling mistake ‘gate’ for ‘gait’ was made on Clinical Trials.gov].
18. https://clinicaltrials.gov/study/NCT04314076. Rhythmic auditory stimulation & gait training [No results posted].
19. https://clinicaltrials.gov/study/NCT01086371. Rhythmic auditory stimulation and walking performance in multiple sclerosis (MS) patients [No results posted].
20. https://clinicaltrials.gov/study/NCT06800144. Effect of music therapy on pain in people with multiple sclerosis [No results posted].
21. https://trialsearch.who.int/Trial2.aspx?TrialID=IRCT20230219057455N1. The effectiveness of slow and fast music on anxiety and quality of life of people with multiple sclerosis [No results posted].
22. https://trialsearch.who.int/Trial2.aspx?TrialID=DRKS00020469. Effectiveness of playing the instruments „Big Bom“and “Temple Blocks” as a music therapy approach to improve gait function among patients with multiple sclerosis [No results posted].
23. Bethoux F, Awad L, Gallagher L, et al. Safety and acceptability of closed-loop rhythmic cueing for gait training in persons with multiple sclerosis: a pilot single-blind randomized controlled trial. Mult Scler J 2023: 29(3): 645 [Abstract].
24. Pedulla L, Addeo S, Rinaldi S, Aerobic training in multiple sclerosis: a pilot study on the effects of music during group treatment. Mult Scler J 2020; *26* (Suppl 2): 54-55 [Abstract].

For peer review only

Supplemental file 6. Study characteristics for included studies.

Author, year	Country	Design	Participants	Inclusion criteria	Exclusion criteria	Outcomes
Aldridge et al. 2005	Germany	Matched controlled pilot study	20 people with MS (10 per arm). 30% male Age range: 29-47	Diagnosis of multiple sclerosis and within the EDSS range used for matching	Pregnancy and mental disorders requiring medication	Primary: Psychological outcomes such as Depression (Beck, HADS), Anxiety (HADS), Self-acceptance (SESA) Secondary: Quality of life (HAQUAMS) Functional disability (EDSS) Cognitive (MSFC) and motor function (MSFC) Fatigue (IFSS)
Conklyn et al, 2010	USA	RCT	10 people with MS (5 per arm), 30% male, Mean age: intervention 47, control 50	Age 18 or over; medical diagnosis of MS; able to walk 30.48m without physical assistance (assistive devices were allowed); able to walk 7.62m in 8 to 60s [distances were published in imperial feet]	Treatment for MS exacerbation in past 30 days; severe comorbidity precluding participation; severe cognitive deficits precluding informed consent or preventing following study instructions	Primary: Gait parameters<br><br>Secondary: Spasticity (MAS Modified Ashworth Scale); muscle strength (Manual Muscle Testing); Pain (out of 10); Ambulation disability (Ambulation Index); MS-related disability

For peer review only

						(Patient-determined Disease Steps); Treatment efficacy (Subject Global Impression, Clinician Global Impression)
Gatti et al, 2015	Italy	RCT	19 people with MS (9 intervention, 10 control), 37% male, mean age intervention 43, control 48	Adults; primary or secondary progressive MS; no relapse or medication changes in past 3 months; hand strength below normal; Nine-Hole Peg Test >18s; no experience playing a musical instrument; no cognitive or psychiatric disorders; no hearing or visual deficits	No additional criteria	Primary: Perceived hand functional use (ABILHAND)<br><br>Secondary: Hand dexterity (Nine-Hole Peg Test); Hand strength (Jamar and Pinch)
Goetschalckx et al, 2021	Belgium	Observational cross-sectional comparative study	38 people with MS (16% male, mean age 49).13 healthy controls (38% male, mean age 52)	MS diagnosis (McDonald criteria); age 18-70; able to walk independently	Other medical conditions influencing walking	Primary: Bilateral gait coordination (Phase Coordination Index)<br><br>Secondary: movement amplitude; movement frequency
Helminger et al, 2025	Austria	Secondary analysis of a randomised controlled trial (longitudinal interventional study)	31 people with MS (52% male, mean age 44). 17 health controls (53% male, mean age 41)	Age at least 18; definite MS (Thompson criteria); mild-to-moderate disability (Expanded Disability Status Scale 2-5);	Relapse or change in disease-modifying treatment or physiotherapy in past 3 months; concomitant	Primary: Walking function (2-minute walk test; timed 25-foot walk)<br><br>Secondary: fMRI brain activation

For peer review only

					diseases; MRI contraindications	changes during movement tasks; correlation of brain activation changes with walking performance
Impellizzeri et al, 2020	Italy	RCT	30 people with MS (15 per arm), 63% male, mean age intervention 52, control 51	MS diagnosis (Lublin criteria); Expanded Disability Status Scale 3-7; enjoy music; no visual or hearing loss; no severe medical and psychiatric illness	No additional criteria	Cognition (Brief Repeatability Battery of Neurophysiology); quality of life (MSQoL-54); depression (Beck); emotions (Emotional Awareness Questionnaire)
Kuhlmann et al, 2025	Switzerland	RCT	57 people with MS (30 intervention, 27 control), 18% male, mean age 50	Aged at least 18; MS diagnosis (McDonald criteria); Expanded Disability Status Scale no more than 6.5	Severe psychiatric disorders; other life-threatening or severely disabling physical disorders; >2 MS relapses in past year or relapse in past 3 months; change in disease-modifying treatment or symptomatic MS medication in past 3 months; pregnancy; unable to lie supine for 15 min; severely compromised hearing (uncorrected);	Primary: Anxiety (HADS)<br><br>Secondary: Depression (HADS); fatigue (self-report); pain (quantitative sensory testing); body perception (self-report)

For peer review only

					numbness on the back of both hands	
Maggio et al, 2021	Italy	Quasi-experimental controlled feasibility study	20 people with MS (10 per arm), 35% male	Secondary progressive MS (Lublin criteria); Expanded Disability Status Scale 3-6; enjoy music; no hearing or visual loss; no change in disease-modifying drugs in past 6 months	Age <65; other severe medical conditions or psychiatric illnesses; history of neuroplasms or other neurological conditions; moderate-severe cognitive decline (MoCA <21); neurologic music therapy in past 3 months	Motor evaluation (Berg Balance; Timed Up and Go; 10-metre walk test); neuropsychological battery (Beck depression; MSQOL-54 quality of life; Goal Attainment Scaling); usability (System Usability Scale)
Moore et al, 2008	USA	RCT	38 people with MS (20 intervention, 18 control), 21% male, mean age 53	Right-handed; non-smoker; Extended Disability Status Scale 3.5-7; at least 5 MS brain lesions on MRI; stable on immunomodulatory therapy; less than 2 exacerbations in past 12 months	Active exacerbation phase; treatment with pulse-cortical steroids in past 3 months; history of seizure disorder, traumatic brain injury or substance abuse; taking cognition-enhancing acetylecho-linesterase inhibitors	Verbal learning and memory (adapted Auditory-Visual Learning Test) – sensitivity index; response bias index
Moumdjian et al, 2019	Belgium	Case-control study	31 people with MS (26% male, mean age 53). 30 healthy controls (27% male, mean age 52). Note that only 27 people	Aged at least 18; diagnosis of MS for over 1 year; no exacerbation in past month; average	Pregnancy; hearing impairment; cognitive impairment hindering understanding of instructions	Primary: Auditory-motor coupling and synchronisation<br><br>Secondary: Perceived physical

For peer review only

			with MS and 30 healthy controls completed the experimental session	comfortable walking speed 0.4-1.2 m/s		and cognitive fatigue (Modified Fatigue Impact Scale); motivation (Likert); cadence; stride length; velocity
Naderi et al, 2022	Iran	Quasi-experimental controlled feasibility study	13 people with MS (0% male, mean age 38). 14 healthy controls (0% male, mean age 38)	MS confirmed by neurologist; Expanded Disability Status Scale 3.5-5.5; no vision or hearing problems; no other active treatment; able to walk without assistive devices	Unable to perform tasks; severe attacks or relapse on day of the test	Primary: Gait (Gait Symmetry Index) – step duration symmetry; stride length symmetry<br><br>Secondary: Additional gait parameters
Schmid et al, 2004	USA	Matched control study	20 people with MS (10 per arm), 30% male, age range 29-47	Not specifically stated (only exclusion criteria)	Pregnancy; mental disorders requiring medication; previous experience of music therapy	Depression (Beck); anxiety (HADS); self-acceptance (Social and Emotional Skills Assessment); quality of life (Hamburg Quality of Life Questionnaire in MS); cognitive and functional disability (Expanded Disability Status Scale)
Seebacher et al, 2024	Austria	RCT	127 people with MS (split 43, 44, 40 across the 3 music therapy arms), 25% male, mean age per arm range 47-50	Age 18 or over; MS diagnosis (McDonald criteria); Expanded Disability Status Scale 2-5; stable disease	Cognitive impairment (MoCA <26), anxiety or depression (HADS subscales 11 or over); suicidality; relapse, starting new medication, medication	Primary: Walking speed (timed 25-foot walk); change in walking distance (2-minute walk test)<br><br>Secondary: Cognition (MoCA); anxiety and

For peer review only

					adjustment or physiotherapy in past 3 months or during the study	depression (HADS); suicidality (narrative screening); fatigue (Neurological Fatigue Index-MS); quality of life (MS International Quality of Life); motor imagery (Kinesthetic and Visual Imagery Questionnaire-10)
Seebacher et al, 2019	Austria	RCT	59 people with MS (split 19, 20, 20 across the 3 music therapy arms), 20% male, mean age 45	Aged 18 or over; German speaking; clinically definite MS (McDonald criteria); mild-to-moderate disability (Expanded Disability Status Scale 1.5-4.5)	Concomitant diseases affecting interventions or walking; relapse in past 3 months or during the study; change of treatment in past 2 months or during the study; pregnancy; clinical symptoms of depression or cognitive dysfunction	Primary: Walking speed (timed 25-foot walk); change in walking distance (2-minute walk test)<br>Secondary: Fatigue (Modified Fatigue Impact Scale); quality of life (MS Impact Scale-29); motor imagery (Kinesthetic and Visual Imagery Questionnaire-10); sensory motor synchronisation
Seebacher et al, 2018	Austria	Feasibility RCT	15 people with MS (split 5, 5, 5 across the 3 music therapy arms), 13% male, mean age 52	Age 18 or over; MS (McDonald criteria); mild-to-moderate disability (Expanded Disability Status Scale 1.5-4.5); German speaking	Concomitant diseases affecting rhythmic cued motor imagery or walking; relapse in past 3 months or during the study; change of medication or start of physiotherapy in past 2 months;	Primary: Feasibility; safety and adverse events<br><br>Secondary: Walking speed (timed 25-foot walk); walking distance (6-minute walk test); fatigue (Modified Fatigue

For peer review only

					pregnancy; overt symptoms of depression or cognitive dysfunction	Impact Scale); quality of life (MS Impact Scale-29); motor imagery (Kinesthetic and Visual Imagery Questionnaire-10)
Seebacher et al, 2017	Austria and UK	RCT	101 people with MS (34 for each of the 2 music therapy arms, 33 controls), 16% male, mean age 44	Aged 18 or over; clinically definite MS (McDonald criteria); mild-to-moderate disease (Expanded Disability Status Scale 1.5-4.5)	Concomitant diseases affecting interventions or walking; relapse in past 3 months; change of medication or physiotherapy in past 2 months; pregnancy; overt symptoms of depression or cognitive dysfunction	Primary: Walking speed (timed 25-foot walk); change in walking distance (2-minute walk test)<br><br>Secondary: Walking perception (MS Walking Scale-12); fatigue (Modified Fatigue Impact Scale); quality of life (MS Impact Scale-29)
Seebacher et al, 2015	Austria	Feasibility RCT	30 people with MS (10 for each of the 2 music therapy arms, 10 controls), 27% male, mean age per arm ranged 42-47	Aged 18 or over; diagnosis of MS (McDonald criteria); mild-to-moderate disability (Expanded Disability Status Scale 1.5-4.5); German speaking	Concomitant diseases affecting rhythmic cued motor imagery or walking; relapse in past 3 months or during the study; medication change in past 2 months; pregnancy; overt symptoms of depression or cognitive dysfunction	Primary: Feasibility; fatigue (Modified Fatigue Impact Scale)<br><br>Secondary: Walking speed (timed 25-foot walk); change in walking distance (2-minute walk test)
Seighalani et al, 2014	Iran	RCT	30 people with MS (15 per arm), gender and age not reported	MS diagnosis by physician; self-esteem below	Cooper Smith self-esteem 25 or above	Depression (Beck); anxiety (Beck); self-

For peer review only

				average (Cooper Smith <25)		esteem (Cooper Smith)
Shahraki et al, 2017	Iran	RCT	18 people with MS (9 per arm), 22% male, mean age intervention 40, control 38	Age 18 or over; Expanded Disability Status Scale 3-6; able to walk 30.48m without assistance; lack of treatment for relapse or MS exacerbation in past 30 days; lack of cardiovascular and rheumatic diseases; lack of severe pain in lower joints; no engagement in regular physical activity in past 3 months; no hearing impairment	No additional criteria	Gait parameters
Thaut et al, 2008	USA	RCT	20 people with MS (10 per arm), 10% male, mean age: intervention 51.7, control 53.8	Right-handed; relapse-remitting MS; normal hearing; no history of other neurological or psychiatric conditions	No additional criteria	Verbal learning and word order memory Recall of higher order word sequences (3–7 words)
Thaut et al, 2014	Germany and USA	RCT	54 people with MS (number per arm not reported), 30% male, mean age intervention 50, control 53	Right-handed; relapse-remitting MS; normal hearing; no history of other neurological or psychiatric conditions; at least 5 brain lesions on MRI;	In active exacerbation phase; treated with pulse-cortical steroids or cognition-enhancing AChE inhibitors	Learning; memory; brain activity with oscillatory network synchronisation

For peer review only

				stable on immunomodulatory therapy; <2 exacerbations in past 12 months		
Wiens et al, 1999	Canada	RCT	19 people with MS (9 intervention, 10 control), gender not reported, mean age 55	Residents in a long-term care centre with MS; able to tolerate 30 minutes of concentrated effort; able to follow verbal instructions; MMSE 20 or above; free from respiratory infection; able to sit in a wheelchair for at least 2 hours	No additional criteria	Strength of respiratory muscles (mouth pressure testing) – inspiratory and expiratory
Young et al, 2019	USA	RCT	81 people with MS (53 intervention across 2 arms, 28 control), 19% male, mean age per arm ranged 47-50	Aged 18-65; self-reported MS diagnosis; Patient-Determined Disease Steps 0-6; able to exercise with arms and/or legs; physician clearance	Participation in similar intervention in past 6 months; use of tobacco in past 6 months; unstable weight; cognitive impairment (MMSE <24); active pressure ulcer; contraindications to exercise (American College of Sports Medicine guidelines)	Primary: Motor evaluation (Timed Up and Go; 6-minute walk test; 5-times sit-to-stand)<br><br>Secondary: Fatigue (Patient-Reported Outcomes Measurement Information System Fatigue); pain (Pain Interference Short Form 8a.)

For peer review only

Supplemental file 7. Intervention profile for included studies.

Author, year	Content	Leader	Location	Duration
Aldridge et al. 2005	Nordoff Robbins approach: Active music therapy - Music-making on instruments, or singing	Therapist	Health facility	3 blocks of music therapy in single sessions over the course of the project (8—10 sessions, respectively)
Conklyn et al, 2010	Rhythmic auditory stimulation	Self	Home	2 sessions of 20 min/day for 4 weeks
Gatti et al, 2015	Playing keyboard (numbered, coloured stickers to guide)	Physiotherapist	Health facility	30 minutes a day, 5 days a week for 3 weeks
Goetschalckx et al, 2021	Rhythmic auditory stimulation with metronome	Facilitator	Health facility	1 single session (each metronome condition lasted 1 minute with a 1-minute rest in between conditions)
Helminger et al, 2025	Actual and/or imagined gait training with rhythmic-auditory cueing	Self	Home	4 sessions of 30 minutes per week for 4 weeks
Impellizzeri et al, 2020	Neurologic music therapy (plus conventional cognitive rehabilitation)	Music therapist	Health facility	3 sessions of 60 minutes per week for 8 weeks
Kuhlmann et al, 2025	Listening to a monochord being played while lying on top of it and feeling the vibration	Music therapist	Health facility	1 session of 45 minutes per week for 6 weeks
Maggio et al, 2021	Listening to music while walking on a treadmill	Physiotherapist	Health facility	3 sessions of 30 minutes per week for 8 weeks
Moore et al, 2008	Hearing word lists in song format and being asked to sing them back	Research team	Health facility	Single session (testing time approximately 60 minutes)

For peer review only

Moumdjian et al, 2019	Rhythmic auditory stimulation (2 groups: music and metronome)	Research team	Health facility	Single session of 12 minutes
Naderi et al, 2022	Rhythmic auditory stimulation	Facilitator	Health facility	Single session (4 walking modes, each 2 minutes walking with 30s rhythm exposure beforehand, and 3-minute rest between modes)
Schmid et al, 2004	Active music therapy (singing or instruments)	Music therapist	Health facility	3 blocks of 8-10 sessions over 1 year
Seebacher et al, 2024	3 groups: cued motor imagery; combined cued motor imagery and cued gait training; cued gait training	Physiotherapist, occupational therapist, psychologist	Home	4 sessions of 30 minutes (including short breaks) per week for 4 weeks
Seebacher et al, 2019	3 groups: music and verbally cued motor imagery; music cued motor imagery; non-cued motor imagery	Self	Home	6 sessions of 17 minutes per week for 4 weeks
Seebacher et al, 2018	3 groups: music and verbally cued motor imagery; music cued motor imagery; non-cued motor imagery	Self	Home	6 sessions of 17 minutes per week for 4 weeks
Seebacher et al, 2017	2 groups: music cued motor imagery; metronome cued motor imagery	Self	Home	6 sessions of 17 minutes per week for 4 weeks
Seebacher et al, 2015	2 groups: music cued motor imagery; metronome cued motor imagery	Self	Home	6 sessions of 17 minutes per week for 4 weeks
Seighalani et al, 2014	Active music therapy (discussion about music; making music with mouth; expressing the effects of music) and passive music therapy (listening to music)	Researcher and music therapist	Health facility	15 sessions of 30 minutes in total (6 active and 9 passive)

For peer review only

Shahraki et al, 2017	Rhythmic auditory stimulation with metronome	Coach	Health facility	3 sessions of 30 minutes per week for 3 weeks
Thaut et al, 2008	Ray's Auditory Verbal Learning Test – sung word lists	Research team	Health facility	Single session
Thaut et al, 2014	Ray's Auditory Verbal Learning Test – sung word lists	Research team	Health facility	Single session
Wiens et al, 1999	Music therapy: focusing on singing: relaxation and diaphragmatic breathing, intonation of syllables, and reading or singing phrases, paragraphs, and simple songs	Music therapist with instrument speciality in voice	Health facility	3 sessions of 30 minutes per week for 12 weeks
Young et al, 2019	2 groups: music to movement; adapted yoga. The yoga group does not meet the inclusion criteria as an intervention for this review and is not presented as a comparator.	Self	Fitness facility	3 sessions of 60 minutes per week for 12 weeks

Supplemental file 8. Control profile for included studies.

**Author, year**	**Synopsis of control arm**
Aldridge et al. 2005	No Intervention
Conklyn et al, 2010	No intervention
Gatti et al, 2015	Playing the keyboard with it turned off
Goetschalckx et al, 2021	Healthy control group who had the same intervention
Helminger et al, 2025	Healthy control group who had no music therapy
Impellizzeri et al, 2020	6 sessions per week for 8 weeks of conventional cognitive rehabilitation

For peer review only

Kuhlmann et al, 2025	Patients laid on top of a monochord and remained relaxed in a supine position in silence
Maggio et al, 2021	Conventional cognitive rehabilitation (same amount of conventional physiotherapy, stretching, muscle strength and occupational therapy as intervention group)
Moore et al, 2008	Spoken word group – learn and recall words without music
Moumdjian et al, 2019	Healthy control group who had no music therapy or metronome
Naderi et al, 2022	Healthy control group who had the same intervention
Schmid et al, 2004	No intervention
Seebacher et al, 2024	[No control arm]
Seebacher et al, 2019	[No control arm]
Seebacher et al, 2018	[No control arm]
Seebacher et al, 2017	No music therapy
Seebacher et al, 2015	No music therapy
Seighalani et al, 2014	No intervention
Shahraki et al, 2017	Exercises as per intervention group but without rhythmic auditory stimulation
Thaut et al, 2008	Spoken word list (same words as sung by intervention group)
Thaut et al, 2014	Spoken 15-word list (same words as sung by intervention group)
Wiens et al, 1999	3 sessions of 30 minutes per week for 12 weeks with a nurse – listened to music, discussed music, learnt about music and composers (but no active music therapy)
Young et al, 2019	No active music therapy or yoga

For peer review only

Supplementary file 9. Narrative results for included studies

Author, year	Results
Aldridge et al. 2005	There were no statistically significant differences between the music therapy group and the control group; however, medium effect sizes were observed for self-esteem (d = 0.54), depression (HAD-D d = 0.63), and anxiety (HAD-A d = 0.63). Within the music therapy group, significant improvements in self-esteem, depression, and anxiety were seen over the treatment period (U1–U4), with deterioration in fatigue, anxiety, and self-esteem during follow-up after therapy cessation.
Conklyn et al, 2010	Compared to no intervention, rhythmic auditory stimulation was associated in statistically significant greater improvement in double-support time (left, p = .0176; right, p = .0247). No statistically significant between-group differences were found for the other outcome measures. However, trends with medium to high effect sizes were observed for other gait parameters including walking speed. Within-group analysis showed statistically significant improvement in cadence, stride length, step length, velocity, and normalized velocity. Participant satisfaction level with the rhythmic auditory stimulation intervention was high.
Gatti et al, 2015	Compared to playing with the keyboard turned off, playing with the keyboard turned on was associated with a statistically significant improvement in hand function (p=0.003, Cohen's d = 1.66), while both groups showed a statistically significant improvement in hand dexterity, although between-group differences were not statistically significant. There was also no statistically significant between-group difference in hand strength.
Goetschalckx et al, 2021	Reported results that people with MS performed more poorly than healthy controls were not surprising and outside the scope of the present review. Within people with MS, interlimb coordination was worse at 0.75Hz metronome frequency compared to 1.00Hz and 1.50Hz. Movement amplitude was greatest at 1.00 Hz but reduced at both 0.75Hz and 1.50HZ. Movement frequency followed the metronome tempo. Poorer coordination was statistically significantly correlated with poorer cognitive performance at 0.75Hz and 1.50Hz metronome frequencies, and with reduced walking capacity especially at the 1.50Hz metronome frequency.
Helminger et al, 2025	In people with MS, walking distance in the 2-minute walk test showed a statistically significant improvement only in those classified as responders (>5% improvement), while no statistically significant improvement was shown in the timed 25-foot walk test. Responders showed reductions in brain activation within the premotor cortex, precuneus, and middle frontal gyrus, areas linked to motor planning, spatial guidance, and attention. Across people with MS, greater decreases in brain activation in these regions showed a statistically significant correlation with greater improvements in the 2-minute walk test. There was no observed statistically significant association between walking function and motor imagery-related brain activation changes.
Impellizzeri et al, 2020	The music therapy group showed statistically significant improvements in cognitive function (p < .001), long-term retrieval (p= 0.007), delayed recall (p=0.001), and on multiple subscales of motivation, emotional awareness, depression, and quality of life. The between-group effect versus conventional cognitive rehabilitation did not show any statistically

	significant differences (either better or worse). It should be noted that conventional cognitive rehabilitation was administered to both arms.
Kuhlmann et al, 2025	Compared to the silent condition, monochord music therapy was associated with a statistically significant reduction in psychosocial fatigue (p=0.029), increased heat pain thresholds (p=0.024) and stronger immediate subjective effects (e.g. feeling balanced: p < 0.001, relaxed: p < 0.001, less pain: p < 0.001). However, there was no statistically significant between-group difference in anxiety levels (p=0.109).
Maggio et al, 2021	The music therapy group showed significant improvements in cognitive function (p < .001), long-term retrieval (p= 0.007), delayed recall (p= 0.001), and on multiple subscales of motivation, emotional awareness, depression, and quality of life. There was no statistically significant difference compared to conventional cognitive rehabilitation (either better or worse). Conventional cognitive rehabilitation was only administered in the control arm.
Moore et al, 2008	There were no statistically significant differences between the spoken word and sung groups for recognition accuracy, false alarms, sensitivity or response bias.
Moumdjian et al, 2019	Participants were able to synchronize to the different tempi. People without cognitive impairment showed statistically significantly higher synchronization with the metronome compared with music (p < 0.001). People with MS perceived statistically significantly less physical (p < 0.0001) and cognitive fatigue (p < 0.0001) when walking to music. People with cognitive impairment perceived lower fatigue walking to music compared with metronomes (p < 0.0001).
Naderi et al, 2022	Rhythmic auditory stimulation statistically significantly improved gait symmetry and spatiotemporal parameters in MS patients. At 90% tempo, stride length, stride width, and double support duration increased, while cadence decreased. At 110% tempo, cadence increased while stride length, stride width, and double support duration decreased, indicating improved efficiency and stability. Across all tempi (90%, 100%, 110%), gait symmetry indices improved compared to walking without rhythmic auditory stimulation.
Schmid et al, 2004	Active music therapy was associated with statistically significant improvements in self-acceptance (p= 0.012), depression (p= 0.036), and anxiety (p= 0.013). However, between-group differences compared to the no intervention control group.
Seebacher et al, 2024	All music therapy groups showed statistically significant improvements in walking (primary) and broader (secondary) outcomes, but there were no statistically significant difference between the 3 cueing groups.
Seebacher et al, 2019	All music therapy interventions statistically significantly improved walking speed and distance. Physical, cognitive, and total fatigue and quality of life significantly improved only after music and verbally cued motor imagery. Synchronisation improved more with music and verbal motor imagery and music motor imagery and worsened with just motor imagery.
Seebacher et al, 2018	Feasibility was demonstrated. There were no safety-related or adverse events related to the study. Improvements in all groups for walking speed and distance, with greatest improvement in the music and verbally cued motor imagery group. Improvements in fatigue, quality of life and motor imagery ability were observed. Only one participant (in the music and verbally cued motor imagery group) showed a clinically significant improvement (defined as at least 20%) in the timed 25-foot walk test. 60% of participants in the music and verbally cued motor imagery group and 40% of participants in other groups showed a clinically significant improvement (defined as at least 20%) in the 6-minute walk test.

For peer review only

Seebacher et al, 2017	Both rhythm-cued and music-cued motor imagery statistically significantly improved walking speed, distance, perception, cognitive fatigue, and quality of life compared to no music therapy. A statistically significant improvement in physical fatigue was only found for the music-cued group.
Seebacher et al, 2015	Motor imagery with instrumental music and metronome cues statistically significantly improved walking speed and distance in intervention groups compared to no music therapy.
Seighalani et al, 2014	Compared to no intervention, music therapy significantly reduced depression and anxiety from pretest to post-test and follow-up, with effects persisting over time. Depression scores decreased markedly (39.13 to ~17), and anxiety scores halved (41.07 to ~21). Self-esteem scores improved substantially, rising from 16.00 at pretest to ~37–38 at post-test and follow-up. While differences between post-test and follow-up were minimal, all outcomes showed significant improvements compared to pretest.
Shahraki et al, 2017	Statistically significant improvements in the gait parameters stride length, time, double support time, cadence, and gait speed were found in the rhythmic auditory stimulation group compared to the control group doing the same exercises without rhythmic auditory stimulation.
Thaut et al, 2008	Music-based learning was associated with superior word order recall compared with spoken learning,(F(1,18) = 4.51, p = 0.038). Musical learning led to greater improvements in early and late learning phases, while spoken learning showed only transient gains that reduced toward the end of the task.
Thaut et al, 2014	The sung word list group showed statistically significantly more improvement in word order memory and recall (p= 0.038) and bilateral frontal alpha learning related synchronization (p= 0.05) than the spoken word group.
Wiens et al, 1999	Compared to passive music engagement, active music therapy was not shown to be associated with statistically significant improvements in the strength of respiratory muscles in people with advanced MS.
Young et al, 2019	Statistically significant improvements for music to movement versus no active music therapy or yoga were shown for in Timed Up and Go (least square mean difference [95% confidence interval] = −1.9s [−3.3 to −0.5], *P*=.01, *d*=0.7 and 6-minute walk test (41.0m [2.2-80.0], *P*=.04, *d*=0.6; controlled for Patient-Determined Disease Steps). No significant group differences were found in 5-times sit-to-stand, fatigue, and pain interference. Tests of music to movement versus control were post-hoc as the study also contained an active yoga intervention and the primary comparisons were made across music to movement, active yoga and control.

For peer review only

Supplementary file 10. Supplementary results

Results

Search results

Database searches identified a total of 5,906 records, comprising MEDLINE (n = 198), EMBASE (n = 4,029), APA PsycINFO (n = 1,155), AMED (n = 17), CINAHL (n = 107), and Cochrane Central (n = 400). In addition, 13 records were identified through supplementary methods, including citation searching. Following automatic and manual deduplication, 1,001 duplicate records were removed prior to screening. A total of 4,905 records proceeded to title and abstract screening, of which 4,871 were excluded at this stage. 34 reports were sought for retrieval from database searches, and all were successfully retrieved. 13 reports were sought for retrieval from supplementary searches; 1 duplicate was excluded at this stage, leaving 12 reports for assessment. In total, 46 reports were assessed at full text eligibility. Of these, 23 studies were included in the systematic review. 23 reports were excluded at full text screening. Reasons for exclusion included wrong publication type (n = 7), wrong outcome (n = 6), wrong intervention (n = 5), ongoing trials (n = 2), wrong population (n = 2), and duplicate records (n = 1). The final review therefore included 23 unique studies, all of which met the predefined eligibility criteria and were included in the narrative synthesis.

Study profile

The 23 studies included in this systematic review were conducted across nine countries in Europe, North America, and the Middle East, including Austria, Belgium, Canada, Germany, Iran, Italy, Switzerland, the United Kingdom, and the United States. No eligible studies were identified from Africa, South America, or Oceania. Studies were published between 1999 and 2025, reflecting sustained interest in music-based interventions for people with MS across diverse healthcare and cultural contexts.

Across all included studies, a total of 938 participants were enrolled, of whom 863 had multiple sclerosis and 75 were healthy controls. All participants receiving music-based therapy had a diagnosis of multiple sclerosis. In total, 575 participants received music-based interventions, while 295 participants were allocated to control conditions, including healthy control groups. Gender was incompletely reported; where data were available, 666 participants (75.0%) were female and 222 (25.0%) were male, consistent with the known female predominance of multiple sclerosis. Mean age was variably reported but generally fell within the mid-40s to early 50s, with overall age ranges spanning approximately 29 to 68 years. Multiple sclerosis phenotypes varied across studies and included relapsing remitting, secondary progressive, primary progressive, and mixed cohorts. Several studies enrolled participants across multiple phenotypes, while others focused on specific subgroups, most commonly relapsing remitting multiple sclerosis. Disease duration was inconsistently reported, but where available typically ranged from approximately 7 to 18 years since diagnosis, with similar durations between intervention and control groups within individual studies.

Baseline disability levels were most commonly reported using the Expanded Disability Status Scale (EDSS). Across studies reporting EDSS, participants generally had mild to moderate disability, with median or mean scores most frequently ranging between 2.0 and 4.5. A small number of studies included participants with higher levels of disability; one study involving individuals with advanced disease reported mean EDSS scores

For peer review only - http://bmjopen.bmj.com/site/about/guidelines.xhtml

above 7 and included only wheelchair users. Several trials explicitly permitted the use of walking aids during interventions and assessments, including unilateral or bilateral aids, while others restricted participation to individuals able to ambulate independently.

Handedness was rarely reported, but where specified, participants were predominantly right-handed, particularly in studies involving music assisted learning paradigms. Only one study explicitly reported the inclusion of left-handed participants.

The majority of included studies employed experimental designs, most commonly randomised controlled trials (RCT). Overall, 15 studies were RCT, alongside three quasi-experimental studies, two matched or non-randomised controlled studies, one observational cross-sectional study, one case control study, and one secondary analysis of a randomised trial. Sample sizes were generally modest, reflecting the exploratory nature of much of the literature.

Music based interventions were heterogeneous in content, delivery, and duration, ranging from single session experimental paradigms to multi week programmes lasting up to 12 weeks. Interventions were delivered in both health facility and home-based settings, led by music therapists, physiotherapists, multidisciplinary teams, researchers, or self-directed by participants. No studies directly compared different music-based intervention types with each other.

The MBT intervention types covered (see Supplemental file 7 for study-level details) are the Nordoff Robins approach (active music therapy - Music-making on instruments, or singing), rhythmic auditory stimulation, playing the keyboard with numbered, coloured stickers to guide, musically- and or-rhythmically-based cueing, neurologic music therapy, listening to a monochord being played while lying on top of it and feeling the vibration, listening to music while walking on a treadmill, hearing word lists in song format and being asked to sing them back, active music therapy (discussion about music, making music vocally, with mouth and/or with instruments), passive music therapy (listening to music), Ray's Auditory Verbal Learning Test – sung word lists, music therapy focusing on singing and breathing, and music to movement. No studies assessed choral singing or community singing groups.

Narrative synthesis

23 studies were included and synthesised narratively due to substantial heterogeneity in intervention content, delivery, duration, settings, study design, and outcome measurements. Music based interventions included a wide range of approaches, including rhythmic auditory stimulation (RAS), neurologic music therapy, music cued motor imagery, active music making, music assisted learning, and receptive listening-based interventions. Interventions were delivered in both health facility and home settings, led by music therapists, physiotherapists, multidisciplinary teams, researchers, or delivered independently by participants.

Motor outcomes

Motor outcomes were assessed in 16 studies and most commonly focused on gait and mobility. RAS was evaluated in several controlled and quasi-experimental studies delivered primarily in health facility settings. Conklyn et al (2010) conducted a RCT in which participants self-

For peer review only

administered RAS at home for 20 minutes twice daily over four weeks and reported statistically significant improvements between groups in double support time, and within group improvements in cadence, stride length, step length, and walking velocity. Similarly, Shahraki et al (2017) reported statistically significant improvements across multiple gait parameters, including stride length, stride time, cadence, double support time, and gait speed, in a RAS group compared with a control group performing the same exercises without auditory cueing.

Naderi et al (2022) examined tempo dependent effects of RAS in a single session laboratory-based study and reported improvements in gait symmetry indices across all tempi compared with uncued walking, with differential changes in cadence, stride length, and double support duration suggesting improved gait efficiency and stability. Moumdjian et al (2019) compared music and metronome cueing in a controlled laboratory setting and demonstrated that participants were able to synchronise to both stimuli, with statistically significantly lower perceived physical and cognitive fatigue reported when walking to music, particularly among participants without cognitive impairment.

A substantial proportion of the motor evidence base consisted of a programme of randomised controlled trials by Seebacher and colleagues, delivered predominantly in home-based settings using self-guided music or rhythm cued motor imagery. Across multiple studies (Seebacher et al, 2015, 2017, 2018, 2019, and 2024), music cued and rhythm cued motor imagery interventions were associated with statistically significant improvements in walking speed and walking distance compared with no intervention. Seebacher et al (2017) reported improvements in walking speed, distance, walking perception, cognitive fatigue, and QoL in both music cued and rhythm cued motor imagery groups, with physical fatigue improving only in the music cued group. Seebacher et al (2019) reported that improvements in fatigue and QoL occurred only following music and verbally cued motor imagery, not non cued motor imagery. In a larger multi arm trial, Seebacher et al (2024) reported statistically significant improvements in walking and secondary outcomes across all cueing approaches, with no statistically significant differences between cueing modalities.

Other studies explored gait related outcomes using alternative music-based approaches. Maggio et al (2021) investigated treadmill walking with music listening and reported improvements in balance, mobility, and walking speed alongside psychosocial outcomes. Young et al (2019) evaluated a music to movement intervention delivered in a fitness facility and reported statistically significant improvements in Timed Up and Go and six-minute walk test compared with control, while no statistically significant differences observed for five times sit to stand, fatigue, or pain interference.

Motor outcomes beyond gait were less frequently assessed. Gatti et al (2015) evaluated a structured keyboard playing intervention delivered by a physiotherapist and reported a statistically significant improvement in perceived hand functional use compared with control, while improvements in hand dexterity occurred in both groups without statistical significance between group differences. Goetschalckx et al (2021) examined bilateral gait coordination in a cross-sectional laboratory study and reported poorer coordination at lower metronome frequencies, alongside statistically significant associations between coordination deficits, cognitive performance, and walking capacity. Respiratory muscle strength was evaluated in a single study by Wiens et al (1999), which found no statistically significant differences between active singing-based music therapy and passive music engagement in people with advanced MS. Helminger et al (2025) reported that only participants classified as responders

For peer review only

demonstrated statistically significant improvements in two minute walk test distance, with correlated reductions in premotor and attentional brain activation.

Cognitive outcomes

Cognitive outcomes were assessed in eight studies and included global cognition, learning and memory, and executive function or attention. Two RCTs by Thaut et al (2008) and (2014) and examined music assisted learning using sung word lists delivered in single session laboratory paradigms. Both studies reported better word order recall and learning performance in musical conditions compared with spoken conditions, with Thaut et al (2014) additionally demonstrating increased bilateral frontal alpha learning related synchronisation measured using EEG.

Moore et al (2008) evaluated verbal learning and executive function using signal detection metrics and reported no statistically significant differences between spoken and sung learning conditions, suggesting that effects of musical structure may vary by task and outcome measure. Impellizzeri et al (2020) conducted a randomised controlled trial of neurologic music therapy combined with conventional cognitive rehabilitation and reported statistically significant within group improvements in cognitive function, memory, motivation, emotional awareness, depression, and QoL; however, no statistically significant between group differences were observed when compared with conventional cognitive rehabilitation alone. Maggio et al (2021) reported similar within group improvements across cognitive and psychosocial outcomes, with no clear superiority over conventional cognitive rehabilitation.

Cognitive outcomes were also incorporated as secondary measures in motor focused trials. Goetschalckx et al (2021) reported statistically significant correlations between interlimb coordination and cognitive performance across tempo conditions, while Seebacher et al (2024) included global cognitive impairment as a secondary outcome and reported improvements across intervention arms without differences between cueing modalities. Overall, results for cognitive outcomes were mixed. Studies that used music specifically to support learning tasks tended to show clearer benefits, whereas studies that used music as part of wider cognitive rehabilitation programmes showed less consistent advantages.

Psychological outcomes

Seven studies assessed psychological outcomes, most commonly depression and anxiety, with additional measures of self-acceptance, self-esteem, emotional processing, and body perception. Aldridge et al (2005) evaluated a Nordoff Robbins based active music therapy programme delivered by a therapist in a health facility and reported no statistically significant differences between groups, but medium effect sizes favouring music therapy for self-esteem, depression, and anxiety, alongside significant within group improvements during the intervention period and deterioration following cessation.

Schmid et al (2004) reported statistically significant improvements in self-acceptance, depression, and anxiety following active music therapy delivered over multiple blocks, although between group differences relative to a matched control group were not clearly demonstrated. Seighalani et al (2014) reported marked and sustained reductions in depression and anxiety and substantial improvements in self-esteem following a combination of active and passive music therapy compared with no intervention. Kuhlmann et al (2025) evaluated a receptive monochord based

For peer review only

intervention and reported statistically significant reductions in psychosocial fatigue, increased pain thresholds, and positive subjective effects, although no statistically significant difference between groups were observed for anxiety.

Psychological outcomes were also reported as secondary outcomes in trials primarily focused on cognition or mobility. Impellizzeri et al (2020) and Maggio et al (2021) reported improvements in depression and emotional awareness within music therapy groups without statistically significant superiority over comparator interventions. Seebacher et al (2024) included anxiety, depression, and suicidality as secondary outcomes and reported improvements across groups with no differences between cueing approaches.

Quality of life outcomes

QoL related outcomes were assessed in 12 studies using MS specific instruments, often alongside fatigue and disability measures. Improvements in quality of life were reported in several interventions, particularly those combining music cueing with motor imagery. Seebacher et al (2017) reported improvements in quality of life and cognitive fatigue following both music cued and rhythm cued motor imagery compared with no intervention, with physical fatigue improving only in the music cued group. Seebacher et al (2018) and (2019) similarly reported improvements in fatigue and quality of life following music-based cueing, while Seebacher et al (2024) reported improvements across groups without differences between cueing modalities.

Fatigue was one of the most commonly assessed non motor outcomes and included physical, cognitive, and psychosocial components. Moumdjian et al (2019) reported statistically significantly lower perceived physical and cognitive fatigue during walking to music compared with metronome cues. Kuhlmann et al (2025) reported reductions in psychosocial fatigue following monochord-based music therapy. Aldridge et al (2005) observed deterioration in fatigue during follow up after cessation of therapy despite improvements during the intervention period. Pain outcomes were less consistently reported. Kuhlmann et al, 2025 reported increased heat pain thresholds and subjective pain reductions, whereas Young et al, 2019 and Conklyn et al, 2010 reported no statistically significant between group differences for pain related outcomes.

Risk of bias

Risk of bias and methodological quality were assessed using the Critical Appraisal Skills Programme (CASP) checklists, selected according to study design (randomised controlled trials, cohort studies, case control studies, and cross-sectional studies). CASP was chosen to provide a structured, domain-based assessment of internal validity, applicability, and reporting quality, rather than a single summary score. Although numerical scores and quality categories (low, medium, high) were derived for transparency, interpretation focused on domain level patterns of bias, consistent with recommendations to avoid over reliance on composite quality scores.

Across the 23 included studies, the RCT checklist was the most frequently applied, reflecting that most studies were experimental in design. Where previous reviews had inconsistently labelled some studies as non-randomised, quasi experimental, or clinical trials, the present review applied CASP checklists based on the actual methodological features reported, rather than labels used in earlier syntheses. This resulted in three

For peer review only

intentional mismatches between study labels used in prior reviews and the CASP checklist applied here. For example, Maggio et al (2021) and Naderi et al (2022) were assessed using the RCT checklist despite being described as quasi experimental in some summaries, as both involved prospective intervention allocation and comparator groups. Conversely, Aldridge et al (2005) and Schmid et al (2004) were assessed using the cohort checklist, as allocation was matched rather than randomised. These decisions were made to ensure the most appropriate appraisal tool was applied to each study's actual design.

Overall, most studies addressed a clearly focused research question, reported outcomes comprehensively, and used validated outcome measures. Baseline characteristics were generally comparable between intervention and control groups where applicable. However, several recurring sources of bias were identified across studies. Blinding of participants and intervention providers was rarely possible, reflecting the nature of music-based interventions. While some studies reported assessor blinding, this was inconsistently applied or insufficiently described. Allocation concealment was rarely reported and may represent either a reporting limitation or a true methodological weakness.

Sample size was a common concern. Many studies were small, exploratory, or pilot in nature, limiting statistical power and increasing the risk of type II error. Participant flow and attrition were not always clearly reported, particularly in older studies and non-randomised designs, raising potential concerns regarding selection and attrition bias. Although most studies reported statistically significant findings where present, clinical significance was rarely addressed, and confidence intervals or precision estimates were inconsistently reported.

Observational and non-randomised studies generally demonstrated appropriate measurement of exposure and outcomes, but often relied on convenience sampling, increasing the risk of selection bias. Cross sectional and case control designs were limited in their ability to account for confounding, although the case control study assessed using CASP demonstrated strong internal consistency across domains. Cohort studies scored highly overall but lacked detailed reporting of precision and effect estimates.

Several important limitations were identified that are not fully captured by CASP or other standard risk of bias tools. These include heterogeneity in intervention content, duration, and delivery personnel; variability in outcome measures across studies; frequent underrepresentation of male participants; and reliance on self-reported outcomes in several domains. In addition, some single session experimental studies lacked a comparator capable of controlling for non-specific effects such as attention, novelty, or participant expectation.

Despite these limitations, higher quality RCTs demonstrated generally good internal validity, clear outcome reporting, and appropriate analytical approaches. Studies rated as high quality were more likely to include blinded outcome assessment, clearer reporting of statistical methods, and better alignment between results and conclusions. Across study designs, conflicts of interest were not identified as a major concern, and authors typically acknowledged at least some methodological limitations.

Overall, the risk of bias profile suggests that while many studies were thoughtfully designed and conducted within the practical constraints of arts-based research, methodological limitations were common, particularly relating to blinding, sample size, and reporting transparency. These factors should be considered when interpreting the strength and consistency of the evidence base.

Supplementary file 11. CASP risk of bias checklists

For peer review only

Part A: RCT checklist

Study	Clear research question?	Randomised?	All participants accounted for?	Participants blinded?	Investigators blinded?	Assessors blinded?	Similar baselines?
Conklyn et al, 2010	Yes	Yes	Yes	No	No	No	Yes
Gatti et al, 2015	Yes	Yes	Yes	No	No	No	Yes
Helminger et al. 2025	Yes	Yes	Yes	No	No	No	Yes
Impellizzeri et al, 2020	Yes	Yes	Yes	Yes	No	No	Yes
Kuhlmann et al, 2025	Yes	Yes	No	No	Yes	No	Yes
Maggio et al. 2021	Yes	No	Yes	No	No	No	Yes
Moore KS. et al. 2008	Yes	Yes	Yes	No	No	No	Yes
Naderi S. Et al. 2022	Yes	No	Yes	No	No	No	Yes
Seebacher et al, 2015	Yes	Yes	Yes	No	No	No	Yes
Seebacher et al, 2017	Yes	Yes	Yes	No	No	No	Yes
Seebacher et al, 2018	Yes	Yes	Yes	No	No	No	Yes
Seebacher et al, 2019	Yes	Yes	Yes	No	No	No	Yes
Seebacher et al, 2024	Yes	Yes	Yes	Yes	Yes	No	Yes
Seighalani et al, 2014	Yes	Yes	Yes	No	No	No	Yes
Shakraki et al, 2017	Yes	Yes	Yes	No	No	No	Yes
Thaut et al, 2008	Yes	yes	Yes	No	No	No	Yes
Thaut et al, 2014	Yes	Yes	Yes	No	Yes	No	Yes
Wiens et al, 1999	Yes	Yes	Yes	No	No	No	Yes
Young et al, 2019	Yes	Yes	Yes	No	No	Yes	Yes

For peer review only

Study	Same level of care?*	Effects of intervention reported comprehensively?	Precision of treatment effect reported?	Benefits outweigh harms and costs?	Applicable to our context?	Greater value than existing treatment?	Score	Quality
Conklyn et al, 2010	Yes	Yes	No	Yes	Yes	Can't tell	17	Low
Gatti et al, 2015	Yes	Yes	Yes	Yes	Yes	Can't tell	19	Medium
Helminger et al. 2025	Yes	Yes	Yes	Yes	Yes	Can't tell	19	Medium
Impellizzeri et al, 2020	Yes	Yes	Yes	Yes	Yes	Can't tell	21	High
Kuhlmann et al, 2025	Yes	Yes	Yes	Yes	Yes	Can't tell	19	Medium
Maggio et al. 2021	Yes	Yes	No	Yes	Yes	Can't tell	15	Low
Moore. et al. 2008	Yes	Yes	No	Yes	Yes	Can't tell	17	Low
Naderi. et al. 2022	Yes	Yes	No	Yes	Yes	Can't tell	15	Low
Seebacher et al, 2015	Yes	Yes	Yes	Yes	Yes	Can't tell	19	Medium
Seebacher et al, 2017	Yes	Yes	Yes	Yes	Yes	Can't tell	19	Medium
Seebacher et al, 2018	Yes	Yes	Yes	Yes	Yes	Can't tell	19	Medium
Seebacher et al, 2019	Yes	Yes	Yes	Yes	Yes	Can't tell	19	Medium
Seebacher et al, 2024	Yes	Yes	Yes	Yes	Yes	Can't tell	23	High
Seighalani et al, 2014	Yes	Yes	No	Yes	Yes	Can't tell	17	Low
Shakraki et al, 2017	Yes	Yes	No	Yes	Yes	Can't tell	17	Low
Thaut et al, 2008	Yes	Yes	No	Yes	Yes	Can't tell	17	Low

For peer review only

Thaut et al, 2014	Yes	Yes	No	Yes	Yes	Can't tell	19	Medium
Wiens et al, 1999	Yes	Yes	No	Yes	Yes	Can't tell	17	Low
Young et al, 2019	Yes	Yes	Yes	Yes	Yes	Can't tell	21	High

* = apart from intervention

Part B: Cross-sectional checklist

**Study**	**Clear research question?**	**Appropriate method?**	**Acceptable recruitment?**	**Accurate measurement to reduce bias?**	**Data collected in a way that addressed research issue?**	**Enough participants?**	**Clear results presentation?**
Goetschalckx et al, 2021	Yes	Yes	Yes	Yes	Yes	Can't tell	Yes

**Study**	**Rigorous analysis?**	**Clear statement of findings?**	**Applicable to local population?**	**How valuable is the research?**	**Score**	**Quality**
Goetschalckx et al, 2021	Yes	Yes	Yes	Can't tell	20	High

Part C: Case control checklist

**Study**	**Clearly focused issue?**	**Appropriate method?**	**Acceptable recruitment?**	**Acceptable control selection?**	**Exposure accurately measured to minimise bias?**	**Groups similar apart from exposure?**	**Confounding taken into account in design and analysis?**
Moumdjian et al, 2019	Yes	Yes	Yes	Yes	Yes	Yes	Yes

**Study**	**Large treatment effect?**	**Precise estimate of treatment effect?**	**Do you believe the results?**	**Applicable to our context?**	**Results fit with other available evidence?**	**Score**	**Quality**
Moumdjian et al, 2019	Can't tell	Can't tell	Yes	Yes	Yes	18	High

For peer review only

Part D: Cohort checklist

**Study**	**Clearly focused issue?**	**Acceptable recruitment?**	**Exposure accurately measured to minimise bias?**	**Outcome accurately measured to minimise bias?**	**All important confounders identified?**	**Confounding taken into account in design and analysis?**	**Follow-up complete enough?**
Aldridge et al. 2005	Yes	Yes	Yes	Yes	Yes	Yes	Yes
Schmid et al, 2004	Yes	Yes	Yes	Yes	Yes	Yes	Yes

**Study**	**Clear results presentation?**	**How precise are the results?**	**Do you believe the results?**	**Applicable to local population?**	**Results fit with other available evidence?**	**Clear implications for practice?**	**Score**	**Quality**
Aldridge et al. 2005	Yes	Can't tell	Yes	Yes	Yes	Can't tell	26	High
Schmid et al, 2004	Can't tell	No	Yes	Yes	Yes	Can't tell	24	High

For peer review only

Supplementary file 12. Unabridged discussion

Summary

This systematic review synthesised evidence on MBT for rehabilitation outcomes in people with multiple sclerosis (MS), identifying 23 eligible studies published between 1999 and 2025, of which two[30,40] had not been included in any of the previous reviews on this topic. Furthermore, no single previous review[14,15] had included more than 12 of the 23 studies (52%) we identified. Furthermore, the present review adds an assessment of clinical significance and certainty of the findings. Across studies reporting gender, participants were predominantly female (75%), broadly consistent with MS epidemiology. Most samples represented mild to moderate disability, with EDSS values most commonly between 2.0 and 4.5, although one study focused on advanced MS with wheelchair users[19].

Across the 23 studies, outcomes mapped to four rehabilitation domains, with motor outcomes most frequently assessed (16 studies), followed by quality of life and related outcomes (12 studies), cognitive outcomes (8 studies), and psychological outcomes (7 studies). Motor findings were most consistent for gait and mobility, particularly where interventions used rhythmic auditory stimulation and music cued motor imagery, with improvements reported in walking speed, walking distance, cadence, stride length, and gait symmetry. Cognitive findings were mixed, with clearer benefits in music assisted learning paradigms (eg. sung versus spoken word learning), while broader cognitive rehabilitation comparisons suggested similar rather than superior benefits relative to conventional rehabilitation. Psychological outcomes showed potential improvements in depression, anxiety, self-acceptance and self-esteem, but between group effects were not uniform. Quality of life and fatigue were frequently co assessed, with several trials reporting improvements in fatigue and MS specific quality of life measures, though findings were heterogeneous.

Importantly, this review incorporated studies that have been less emphasised in prior summaries and were necessary to include for a complete synthesis. Seighalani et al (2014) provides controlled evidence of sustained improvements in depression, anxiety and self-esteem after a combined active and receptive intervention, strengthening the psychological signal beyond the frequently cited early matched studies[40]. Kuhlmann et al (2025) adds contemporary randomised evidence for receptive monochord-based therapy, extending the evidence base into pain perception, body perception, and psychosocial fatigue outcomes[30]. The review focused specifically on MBT interventions in MS and excluded non peer reviewed sources such as conference abstracts and chapters, improving interpretability at the cost of excluding some early-stage evidence. Challenges were identified in terms of certainty of evidence as assessed by GRADE and reaching clinical significance thresholds across the sample for some outcomes. These may reflect the emerging nature of the evidence base for MBTs in MS, and the arts in health more generally, and the methodological challenges associated therewith. In particular, considerable heterogeneity was observed in terms of intervention details, sample, and outcome measure assessment. Some studies were assessed as low quality, reflecting these methodological challenges. Furthermore, it is important to reflect whether medically-oriented certainty assessments such as GRADE are a good match for complex interventions, including the arts, and what alternatives may be available.

For peer review only

Interpretation of findings

This review extends and updates the MS rehabilitation literature on MBT by both increasing the number of included evidence and broadening the scope of interventions and outcomes compared with prior syntheses. In particular, Lopes et al (2021) selected 10 clinical trials from 282 records, clustering MBT into rhythmic auditory, instrument playing, dance strategy, and neurologic music therapy approaches[13]. In contrast, the present review included 23 studies, representing an additional 13 studies beyond that earlier rehabilitation focused review, as well as a longer publication window (1999 to 2025). This broader capture is clinically useful because it incorporates both single session mechanistic models (such as learning and synchronisation studies) and multi week home and facility programmes that more closely resemble real world rehabilitation delivery.

A key interpretive issue is the distinction between active and receptive MBT. The evidence base remains weighted toward receptive approaches, particularly music listening during walking or cueing based strategies. Phillips et al (2025) similarly concluded that most included interventions were receptive, with fewer active interventions, and highlighted gait parameters and fatigue as the most common outcomes. The findings in the present review support that pattern, but also suggest that the active versus receptive categorisation may be too simplistic for MS rehabilitation[15]. Several interventions combine elements of both, for example music cued motor imagery or active music making that targets motor control and dexterity rather than expressive goals. The most consistent motor improvements were observed in cueing based studies, where music may provide external temporal structure that supports auditory motor coupling and gait automaticity, aligning with broader neurological rehabilitation theory.

This review also differs from Phillips et al (2025) in scope and included studies. Phillips et al searched from 2002 to June 2024 and included 15 studies[15]. By design, that excludes earlier work such as the 1999 singing and respiratory intervention trial[19], and it does not capture more recent 2025 studies. The inclusion here of Kuhlmann et al (2025) provides additional randomised evidence on receptive vibration based therapy effects on fatigue and pain perception[30], while Helmlinger et al (2025) contributes neuroimaging correlated outcomes that are not represented in reviews with earlier cut offs[29]. Including these newer studies is advantageous because it shows how the field is moving beyond gait outcomes alone and going toward mechanistic markers and broader symptom domains.

It is notable that, compared with Parkinson's disease (PD), there is a relative lack of research on singing based interventions in MS. In contrast to the extensive evidence base for singing in PD, as synthesised by Barnish et al. (2025)[8], singing interventions in MS were absent or only minimally represented, highlighting a clear gap for future research.

Across outcomes, the pattern of benefit appears strongest and most consistent for gait and mobility, with more heterogeneous findings for cognition and psychological outcomes. This heterogeneity likely reflects real differences in intervention intent and dose, diversity in comparators, and inconsistent outcome measures. Overall, MBT appears promising across multiple rehabilitation domains in MS, but the evidence remains constrained by intervention heterogeneity, limited head to head comparisons of MBT types, and variable methodological reporting, which together limit the certainty of any single conclusion.

Strengths and limitations

This systematic review has several strengths. A comprehensive and transparent search strategy was employed across multiple databases, with the final search conducted in July 2025, within six months of manuscript submission, in line with best practice guidance for systematic reviews. The review adhered to PRISMA[21] reporting standards and applied predefined eligibility criteria, reducing the risk of selection bias. Data extraction was undertaken using standardised forms, which helped ensure consistency in capturing study characteristics, intervention details, and outcomes across heterogeneous study designs.

A key methodological strength is the exclusive use of CASP risk of bias tools[22], selected according to study design. All assessments were drawn from the same CASP suite, ensuring conceptual consistency across randomised controlled trials, cohort studies, case-control studies, and cross-sectional studies. This approach avoided the methodological fragmentation that can arise when multiple unrelated appraisal tools are combined. Where inconsistencies existed in how previous systematic reviews labelled study designs, studies were appraised based on their actual methodological features rather than historical labels, ensuring the most appropriate CASP checklist[22] was applied. This strengthened the internal coherence of the risk of bias assessment and improved transparency.

However, our limitations should be acknowledged. Although many included studies were randomised, blinding of participants and intervention providers was rarely possible due to the nature of music-based interventions, increasing the potential for performance bias. Sample sizes were frequently small, and several studies were exploratory or pilot in nature, limiting the statistical power. Reporting of participant flow, allocation concealment, and precision estimates was inconsistent, particularly in older studies and non-randomised designs.

Limitations of the evidence base also restrict interpretation. There was substantial heterogeneity in intervention content, duration, delivery setting, and comparator conditions, precluding meta-analysis. Outcome measures varied widely, even within the same rehabilitation domain, limiting cross-study comparability. Male participants were consistently underrepresented, reflecting broader challenges in MS rehabilitation research. Finally, the review focused on peer-reviewed literature and excluded conference abstracts and non-peer-reviewed sources, improving methodological robustness but potentially omitting emerging evidence.

Implications for research and practice

Future research should prioritise addressing the methodological limitations identified in this review. Larger, adequately powered randomised controlled trials are needed, with clearer reporting of allocation concealment, participant flow, and effect size precision. Greater standardisation of outcome measures, particularly for gait, fatigue, cognition, and quality of life, would enhance comparability and enable future quantitative synthesis. Reporting of clinically meaningful change, alongside statistical significance, should be prioritised to support translation into practice.

There is a clear need for studies that directly compare different music-based intervention approaches, including active versus receptive therapies. The relative lack of research on singing-based interventions in MS represents a notable gap, particularly given the evidence base for singing in Parkinson's disease[8]. Future studies

For peer review only

should also expand beyond gait-focused outcomes to include communication, social participation, and patient-prioritised outcomes.

From a clinical perspective, the current evidence base supports cautious integration of MBT, particularly rhythmic cueing and music-cued motor imagery, within MS rehabilitation programmes. These interventions appear feasible in both facility and home-based settings and may complement conventional physiotherapy and cognitive rehabilitation. However, given heterogeneity and residual risk of bias, music-based therapy should currently be considered an adjunct rather than a replacement for established rehabilitation approaches.

Conclusion

This systematic review synthesised evidence from 23 studies examining the effects of music-based therapy on motor, cognitive, psychological, and quality of life outcomes in people with multiple sclerosis. The narrative synthesis suggests that music-based interventions, particularly rhythmic auditory stimulation and music-cued motor imagery, may suggest benefits for gait and mobility, with more variable evidence for cognitive, psychological, and quality of life outcomes. Evidence for cognitive benefit was strongest in music-assisted learning paradigms, while psychological and fatigue-related outcomes showed mixed but promising signals.

The review extends prior rehabilitation-focused syntheses by including a broader range of intervention types, outcome domains, and recent studies, while maintaining methodological consistency through exclusive use of CASP risk of bias tools. Nonetheless, heterogeneity in interventions, outcomes, and study design limits the strength of conclusions. Overall, music-based therapy appears to be a promising adjunct in MS rehabilitation, but higher-quality, standardised research is required to clarify optimal intervention models and establish clinical effectiveness.

For peer review only

BMJ Open

Potential benefits of music-based therapies on rehabilitation outcomes in people with multiple sclerosis: a systematic review

Journal:	*BMJ Open*
Manuscript ID	bmjopen-2026-116420.R1
Article Type:	Original research
Date Submitted by the Author:	05-Jun-2026
Complete List of Authors:	Ahadi, Nicky ; University of Exeter, University of Exeter Medical School<br>Kim, Jeeyeon ; University of Michigan, Department of Physical Medicine and Rehabilitation<br>Whibley, Daniel; University of Michigan, Department of Physical Medicine and Rehabilitation; University of Michigan, Center for History, Humanities, Arts, Social Sciences, and Ethics in Medicine<br>Barnish, Maxwell; University of Exeter, Peninsula Technology Assessment Group (PenTAG), School of Public Health and Sport Sciences, University of Exeter Medical School
**Primary Subject Heading**:	Public health
Secondary Subject Heading:	Neurology
Keywords:	Multiple sclerosis < NEUROLOGY, COMPLEMENTARY MEDICINE, PUBLIC HEALTH

I, the Submitting Author has the right to grant and does grant on behalf of all authors of the Work (as defined in the below author licence), an exclusive licence and/or a non-exclusive licence for contributions from authors who are: i) UK Crown employees; ii) where BMJ has agreed a CC-BY licence shall apply, and/or iii) in accordance with the terms applicable for US Federal Government officers or employees acting as part of their official duties; on a worldwide, perpetual, irrevocable, royalty-free basis to BMJ Publishing Group Ltd ("BMJ") its licensees and where the relevant Journal is co-owned by BMJ to the co-owners of the Journal, to publish the Work in this journal and any other BMJ products and to exploit all rights, as set out in our licence.

The Submitting Author accepts and understands that any supply made under these terms is made by BMJ to the Submitting Author unless you are acting as an employee on behalf of your employer or a postgraduate student of an affiliated institution which is paying any applicable article publishing charge ("APC") for Open Access articles. Where the Submitting Author wishes to make the Work available on an Open Access basis (and intends to pay the relevant APC), the terms of reuse of such Open Access shall be governed by a Creative Commons licence – details of these licences and which Creative Commons licence will apply to this Work are set out in our licence referred to above.

Other than as permitted in any relevant BMJ Author's Self Archiving Policies, I confirm this Work has not been accepted for publication elsewhere, is not being considered for publication elsewhere and does not duplicate material already published. I confirm all authors consent to publication of this Work and authorise the granting of this licence.

For peer review only

Potential benefits of music-based therapies on rehabilitation outcomes in people with multiple sclerosis: a systematic review

Nicky Ahadi[1], Jeeyeon Kim[2], Daniel Whibley[2,3], Maxwell S. Barnish[4]

1 University of Exeter Medical School, University of Exeter, Exeter, UK

2 Department of Physical Medicine and Rehabilitation, University of Michigan, Ann Arbor, USA

3 Center for History, Humanities, Arts, Social Sciences, and Ethics in Medicine, University of Michigan, Ann Arbor, USA

4 Peninsula Technology Assessment Group (PenTAG), School of Public Health and Sport Sciences, University of Exeter Medical School, University of Exeter, Exeter, UK

Correspondence to: Dr Maxwell S. Barnish; m.s.barnish@exeter.ac.uk

Keywords: Multiple sclerosis; Music therapy; Music-based therapies

For peer review only

ABSTRACT

Objectives. To assess the evidence for a benefit of music-based therapies (MBTs) on rehabilitation outcomes in adults with multiple sclerosis (MS).

Setting. Scholarly literature (published in English) from any country. This systematic review received no specific funding and was registered on PROSPERO as CRD420251083656.

Data sources. Six bibliographic databases: AMED (Ebsco), APA PsycINFO (Ovid), CINAHL (Ebsco), Cochrane CENTRAL, EMBASE (Ovid) and MEDLINE (Ovid), plus supplementary searches and citation chasing (search date July 2025).

Primary and secondary outcome measures. Eligible studies used a clinical trial or controlled quantitative observational study design to assess the benefit of MBTs on rehabilitation outcomes in adults with MS. Risk of bias was assessed using CASP checklists. Data were synthesised using narrative synthesis.

Results. 23 studies were included (955 participants, of whom 880 people had MS and 691 received MBTs). The most frequently assessed outcome was motor function (16 studies), followed by quality of life (12 studies). Evidence suggests potential benefits for MBTs, particularly rhythmic auditory stimulation and music-cued motor imagery, on gait and mobility outcomes, including walking speed, walking distance, cadence, stride length, and gait symmetry. Findings for other outcomes were less clear.

Conclusions. MBTs may benefit people with MS, with the most consistent evidence observed for gait and mobility outcomes. However, heterogeneity in intervention type, study design, and outcome measurement limits the strength of conclusions. Higher-quality, adequately powered trials with standardised outcomes and direct comparisons between MBTs are needed to clarify effectiveness and inform clinical implementation.

STRENGTHS AND LIMITATIONS OF THIS STUDY

- Systematic review methods minimised subjectivity and bias.
- A structured outcome set was used.
- An independent dual review was conducted.
- Patient and public involvement was not conducted.
- Only English-language studies could be included.

For peer review only

INTRODUCTION

Multiple sclerosis

Multiple sclerosis (MS) is a chronic inflammatory and neurodegenerative disease of the central nervous system and a leading cause of neurological disability in young adults.[1,2] It affects more than 2.8 million people worldwide and its prevalence has increased substantially in many regions, with the highest rates reported in North America, western Europe and Australasia.[1-3] This rise is attributed to a combination of greater life expectancy, population growth, and better access to diagnostic services.[2,3] Common manifestations include optic neuritis, sensory disturbance, limb weakness and gait ataxia as any site in the central nervous system can be affected.[1-3] MS is usually described as having three main types with Relapsing Remitting MS (RRMS) being the most common, affecting around 85-90% of people with MS. Primary progressive MS (PPMS) accounts for about 10-15% and progresses steadily without relapses. Over time, many with RRMS develop secondary progressive MS (SPMS), where disability gradually increases.[1]

Treatment options for MS

Disease modifying therapies can reduce relapse frequency and delay disability progression, yet they do not fully address the multifactorial physical, cognitive, and emotional consequences of living with a chronic, unpredictable condition. MS places a heavy burden on health systems and carries high economic costs.[2,3] As symptoms often persist despite medication, there is increasing interest in non-pharmacological approaches to support holistic rehabilitation and improve quality of life (QoL).[3,4] These approaches include arts-based interventions, which have been shown to be promising therapeutic approaches in anxiety and depression[5], palliative care[6], stroke[7] and Parkinson's disease[8-10]. Within this broad arts sphere, one focal point of therapeutic interest is the use of music for therapeutic benefit.

The concept of Music-Based Therapies

A range of different approaches have been taken to conceptualising music interventions for therapeutic benefit. One established concept is music therapy (MT). The American Music Therapy Association defines MT as "the clinical and evidence-based use of music interventions to accomplish individualized goals within a therapeutic relationship by a credentialed professional who has completed an approved music therapy program".[11] However, definitions of MT vary greatly internationally, making the concept challenging to interpret in the international literature. The UK, USA and Canada take a highly formalised approach to MT using protected, clinically based legal definitions, requiring specific MT qualifications and registration with a statutory body.[11-13] Meanwhile, the German Music Therapy Society focuses on the "targeted use of music" to restore, maintain and promote mental, physical and cognitive health",[14] the Norwegian approach focuses on "community music therapy", combining individual clinical work with social, community-based approaches,[15-16] and the Chinese approach incorporates theories from traditional Chinese medicine (TCM), such as the 'five tones' model, alongside western MT approaches.[17] Areas of between-country variation include whether statutory registration is required, whether the focus is on active or receptive MT approaches, and the extent to which MT is clinically focused and conducted individually or socially focused and conducted in groups. Therefore, MT does not have the same meaning in different countries - interventions that would be described as MT in some countries would not be permitted to be described as MT in other countries.[14] Pragmatic definitions of MT have been used by some researchers[5,8,9] to overcome this lack of a universal definition of MT, however the misalignment of these definitions with those used by statutory bodies may be problematic.

For peer review only

Noting these issues defining MT in a consistent and meaningful way for international research, broader concepts have been proposed that include interventions that may be beneficial but need not be delivered by a certified music therapist. The broadest alternative conceptualisation of the use of music for health benefit is “musical care” (MC) – a concept developed by Spiro and Sanfilippo.[18] MC is defined as “the role of music—music listening as well as music-making—in supporting any aspect of people's developmental or health needs: for example, physical and mental health, cognitive and behavioral development, and interpersonal relationships”.[18] The authors state[18] that MC encompasses MT but goes beyond MT and includes a wide range of musically based interventions. The concept of MC considers the role of music in its broadest sense and does not only consider structured interventions, nor does it only consider interventions that are therapeutic – MC includes unstructured activities that include music and interventions where music is not the primary therapeutic modality.[18] This may be considered a limitation of the MC concept.

Therefore, the concept of music-based therapies (MBTs) has been proposed, as well as the alternative term music-based interventions (MBIs). As these terms are synonymous, we shall use the term MBTs consistently. The concept of MBTs has been used in several previous systematic reviews in neurological conditions.[19,20,21] Lin et al[19] delineate their review in the full text as assessing MBTs, although the term MT is used in the article title. However, a common limitation in previous reviews using the MBTs concept across different health conditions has been a lack of an explicit definition of MBTs. The key distinction between MBTs and music therapy is that while MBTs are designed to be therapeutic and often to enhance general wellbeing or provide short-term symptom relief, they need not be clinical interventions delivered by a certified music therapist.[22] Based on the work of Raglio,[22] we propose the following explicit operational definition of MBTs for the purposes of our work: “structured musical activities designed to enhance well-being, reduce stress, and improve quality of life, without necessarily focusing on clinical diagnosis, assessment, or long-term psychotherapy”. While professional regulation and licensing remain important for clinical governance, our conceptualisation prioritises the active components of MBTs over jurisdiction-specific labels. Ultimately, understanding which components produce therapeutic effects is most important for advancing the evidence base. Our theoretical and conceptual justification for aggregating diverse music-based activities under the concept of MBTs lies in their shared complexity as interventions with potentially multiple “active ingredients” that promote health, well-being, and neurophysiological change.[23] MBTs are mid-range in their scope – broader than MT but narrower than MC, as MBTs do not include activities that are not therapeutic or do not comprise structured interventions. MT falls within the stated definition of MBTs, but not all aspects of MC are included under MBTs.

Evidence for Music-Based Therapies in MS

We have identified six previous systematic,[20, 24-28] scoping or comprehensive reviews on MBTs for MS, in addition to a broad non-systematic literature review on ‘cognitive re-education’ for QoL and psychological wellbeing in MS.[29] Table 1 summarises the scope, methodological approaches, interventions, outcomes, and included studies of these reviews. Due to clinical, methodological and statistical heterogeneity between studies, no reviews have been able to include a meta-analysis. An early comprehensive review[26] assessed MT in relation to a non-specified set of outcomes and found very limited evidence, with the only full-text peer-reviewed papers besides case reports or case series being two pilot studies.[30,31] Systematic and scoping reviews have typically been limited to specific outcome domains: i) gait and mobility[25,28] and ii) psychological outcomes.[24] Gardener et al[24] conducted a systematic review on MT for psychological outcomes in a broader range of conditions and included only one pilot RCT[32] on MS. Tan et al[28] conducted a recent scoping review of 15 studies on music based and rhythmic

auditory interventions for walking in MS. They focused exclusively on gait and mobility outcomes and reported high acceptability and consistent short-term improvements in walking speed and quality. Kong et al, 2023[25] similarly undertook a systematic review of 12 clinical trials of MT for gait disorders in MS, again concentrating on mobility outcomes and finding that music based rhythmic cueing improved gait performance. Together, these reviews support the role of music-based approaches for gait rehabilitation but do not explore wider motor, cognitive, or psychosocial domains. Lopes et al, 2021[20] provided the first MS specific systematic review of MBTs as a rehabilitative approach. Across 10 clinical trials, they grouped interventions into rhythmic auditory strategies, instrument playing, dance-based strategies and neurological MT. They found that MBTs were superior to conventional therapy or no intervention for several motor and non-motor outcomes, including gait, fatigue, balance, hand dexterity, emotional status and pain, although evidence for memory and mental fatigability was less clear. The search period ended in 2020 and interventions and outcome measures were highly heterogeneous, with several protocols primarily dance-based rather than primarily music focused.[20] Phillips et al, 2025[27] extended the scope of the literature by comparing active and receptive music interventions across 15 studies in people with MS, with outcomes including anxiety, depression, QoL, cognition, gait, balance, fatigue, and hand dexterity. Most interventions were receptive, often involving music listening during walking. While the review showed encouraging effects across motor and non-motor domains, its focus on engagement type and the limited number of studies mean it does not fully represent the breadth of MBTs for MS rehabilitation.[27]

Rationale

Existing systematic reviews are fragmented and limited in scope. Available reviews either focus narrowly on gait and walking-related outcomes,[25,28] only include a small number of heterogeneous studies,[20,27] or provide findings from multiple neurological conditions without MS-specific conclusions.[24] Reviews of related performing arts modalities, e.g. dance, highlight potential motor and psychological benefits for people with MS, yet these interventions do not isolate the therapeutic contribution of the musical component.[4] There is no recent systematic review of the potential benefit of MBTs across a broad range of rehabilitation outcomes relevant to people with MS. No existing review provides a comprehensive synthesis of the impact of MBTs on motor, cognitive, psychological or QoL domains, nor evaluates the diversity of interventions used.

Considering specifically reviews published in 2025, here is a clear statement of how they differ from our systematic review and the advance to knowledge our work offers:

- Gardener et al, 2025[24] - this systematic review has a specific focus on MT and psychological outcomes; our review considers MBTs and mobility, cognitive function and QoL outcomes, therefore offering broader intervention and outcome measure coverage.
- Phillips et al, 2025[27] – this systematic review considers ‘music interventions’, mode of engagement (active versus receptive interventions), and ‘balance’, ‘gait’, ‘anxiety’, ‘depression’, and ‘fatigue’.. Our review explicitly uses the MBTs framework to define eligibility, includes mobility and psychological outcomes, and includes comparison of MBTs and control arms.
- Tan et al, 2025[28] – this is a scoping review, while our review is a systematic review. Systematic reviews are considered a higher level of evidence compared to scoping reviews and offer a structured assessment of risk of bias.[33] This review considers ‘music interventions’ but does not use an explicitly defined framework such as MBTs, while our review explicitly uses the MBTs framework to define eligibility. This review

For peer review only

limits its outcomes to gait and mobility, while our review also considers cognitive function, psychological well-being, and QoL.

Therefore, our review provides a clear advance in knowledge in terms of coverage of interventions and outcomes. We provide the first systematic review to provide an assessment of the potential benefit of music in MS using the MBTs framework to ensure appropriately broad intervention coverage and assessment of mobility, cognitive function, psychological outcomes, and QoL outcomes.

Aims

The aim of our work was to provide a systematic review of the evidence for a benefit of MBTs on rehabilitation outcomes in adults with multiple sclerosis. In particular, the primary review question was:

- What is the impact of MBTs on rehabilitation outcomes in people with MS?

Secondary review questions were:

- What types of MBTs have been studied for their rehabilitation potential for people with MS?
- What specific rehabilitation domains (for example motor, cognitive, psychological, and QoL) show the greatest response to MBTs in people with MS?

METHODS

Design

A systematic review was conducted following the Preferred Reporting Items for Systematic Reviews and Meta-Analyses (PRISMA) 2020 guidelines.[34] Completed PRISMA and PRISMA for Abstracts checklists are provided (Supplemental files 1 and 2). Reporting of the narrative synthesis was compliant with the Synthesis without meta-analysis (SWiM) guidelines.[35] This systematic review was registered on the PROSPERO database (CRD420251083656). One protocol amendment was made – to use risk of bias tools from the same suite for all study designs.

Data sources

AMED (Ebsco), APA PsycINFO (Ovid), CINAHL (Ebsco), Cochrane-CENTRAL, EMBASE (Ovid) and MEDLINE (Ovid) were searched from inception until July 2025. Supplementary searches were conducted on Google Scholar and through forward and backward citation chasing on studies identified for full-text review up to November 2025. Searches were designed to retrieve articles on MS and MBTs (strategies for all databases are shown in online supplemental file 3) and were designed by NA and MSB and conducted by the lead author NA. MSB conducted supplementary searches and citation chasing.

Inclusion criteria

Screening was initially conducted based on the title and abstract. Potentially relevant articles were screened at the full-text stage to determine inclusion (online supplemental file 4) or exclusion (online supplemental file 5) in the systematic review. Screening was initially conducted by NA based on titles and abstracts, followed by full text screening of potentially eligible studies. A second reviewer (MSB) independently screened all records to verify consistency, and any disagreements were resolved through discussion.

For peer review only

Eligibility criteria are shown in Box 1. The outcomes of interest were rehabilitation-related outcomes: functional recovery, motor function, gait and balance, cognitive function, psychological well-being, and QoL. Studies with and without control arms were eligible. MBTs were defined using the operational definition proposed in the Introduction: "structured musical activities designed to enhance well-being, reduce stress, and improve quality of life, without necessarily focusing on clinical diagnosis, assessment, or long-term psychotherapy". As MT falls within this definition of MBTs, studies on MT were eligible for inclusion. There were no specific requirements for what control arms should involve. Therefore, control arms could include alternative populations, such as healthy controls or people with another medical condition rather than MS, in addition to alternative interventions. While no limits were set on the types of control arms that were eligible, based on previous systematic reviews on the performing arts in neurological settings[8-9] and knowledge of the field, it was anticipated that control arms may include exercise, physiotherapy, usual care (or no intervention), conventional cognitive rehabilitation, and other MBTs. Screening was conducted using Rayyan, which uses artificial intelligence, machine learning, and natural language processing to make systematic reviewing more efficient by learning from user decisions to predict article relevance and highlight key PICO terms. All screening was done manually by the two human reviewers.

Data extraction

Information extracted is shown in Box 2. All data extraction processes were conducted by NA, and MSB independently reviewed all records to verify consistency. Any disagreements were resolved through discussion. No automation tools were used. The appendix provides additional information on study characteristics (online supplemental file 6), interventions (online supplemental file 7), controls (online supplemental file 8) and narrative results (online supplemental file 9).

Risk of bias assessment

Risk of bias and methodological quality were assessed using the relevant Critical Appraisal Skills Programme (CASP) checklists for randomised controlled trials, cohort studies, case control studies and cross-sectional studies[36]. The descriptive, qualitative approach to CASP was used, whereby we tabulated and described the patterns of bias both within and between studies but did not use a numerical scoring system or assign overall 'high', 'moderate' and 'low' quality labels to studies. Avoidance of the use of numerical composite scores for risk of bias is in line with recommendations from the Agency for Healthcare Research and Quality (AHRQ)[37] in the USA. Quality appraisal was conducted independently by NA, with MSB reviewing all studies to ensure consistency. Disagreements were resolved through discussion. No automation tools were used. The appendix provides additional information on risk of bias checklists used (supplemental file 10).

Narrative synthesis

A thematic narrative synthesis was used to analyse all studies that met the inclusion criteria. Guidance recommends[38] that the decision whether to undertake a meta-analysis should be pre-specified based on clear a priori justification and conceptual understanding rather than decided post-hoc based on formal evaluation of identified studies. In all previous systematic reviews in the field, meta-analysis was not considered appropriate due to substantial methodological heterogeneity across eligible studies. These sources of heterogeneity included: i) study design (including RCTs and observational studies), ii) differences in type and delivery of MBTs (including active and receptive MBTs), iii) differences in disciplinary backgrounds of intervention leaders (including music therapists, other health professionals,

For peer review only - http://bmjopen.bmj.com/site/about/guidelines.xhtml

and professional performing artists), iv) differences in session length, frequency and duration, and v) differences in outcome measurement (both conceptual differences and differences in terms of what assessment tools and scales were used). In previous systematic reviews in the field, these factors were considered to preclude the forming of meaningful meta-analysis sets comprising sufficient studies assessing the same combination of intervention, control, and outcome. This precedent from previous systematic reviews was the key factor in our a priori decision that conducting meta-analysis was not feasible. Additionally, we conducted a qualitative post-hoc assessment of whether these issues were also present in the evidence base for our systematic review to confirm the appropriateness of our a priori decision. This showed that the same issues with conducting meta-analysis were present in the evidence base we identified as in the previous systematic reviews, supporting our decision to conduct exclusively narrative synthesis.

To conduct narrative synthesis, initially the narrative study results extracted during data extraction were reviewed to identify thematic commonalities and patterns in the data. This process identified four key outcome themes in the data: i) motor function, ii) cognitive function, iii) psychological outcomes and iv) QoL. The narrative synthesis was initially stratified by outcome using these four outcome domains. All studies that addressed a particular outcome domain were included in the synthesis for that outcome. Within each outcome domain, the synthesis was further stratified by the type of MBTs assessed. This could include rhythmic auditory stimulation, receptive music listening, active music making and structured MT interventions. However, the types of MBTs were not imposed on the synthesis a priori to allow these to emerge thematically from the data within each outcome domain. As a third level of stratification, within each type of MBTs for each outcome domain, evidence was stratified by interventional and observational study designs, also noting the evidence specifically available from RCTs. Our approach to narrative synthesis enabled comparison across rehabilitation domains and facilitated evaluation of how different MBTs contribute to rehabilitation outcomes in people with MS. For all outcomes, the primary standardised metric for narrative synthesis – based on the SWiM guidelines –[35] was direction of findings to provide an effective overall synthesis of the patterns of findings, while noting considerable differences in how outcomes were measured and analysed across studies. The potential of small-study bias was assessed using an effect direction plot. The potential of outcome reporting bias was assessed by comparing outcomes in published protocols or clinical trials registries with the outcomes in the journal publications. Heterogeneity could not be assessed quantitatively due to the differences in outcome assessment that precluded meta-analysis. However, differences in study designs, study characteristics, interventions and comparators were profiled in Supplemental files 6-8 and reflected upon at appropriate points in the results and discussion. The textual write-up of the narrative synthesis was supplemented by a detailed results table (Supplemental file 9). The table was ordered alphabetically, while the textual write-up was structured according to the stratification factors stated above (outcome, type of MBTs, and study design). The synthesis was initially conducted by one author (NA) and then reviewed, checked and revised by a second author (MSB).

Clinical significance

The approach to assessing clinical significance was pre-specified. Searches were conducted to identify minimally clinically significant differences (MCIDs)[39] for each of the key thematic areas in the narrative synthesis: i) motor function, ii) cognitive function, iii) psychological outcomes and iv) QoL. There was heterogeneity in the outcome measures used to assess these outcomes across studies. In assessing MCIDs,[40] it is considered best practice to evaluate only outcomes used in multiple studies (typically three or more studies) to ensure validity, stability, and consensus. Therefore, we conducted MCID assessment on outcomes

For peer review only

used on at least three studies included in our review. Furthermore, only outcomes that are on measurement scales amenable to MCID assessment and for which a published MCID can be identified could be included.[41] The distinction between within-group and between-group differences was considered in the reporting and in interpretation of MCID results. The MCIDs identified and applied are shown in Table 2.

Certainty assessment

We conducted certainty assessment using GRADE for each outcome domain supported by the narrative synthesis. As GRADE methods are currently being updated, we used the updated GRADE Book[42] for chapters that had been updated and the 2013 GRADE Handbook[43] for the remaining chapters, as recommended in the GRADE BOOK.[42] We refer to both books below as 'the GRADE guidance'. We describe the approach concisely below. In the GRADE guidance, evidence from RCTs initially start as high certainty evidence, while evidence from non-randomised and observational studies start as low certainty evidence. For RCTs, the risk of systematic or random errors may reduce confidence in the findings. Four GRADE domains assess the risk of systematic errors: 1) study limitations (risk of bias), 2) inconsistency, 3) indirectness, and 4) dissemination bias (publication bias). As noted in the limitations section of the discussion, it was not possible to formally assess publication bias in this systematic review. One GRADE domain evaluates the risk of random error: 1) imprecision. An RCT is downgraded one level from high certainty for every domain for which there are serious concerns, or by two levels if there are very serious concerns. Non-randomised and observational studies may be upgraded from low certainty evidence where the risk of confounding variables is relatively low (for example due to large effect sizes or dose-response gradients) or where the presence of a confounding variable strengthens the inference of effect. Detailed operationalisation criteria are found in the GRADE guidance. We followed the GRADE guidance that the starting point for an outcome domain shall be set as high if the body of evidence consists primarily of RCTs and low if it does not consist primarily of RCTs. The evidence is profiled in Table 3 along with explanations for the GRADE scoring decisions – the presentation of GRADE results is consistent with Barnish et al,[8] although we include this information in the main manuscript rather than the supplemental material for clarity. It was determined that the different forms of MBTs in the evidence base did not form sufficiently clear and distinct categories to conduct separate GRADE assessments by type of MBTs.

Patient and public involvement

Patient and public involvement could not be conducted for this systematic review assessing a broad range of MBTs due to a lack of funding. The corresponding author will respond to any reputable media enquiries.

RESULTS

Search results

Database searches returned a total of 5906 records (AMED 17, PsycINFO/APA PsycINFO 1155, EMBASE 4029 and MEDLINE 198, CINAHL 107, Cochrane central 400), plus 13 from supplementary searches. A total of 4918 records proceeded to title and abstract screening (including one duplicate). 47 unique records (including one duplicate) were assessed at full-text screening; 23 studies were included in the systematic review (Figure 1).

Study profile

Of the 23 included studies, there were 20 interventional studies (14 RCTs, two feasibility RCTs, two quasi-experimental controlled feasibility studies, one secondary analysis of an

For peer review only

RCT, and one matched controlled pilot study) and three observational studies (one observational cross-sectional comparative study, one case-control study, and one matched control study (details in Supplemental file 6). Six included studies (26%) described their approach as MT or the session leader as a music therapist (details in Supplemental file 7). Among the remaining 17 studies, six used gait training or motor imagery, five used rhythmic auditory stimulation, three used sung word list tasks, one used instrument playing, one used listening to music, and one used music to movement (M2M).[44] Three studies (13%) had no control arm. A further four studies (17%) had no control arm comprising people with MS – two of these had a 'healthy' control arm who received the MBTs intervention and two had a 'healthy' control arm who received no intervention. Sixteen studies (70%) had a control group with MS; in seven studies the control was no intervention, in three studies it was learning spoken word lists, in three studies it was a 'placebo' or sham intervention (playing a keyboard that was switched off, lying on top of a monochord that is not being played, or doing the same exercises without rhythmic auditory stimulation), in two studies it was conventional cognitive rehabilitation, and in one study it was receptive music activities – listening to music, discussing music, and learning about music and composers (details in Supplemental file 8). No studies compared MBTs to movement interventions (e.g., exercise or physiotherapy).

The included studies comprised 955 participants, of whom 880 had MS and 75 did not. Across all studies, 691 participants received MBT and 264 did not receive MBT and were therefore classified as control participants. Of the 75 participants without MS, 58 received MBT and 17 did not receive MBT. Therefore, the MBT and control totals include both participants with MS and participants without MS, depending on the study design. Sex was incompletely reported; where data were available, 666 participants (75.0%) were female and 222 (25.0%) were male. Reported mean ages across studies generally ranged from the mid-40s to early 50s, with overall participant ages spanning approximately 29 to 68 years. Disease duration was inconsistently reported, but where available typically ranged from approximately 7 to 18 years since diagnosis, with similar durations between intervention and control groups within individual studies. Baseline disability levels were most reported using the Expanded Disability Status Scale (EDSS). Across studies reporting EDSS, participants generally had mild to moderate disability, with median or mean scores most frequently ranging between 2.0 and 4.5. No studies directly compared different music-based intervention types with each other. MBTs assessed covered both active and passive forms of MT (instrumental and vocal) as well as other MBTs such as musically and rhythmically based cueing, and rhythmic auditory stimulation. No studies assessed choral singing or community singing groups. Further details, including on the types of MBTs assessed, are provided in Supplemental file 10.

Narrative synthesis

As there are 23 included studies, an overview of the evidence landscape is provided in Table 4. Several factors appeared to influence intervention effectiveness. Positive motor outcomes were most consistently reported in studies using rhythmic auditory cueing, RAS, and music-cued motor imagery. Benefits were generally observed in walking related outcomes; However, effects on fatigue, QoL, and psychological outcomes were more variable. In terms of contextual moderators, variations in intervention type (active vs. passive), cueing method, intervention duration, baseline disability level, and sample size may have contributed to differences in outcomes across studies.

Motor outcomes

For peer review only

Motor outcomes were assessed in 16 studies (70%) – profiled in Supplemental files 4-6. Fourteen studies were interventional – of which 9 were RCTs,[31,44,46,47,52,56,58,59,61] and 5 were other interventional designs (secondary analysis of RCT, quasi-experimental controlled feasibility study, and feasibility RCT)[49,51,54-56] – and two were observational studies.[48,53]

One study[31] (an RCT) looked at active music therapy and showed that numerically people with MS receiving active music therapy improved in terms of the strength of their respiratory muscles, while those in the passive music engagement group showed a numerical decline, although the between-group difference did not reach statistical significance. Active music making (keyboard playing) – not led by a music therapist – was associated with a statistically significant improvement (p=0.003, Cohen's d = 1.66, large effect) in hand function compared to playing with the keyboard switched off in one study[47] (an RCT). However, there was no between-group difference in hand dexterity or hand strength. Music to movement – which is a specific type[44] of active music intervention that is not led by a music therapist – showed a statistically significant difference compared to no active music intervention or yoga on two mobility outcomes (timed Up and Go, p=0.01, Cohen's d = 0.7, moderate effect; 6-minute walk test (p=0.05, Cohen's d = 0.6, moderate effect) in one study[44] (an RCT), although no statistically significant difference was found for 5 times sit-to-stand.

There were three RCTs[56,58,59] on motor imagery, all conducted by the same research group. All music-based groups showed statistically significant improvements in walking in all three studies, but there were no statistically significant differences between the different music-based groups. One study[59] showed synchronisation improved more with music and verbal motor imagery and music-cued motor imagery and worsened with just motor imagery. While one study[59] showed that physical fatigue only showed a statistically significant improvement after music and verbally cued motor imagery, another study[56] showed that this was found only for music-cued imagery. Studies using other interventional designs partly corroborated these findings – showing improved walking speed and distance after all music interventions[55,57] and reduced physical fatigue after music and verbally-cued motor imagery,[57] although one study only showed improvements in walking tests for those classed as 'responders', while response was associated with changes in brain activation patterns.[49] There were no observational studies for this intervention-outcome combination.

There were two RCTs[46,61] that assessed rhythmic auditory stimulation in relation to motor outcomes, both showing improvements in at some gait parameters. In one study, significant improvements were observed in double-support time, while additional parameters including velocity, cadence, stride length and step length consistently favoured the intervention group and were associated with moderate effect sizes despite not reaching statistical significance.[46] There was one study[54] using a different intervention design, which corroborated the findings from RCTs in terms of improvements in gait parameters versus no rhythmic stimulation. There was one observational study,[48] which showed an effect of frequency of rhythmic auditory stimulation on interlimb coordination (0.75Hz was worse than either 1.00Hz or 1.50Hz) with higher cognitive processing speed and attention scores were associated with better coordination.

Cognitive outcomes

Cognitive outcomes were assessed in 8 studies (35%) – profiled in Supplemental files 4-6. Seven studies were interventional – of which five were RCTs[32,52,59,62,63] and 2 were other interventional designs[30,45] – and one was an observational study.[53]

For peer review only

Specifically using neurologic music therapy methods, one study[32] (an RCT) showed statistically significant improvements in cognitive function ($p<0.001$), long-term retrieval ($p=0.007$) and delayed recall ($p=0.001$) in the music therapy group. One study[51] (using a different interventional design) on passive music listening (not led by a music therapist) showed statistically significant within-group effects in the music group on cognitive function ($p<0.001$) and long-term retrieval ($p<0.007$). There was no statistically significant difference versus conventional cognitive rehabilitation. It should be noted that conventional cognitive rehabilitation was only administered in the control arm for this study.

One study[53] (observational) assessed rhythmic auditory stimulation and found that people with MS perceived statistically significantly lower cognitive fatigue ($p<0.001$) while listening to music.

Psychological outcomes

Psychological outcomes were assessed in 7 studies (30%) – profiled in Supplemental files 4-6. Six studies were interventional – of which 4 were RCTs[32,50,59,60] and two were other interventional designs[45,51] – and one was an observational study.[30]

There was one RCT[60] that assessed active music therapy and showed that – from pre-test to post-test to follow-up, music therapy statistically significantly reduced depression and anxiety and improved self-esteem. One study[45] using a different interventional design did not find statistically significant differences versus control but did show medium effect sizes for improvements in self-esteem (Cohen's $d=0.54$), depression (Cohen's $d=0.64$), and anxiety (Cohen's $d= 0.63$). One observational study reported significant within-group improvements in depression ($p=0.036$), anxiety ($p=0.013$), and self-acceptance ($p=0.012$) following music therapy, however no significant differences were observed compared with the control group.[30] One study[50] (an RCT) assessed passive music therapy (listening to a monochord while lying on it) and showed statistically significant benefits for music therapy versus a silent condition lying on the monochord while it is not being played on psychosocial fatigue ($p=0.029$) and stronger immediate subjective effects ($p<0.001$), although the numerical improvement in anxiety outcomes did not reach statistical significance ($p=0.109$). One study[51] (using a different interventional design) showed statistically significant within-group improvements for passive music listening (not led by a music therapist) on motivation, emotional awareness and depression. There were no statistically significant differences versus conventional cognitive rehabilitation, which was only administered in the control arm.

Quality of life outcomes

QoL-related outcomes were assessed in 12 studies (52%) – profiled in Supplemental files 4-6. Ten studies were interventional – of which 6 were RCTs[32,44,50,56,58,59] and four were other interventional designs[45,51,55,57] – and two were observational.[30,53]

One RCT found that adding neurologic music therapy to conventional cognitive rehabilitation was associated with greater improvement in the mental health component of the MSQoL-54 ($p<0.001$) than conventional cognitive rehabilitation alone.[32] One interventional study[45] (using a different interventional design) and one observational study[30] of active music therapy, reported no significant improvement in QoL compared with a matched control group. Evidence for QoL and wellbeing outcomes was mixed. Studies assessing QoL directly reported variable findings, with significant improvements observed following passive music listening[50] and music-assisted gait training[51], whereas active music therapy studies found no significant improvements in overall QoL[30,45] Studies assessing related outcomes such as fatigue and pain interference also produced mixed findings, reporting a trend towards reduced fatigue but no significant effect on pain interference.[44]

For peer review only - http://bmjopen.bmj.com/site/about/guidelines.xhtml

Three RCTs assessed motor imagery[56,58,59] In two RCTs,[56,58] QoL showed a statistically significant improvement only after music and verbally cued motor imagery. A smaller feasibility study[57] using a different interventional design provided preliminary evidence to support a benefit on QoL.

Summary of synthesis and risk of bias

Overall, current evidence suggests that MBTs may provide benefits across motor, cognitive, psychological, and QoL domains in people with MS. However, heterogeneity in intervention type, outcome measures, control arms, and study design limits the strength of conclusions that can be drawn, and several outcomes were assessed in only a small number of studies. The risk of bias profile as well as the potential impact of risk of bias on the outcomes of the narrative synthesis are presented in online supplemental file 10. Potential outcome reporting bias was not evaluable in 13 studies (57%) as no protocol was available. In five studies (22%), all outcomes from the protocol were reported in the journal article and in one further study all primary outcomes were reported. In two studies (9%), primary outcomes from the protocol were not reported in the journal article, while in two further studies (9%), the primary outcomes from the protocol were reported in the journal article but were switched to secondary outcomes. Further details can be found in online supplemental file 11. The effect direction plot (supplemental file 12) show that all studies were classed as small or medium (as there were no studies with a sample size more than 300) – however there was no evidence that the results of studies differed materially dependent on sample size.

Clinical significance

Available evidence consistently supported a clinically significant benefit of MBTs on depressive symptoms as assessed by the Beck depression scale. However, less consistent evidence of a clinically significant benefit was found for motor function assessed by the timed 25-foot walk test and QoL assessed by the MSIS-29 physical subscale. Meanwhile, the only evaluable study for anxiety assessed by HADS-anxiety subscale did not find evidence of a clinically significant benefit. Further details can be found in supplemental file 13.

Certainty assessment

Certainty of evidence was assessed as Low for all outcome domains using the GRADE schemata, reflecting potential issues around heterogeneity and the presence of studies where multiple CASP domains were assessed at potential high risk of bias, although most included studies for each outcome domain were RCTs. All studies assessed within-group rather than between-group differences, although for some studies information was available for more than one group.

DISCUSSION

Summary

This systematic review synthesised evidence on MBTs for rehabilitation outcomes in people with MS, identifying 23 eligible studies published between 1999 and 2025. The value of a systematic review comes in the number of studies integrated in the same systematic review rather than how many studies had featured in any previous systematic review.[64] No previous systematic review had included more than 12[26-27] of the 23 studies (52%) that we identified. Indeed, we double the amount of evidence included in the most recent related review by Philips et al.[27] By doubling the amount of evidence on MBTs in MS that has been integrated together compared to any previous systematic review, we offer a clear advance in knowledge. Furthermore, the present review adds an assessment of clinical significance and certainty of findings. Across the 23 studies, outcomes could be mapped to four rehabilitation domains,

For peer review only

with motor outcomes most frequently assessed (16 studies), followed by QoL and related outcomes (12 studies), cognitive outcomes (8 studies), and psychological outcomes (7 studies). The most evidence among motor outcomes was found for gait and mobility, particularly where interventions used rhythmic auditory stimulation and music cued motor imagery, with improvements reported in walking speed, walking distance, cadence, stride length, and gait symmetry. Cognitive findings were mixed, with clearer benefits in music assisted learning paradigms (e.g. sung versus spoken word learning), while broader cognitive rehabilitation comparisons suggested similar rather than superior benefits relative to conventional rehabilitation. Psychological outcomes showed potential improvements in depression, anxiety, self-acceptance and self-esteem, but between group effects were not uniform. QoL and fatigue were frequently co-assessed, with several trials reporting improvements in fatigue and MS-specific QoL measures, though findings were heterogeneous. Importantly, this review incorporated studies that have been less emphasised in prior summaries and were necessary to include for a complete synthesis. Challenges were identified in terms of certainty of evidence as assessed by GRADE and reaching clinical significance thresholds across the sample for some outcomes. These may reflect the emerging nature of the evidence base for MBTs in MS, the arts in health more generally, and the methodological challenges associated therewith.

Interpretation of findings

This review extends and updates the MS rehabilitation literature on MBTs by both increasing the quantity of included evidence and the scope of interventions and outcomes compared with prior syntheses. A key interpretive issue is the distinction between active and receptive MBTs. The evidence base remains weighted toward receptive approaches, particularly music listening during walking or cueing based strategies. Phillips et al[27] concluded that most included interventions were receptive, with fewer active interventions, and highlighted gait parameters and fatigue as the most common outcomes. The findings in the present review corroborate that pattern using much larger evidence base but also suggest that the active versus receptive categorisation favoured by Phillips et al[27] may be too simplistic for MS rehabilitation. Phillips et al[27] searched from 2002 to June 2024 and included 15 studies,[27] three of which did not meet the inclusion criteria for our review as the interventions were not MBTs (see Table 1 for details). Furthermore, Phillips et al[27] (due to their 2002 search date limit) excluded key earlier work[19] as well as not identifying studies published since their June 2024 search date. The inclusion of Kuhlmann et al[50] in our review provides additional randomised evidence on receptive vibration-based therapy effects on fatigue and pain perception, while Helmlinger et al[49] contributes neuroimaging correlated outcomes that are not represented in reviews with earlier search cut-offs. While group singing-based interventions were shown to be common in Parkinson's disease (PD),[8] no evidence on these types of MBTs was identified in our review on MS. Across outcomes, evidence from our review suggests the greatest benefit for gait and mobility, with more heterogeneous findings for cognition and psychological outcomes. This likely reflects differences in intervention intent and dose, diversity in control arms, and inconsistent outcome measures. While there were multiple studies by the same authors,[49,55-59] they were separate studies, and except for one pair of studies that had a small overlap in sample (3%), it did not appear that there were overlapping participants (Supplemental file 11). However, comparability of findings across studies may be limited by differences in control arms across studies, meaning that MBTs are not always being compared to interventions of equivalent effectiveness. A further limitation of the evidence base was that the follow-up assessment typically took place shortly after the intervention, meaning we lack information about long-term benefit. This is a common limitation with clinical trials and could be addressed through observational registry studies.[65] As sample sizes were typically small, there is a possibility of findings being affected by small-sample bias. Furthermore, arts interventions are difficult to blind and therefore blinding is seldom conducted in research on the arts in health.[5,8,9] Therefore, the possibility of performance bias due to non-blinded

For peer review only

interventions should be considered. Overall, based on the available evidence, MBTs appeared promising across multiple rehabilitation domains in MS, but the evidence remains constrained by intervention heterogeneity, limited head-to-head comparisons of types of MBTs, and variable methodological reporting, which together limit the certainty of any single conclusion.

Strengths and limitations

This systematic review has several strengths. We used a comprehensive search strategy across multiple databases, followed PRISMA reporting standards,[34] and applied predefined eligibility criteria. The final search conducted in July 2025, within six months of manuscript submission (January 2026), which strengthens the currency of the evidence base.[66] The review adhered to PRISMA[34] reporting standards and applied predefined eligibility criteria, reducing the risk of selection bias. Data extraction was conducted using standardised forms, and risk of bias was assessed using design-appropriate CASP tools,[36] supporting consistency across heterogeneous study designs. The review also incorporated assessments of clinical significance and certainty of evidence, allowing interpretation beyond statistical significance alone.

However, limitations should be acknowledged. First, meta-analysis was not feasible because of substantial heterogeneity in study design, intervention type, comparator condition, intervention dose, and outcome measurement. As a result, the synthesis relied on narrative methods, which are less precise than quantitative pooling. Second, many included studies had small samples and were exploratory or pilot in nature, limiting the statistical power. Third, although many studies were randomised, blinding of participants and intervention providers was generally not possible because of the nature of MBTs, increasing the potential for performance bias. Also, Participant characteristic such as race and ethnicity were not consistently reported, limiting the assessment of generalizability. Finally, only studies published in English were eligible for inclusion – however no studies were excluded on grounds of language of publication, as no studies published in languages other than English that would otherwise be eligible for inclusion were identified. This may reflect limitations[67] in indexing of non-English language journals in major bibliographic databases. It is also noted[68] that subscription coverage of non-English language journals in university libraries in English-speaking countries is limited, which would limit access to full texts of non-English language articles if they had been eligible for inclusion. It was not possible to assess publication bias as the data for most outcomes were not amenable to such aggregation – for many of the same reasons as meta-analysis could not be conducted, including differences in sampling frames and differences in how outcomes were assessed. The groupings in the narrative synthesis were broad and may not reflect granular differences in outcome and intervention types – this was necessary because of the relatively low (n=23) number of total studies.

Implications for research and practice

Future research should prioritise addressing the methodological limitations identified in this review. Larger, adequately powered randomised controlled trials are needed, with clearer reporting[67] of allocation concealment, participant flow, and effect size precision. Greater standardisation of outcome measures, particularly for gait, fatigue, cognition, and QoL, would enhance comparability and enable future quantitative synthesis. Reporting of clinically meaningful change, alongside statistical significance, should be prioritised to support translation into practice. There is a clear need for studies that directly compare different MBTs, including active versus receptive therapies. The relative lack of research on singing-based interventions in MS represents an evidence gap, which contrasts with evidence in Parkinson's disease, where singing is a frequently studied therapeutic modality.[8] Future studies should also expand beyond gait-focused outcomes to include communication, social participation, and patient-prioritised outcomes. From a clinical perspective, the current evidence base

For peer review only

supports cautious integration of MBTs, particularly rhythmic cueing and music-cued motor imagery, within MS rehabilitation programmes. These interventions appear feasible in both facility and home-based settings and may complement conventional physiotherapy and cognitive rehabilitation. However, given heterogeneity and residual risk of bias, MBTs should currently be considered an adjunct rather than a replacement for established rehabilitation approaches.

Conclusion

This systematic review synthesised evidence from 23 studies examining the effects of MBTs on motor, cognitive, psychological, and QoL outcomes in people with multiple sclerosis. The narrative synthesis suggests that MBTs, particularly rhythmic auditory stimulation and music-cued motor imagery, may provide benefits for gait and mobility, with more variable evidence for cognitive, psychological, and QoL outcomes. Evidence for cognitive benefit was found most in music-assisted learning paradigms, while psychological and fatigue-related outcomes showed mixed but promising signals.

This review extends prior rehabilitation-focused syntheses by including a broader range of intervention types, outcome domains, and recent studies, while maintaining methodological consistency through exclusive use of CASP risk of bias tools. Nonetheless, heterogeneity in interventions, outcomes, and study design limits the strength of conclusions. Overall, MBTs appear to be a promising adjunct in MS rehabilitation, but higher-quality, standardised research is required to clarify optimal intervention models and establish clinical effectiveness.

DECLARATIONS

Contributors. MSB provided some initial suggestions of topic areas in which he would be willing to supervise a student project on the Royal Society for Medicine systematic review programme for medical students. NA suggested a specific topic for this review which, through discussion, NA and MSB refined to form the protocol for this systematic review. NA designed the database searches, refined them through discussion with MSB, and ran these searches. MSB designed and conducted the supplementary searches and citation chasing. Screening, data extraction and risk of bias assessment were conducted by NA and MSB. NA and MSB conducted the narrative synthesis and interpreted the findings. MSB conducted the GRADE analysis. NA wrote the first draft of the manuscript, which was initially revised by MSB prior to circulation to all authors. MSB wrote the supplemental materials. DW and JK provided valuable contributions to interpreting the findings and revised the manuscript for substantive intellectual content. All authors read the final version of the manuscript prior to submission and take responsibility for their own work as appropriate. NA is a medical student and her work on this project was supervised by MSB (Senior Research Fellow). JK is a postdoctoral fellow and her work on this project was supervised by DW (Assistant Professor and centre Co-Director). NA was the project manager and MSB was the project director. MSB is the guarantor and takes overall responsibility for the work.

Acknowledgements. This work was done under the auspices of the Royal Society of Medicine systematic review programme for medical students. NA was a co-ordinator of the programme as well as a participant. MSB supervised NA on this programme. The programme did not provide any specific funding for this work. DW is Assistant Professor in Physical Medicine and Rehabilitation and Co-Director (Arts & Medicine), Center for History, Humanities, Arts, Social Sciences & Ethics in Medicine, University of Michigan, Ann Arbor, USA. MSB previously received expenses but not payment for arts promotion activities, however these ceased in 2010 and consequently no longer represent a competing interest.

Funding. The authors have not declared a specific grant for this research from any funding agency in the public, commercial or not-for-profit sectors.

For peer review only

Competing interests. The authors declare no competing interests.

Patient and public involvement. Patients and/or the public were not involved in the design, or conduct, or reporting, or dissemination plans of this research.

Patient consent for publication. Not applicable.

Ethics approval. No ethical approval was required as this is a systematic review. The University contact for research integrity is researchintegrity@exeter.ac.uk.

Conference presentation. This work has not been published or submitted to any other journal. A conference abstract has been accepted an oral presentation at the 2nd International Meeting of Arts Prescribing in HealthCare, Olympus Riviera, Greece, April 2026.

Data availability statement. All data relevant to the study are included in the article or uploaded as supplementary information. The presented work is a systematic review. All relevant information is provided in the manuscript and appendices. This includes the data extraction form completed with the data from all included studies. No meta-analyses were conducted, so there is no analytical code.

REFERENCES

1. Portaccio E, Magyari M, Havrdova EK, et al. Multiple sclerosis: emerging epidemiological trends and redefining the clinical course. *Lancet Reg Health Eur;* 2024;44: 100977.
2. GBD 2016 Multiple Sclerosis Collaborators. Global, regional, and national burden of multiple sclerosis 1990–2016: a systematic analysis for the Global Burden of Disease Study 2016. *Lancet Neurol* 2019;18(3):269-85.
3. Jakimovski D, Bittner S, Zivadinov R, et al. Multiple sclerosis. *Lancet* 2023;403 (10422): 183-202.
4. Davis E, Webster A, Whiteside B, et al. Dance for multiple sclerosis: a systematic review. *Int J MS Care* 2023; 25(4): 176-85.
5. Barnish MS, Nelson-Horne B. Group-based active artistic interventions for adults with primary anxiety and depression: a systematic review. *BMJ Open* 2023;13(6):e069310.
6. Pérez-Eizaguirre M, Vergara-Moragues E. Music therapy interventions in palliative care: a systematic review. *J Palliat Care* 2021;36(3):194–205.
7. Liu Q, Li W, Yin Y, et al. The effect of music therapy on language recovery in patients with aphasia after stroke: a systematic review and meta-analysis. *Neurol Sci* 2022;42(3):863–72.
8. Barnish MS, Reynolds SE, Nelson-Horne RV. Active group-based performing arts interventions in Parkinson's disease: an updated systematic review and meta-analysis. *BMJ Open* 2025;15(4):e089920.
9. Barnish MS, Barran SM. A systematic review of active group-based dance, singing, music therapy and theatrical interventions for quality of life, functional communication, speech, motor function and cognitive status in Parkinson's disease. *BMC Neurol* 2020;20(1):371.
10. Barnish J, Atkinson RAA, Barran SM, et al. Potential benefit of singing for people with Parkinson's disease: a systematic review. *J Parkinsons Dis* 2016;6(3):473–84.
11. American Music Therapy Association. What is music therapy? Available at:// https://www.musictherapy.org/about/musictherapy/ [Accessed 8 May 2026].
12. British Association for Music Therapy. What is a music therapist? Available at :// https://www.bamt.org/music-therapy/what-is-a-music-therapist [Accessed 8 May 2026].
13. Canadian Association of Music Therapists. About music therapy. Available at:// https://www.musictherapy.ca/about-camt-music-therapy/about-music-therapy/ [Accessed 8 May 2026].
14. Mainka S, Weymann E. Music therapy newly defined. Approaches 2025; 17(4): 812-24.

15. Skanland MS. Music therapy and social recovery in flexible assertive community treatment. Nord J Music Ther 2023; 32(4): 290-306.
16. Nebelung I, Kruger V. Norway: country report on professional recognition of music therapy. Approaches 2015; 7(1): 171-2.
17. Xiao H, Chen C. The study of traditional Chinese medicine five-element music therapy for depression: a bibliometric analysis. Digit Chin Med 2025; 8(4): 558-70.
18. Spiro N, Sanfilippo KRM (Eds.). Collaborative insights: interdisciplinary perspectives on musical care throughout the life course. Oxford: Oxford University Press; 2022.
19. Lin TH, Liao YC, Tam KW, et al. Effects of music therapy on cognition, quality of life, and neuropsychiatric symptoms of patients with dementia: A systematic review and meta-analysis of randomized controlled trials. *Psychiatry Res* 2023; 329: 115498.
20. Lopes J, Keppers II. Music-based therapy in rehabilitation of people with multiple sclerosis: a systematic review of clinical trials. *Arq Neuropsiquiatr* 2021;79(6):527–35.
21. Stegemann T, Geretsegger M, Phan QC, et al. Music therapy and other music-based interventions in pediatric health care: an overview. *Medicines (Basel)* 2019; 6(1): 25.
22. Raglio A. A novel music-based therapeutic approach: the Therapeutic Music Listening. Front Hum Neurosci 2023; 17: 1204593.
23. Wolff L, Quan Y, Perry G, et al. Music engagement as a source of cognitive reserve. Am J Alzheimers Dis Other Demen 2023; 38: 15333175231214833.
24. Gardener SH, Mukaetova-Ladinska EB, Perera NA. The effect of music therapy on psychological outcomes for neurological conditions: a systematic review. *Medicina* 2025;61(9):1611.
25. Kong L, Zhang X, Meng L, et al. Effects of music therapy intervention on gait disorders in persons with multiple sclerosis: a systematic review of clinical trials. *Mult Scler Relat Disord* 2023;73:104629.
26. Ostermann T, Schmid W. Music therapy in the treatment of multiple sclerosis: a comprehensive literature review. *Expert Rev Neurother* 2006;6(4):469–77.
27. Phillips CS, Kim J, Ganesh A, et al. Impact of active versus receptive music interventions on psychosocial and neurological outcomes in people with multiple sclerosis: a systematic review. *J Integr Complement Med* 2025;31(10):889–903.
28. Tan Y, Chen X, Wang L, et al. Music interventions for gait and mobility in multiple sclerosis: a scoping review. *Mult Scler Relat Disord* 2025;101: 106563.
29. Manocchio L, Moriano C, D'Amato A, et al. Beyond cognition: cognitive re-education and psychological wellbeing in people with multiple sclerosis: a narrative review. *NeuroSci* 2025; 6(3): 45.
30. Schmid W, Aldridge D. Active music therapy in the treatment of multiple sclerosis patients: a matched control study. *J Music Ther* 2004;41(3):225–40.
31. Wiens ME, Reimer MA, Guyn HL. Music therapy as a treatment method for improving respiratory muscle strength in patients with advanced multiple sclerosis: a pilot study. *Rehabil Nurs* 1999;24(2):74–80.
32. Impellizzeri F, Leonardi S, Latella D, et al. An integrative cognitive rehabilitation using neurologic music therapy in multiple sclerosis: a pilot study. *Medicine (Baltimore)* 2020;99(4):e18866.
33. Munn Z, Peters MDJ, Stern C, et al. Systematic review or scoping review? Guidance for authors when choosing between a systematic or scoping review approach. *BMC Med Res Methodol* 2018; 18(1): 143.
34. Page MJ, McKenzie JE, Bossuyt PM, et al. The PRISMA 2020 statement: an updated guideline for reporting systematic reviews. *BMJ* 2021;372:n71.
35. Campbell M, McKenzie JE, Sowden A, et al. Synthesis without meta-analysis (SWiM) in systematic reviews: reporting guideline. BMJ 2020; 368: l6890.
36. Critical Appraisal Skills Programme. CASP checklists [online]. https://casp-uk.net/casp-tools-checklists/ (accessed 12 December 2025).
37. Viswanathan M, Ansari NT, Berkman ND, et al. Assessing the risk of bias of individual studies in systematic reviews of health care interventions. In: Agency for Healthcare Research and Quality (US). Methods Guide for Effectiveness and Comparative

For peer review only

Effectiveness Reviews. Rockville (MD): Agency for Healthcare Research and Quality; 2008.
38. Johnson BT, Hennessy EA. Systematic reviews and meta-analyses in the health sciences: Best practice methods for research syntheses. *Soc Sci Med* 2019; 233: 237-51.
39. Jaeschke R, Singer J, Guyatt GH. Measurement of health status. Ascertaining the minimal clinically important difference. *Control Clin Trials* 1989;10(4):407-415.
40. Copay AG, Subach BR, Glassman SD, et al. Understanding the minimum clinically important difference (MCID) of patient-reported outcome measures. Spine J 2007; 7(3): 333-43.
41. Engel L, Beaton D E, Touma Z. Minimal clinically important difference: a review of outcome measure score interpretation. *Rheum Dis Clin* 2018*:* 44(2), 177-88.
42. The GRADE Working Group. The GRADE Book. Available at:// https://book.gradepro.org/about [Accessed 10 May 2026].
43. Schunemann H, Brozek J, Guyatt G, et al (Editors). GRADE handbook for grading quality of evidence and strength of recommendations. The GRADE Working Group; 2013
44. Young HJ, Mehta TS, Herman C, et al. The effects of M2M and adapted yoga on physical and psychosocial outcomes in people with multiple sclerosis. *Arch Phys Med Rehabil* 2019;100(3):391–400.
45. Aldridge D, Schmid W, Kaeder M, et al. Functionality or aesthetics? A pilot study of music therapy in the treatment of multiple sclerosis patients. *Complement Ther Med* 2005;13(1):25–33.
46. Conklyn D, Stough D, Novak E, et al. A home-based walking program using rhythmic auditory stimulation improves gait performance in patients with multiple sclerosis: a pilot study. *Neurorehabil Neural Repair* 2010;24(9):835–42.
47. Gatti R, Tettamanti A, Lambiase S, et al. Improving hand functional use in subjects with multiple sclerosis using a musical keyboard: a randomized controlled trial. *Physiother Res Int* 2015;20(2):100–7.
48. Goetschalckx M, Van Geel F, Meesen R, et al. Rhythmic interlimb coordination of the lower limbs in multiple sclerosis during auditory pacing to three different frequencies. *Gait Posture* 2021;86:334–40.
49. Helmlinger B, Seebacher B, Ropele S, et al. Effects of rhythmic-cued gait training on gait-like task related brain activation in people with multiple sclerosis. *J Neurol Sci* 2025;471:123426.
50. Kuhlmann J, Ebner K, Zimmer A, et al. Music therapy with a monochord in multiple sclerosis (“MUTIMS”): a randomized, controlled, rater-blinded trial. *Mult Scler J Exp Transl Clin* 2025;11(3):20552173251352712.
51. Maggio MG, Tripoli D, Porcari B, et al. How may patients with MS benefit from using music assisted therapy? A case-control feasibility study investigating motor outcomes and beyond. *Mult Scler Relat Disord* 2021;48:102713.
52. Moore KS, Peterson DA, O’Shea G, et al. The effectiveness of music as a mnemonic device on recognition memory for people with multiple sclerosis. *J Music Ther* 2008;45(3):307–29.
53. Moumdjian L, Moens B, Maes PJ, et al. Continuous 12 min walking to music, metronomes and in silence: auditory-motor coupling and its effects on perceived fatigue, motivation and gait in persons with multiple sclerosis. *Mult Scler Relat Disord* 2019;35:92–9.
54. Naderi S, Sadeghi H, Amirseyfaddini M. The effects of rhythmic auditory stimulation with different tempos on spatio-temporal parameters and gait symmetry in patients with multiple sclerosis: a quasi-experimental study. *J Res Rehabil Sci* 2022;18(1): 92-9.
55. Seebacher B, Kuisma R, Glynn A, et al. Rhythmic cued motor imagery and walking in people with multiple sclerosis: a randomized controlled feasibility study. *Pilot Feasibility Stud* 2015;1:25.

For peer review only

56. Seebacher B, Kuisma R, Glynn A, et al. The effect of rhythmic-cued motor imagery on walking, fatigue and quality of life in people with multiple sclerosis: a randomized controlled trial. *Mult Scler J* 2017;23(2):286–96.
57. Seebacher B, Kuisma R, Glynn A, et al. Exploring cued and non-cued motor imagery interventions in people with multiple sclerosis: a randomized feasibility trial and reliability study. *Arch Physiother* 2018;8:6.
58. Seebacher B, Kuisma R, Glynn A, et al. Effects and mechanisms of differently cued and non-cued motor imagery in people with multiple sclerosis: a randomised controlled trial. Mult Scler J 2019; 25(12): 1593-1604.
59. Seebacher B, Helmlinger B, Pinter D, et al. Actual and imagined music-cued gait training in people with multiple sclerosis: a double-blind randomized parallel multicenter trial. *Neurorehabil Neural Repair* 2024;38(8):555–69.
60. Seighalani MZ, Ghahari S, Zarbakhsh M. The effectiveness of music therapy on depression, anxiety and self-esteem of patients with multiple sclerosis. *Adv Environ Biol* 2014;8(9):253–60.
61. Shahraki M, Sohrabi M, Taheri Torbati HR, et al. Effect of rhythmic auditory stimulation on gait kinematic parameters of patients with multiple sclerosis. *J Med Life* 2017;10(1):33–7.
62. Thaut MH, Peterson DA, Sena KM, et al. Musical structure facilitates verbal learning in multiple sclerosis. *Music Percept* 2008;25(4):325–30.
63. Thaut MH, Peterson DA, McIntosh GC, et al. Music mnemonics aid verbal memory and induce learning-related brain plasticity in multiple sclerosis. *Front Hum Neurosci* 2014;8:395.
64. Ioannidis JP The mass production of redundant, misleading, and conflicted systematic reviews and meta-analyses. *Millbank Q* 2016; 94(3): 485-514.
65. Barnish MS, Turner S. The value of pragmatic and observational studies in health care and public health. *Pragmat Obs Res*.2017;8:49-55.
66. Higgins JPT, Thomas J, Chandler J, et al (Eds). *Cochrane Handbook for Systematic Reviews of Interventions* version 6.5 (updated August 2024). Available at:// www.cochrane.org/handbook [Last accessed 6 January 2026].
67. Aali G, Shokraneh, F. No limitations to language, date, publication type, and publication status in search step of systematic reviews. *J Clin Epidemiol 2021; 133: 165-7.*
68. Absher LU, Desilets MR. English as the scholarly language: diversity, equity, and inclusion implications for academic reference and instruction librarians. *Libr Q* 2024; 94(3): 296-315.
69. Robb SL, Story KM, Harman E, et al. Reporting Guidelines for Music-based Interventions checklist: explanation and elaboration guide. Front Psychol 2025;16:1552659.
70. Ng A, Bunyan S, Suh J, et al. Ballroom dance for persons with multiple sclerosis: a pilot feasibility study. *Disabil Rehabil* 2020;42(8):1115–21.
71. Van Geel F, Van Asch P, Veldkamp R, et al. Effects of a 10-week multimodal dance and art intervention program leading to a public performance in persons with multiple sclerosis: a controlled pilot trial. *Mult Scler Relat Disord* 2020;44:102256.
72. Basturk S, Ekici G, Kirteke F, et al. Therapeutic effects of line dancing in people with multiple sclerosis: an evaluator-blinded randomized controlled study. *Arts Health* 2024; doi: 10.1080/17533015.2024.2325425 [Online ahead of print].
73. Baram Y, Miller A. Auditory feedback control for improvement of gait in patients with multiple sclerosis. *J Neurol Sci* 2007;254 (1-2):90–4.
74. Moumdjian L, Maes PJ, Dalla Bella S, et al. Detrended fluctuation analysis of gait dynamics when entraining to music and metronomes at different tempi in persons with multiple sclerosis. *Sci Rep* 2020;10(1):12934.
75. Moumdjian L, Moens B, Maes PJ, et al. Walking to music and metronome at various tempi in persons with multiple sclerosis: a basis for rehabilitation. *Neurorehabil Neural Repair* 2019;33(6):464–75.

For peer review only

76. Coleman CI, Sobieraj DM, Marinucci LN. Minimally important clinical difference of the Timed 25-Foot Walk Test: results from a randomized controlled trial in patients with multiple sclerosis. *Curr Med Res Opin*. 2012;28(1):49-56.
77. Button KS, Kounali D, Thomas L, et al. Minimal clinically important difference on the Beck Depression Inventory--II according to the patient's perspective. *Psychol Med*. 2015;45(15):3269-79.
78. Lemay KR, Tulloch HE, Pipe AL, et al. Establishing the minimal clinically important difference for the Hospital Anxiety and Depression Scale in patients with cardiovascular disease. *J Cardiopulm Rehabil Prev* 2019; 39(6): E6-11.
79. Puhan MA, Frey M, Buchi S, et al. The minimal important difference of the hospital anxiety and depression scale in patients with chronic obstructive pulmonary disease. *Health Qual Life Outcomes* 2008: 2(6):46.
80. Canadian Agency for Drugs and Technologies in Health. *Clinical review report: Daclizumab (Zinbryta*). Ottawa (ON): CADTH; 2017.
81. Costelloe L, O'Rourke K, Kearney H, et al. The patient knows best: significant change in the physical component of the Multiple Sclerosis Impact Scale (MSIS-29 physical). *J Neurol Neurosurg Psychiatry* 2007;78(8):841-
82. Boon MH, Thomson H. The effect direction plot revisited: Application of the 2019 Cochrane Handbook guidance on alternative synthesis methods. *Res Synth Methods* 2020; 12(1): 29-33.

For peer review only

TABLES AND FIGURES

Box 1. Inclusion criteria for the systematic review

Eligible studies assessed:

- Population: People with a diagnosis of MS
- Intervention: Studies involving MBTs or music interventions delivered for the purpose of rehabilitation in people with multiple sclerosis. Interventions could include:
  - Active music-making (e.g., instrument playing)
  - Passive listening to music (pre-recorded or live)
  - Rhythmic auditory stimulation (RAS)
  - Structured MT delivered by a trained therapist
  - Group or individual sessions of MBTs
- Control arms: Studies with and without control arms were eligible. There were no specific requirements for what control arms should involve.
- Outcomes: Studies that report rehabilitation-related outcomes: functional recovery, motor function, gait and balance, cognitive function, psychological well-being, QoL.
- Other: Studies available in full-text and published in the English language involving human participants aged 18 and over with a confirmed diagnosis of multiple sclerosis. Studies conducted in any healthcare, community, or rehabilitation setting, provided they meet the control arm and outcome criteria outlined above.

Given the emergent evidence base, we included studies that provided data that were informative for the listed outcome domains but did not cover the full breadth of the domain. For example, the MSFC scale covers cognition but is not exclusively on cognition but was considered relevant. Furthermore, studies addressing pain, general fatigue, and quality of life subscales rather than a formal quality of life scale that covers the full breadth of quality of life were considered relevant.

Box 2. Data Extracted

The following information was extracted for each included study:

- Bibliographic details (authors, year and citation).
- Country of study.
- Study design.
- Participants (sample size, sex profile, mean age or age range, MS type, disease duration, EDSS score, use of walking aids, left/right-handed – as this variable is often considered in neuropsychological studies).
- Inclusion criteria.
- Outcomes.
- Content of intervention.
- Professional background of intervention leader – as work in PD[8] has shown large differences in leader background and expertise within and between studies.
- Location of intervention (eg, health centre or home).
- Frequency and duration of intervention.
- Content of control arm.
- Professional background of leader of intervention delivered to the control arm.
- Location of control arm (eg, health centre or home).
- Frequency and duration of control arm.
- Study results for narrative synthesis for all eligible reported outcomes.

For peer review only - http://bmjopen.bmj.com/site/about/guidelines.xhtml

Table 1. Previous systematic, scoping and comprehensive reviews

Authors	Method	Search date	PROSPERO	Interventions	Outcomes	Included studies
Gardener et al, 2025[24]	Systematic review (without meta-analysis)	January 2025 (limited to literature since 2015)	Not registered, no pre-published protocol	MT	Psychological outcomes	Wider range of neurological conditions including MS. For MS:<br>1 pilot RCT (Impellizeri et al, 2020).[32]
Kong et al, 2023[25]	Systematic review (without meta-analysis)	October 2022	CRD 42022365668	MT	Gait and mobility outcomes	2 case series. 7 RCTs (Conklyn et al, 2010;[46] Seebacher et al, 2015;[55] Seebacher et al, 2017;[56] Seebacher et al, 2018;[57] Seebacher et al, 2019;[58] Shahraki et al, 2017;[61] Young et al, 2019).[44]<br>3 non-RCTs (Maggio et al, 2021;[51] Ng et al, 2020;[70] Van Geel et al, 2020).[71]
Lopes et al, 2021[20]	Systematic review (without meta-analysis)	July 2020	CRD 42020143080	MBTs	Balance, cognition, dexterity, fatigue, gait, pain, QoL	10 clinical trials (Aldridge et al, 2005;[45] Conklyn et al, 2010;[46] Gatti et al, 2015;[47] Van Geel et al, 2020;[71] Impellizeri et al, 2020;[32] Moore et al, 2008;[52] Seebacher et al, 2019;[58] Seebacher et al, 2017;[56] Thaut et al, 2014;[63]* Young et al, 2019).[44]
Ostermann et al, 2006[26]	Comprehensive review	Not stated	Not registered (review pre-dates PROSPERO)	MT	Not specified	8 conference abstracts, chapters or non-peer-reviewed sources.<br>2 case reports or case series.<br>2 pilot studies (Schmid et al 2004;[30] Wiens et al, 1999).[19]

For peer review only

Phillips et al, 2025[27]	Systematic review (without meta-analysis)	June 2024	CRD 42022338291	Music interventions (active or receptive)	Anxiety, balance, cognition, depression, dexterity, fatigue, gait, QoL	10 RCTs (Basturk et al, 2024;[72] Conklyn et al, 2010;[46] Gatti et al, 2015;[47] Impellizzeri et al, 2020;[32] Moore et al, 2008;[52] Seebacher et al, 2017;[56] Seebacher et al, 2019;[58] Thaut et al, 2008;[62] Thaut et al, 2014;[63] Young et al, 2019).[44] 5 quasi-experimental studies (Maggio et al, 2021;[51] Moumdjian et al, 2019;[53] Schmid et al, 2004;[30] Van Geel et al, 2020).[71]
Tan et al, 2025[28]	Scoping review	April 2025	Not registered (but protocol uploaded to Open Science Framework)	Music interventions	Gait and mobility	8 RCTs (Baram & Miller, 2007;[73] Conklyn et al, 2010;[46] Helmlinger et al, 2025;[49] Seebacher et al, 2024;[59] Seebacher et al, 2019;[58] Seebacher et al, 2018;[57] Seebacher et al, 2017;[56] Seebacher et al, 2015;[54] Shahraki et al, 2017).[41]** 3 non-randomised experimental studies (Maggio et al, 2021;[51] Moumdjian et al, 2020;[74] Naderi et al, 2022).[54] One cross-sectional study (Goetschalckx et al, 2021).48 Two case-control studies (Moumdjian et al, 2019a;[53],Moumdjian et al, 2019b).[75] Two case studies.

* The citation for Thaut et al (2014)[63] was incorrect in the bibliography of the cited review but appears to refer to this study. ** The review states 8 RCTs but provides 9 citations – it would appear that 2 papers are being referred to as parts of the same study, but this is not made clear. All included studies from these previous reviews were included in our review with the following exceptions: i) case studies, case reports, case series, conference abstracts, chapters and non-peer-reviewed sources were not eligible publication types for our review; ii) Basturk et al, 2024;[72] Ng et al (2020),[70] and Van Geel et al (2020)[71] were both dance intervention studies which were not eligible for our review because the intervention was not primarily music-based; Baram and Miller (2007)[73] is about auditory feedback control but makes no mention of music and is therefore not eligible for our review; Moumdjian et al. (2019b,[74] 2020)[75] were excluded because they examined movement synchronisation to music or metronome without assessing rehabilitation or clinical outcomes in people with MS. We have checked (May 2026) and the Basturk et al[72] 'online ahead of print' paper from 2024 has not yet been published in its final version.

Table 2. Minimally clinically important differences identified

Outcome domain	Measure	Number of studies	MCID
Motor	Timed 25-foot walk test	6	MCID of a 17.2% improvement in test performance has been proposed based on trial data,[76] which accords well to previous clinical proposals of a 20% improvement
Cognitive	N/A	N/A	No pivotal measures used in at least three studies identified
Psychological	Beck depression	3	No MS-specific MCID identified, however noting clinical significance depends on baseline depression severity, a population-agnostic MCID of a 17.5% reduction from baseline has been proposed[77]

For peer review only

Psychological	HADS anxiety	5	No MS-specific MCID identified, however an MCID of 1.70 points[78] has been found for HADS told in cardiovascular disease and 1.60 points[79] in chronic obstructive pulmonary disease. Halving for the anxiety subscale alone, gives an MCID of 0.80-0.85
QoL	MS Impact Scale-29 (MSIS-29)	3	No overall MCID identified,[80] although an MCID of 8 points has been proposed for the physical subscale.[81]

Table 3. GRADE scoring and explanation

Outcome domain	GRADE	Explanation
Motor	Low	16 studies, of which 10 RCTs (including feasibility RCTs but excluding secondary analysis of RCT data). Starting GRADE High as most of the evidence is randomised (as per GRADE guidance). Downgrade to Moderate because of heterogeneity. Downgrade to Low because the set of studies included evidence considered to be low quality.
Cognitive	Low	8 studies, of which 5 RCTs. Starting GRADE High as most of the evidence is randomised (as per GRADE guidance). Downgrade to Moderate because of heterogeneity. Downgrade to Low because the set of studies included evidence considered to be low quality.
Psychological	Low	7 studies, of which 4 RCTs. Starting GRADE High as most of the evidence is randomised (as per GRADE guidance). Downgrade to Moderate because of heterogeneity. Downgrade to Low because the set of studies included evidence considered to be low quality.
QoL	Low	12 studies, of which 8 RCTs. Starting GRADE High as most of the evidence is randomised (as per GRADE guidance). Downgrade to Moderate because of heterogeneity. Downgrade to Low because the set of studies included evidence considered to be low quality.

Table 4. Distribution of included studies according to rehabilitation outcome domains

First Author, Year	Motor outcomes	Cognitive outcomes	Psychological outcomes	QoL outcomes
Aldridge et al, 2005[45]	–	Global cognitive function	Depression, anxiety, self-acceptance	QoL, functional disability, fatigue
Conklyn et al, 2010[46]	Gait and mobility; muscle strength; spasticity and movement related disability	–	–	Pain; functional disability
Gatti et al, 2015[47]	Upper limb function	–	–	–
Goetschalckx et al, 2021[48]	Balance and stability; coordination and synchronisation	–	–	–
Helminger et al, 2025[49]	Gait and mobility	–	–	–

For peer review only

Impellizzeri et al, 2020[32]	–	Global cognitive function	Mood and emotional processing	MS specific QoL
Kuhlmann et al, 2025[50]	–	–	Anxiety; depression; body perception	Fatigue; pain
Maggio et al, 2021[51]	Gait and mobility; balance and stability	–	Depression; emotional components	MS specific QoL
Moore et al, 2008[52]	–	Learning and memory; executive function and attention	–	–
Moumdjian et al, 2019[53]	Gait and mobility; coordination and synchronisation	Cognitive Fatigue	–	Fatigue
Naderi et al, 2022[54]	Gait and mobility	–	–	–
Schmid et al, 2004[30]	Spasticity and movement related disability	Cognitive function	Depression; anxiety; self-acceptance	QoL; functional disability
Seebacher et al, 2015[55]	Gait and mobility	–	–	Fatigue
Seebacher et al, 2017[56]	Gait and mobility	–	–	QoL; fatigue
Seebacher et al, 2018[57]	Gait and mobility	–	–	QoL; fatigue
Seebacher et al, 2019[58]	Gait and mobility; coordination and synchronisation	–	–	QoL; fatigue
Seebacher et al, 2024[59]	Gait and mobility	Global cognitive function	Anxiety; depression; suicidality	QoL; fatigue
Seighalani et al, 2014[60]	–	–	Depression; anxiety; self esteem	–
Shahraki et al, 2017[61]	Gait and mobility	–	–	–
Thaut et al, 2008[62]	–	Learning and memory	–	–
Thaut et al, 2014[63]	–	Learning and memory; neural synchronisation	–	–
Wiens et al, 1999[31]	Muscle strength (respiratory)	–	–	–
Young et al, 2019[44]	Gait and mobility	–	–	Fatigue; pain

Figure 1. PRISMA 2020 flow diagram. PRISMA, Preferred Reporting Items for Systematic Reviews and Meta-Analyses

For peer review only

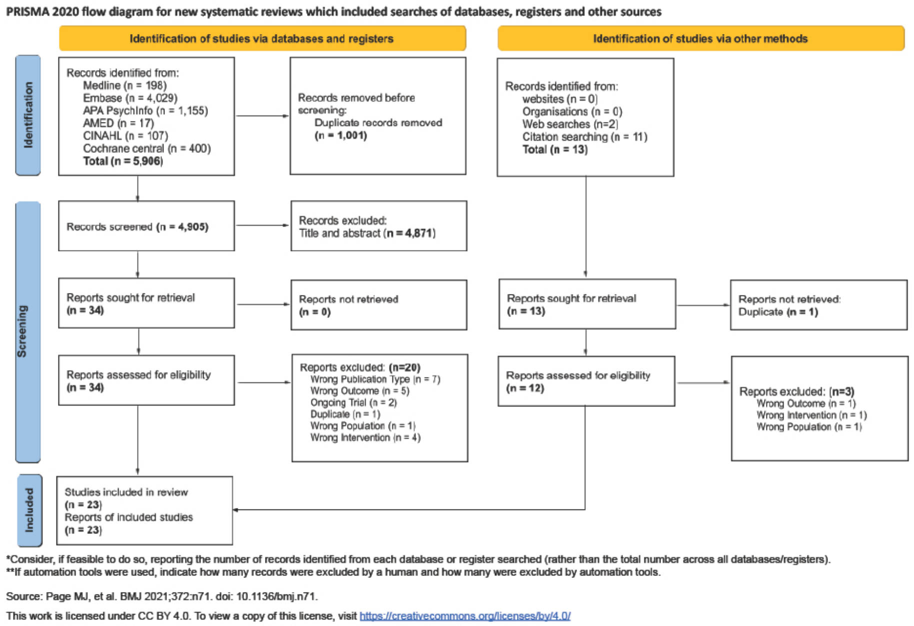

PRISMA 2020 flow diagram. PRISMA, Preferred Reporting Items for Systematic Reviews and Meta-Analyses

361x255mm (72 x 72 DPI)

Ahadi et al. Potential benefits of music-based therapies on rehabilitation outcomes in people with multiple sclerosis: a systematic review. Supplementary files. R1.

Supplemental file 1. Completed PRISMA 2020 checklist

Section and Topic	Item #	Checklist item	Location where item is reported
**TITLE**			
Title	1	Identify the report as a systematic review.	1
**ABSTRACT**			
Abstract	2	See the PRISMA 2020 for Abstracts checklist.	2
**INTRODUCTION**			
Rationale	3	Describe the rationale for the review in the context of existing knowledge.	5-6
Objectives	4	Provide an explicit statement of the objective(s) or question(s) the review addresses.	6
**METHODS**			
Eligibility criteria	5	Specify the inclusion and exclusion criteria for the review and how studies were grouped for the syntheses.	6-8
Information sources	6	Specify all databases, registers, websites, organisations, reference lists and other sources searched or consulted to identify studies. Specify the date when each source was last searched or consulted.	6, Supp file 3
Search strategy	7	Present the full search strategies for all databases, registers and websites, including any filters and limits used.	Supp file 3
Selection process	8	Specify the methods used to decide whether a study met the inclusion criteria of the review, including how many reviewers screened each record and each report retrieved, whether they worked independently, and if applicable, details of automation tools used in the process.	6-7
Data collection process	9	Specify the methods used to collect data from reports, including how many reviewers collected data from each report, whether they worked independently, any processes for obtaining or confirming data from study investigators, and if applicable, details of automation tools used in the process.	7
Data items	10a	List and define all outcomes for which data were sought. Specify whether all results that were compatible with each outcome domain in each study were sought (e.g. for all measures, time points, analyses), and if not, the methods used to decide which results to collect.	7, Table 3
	10b	List and define all other variables for which data were sought (e.g. participant and intervention characteristics, funding sources). Describe any assumptions made about any missing or unclear information.	7, Table 3
Study risk of bias assessment	11	Specify the methods used to assess risk of bias in the included studies, including details of the tool(s) used, how many reviewers assessed each study and whether they worked independently, and if applicable, details of automation tools used in the process.	7
Effect measures	12	Specify for each outcome the effect measure(s) (e.g. risk ratio, mean difference) used in the synthesis or presentation of results.	N/A – no meta-analysis

For peer review only

Section and Topic	Item #	Checklist item	Location where item is reported
Synthesis methods	13a	Describe the processes used to decide which studies were eligible for each synthesis (e.g. tabulating the study intervention characteristics and comparing against the planned groups for each synthesis (item #5)).	6-8
	13b	Describe any methods required to prepare the data for presentation or synthesis, such as handling of missing summary statistics, or data conversions.	N/A – no meta-analysis
	13c	Describe any methods used to tabulate or visually display results of individual studies and syntheses.	N/A – no meta-analysis
	13d	Describe any methods used to synthesize results and provide a rationale for the choice(s). If meta-analysis was performed, describe the model(s), method(s) to identify the presence and extent of statistical heterogeneity, and software package(s) used.	7-8
	13e	Describe any methods used to explore possible causes of heterogeneity among study results (e.g. subgroup analysis, meta-regression).	N/A – no meta-analysis
	13f	Describe any sensitivity analyses conducted to assess robustness of the synthesized results.	N/A – no meta-analysis
Reporting bias assessment	14	Describe any methods used to assess risk of bias due to missing results in a synthesis (arising from reporting biases).	N/A – no meta-analysis
Certainty assessment	15	Describe any methods used to assess certainty (or confidence) in the body of evidence for an outcome.	9
**RESULTS**			
Study selection	16a	Describe the results of the search and selection process, from the number of records identified in the search to the number of studies included in the review, ideally using a flow diagram.	Fig 1
	16b	Cite studies that might appear to meet the inclusion criteria, but which were excluded, and explain why they were excluded.	Supp file 5
Study characteristics	17	Cite each included study and present its characteristics.	Supp file 6
Risk of bias in studies	18	Present assessments of risk of bias for each included study.	Supp file 11

For peer review only

Section and Topic	Item #	Checklist item	Location where item is reported
Results of individual studies	19	For all outcomes, present, for each study: (a) summary statistics for each group (where appropriate) and (b) an effect estimate and its precision (e.g. confidence/credible interval), ideally using structured tables or plots.	No meta-analysis, relevant info in Supp file 9
Results of syntheses	20a	For each synthesis, briefly summarise the characteristics and risk of bias among contributing studies.	9-10, Suppl file 10
	20b	Present results of all statistical syntheses conducted. If meta-analysis was done, present for each the summary estimate and its precision (e.g. confidence/credible interval) and measures of statistical heterogeneity. If comparing groups, describe the direction of the effect.	N/A – no meta-analysis
	20c	Present results of all investigations of possible causes of heterogeneity among study results.	N/A – no meta-analysis
	20d	Present results of all sensitivity analyses conducted to assess the robustness of the synthesized results.	N/A – no meta-analysis
Reporting biases	21	Present assessments of risk of bias due to missing results (arising from reporting biases) for each synthesis assessed.	N/A – no meta-analysis
Certainty of evidence	22	Present assessments of certainty (or confidence) in the body of evidence for each outcome assessed.	13, Table 5
**DISCUSSION**			
Discussion	23a	Provide a general interpretation of the results in the context of other evidence.	14-15
	23b	Discuss any limitations of the evidence included in the review.	14-16
	23c	Discuss any limitations of the review processes used.	15-16
	23d	Discuss implications of the results for practice, policy, and future research.	16
**OTHER INFORMATION**			
Registration and protocol	24a	Provide registration information for the review, including register name and registration number, or state that the review was not registered.	6
	24b	Indicate where the review protocol can be accessed, or state that a protocol was not prepared.	6
	24c	Describe and explain any amendments to information provided at registration or in the protocol.	6

For peer review only

Section and Topic	Item #	Checklist item	Location where item is reported
Support	25	Describe sources of financial or non-financial support for the review, and the role of the funders or sponsors in the review.	17
Competing interests	26	Declare any competing interests of review authors.	17
Availability of data, code and other materials	27	Report which of the following are publicly available and where they can be found: template data collection forms; data extracted from included studies; data used for all analyses; analytic code; any other materials used in the review.	17

From: Page MJ, McKenzie JE, Bossuyt PM, Boutron I, Hoffmann TC, Mulrow CD, et al. The PRISMA 2020 statement: an updated guideline for reporting systematic reviews. BMJ 2021;372:n71. doi: 10.1136/bmj.n71. This work is licensed under CC BY 4.0. To view a copy of this license, visit https://creativecommons.org/licenses/by/4.0/

Synthesis Without Meta-analysis (SWiM) reporting items

The citation for the Synthesis Without Meta-analysis explanation and elaboration article is: Campbell M, McKenzie JE, Sowden A, Katikireddi SV, Brennan SE, Ellis S, Hartmann-Boyce J, Ryan R, Shepperd S, Thomas J, Welch V, Thomson H. Synthesis without meta-analysis (SWiM) in systematic reviews: reporting guideline BMJ 2020;368:l6890 http://dx.doi.org/10.1136/bmj.l6890

**SWiM is intended to complement and be used as an extension to PRISMA**			
**SWiM reporting item**	**Item description**	**Page in manuscript where item is reported**	**Other***
Methods			
**1** Grouping studies for synthesis	1a) Provide a description of, and rationale for, the groups used in the synthesis (e.g., groupings of populations, interventions, outcomes, study design)	8	
	1b) Detail and provide rationale for any changes made subsequent to the protocol in the groups used in the synthesis	N/A (no changes)	
**2** Describe the standardised metric and transformation methods used	Describe the standardised metric for each outcome. Explain why the metric(s) was chosen, and describe any methods used to transform the intervention effects, as reported in the study, to the standardised metric, citing any methodological guidance consulted	8	

For peer review only

**3** Describe the synthesis methods	Describe and justify the methods used to synthesise the effects for each outcome when it was not possible to undertake a meta-analysis of effect estimates	8	
**4** Criteria used to prioritise results for summary and synthesis	Where applicable, provide the criteria used, with supporting justification, to select the particular studies, or a particular study, for the main synthesis or to draw conclusions from the synthesis (e.g., based on study design, risk of bias assessments, directness in relation to the review question)	8	
**SWiM reporting item**	**Item description**	**Page in manuscript where item is reported**	**Other***
**5** Investigation of heterogeneity in reported effects	State the method(s) used to examine heterogeneity in reported effects when it was not possible to undertake a meta-analysis of effect estimates and its extensions to investigate heterogeneity	8	
**6** Certainty of evidence	Describe the methods used to assess certainty of the synthesis findings	8	
**7** Data presentation methods	Describe the graphical and tabular methods used to present the effects (e.g., tables, forest plots, harvest plots).<br>Specify key study characteristics (e.g., study design, risk of bias) used to order the studies, in the text and any tables or graphs, clearly referencing the studies included	8	
Results			
**8** Reporting results	For each comparison and outcome, provide a description of the synthesised findings, and the certainty of the findings. Describe the result in language that is consistent with the question the synthesis addresses, and indicate which studies contribute to the synthesis	10-13	
Discussion			
**9** Limitations of the synthesis	Report the limitations of the synthesis methods used and/or the groupings used in the synthesis, and how these affect the conclusions that can be drawn in relation to the original review question	16	

PRISMA=Preferred Reporting Items for Systematic Reviews and Meta-Analyses.
*If the information is not provided in the systematic review, give details of where this information is available (e.g., protocol, other published papers (provide citation details), or website (provide the URL)).

For peer review only

Supplemental file 2. Completed PRISMA for Abstracts 2020 checklist

Section and Topic	Item #	Checklist item	Reported (Yes/No)
**TITLE**			
Title	1	Identify the report as a systematic review.	Yes
**BACKGROUND**			
Objectives	2	Provide an explicit statement of the main objective(s) or question(s) the review addresses.	Yes
**METHODS**			
Eligibility criteria	3	Specify the inclusion and exclusion criteria for the review.	Yes
Information sources	4	Specify the information sources (e.g. databases, registers) used to identify studies and the date when each was last searched.	Yes
Risk of bias	5	Specify the methods used to assess risk of bias in the included studies.	Yes
Synthesis of results	6	Specify the methods used to present and synthesise results.	Yes
**RESULTS**			
Included studies	7	Give the total number of included studies and participants and summarise relevant characteristics of studies.	Yes
Synthesis of results	8	Present results for main outcomes, preferably indicating the number of included studies and participants for each. If meta-analysis was done, report the summary estimate and confidence/credible interval. If comparing groups, indicate the direction of the effect (i.e. which group is favoured).	Yes
**DISCUSSION**			
Limitations of evidence	9	Provide a brief summary of the limitations of the evidence included in the review (e.g. study risk of bias, inconsistency and imprecision).	Yes
Interpretation	10	Provide a general interpretation of the results and important implications.	Yes
**OTHER**			
Funding	11	Specify the primary source of funding for the review.	Yes
Registration	12	Provide the register name and registration number.	Yes

For peer review only

Supplemental file 3. Detailed search strategies

Medline – via OVID

Search date: Wednesday 16th July 2025

1. exp Multiple Sclerosis/

2. multiple sclerosis.mp.

3. MS.mp.

4. 1 or 2 or 3

5. exp Music Therapy/

6. music therap.mp.*

7. (music-based adj3 therap*).mp.

8. Music-based therapy.mp.

9. music intervention

10. music interventions.mp.

11. musical intervention.mp.

12. musical interventions.mp.

13. rhythmic auditory stimulation.mp.

14. sound therap.mp.*

15. singing therapy.mp.*

16. auditory stimulation.mp.*

17. 5 or 6 or 7 or 8 or 9 or 10 or 11 or 12 or 13 or 14 or 15

18. 4 and 17

19. limit 14 to English language

For peer review only

Results: 198

Search History (19) View Saved

#	Searches	Results	Runtime	Type	Actions	Annotations
1	exp Multiple Sclerosis/	75635	0.62	Advanced	Display Results More	
2	Multiple Sclerosis.mp.	109818	0.98	Advanced	Display Results More	
3	MS.mp.	478942	0.48	Advanced	Display Results More	
4	1 or 2 or 3	531625	0.09	Advanced	Display Results More	
5	exp Music Therapy/	4964	0.09	Advanced	Display Results More	
6	music therap*.mp.	6740	2.61	Advanced	Display Results More	
7	(music-based adj3 therap*).mp. [mp=title, book title, abstract, original title, name of substance word, subject heading word, floating sub-heading word, keyword heading word, organism supplementary concept word, protocol supplementary concept word, rare disease supplementary concept word, unique identifier, synonyms, population supplementary concept word, anatomy supplementary concept word]	103	2.42	Advanced	Display Results More	
8	music-based therapy.mp.	27	0.64	Advanced	Display Results More	
9	music intervention.mp.	700	0.34	Advanced	Display Results More	
10	music interventions.mp.	456	0.32	Advanced	Display Results More	
11	musical intervention.mp.	86	0.28	Advanced	Display Results More	
12	musical interventions.mp.	68	0.27	Advanced	Display Results More	
13	rhythmic auditory stimulation.mp.	215	0.52	Advanced	Display Results More	
14	sound therap*.mp.	458	2.16	Advanced	Display Results More	
15	singing therapy*.mp.	22	1.16	Advanced	Display Results More	
16	auditory stimulation*.mp.	2366	0.63	Advanced	Display Results More	
17	5 or 6 or 7 or 8 or 9 or 10 or 11 or 12 or 13 or 14 or 15 or 16	9799	0.02	Advanced	Display Results More	
18	4 and 17	211	0.05	Advanced	Display Results More	
19	limit 18 to english language	198	0.02	Advanced	Display Results More	

Save Remove Combine with: AND OR Contract

Embase via Ovid

Search date: Wednesday 16th July 2025

1. *exp Multiple Sclerosis/*
2. *multiple sclerosis.mp.*
3. *MS.mp.*
4. *1 or 2 or 3*
5. *exp Music Therapy/*
6. *music therap*.mp.*
7. music-based therap*.mp.

8. music intervention,mp.
9. music interventions.mp.
10. musical intervention.mp.
11. musical interventions.mp.
12. *rhythmic auditory stimulation.mp.*
13. *sound therap*.mp.*
14. *singing therap*.mp.*
15. *auditory stimulation*.mp.*
16. *5 or 6 or 7 or 8 or 9 or 10 or 11 or 12 or 13 0r 14 or 15*
17. *4 and 16*
18. *limit 17 to English language*

results: 4029

# ▲	Searches	Results	Runtime	Type	Actions		Annotations
1	exp multiple sclerosis/	176930	0.07	Advanced	Display Results	More	
2	multiple sclerosis.mp.	193474	0.77	Advanced	Display Results	More	
3	MS.mp.	664062	0.18	Advanced	Display Results	More	
4	1 or 2 or 3	754065	0.15	Advanced	Display Results	More	
5	exp music therapy/	11246	0.08	Advanced	Display Results	More	
6	music therap*.mp.	11935	4.36	Advanced	Display Results	More	
7	music-based therap*.mp.	61	4.43	Advanced	Display Results	More	
8	music intervention.mp.	1086	0.23	Advanced	Display Results	More	
9	music interventions.mp.	595	0.15	Advanced	Display Results	More	
10	musical intervention.mp.	143	0.22	Advanced	Display Results	More	
11	musical interventions.mp.	98	0.16	Advanced	Display Results	More	
12	rhythmic auditory stimulation.mp.	338	0.65	Advanced	Display Results	More	
13	sound therap*.mp.	625	4.48	Advanced	Display Results	More	
14	singing therap*.mp.	36	4.21	Advanced	Display Results	More	
15	auditory stimulation*.mp.	43773	0.43	Advanced	Display Results	More	
16	5 or 6 or 7 or 8 or 9 or 10 or 11 or 12 or 13 or 14 or 15	56404	0.02	Advanced	Display Results	More	
17	4 and 16	4168	0.07	Advanced	Display Results	More	
18	limit 17 to english language	4029	0.02	Advanced	Display Results	More	

Save Remove Combine with: AND OR Contract

For peer review only

APA PsycInfo via Ovid

Search date: Wednesday 16th July 2025

1. *exp Multiple Sclerosis/*
2. *multiple sclerosis.mp.*
3. *MS.mp.*
4. *1 or 2 or 3*
5. *exp Music Therapy/*
6. *music therap*.mp.*
7. music-based therap*.mp.
8. music intervention.mp.
9. music interventions.mp.
10. musical intervention.mp.
11. musical interventions.mp.
12. *rhythmic auditory stimulation.mp.*
13. *sound therap*.mp.*
14. *singing therap*.mp.*
15. *auditory stimulation*.mp.*
16. *5 or 6 or 7 or 8 or 9 or 10 or 11 or 12 or 13 0r 14 or 15*
17. *4 and 16*
18. *limit 17 to English language*

results: 1155

For peer review only

# ▲	Searches	Results	Runtime	Type	Actions	Annotations
1	exp Multiple Sclerosis/	15247	0.14	Advanced	Display Results More	
2	Multiple Sclerosis.mp.	19196	1.06	Advanced	Display Results More	
3	MS.mp.	37720	0.20	Advanced	Display Results More	
4	1 or 2 or 3	44700	0.01	Advanced	Display Results More	
5	exp Music Therapy/	6337	0.13	Advanced	Display Results More	
6	Music therap*.mp.	7695	0.57	Advanced	Display Results More	
7	music-based therap*.mp.	27	1.03	Advanced	Display Results More	
8	music intervention.mp.	353	0.29	Advanced	Display Results More	
9	music interventions.mp.	304	0.24	Advanced	Display Results More	
10	musical intervention.mp.	59	0.29	Advanced	Display Results More	
11	musical interventions.mp.	76	0.23	Advanced	Display Results More	
12	rhythmic auditory stimulation.mp.	83	0.58	Advanced	Display Results More	
13	sound therap*.mp.	167	0.68	Advanced	Display Results More	
14	singing therap*.mp.	13	0.73	Advanced	Display Results More	
15	auditory stimulation*.mp.	24980	0.60	Advanced	Display Results More	
16	5 or 6 or 7 or 8 or 9 or 10 or 11 or 12 or 13 or 14 or 15	32845	0.01	Advanced	Display Results More	
17	4 and 16	1175	0.01	Advanced	Display Results More	
18	limit 17 to english language	1155	0.13	Advanced	Display Results More	

Save Remove Combine with: AND OR Contract

AMED via EBSCOhost

Search date: Wednesday 16th July 2025

"multiple sclerosis" OR "MS"

AND

"music therap*" OR "music-based therap*" OR "musical intervention" OR "musical interventions" OR “music intervention” OR “music interventions” OR "rhythmic auditory stimulation*" OR "sound therap*" OR "singing therap*" OR "auditory stimulation*"

Limit = English language

Results: 17

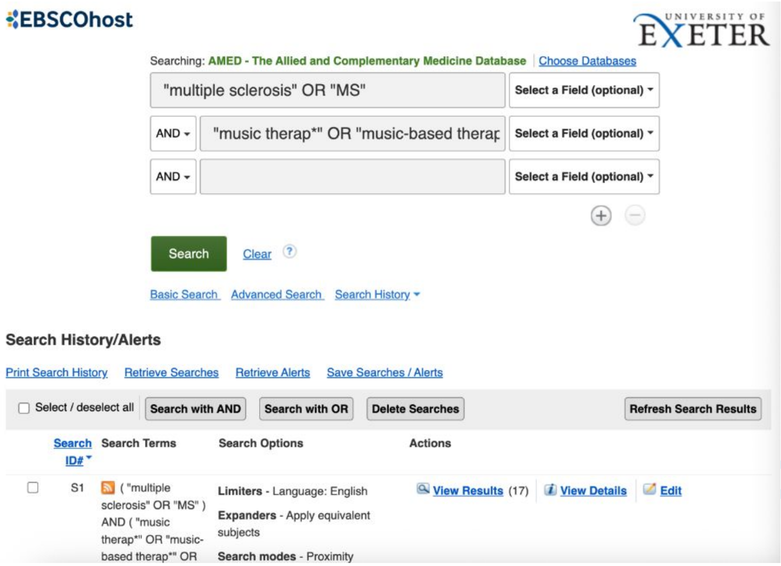

CINAHL Ultimate via EBSCOhost

Search date: Wednesday 16th July 2025

"multiple sclerosis" OR "MS"

AND

"music therap*" OR "music-based therap*" OR "musical intervention" OR "musical interventions" OR “music intervention” OR “music interventions” OR "rhythmic auditory stimulation*" OR "sound therap*" OR "singing therap*" OR "auditory stimulation*"

Limit = English language

Results: 107

For peer review only

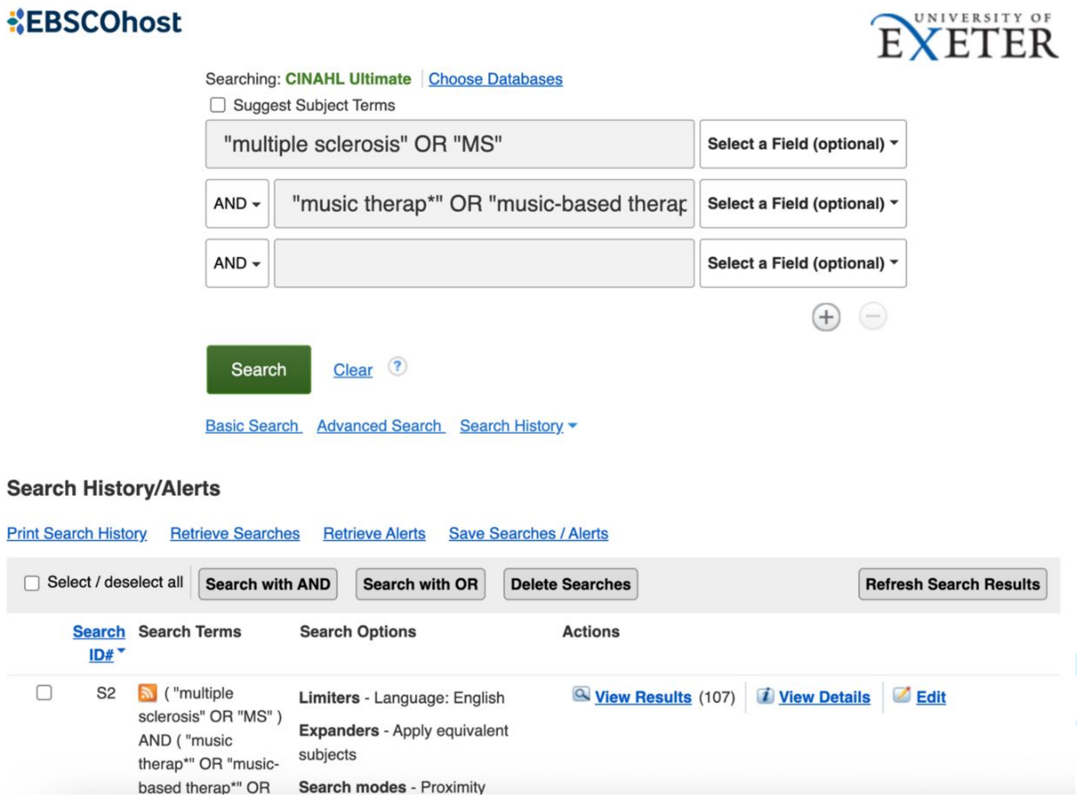

<u>COCHRANE-Central</u>

Search date: Wednesday 16th July 2025

"multiple sclerosis" OR "MS"

AND

(music therapy) OR (music-based therapy) OR (musical intervention) OR (musical interventions) OR (music intervention) OR (music interventions) OR (rhythmic auditory stimulation) OR (sound therapy) OR (singing therapy) OR (auditory stimulation)

Results: 400 trials (Trials = 392 are English and 8 non-English)

w only

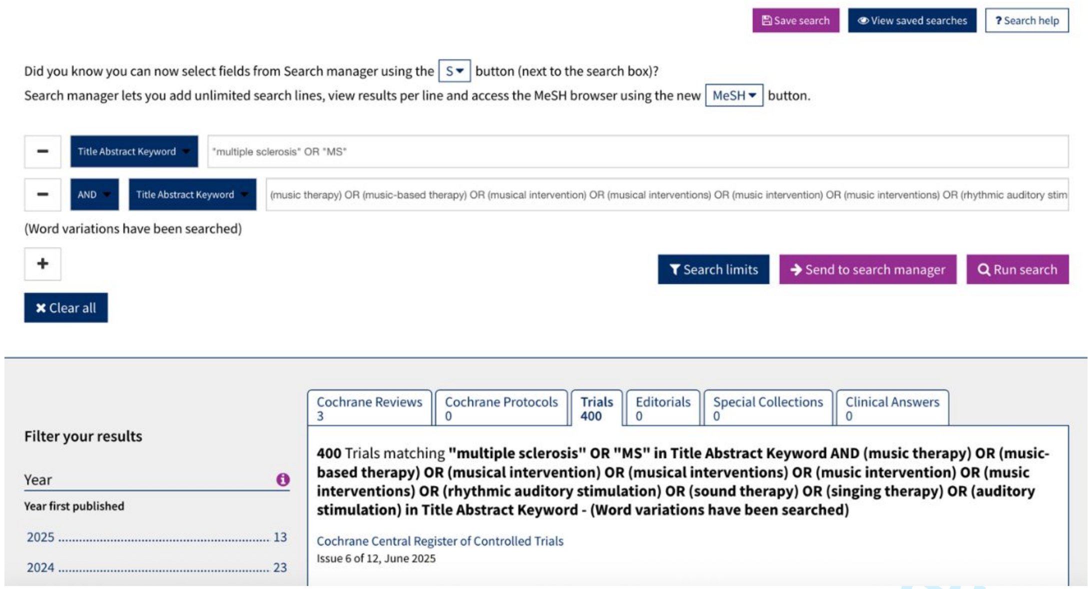
Save search
View saved searches
? Search help
Did you know you can now select fields from Search manager using the S button (next to the search box)?
Search manager lets you add unlimited search lines, view results per line and access the MeSH browser using the new MeSH button.
Title Abstract Keyword
"multiple sclerosis" OR "MS"
AND
Title Abstract Keyword
(music therapy) OR (music-based therapy) OR (musical intervention) OR (musical interventions) OR (music intervention) OR (music interventions) OR (rhythmic auditory stim
(Word variations have been searched)
Search limits
Send to search manager
Run search
Clear all
Filter your results
Year
Year first published
2025 13
2024 23
Cochrane Reviews 3
Cochrane Protocols 0
Trials 400
Editorials 0
Special Collections 0
Clinical Answers 0
400 Trials matching "multiple sclerosis" OR "MS" in Title Abstract Keyword AND (music therapy) OR (music-based therapy) OR (musical intervention) OR (musical interventions) OR (music intervention) OR (music interventions) OR (rhythmic auditory stimulation) OR (sound therapy) OR (singing therapy) OR (auditory stimulation) in Title Abstract Keyword - (Word variations have been searched)
Cochrane Central Register of Controlled Trials
Issue 6 of 12, June 2025

eview only

Supplemental file 4. Full list of included publications

1. Aldridge D, Schmid W, Kaeder M, et al. Functionality or aesthetics? A pilot study of music therapy in the treatment of multiple sclerosis patients. *Complement Ther Med* 2005; 13(1): 25–33.
2. Conklyn D, Stough D, Novak E, et al. A home-based walking program using rhythmic auditory stimulation improves gait performance in patients with multiple sclerosis: a pilot study. Neurorehabil Neural Repair 2010; 24(9): 835-42.
3. Gatti R, Tettamanti A, Lambiase S, et al. Improving hand functional use in subjects with multiple sclerosis using a musical keyboard: a randomized controlled trial. Physiother Res Int 2015; 20: 100-7.
4. Goetschalckx M, Van Geel F, Meesen R, et al. Rhythmic interlimb coordination of the lower limbs in multiple sclerosis during auditory pacing to three different frequencies. Gait Posture 2021; 334-40.
5. Helminger B, Seebacher B, Ropele S, et al. Effects of rhythmic-cued gait training on gait-like task related brain activation in people with multiple sclerosis. J Neurol Sci 2025; 471: 123426.
6. Impellizzeri F, Leonardi S, Latella D, et al. An integrative cognitive rehabilitation using neurologic music therapy in multiple sclerosis: a pilot study. Medicine 2020: 99(4): e18866.
7. Kuhlmann J, Ebner K, Zimmer A, et al. Music therapy with a monochord in multiple sclerosis (“MUTIMS”): A randomized, controlled, rater-blinded trial. Mult Scler J Exp Transl Clin 2025; 11(3):20552173251352712.
8. Maggio MG, Tripoli D, Porcari B, et al. How may patients with MS benefit from using music assisted therapy? A case-control feasability study investigating motor outcomes and beyond. Mult Scler Relat Disord 2021; 48: 102713.
9. Moore KS, Peterson DA, O’Shea G, et al. The effectiveness of music as a mnemonic device on recognition memory for people with multiple sclerosis. J Music Ther 2008; 45(3): 307-29.
10. Moumdjian L, Moens B, Maes PJ, et al. Continuous 12 min walking to music, metronomes and in silence: auditory-motor coupling and its effects on perceived fatigue, motivation and gait in persons with multiple sclerosis. Mult Scler Relat Disord 2019; 35: 92-9.
11. Naderi S, Sadeghi H, Amirseyfaddini M. The effects of rhythmic auditory stimulation with different tempos on spatio-temporal parameters and gait symmetry in patients with multiple sclerosis: a quasi-experimental study. J Res Rehabil Sci 2022; 18: doi: 10.48305/jrrs.2023.41771.1041.
12. Schmid W, Aldridge D. Active music therapy in the treatment of multiple sclerosis patients: a matched control study. J Music Ther 2004; 41(3): 225-40.
13. Seebacher B, Helmlinger B, Pinter D, et al. Actual and imagined music-cued gait training in people with multiple sclerosis: a double-blind randomized parallel multicenter trial. Neurorehabil Neural Repair 2024; 38(8): 555-69.
14. Seebacher B, Kuisma R, Glynn A, et al. Effects and mechanisms of differently cued and non-cued motor imagery in people with multiple sclerosis: a randomised controlled trial. Mult Scler J 2019; 25(12): 1593-1604.
15. Seebacher B, Kuisma R, Glynn A, et al. Exploring cued and non-cued motor imagery interventions in people with multiple sclerosis: a randomised feasibility trial and reliability study. Arch Physiother 2018; 8:6.
16. Seebacher B, Kuisma R, Glynn A, et al. The effect of rhythmic-cued motor imagery on walking, fatigue and quality of life in people with multiple sclerosis: a randomised controlled trial. Mult Scler J 2017; 23(2): 286-96.
17. Seebacher B, Kuisma R, Glynn A, et al. Rhythmic cued motor imagery and walking in people with multiple sclerosis: a randomised controlled feasibility study.
    Pilot Feasibility Stud 2015; 1: 25.
18. Seighalani MZ, Ghahari S, Zarbakhsh M. The effectiveness of music therapy on depression, anxiety and self-esteem of patients with multiple sclerosis (MS). Adv Environ Biol 2014; 8(9): 253-60.

For peer review only

19. Shahraki M, Sohrabi M, Taheri Torbati HR, et al. Effect of rhythmic auditory stimulation on gait kinematic parameters of patients with multiple sclerosis. J Med Life 2017; 10(1): 33-7.
20. Thaut MH, Peterson DA, Sena KM, et al. Musical structure facilitates verbal learning in multiple sclerosis. *Music Percept* 2008; 25(4): 325–30.
21. Thaut MH, Peterson DA, McIntosh GC, et al. Music mnemonics aid verbal memory and induce learning – related brain plasticity in multiple sclerosis. Front Hum Neurosci 2014; 8: 395.
22. Wiens ME, Reimer MA, Guyn HL. Music therapy as a treatment method for improving respiratory muscle strength in patients with advanced multiple sclerosis: a pilot study. Rehabil Nurs 1999; 24(2): 74-80.
23. Young HJ, Mehta TS, Herman C, et al. The effects of M2M and adapted yoga on physical and psychosocial outcomes in people with multiple sclerosis. Arch Phys Med Rehabil 2019;100(3):391-400.

List of studies for each outcome domain (studies can contribute to multiple outcome domains)

<u>Motor</u>

1. Conklyn et al. 2010
2. Gatti et al. 2015
3. Goetschalckx. et al. 2021
4. Helminger. et al. 2025
5. Maggio et al. 2021
6. Moumdjian et al. 2019
7. Naderi et al 2022
8. Schmid et al, 2004
9. Seebacher et al. 2015
10. Seebacher et al. 2017
11. Seebacher et al. 2018
12. Seebacher et al. 2019
13. Seebacher et al. 2024
14. Shahraki et al, 2017
15. Wiens et al. 1999
16. Young et al. 2019

<u>Cognitive</u>

1. Aldridge et al. 2005
2. Impellizzeri et al. 2020
3. Moore et al. 2008
4. Moumdjian et al. 2019
5. Schmid et al, 2004
6. Seebacher et al. 2024
7. Thaut et al 2008

For peer review only

8. Thaut et al 2014

Psychological

1. Aldridge et al. 2005
2. Impellizzeri et al. 2020
3. Kuhlmann et al. 2025
4. Maggio et al. 2021
5. Schmid et al. 2004
6. Seebacher et al. 2024
7. Seighalani et al. 2014

Quality of life

1. Aldridge et al. 2005
2. Concklyn et al 2010
3. Impellizzeri et al. 2020
4. Kuhlmann et al. 2025
5. Maggio et al. 2021
6. Schmid et al. 2004
7. Seebacher et al. 2015
8. Seebacher et al. 2017
9. Seebacher et al. 2018
10. Seebacher et al. 2019
11. Seebacher et al. 2024
12. Young et al. 2019

For peer review only

Supplemental file 5. Full list of studies excluded at full text review with reasons

Duplicate

1. Lambiase S, Nava C, Tettamanti A, et al. Improving hand functional use in subjects with multiple sclerosis using a musical keyboard: a randomized controlled trial. Physiother Res Int 2015;20: 100-7. [This is Gatti et al, 2015, included study].
2. Moore KS, Peterson DA, O'Shea G, et al. The effectiveness of music as a mnemonic device on recognition memory for people with multiple sclerosis. *J Music Ther* 2008; 45(3): 307–29.

Intervention

3. Baram Y, Miller A. Auditory feedback control for improvement of gait in patients with multiple sclerosis. J Neurol Sci 2007; 254: 90-4.
4. Basturk S, Ekici G, Kirteke F, et al. Therapeutic effects of line dancing in people with multiple sclerosis: an evaluator-blinded, randomized controlled study. Art Health 2024; doi: 10.1080/17533015.2024.2325425.
5. Mandelbaum R, Triche EW, Fasoli SE, et al. A pilot study: examining the effects and tolerability of structured dance intervention for individuals with multiple sclerosis. *Disabil Rehabil* 2016; 38(3): 218–22
6. Trinchillo A, Caliendo D, Nicolella V, et al. Tango classes in people with Multiple Sclerosis (PwMS): Impact on motor and non-motor functions. Clin Neurol Neurosurg 2024; 243: 108380.
7. Van Geel F, Van Asch P, Veldkamp R, et al. Effects of a 10-week multimodal dance and art intervention program leading to a public performance in persons with multiple sclerosis: a controlled pilot-trial. Mult Scler Relat Disord 2020; 44: 102256.

Outcome

8. Fasching B, Mildner S, Fink F, et al. MuSic Moves-co-creating a music-supported exercise programme with and for people with multiple sclerosis: a bicentre participatory mixed methods study. BMJ Open 2024; 14(11): e091168.
9. Moumdjian L, Maes PJ, Dalla Bella S, et al. Detrended fluctuation analysis of gait dynamics when entraining to music and metronomes at different tempi in persons with multiple sclerosis. Sci Rep 2020; 10: 12934.
10. Moumdjian L, Moens B, Maes PJ, et al. Walking to music and metronome at various tempi in persons with multiple sclerosis: a basis for rehabilitation. Neurorehabil Neural Repair 2019; 33(6): 464-75.
11. Seebacher B, Helmlinger B, Hotz I, et al. Actual and imagined music-cued gait training for people with multiple sclerosis: a multicentre qualitative study. BMJ Open 2024; 14: e086555.
12. Seighalani MZ, Cheraghali Gol H, Rostami AM. The effectiveness of music therapy on depression, anxiety and self-esteem of patients with multiple sclerosis. *Adv Environ Biol* 2014; 8(10): 759–64.
13. Vanbilsen N, Moumdjian L, Kinnaert F, et al. Step to the beat: Auditory-motor coupling during walking to higher and lower tempi with music and metronomes in progressive multiple sclerosis: an observational study. Mult Scler Relat Disord 2024; 92: 106152.

Population

14. Srinithiwat P, Foocharoen C, Mahakkanukrauh A, et al. Patient perspectives and satisfaction following a short-term singing intervention in systemic sclerosis patients: a cross-sectional study. *J Med Assoc Thai* 2025; 108: S59–65.

For peer review only

15. Young HJ, Lai B, Wilroy J, et al. Effects of a teleexercise movement-to-music intervention on health outcomes in individuals with mobility disabilities: a randomized controlled trial. Arch Phy Med Rehabil 2025; https://doi.org/10.1016/j.apmr.2025.05.017.

<u>Publication type</u>

16. https://clinicaltrials.gov/study/NCT05792176. Ukulele playing to improve cognition in people with multiple sclerosis: a feasibility study [No results posted].
17. https://clinicaltrials.gov/study/NCT04019912. The novel use of treadmill plus music in MS patients gate rehabilitation (SMUSIC) [No results posted – spelling mistake 'gate' for 'gait' was made on Clinical Trials.gov].
18. https://clinicaltrials.gov/study/NCT04314076. Rhythmic auditory stimulation & gait training [No results posted].
19. https://clinicaltrials.gov/study/NCT01086371. Rhythmic auditory stimulation and walking performance in multiple sclerosis (MS) patients [No results posted].
20. https://clinicaltrials.gov/study/NCT06800144. Effect of music therapy on pain in people with multiple sclerosis [No results posted].
21. https://trialsearch.who.int/Trial2.aspx?TrialID=IRCT20230219057455N1. The effectiveness of slow and fast music on anxiety and quality of life of people with multiple sclerosis [No results posted].
22. https://trialsearch.who.int/Trial2.aspx?TrialID=DRKS00020469. Effectiveness of playing the instruments „Big Bom"and "Temple Blocks" as a music therapy approach to improve gait function among patients with multiple sclerosis [No results posted].
23. Bethoux F, Awad L, Gallagher L, et al. Safety and acceptability of closed-loop rhythmic cueing for gait training in persons with multiple sclerosis: a pilot single-blind randomized controlled trial. Mult Scler J 2023: 29(3): 645 [Abstract].
24. Pedulla L, Addeo S, Rinaldi S, Aerobic training in multiple sclerosis: a pilot study on the effects of music during group treatment. Mult Scler J 2020; *26* (Suppl 2): 54-55 [Abstract].

For peer review only

Supplemental file 6. Study characteristics for included studies.

Author, year	Country	Design	Participants	Inclusion criteria	Exclusion criteria	Outcomes
Aldridge et al. 2005	Germany	Matched controlled pilot study	20 people with MS (10 per arm). 30% male Age range: 29-47	Diagnosis of multiple sclerosis and within the EDSS range used for matching	Pregnancy and mental disorders requiring medication	Primary: Psychological outcomes such as Depression (Beck, HADS), Anxiety (HADS), Self-acceptance (SESA) Secondary: Quality of life (HAQUAMS) Functional disability (EDSS) Cognitive (MSFC) and motor function (MSFC) Fatigue (IFSS)
Conklyn et al, 2010	USA	RCT	10 people with MS (5 per arm), 30% male, Mean age: intervention 47, control 50	Age 18 or over; medical diagnosis of MS; able to walk 30.48m without physical assistance (assistive devices were allowed); able to walk 7.62m in 8 to 60s [distances were published in imperial feet]	Treatment for MS exacerbation in past 30 days; severe comorbidity precluding participation; severe cognitive deficits precluding informed consent or preventing following study instructions	Primary: Gait parameters<br><br>Secondary: Spasticity (MAS Modified Ashworth Scale); muscle strength (Manual Muscle Testing); Pain (out of 10); Ambulation disability (Ambulation Index); MS-related disability

For peer review only

						(Patient-determined Disease Steps); Treatment efficacy (Subject Global Impression, Clinician Global Impression)
Gatti et al, 2015	Italy	RCT	19 people with MS (9 intervention, 10 control), 37% male, mean age intervention 43, control 48	Adults; primary or secondary progressive MS; no relapse or medication changes in past 3 months; hand strength below normal; Nine-Hole Peg Test >18s; no experience playing a musical instrument; no cognitive or psychiatric disorders; no hearing or visual deficits	No additional criteria	Primary: Perceived hand functional use (ABILHAND)<br><br>Secondary: Hand dexterity (Nine-Hole Peg Test); Hand strength (Jamar and Pinch)
Goetschalckx et al, 2021	Belgium	Observational cross-sectional comparative study	38 people with MS (16% male, mean age 49).13 healthy controls (38% male, mean age 52). Note that the 'controls' were not a comparator group in terms of intervention – they received the same MBTs, the only difference was they did not have MS	MS diagnosis (McDonald criteria); age 18-70; able to walk independently	Other medical conditions influencing walking	Primary: Bilateral gait coordination (Phase Coordination Index)<br><br>Secondary: movement amplitude; movement frequency

For peer review only

Helminger et al, 2025	Austria	Secondary analysis of a randomised controlled trial (longitudinal interventional study)	48 people with MS (52% male, mean age 44). 17 health controls (53% male, mean age 41)	Age at least 18; definite MS (Thompson criteria); mild-to-moderate disability (Expanded Disability Status Scale 2-5);	Relapse or change in disease-modifying treatment or physiotherapy in past 3 months; concomitant diseases; MRI contraindications	Primary: Walking function (2-minute walk test; timed 25-foot walk)<br><br>Secondary: fMRI brain activation changes during movement tasks; correlation of brain activation changes with walking performance
Impellizzeri et al, 2020	Italy	RCT	30 people with MS (15 per arm), 63% male, mean age intervention 52, control 51	MS diagnosis (Lublin criteria); Expanded Disability Status Scale 3-7; enjoy music; no visual or hearing loss; no severe medical and psychiatric illness	No additional criteria	Cognition (Brief Repeatability Battery of Neurophysiology); quality of life (MSQoL-54); depression (Beck); emotions (Emotional Awareness Questionnaire)
Kuhlmann et al, 2025	Switzerland	RCT	57 people with MS (30 intervention, 27 control), 18% male, mean age 50	Aged at least 18; MS diagnosis (McDonald criteria); Expanded Disability Status Scale no more than 6.5	Severe psychiatric disorders; other life-threatening or severely disabling physical disorders; >2 MS relapses in past year or relapse in past 3 months; change in disease-modifying treatment or symptomatic MS medication in past 3	Primary: Anxiety (HADS)<br><br>Secondary: Depression (HADS); fatigue (self-report); pain (quantitative sensory testing); body perception (self-report)

For peer review only

					months; pregnancy; unable to lie supine for 15 min; severely compromised hearing (uncorrected); numbness on the back of both hands	
Maggio et al, 2021	Italy	Quasi-experimental controlled feasibility study	20 people with MS (10 per arm), 35% male	Secondary progressive MS (Lublin criteria); Expanded Disability Status Scale 3-6; enjoy music; no hearing or visual loss; no change in disease-modifying drugs in past 6 months	Age <65; other severe medical conditions or psychiatric illnesses; history of neuroplasms or other neurological conditions; moderate-severe cognitive decline (MoCA <21); neurologic music therapy in past 3 months	Motor evaluation (Berg Balance; Timed Up and Go; 10-metre walk test); neuropsychological battery (Beck depression; MSQOL-54 quality of life; Goal Attainment Scaling); usability (System Usability Scale)
Moore et al, 2008	USA	RCT	38 people with MS (20 intervention, 18 control), 21% male, mean age 53	Right-handed; non-smoker; Extended Disability Status Scale 3.5-7; at least 5 MS brain lesions on MRI; stable on immunomodulatory therapy; less than 2 exacerbations in past 12 months	Active exacerbation phase; treatment with pulse-cortical steroids in past 3 months; history of seizure disorder, traumatic brain injury or substance abuse; taking cognition-enhancing acetylecho-linesterase inhibitors	Verbal learning and memory (adapted Auditory-Visual Learning Test) – sensitivity index; response bias index

Moumdjian et al, 2019	Belgium	Case-control study	31 people with MS (26% male, mean age 53). 30 healthy controls (27% male, mean age 52). Note that only 27 people with MS and 30 healthy controls completed the experimental session. Note that the 'controls' were not a comparator group in terms of intervention – they received the same MBTs, the only difference was they did not have MS	Aged at least 18; diagnosis of MS for over 1 year; no exacerbation in past month; average comfortable walking speed 0.4-1.2 m/s	Pregnancy; hearing impairment; cognitive impairment hindering understanding of instructions	Primary: Auditory-motor coupling and synchronisation<br><br>Secondary: Perceived physical and cognitive fatigue (Modified Fatigue Impact Scale); motivation (Likert); cadence; stride length; velocity
Naderi et al, 2022	Iran	Quasi-experimental controlled feasibility study	13 people with MS (0% male, mean age 38). 14 healthy controls (0% male, mean age 38). Note that the 'controls' were not a comparator group in terms of intervention – they received the same MBTs, the only difference was they did not have MS	MS confirmed by neurologist; Expanded Disability Status Scale 3.5-5.5; no vision or hearing problems; no other active treatment; able to walk without assistive devices	Unable to perform tasks; severe attacks or relapse on day of the test	Primary: Gait (Gait Symmetry Index) – step duration symmetry; stride length symmetry<br><br>Secondary: Additional gait parameters
Schmid et al, 2004	USA	Matched control study	20 people with MS (10 per arm), 30%	Not specifically stated (only exclusion criteria)	Pregnancy; mental disorders requiring medication; previous	Depression (Beck); anxiety (HADS); self-acceptance (Social

For peer review only

			male, age range 29-47		experience of music therapy	and Emotional Skills Assessment); quality of life (Hamburg Quality of Life Questionnaire in MS); cognitive and functional disability (Expanded Disability Status Scale)
Seebacher et al, 2024	Austria	RCT	127 people with MS (split 43, 44, 40 across the 3 music therapy arms), 25% male, mean age per arm range 47-50	Age 18 or over; MS diagnosis (McDonald criteria); Expanded Disability Status Scale 2-5; stable disease	Cognitive impairment (MoCA <26), anxiety or depression (HADS subscales 11 or over); suicidality; relapse, starting new medication, medication adjustment or physiotherapy in past 3 months or during the study	Primary: Walking speed (timed 25-foot walk); change in walking distance (2-minute walk test)<br><br>Secondary: Cognition (MoCA); anxiety and depression (HADS); suicidality (narrative screening); fatigue (Neurological Fatigue Index-MS); quality of life (MS International Quality of Life); motor imagery (Kinesthetic and Visual Imagery Questionnaire-10)
Seebacher et al, 2019	Austria	RCT	59 people with MS (split 19, 20, 20 across the 3 music therapy arms), 20% male, mean age 45	Aged 18 or over; German speaking; clinically definite MS (McDonald criteria); mild-to-moderate disability (Expanded Disability Status Scale 1.5-4.5)	Concomitant diseases affecting interventions or walking; relapse in past 3 months or during the study; change of treatment in past 2 months or	Primary: Walking speed (timed 25-foot walk); change in walking distance (2-minute walk test)<br>Secondary: Fatigue (Modified Fatigue Impact Scale); quality

For peer review only

					during the study; pregnancy; clinical symptoms of depression or cognitive dysfunction	of life (MS Impact Scale-29); motor imagery (Kinesthetic and Visual Imagery Questionnaire-10); sensory motor synchronisation
Seebacher et al, 2018	Austria	Feasibility RCT	15 people with MS (split 5, 5, 5 across the 3 music arms), 13% male, mean age 52	Age 18 or over; MS (McDonald criteria); mild-to-moderate disability (Expanded Disability Status Scale 1.5-4.5); German speaking	Concomitant diseases affecting rhythmic cued motor imagery or walking; relapse in past 3 months or during the study; change of medication or start of physiotherapy in past 2 months; pregnancy; overt symptoms of depression or cognitive dysfunction	Primary: Feasibility; safety and adverse events<br><br>Secondary: Walking speed (timed 25-foot walk); walking distance (6-minute walk test); fatigue (Modified Fatigue Impact Scale); quality of life (MS Impact Scale-29); motor imagery (Kinesthetic and Visual Imagery Questionnaire-10)
Seebacher et al, 2017	Austria and UK	RCT	101 people with MS (34 for each of the 2 music arms, 33 controls), 16% male, mean age 44	Aged 18 or over; clinically definite MS (McDonald criteria); mild-to-moderate disease (Expanded Disability Status Scale 1.5-4.5)	Concomitant diseases affecting interventions or walking; relapse in past 3 months; change of medication or physiotherapy in past 2 months; pregnancy; overt symptoms of depression or cognitive dysfunction	Primary: Walking speed (timed 25-foot walk); change in walking distance (2-minute walk test)<br><br>Secondary: Walking perception (MS Walking Scale-12); fatigue (Modified Fatigue Impact Scale); quality of life

For peer review only

						(MS Impact Scale-29)
Seebacher et al, 2015	Austria	Feasibility RCT	30 people with MS (10 for each of the 2 music arms, 10 controls), 27% male, mean age per arm ranged 42-47	Aged 18 or over; diagnosis of MS (McDonald criteria); mild-to-moderate disability (Expanded Disability Status Scale 1.5-4.5); German speaking	Concomitant diseases affecting rhythmic cued motor imagery or walking; relapse in past 3 months or during the study; medication change in past 2 months; pregnancy; overt symptoms of depression or cognitive dysfunction	Primary: Feasibility; fatigue (Modified Fatigue Impact Scale)<br><br>Secondary: Walking speed (timed 25-foot walk); change in walking distance (2-minute walk test)
Seighalani et al, 2014	Iran	RCT	30 people with MS (15 per arm), gender and age not reported	MS diagnosis by physician; self-esteem below average (Cooper Smith <25)	Cooper Smith self-esteem 25 or above	Depression (Beck); anxiety (Beck); self-esteem (Cooper Smith)
Shahraki et al, 2017	Iran	RCT	18 people with MS (9 per arm), 22% male, mean age intervention 40, control 38	Age 18 or over; Expanded Disability Status Scale 3-6; able to walk 30.48m without assistance; lack of treatment for relapse or MS exacerbation in past 30 days; lack of cardiovascular and rheumatic diseases; lack of severe pain in lower joints; no engagement in regular physical	No additional criteria	Gait parameters

For peer review only

				activity in past 3 months; no hearing impairment		
Thaut et al, 2008	USA	RCT	20 people with MS (10 per arm), 10% male, mean age: intervention 51.7, control 53.8	Right-handed; relapse-remitting MS; normal hearing; no history of other neurological or psychiatric conditions	No additional criteria	Verbal learning and word order memory Recall of higher order word sequences (3–7 words)
Thaut et al, 2014	Germany and USA	RCT	54 people with MS (27 intervention, 27 control), 30% male, mean age intervention 50, control 53	Right-handed; relapse-remitting MS; normal hearing; no history of other neurological or psychiatric conditions; at least 5 brain lesions on MRI; stable on immunomodulatory therapy; <2 exacerbations in past 12 months	In active exacerbation phase; treated with pulse-cortical steroids or cognition-enhancing AChE inhibitors	Learning; memory; brain activity with oscillatory network synchronisation
Wiens et al, 1999	Canada	RCT	19 people with MS (9 intervention, 10 control), gender not reported, mean age 55	Residents in a long-term care centre with MS; able to tolerate 30 minutes of concentrated effort; able to follow verbal instructions; MMSE 20 or above; free from respiratory infection; able to sit in a wheelchair for at least 2 hours	No additional criteria	Strength of respiratory muscles (mouth pressure testing) – inspiratory and expiratory

For peer review only

Young et al, 2019	USA	RCT	81 people with MS (53 intervention across 2 arms, 28 control), 19% male, mean age per arm ranged 47-50	Aged 18-65; self-reported MS diagnosis; Patient-Determined Disease Steps 0-6; able to exercise with arms and/or legs; physician clearance	Participation in similar intervention in past 6 months; use of tobacco in past 6 months; unstable weight; cognitive impairment (MMSE <24); active pressure ulcer; contraindications to exercise (American College of Sports Medicine guidelines)	Primary: Motor evaluation (Timed Up and Go; 6-minute walk test; 5-times sit-to-stand)<br><br>Secondary: Fatigue (Patient-Reported Outcomes Measurement Information System Fatigue); pain (Pain Interference Short Form 8a.)

For peer review only

Supplemental file 7. Intervention profile for included studies.

Author, year	Content	Leader	Location	Duration
Aldridge et al. 2005	Nordoff Robbins approach: Active music therapy - Music-making on instruments, or singing	Therapist	Health facility	3 blocks of music therapy in single sessions over the course of the project (8—10 sessions, respectively)
Conklyn et al, 2010	Rhythmic auditory stimulation	Self	Home	2 sessions of 20 min/day for 4 weeks
Gatti et al, 2015	Playing keyboard (numbered, coloured stickers to guide)	Physiotherapist	Health facility	30 minutes a day, 5 days a week for 3 weeks
Goetschalckx et al, 2021	Rhythmic auditory stimulation with metronome	Facilitator	Health facility	1 single session (each metronome condition lasted 1 minute with a 1-minute rest in between conditions)
Helminger et al, 2025	Actual and/or imagined gait training with rhythmic-auditory cueing	Self	Home	4 sessions of 30 minutes per week for 4 weeks
Impellizzeri et al, 2020	Neurologic music therapy (plus conventional cognitive rehabilitation)	Music therapist	Health facility	3 sessions of 60 minutes per week for 8 weeks
Kuhlmann et al, 2025	Listening to a monochord being played while lying on top of it and feeling the vibration	Music therapist	Health facility	1 session of 45 minutes per week for 6 weeks
Maggio et al, 2021	Listening to music while walking on a treadmill	Physiotherapist	Health facility	3 sessions of 30 minutes per week for 8 weeks
Moore et al, 2008	Hearing word lists in song format and being asked to sing them back	Research team	Health facility	Single session (testing time approximately 60 minutes)

For peer review only

Moumdjian et al, 2019	Rhythmic auditory stimulation (2 groups: music and metronome)	Research team	Health facility	Single session of 12 minutes
Naderi et al, 2022	Rhythmic auditory stimulation	Facilitator	Health facility	Single session (4 walking modes, each 2 minutes walking with 30s rhythm exposure beforehand, and 3-minute rest between modes)
Schmid et al, 2004	Active music therapy (singing or instruments)	Music therapist	Health facility	3 blocks of 8-10 sessions over 1 year
Seebacher et al, 2024	3 groups: cued motor imagery; combined cued motor imagery and cued gait training; cued gait training	Physiotherapist, occupational therapist, psychologist	Home	4 sessions of 30 minutes (including short breaks) per week for 4 weeks
Seebacher et al, 2019	3 groups: music and verbally cued motor imagery; music cued motor imagery; non-cued motor imagery	Self	Home	6 sessions of 17 minutes per week for 4 weeks
Seebacher et al, 2018	3 groups: music and verbally cued motor imagery; music cued motor imagery; non-cued motor imagery	Self	Home	6 sessions of 17 minutes per week for 4 weeks
Seebacher et al, 2017	2 groups: music cued motor imagery; metronome cued motor imagery	Self	Home	6 sessions of 17 minutes per week for 4 weeks
Seebacher et al, 2015	2 groups: music cued motor imagery; metronome cued motor imagery	Self	Home	6 sessions of 17 minutes per week for 4 weeks
Seighalani et al, 2014	Active music therapy (discussion about music; making music with mouth; expressing the effects of music) and passive music therapy (listening to music)	Researcher and music therapist	Health facility	15 sessions of 30 minutes in total (6 active and 9 passive)

For peer review only

Shahraki et al, 2017	Rhythmic auditory stimulation with metronome	Coach	Health facility	3 sessions of 30 minutes per week for 3 weeks
Thaut et al, 2008	Ray's Auditory Verbal Learning Test – sung word lists	Research team	Health facility	Single session
Thaut et al, 2014	Ray's Auditory Verbal Learning Test – sung word lists	Research team	Health facility	Single session
Wiens et al, 1999	Music therapy: focusing on singing: relaxation and diaphragmatic breathing, intonation of syllables, and reading or singing phrases, paragraphs, and simple songs	Music therapist with instrument speciality in voice	Health facility	3 sessions of 30 minutes per week for 12 weeks
Young et al, 2019	2 groups: music to movement; adapted yoga. The yoga group does not meet the inclusion criteria as an intervention for this review and is not presented as a comparator.	Self	Fitness facility	3 sessions of 60 minutes per week for 12 weeks

For peer review only

Supplemental file 8. Control profile for included studies.

**Author, year**	**Synopsis of control arm**
Aldridge et al. 2005	No Intervention
Conklyn et al, 2010	No intervention
Gatti et al, 2015	Playing the keyboard with it turned off
Goetschalckx et al, 2021	Healthy control group who had the same intervention
Helminger et al, 2025	Healthy control group who had no music therapy
Impellizzeri et al, 2020	6 sessions per week for 8 weeks of conventional cognitive rehabilitation
Kuhlmann et al, 2025	Patients laid on top of a monochord and remained relaxed in a supine position in silence
Maggio et al, 2021	Conventional cognitive rehabilitation (same amount of conventional physiotherapy, stretching, muscle strength and occupational therapy as intervention group)
Moore et al, 2008	Spoken word group – learn and recall words without music
Moumdjian et al, 2019	Healthy control group who had no music therapy or metronome
Naderi et al, 2022	Healthy control group who had the same intervention
Schmid et al, 2004	No intervention
Seebacher et al, 2024	[No control arm]
Seebacher et al, 2019	[No control arm]
Seebacher et al, 2018	[No control arm]
Seebacher et al, 2017	No music therapy
Seebacher et al, 2015	No music therapy
Seighalani et al, 2014	No intervention
Shahraki et al, 2017	Exercises as per intervention group but without rhythmic auditory stimulation
Thaut et al, 2008	Spoken word list (same words as sung by intervention group)
Thaut et al, 2014	Spoken 15-word list (same words as sung by intervention group)
Wiens et al, 1999	3 sessions of 30 minutes per week for 12 weeks with a nurse – listened to music, discussed music, learnt about music and composers (but no active music therapy)

For peer review only

Young et al, 2019	No active music therapy or yoga

For peer review only

Supplementary file 9. Narrative results for included studies

Author, year	Results
Aldridge et al. 2005	There were no statistically significant differences between the music therapy group and the control group; however, medium effect sizes were observed for self-esteem (d = 0.54), depression (HAD-D d = 0.63), and anxiety (HAD-A d = 0.63). Within the music therapy group, significant improvements in self-esteem, depression, and anxiety were seen over the treatment period (U1–U4). No statistically significant improvement in quality of life was observed, although a trend towards improvement was reported (HAQUAMS p = 0.066). No differences were found for the functional and physiological values (MSFC, EDSS).
Conklyn et al, 2010	Compared to no intervention, rhythmic auditory stimulation was associated in statistically significant greater improvement in double-support time (left, p = .0176; right, p = .0247). No statistically significant between-group differences were found for the other outcome measures. However, trends with medium to high effect sizes were observed for other gait parameters including walking speed. Within-group analysis showed statistically significant improvement in cadence, stride length, step length, velocity, and normalized velocity. Participant satisfaction level with the rhythmic auditory stimulation intervention was high.
Gatti et al, 2015	Compared to playing with the keyboard turned off, playing with the keyboard turned on was associated with a statistically significant improvement in hand function (p=0.003, Cohen's d = 1.66), while both groups showed a statistically significant improvement in hand dexterity, although between-group differences were not statistically significant. There was also no statistically significant between-group difference in hand strength.
Goetschalckx et al, 2021	Reported results that people with MS performed more poorly than healthy controls were not surprising and outside the scope of the present review. Within people with MS, interlimb coordination was worse at 0.75Hz metronome frequency compared to 1.00Hz and 1.50Hz. Movement amplitude was greatest at 1.00 Hz but reduced at both 0.75Hz and 1.50HZ. Movement frequency followed the metronome tempo. Poorer coordination was statistically significantly correlated with poorer cognitive performance at 0.75Hz and 1.50Hz metronome frequencies, and with reduced walking capacity especially at the 1.50Hz metronome frequency.
Helminger et al, 2025	In people with MS, walking distance in the 2-minute walk test showed a statistically significant improvement only in those classified as responders (>5% improvement), while no statistically significant improvement was shown in the timed 25-foot walk test. Responders showed reductions in brain activation within the premotor cortex, precuneus, and middle frontal gyrus, areas linked to motor planning, spatial guidance, and attention. Across people with MS, greater decreases in brain activation in these regions showed a statistically significant correlation with greater improvements in the 2-minute walk test. There was no observed statistically significant association between walking function and motor imagery-related brain activation changes.
Impellizzeri et al, 2020	The music therapy group showed statistically significant improvements in cognitive function (p < .001), long-term retrieval (p= 0.007), delayed recall (p=0.001), and on multiple subscales of motivation, emotional awareness, depression, and quality of life. The between-group effect versus conventional cognitive rehabilitation did not show any statistically

For peer review only

	significant differences (either better or worse). It should be noted that conventional cognitive rehabilitation was administered to both arms.
Kuhlmann et al, 2025	Compared to the silent condition, monochord music therapy was associated with a statistically significant reduction in psychosocial fatigue (p=0.029), increased heat pain thresholds (p=0.024) and stronger immediate subjective effects (e.g. feeling balanced: p < 0.001, relaxed: p < 0.001, less pain: p < 0.001). However, there was no statistically significant between-group difference in anxiety levels (p=0.109).
Maggio et al, 2021	Participants receiving music-assisted treadmill training demonstrated significant improvements in balance (BBS, p<0.002), walking speed (10mWT, p<0.002), mobility (TUG, p<0.002), mood (BDI-II, p<0.002), physical QoL (MSQOL-PH, p<0.002), mental QoL (MSQOL-MH, p<0.001), and goal attainment (GAS, p<0.002). Compared with conventional gait training, significantly greater improvements were observed in walking speed (p=0.004), mobility (p=0.001–0.003), physical QoL (p=0.004), mental QoL (p<0.001), and goal attainment (p=0.002).
Moore et al, 2008	There were no statistically significant differences between the spoken word and sung groups for recognition accuracy, false alarms, sensitivity or response bias.
Moumdjian et al, 2019	Participants were able to synchronize to the different tempi. People without cognitive impairment showed statistically significantly higher synchronization with the metronome compared with music (p < 0.001). People with MS perceived statistically significantly less physical (p < 0.0001) and cognitive fatigue (p < 0.0001) when walking to music. People with cognitive impairment perceived lower fatigue walking to music compared with metronomes (p < 0.0001).
Naderi et al, 2022	Rhythmic auditory stimulation statistically significantly improved gait symmetry and spatiotemporal parameters in MS patients. At 90% tempo, stride length, stride width, and double support duration increased, while cadence decreased. At 110% tempo, cadence increased while stride length, stride width, and double support duration decreased, indicating improved efficiency and stability. Across all tempi (90%, 100%, 110%), gait symmetry indices improved compared to walking without rhythmic auditory stimulation.
Schmid et al, 2004	No significant between-group differences were observed in functional status (MSFC), self-acceptance, depression, anxiety, or quality of life at the end of treatment. However, medium effect sizes favoured music therapy for self-esteem (d=0.54), depression (d=0.63), and anxiety (d=0.63). Within the music therapy group, significant improvements were observed in self-acceptance (p=0.012), depression (p=0.035–0.036), and anxiety (p=0.013), while no significant changes were found in functional status or quality of life.
Seebacher et al, 2024	Music-cued motor imagery, combined motor imagery and gait training, and conventional gait training (CGT) all improved walking performance, fatigue, and several domains of health-related quality of life. However, no significant between-group differences or interaction effects were observed for quality of life outcomes. Reductions in physical, cognitive, and total fatigue were reported across all groups, with a significant between-group difference favouring CGT for cognitive fatigue (p=0.048). No significant effects were reported for anxiety, depression, or cognitive function.

For peer review only

Seebacher et al, 2019	All music interventions statistically significantly improved walking speed and distance. Motor outcomes, fatigue and quality of life significantly improved only after music and verbally cued motor imagery. Synchronisation improved more with music and verbal motor imagery and music motor imagery and worsened with just motor imagery.
Seebacher et al, 2018	Feasibility was demonstrated. There were no safety-related or adverse events related to the study. Improvements in all groups for walking speed and distance, with greatest improvement in the music and verbally cued motor imagery group. Improvements in fatigue, quality of life and motor imagery ability were observed. Only one participant (in the music and verbally cued motor imagery group) showed a clinically significant improvement (defined as at least 20%) in the timed 25-foot walk test. 60% of participants in the music and verbally cued motor imagery group and 40% of participants in other groups showed a clinically significant improvement (defined as at least 20%) in the 6-minute walk test. Fatigue decreased and QoL improved following music-cued and non-cued motor imagery interventions, although findings were exploratory as this is a feasibility study.
Seebacher et al, 2017	Music-cued and metronome-cued motor imagery significantly improved walking speed and walking distance compared with controls (both $p<0.0001$). Cognitive and total fatigue improved in both intervention groups, while physical fatigue, vitality, and several QoL domains improved only after music-cued motor imagery.
Seebacher et al, 2015	Motor imagery with instrumental music and metronome cues statistically significantly improved walking speed and distance in intervention groups compared to no music.
Seighalani et al, 2014	Compared to no intervention, music therapy significantly reduced depression and anxiety from pretest to post-test and follow-up, with effects persisting over time. Depression scores decreased markedly (39.13 to ~17), and anxiety scores halved (41.07 to ~21). Self-esteem scores improved substantially, rising from 16.00 at pretest to ~37–38 at post-test and follow-up. While differences between post-test and follow-up were minimal, all outcomes showed significant improvements compared to pretest.
Shahraki et al, 2017	Statistically significant improvements in the gait parameters stride length, time, double support time, cadence, and gait speed were found in the rhythmic auditory stimulation group compared to the control group doing the same exercises without rhythmic auditory stimulation.
Thaut et al, 2008	Music-based learning was associated with superior word order recall compared with spoken learning,($F(1,18) = 4.51$, $p = 0.038$). Musical learning led to greater improvements in early and late learning phases, while spoken learning showed only transient gains that reduced toward the end of the task.
Thaut et al, 2014	The sung word list group showed statistically significantly more improvement in word order memory and recall ($p= 0.038$) and bilateral frontal alpha learning related synchronization ($p= 0.05$) than the spoken word group.
Wiens et al, 1999	Compared to passive music engagement, active music therapy was not shown to be associated with statistically significant improvements in the strength of respiratory muscles in people with advanced MS.
Young et al, 2019	Statistically significant improvements for music to movement versus no active music therapy or yoga were shown for in Timed Up and Go (least square mean difference [95% confidence interval] = −1.9s [−3.3 to −0.5], P=.01, d=0.7 and 6-minute walk test (41.0m [2.2-80.0], P=.04, d=0.6; controlled for Patient-Determined Disease Steps). No significant group

	differences were found in 5-times sit-to-stand, fatigue, and pain interference. Tests of music to movement versus control were post-hoc as the study also contained an active yoga intervention and the primary comparisons were made across music to movement, active yoga and control.

For peer review only

Supplementary file 10. Risk of bias results

Risk of bias and methodological quality were assessed using the Critical Appraisal Skills Programme (CASP) checklists, selected according to study design (randomised controlled trials, cohort studies, case control studies, and cross-sectional studies). CASP was chosen to provide a structured, domain-based assessment of internal validity, applicability, and reporting quality, rather than a single summary score. Interpretation focused on domain level patterns of bias, consistent with recommendations to avoid reliance on composite quality scores.
Across the 23 included studies, the RCT checklist was the most frequently applied, reflecting that most studies were experimental in design. Where previous reviews had inconsistently labelled some studies as non-randomised, quasi experimental, or clinical trials, the present review applied CASP checklists based on the actual methodological features reported, rather than labels used in earlier syntheses. This resulted in three intentional mismatches between study labels used in prior reviews and the CASP checklist applied here. For example, Maggio et al (2021) and Naderi et al (2022) were assessed using the RCT checklist despite being described as quasi experimental in some summaries, as both involved prospective intervention allocation and comparator groups. Conversely, Aldridge et al (2005) and Schmid et al (2004) were assessed using the cohort checklist, as allocation was matched rather than randomised. These decisions were made to ensure the most appropriate appraisal tool was applied to each study's actual design.

Overall, most studies addressed a clearly focused research question, reported outcomes comprehensively, and used validated outcome measures. Baseline characteristics were generally comparable between intervention and control groups where applicable. However, several recurring sources of bias were identified across studies. Blinding of participants and intervention providers was rarely possible, reflecting the nature of music-based interventions. While some studies reported assessor blinding, this was inconsistently applied or insufficiently described. Allocation concealment was rarely reported and may represent either a reporting limitation or a true methodological weakness.

Sample size was a common concern. Many studies were small, exploratory, or pilot in nature, limiting statistical power and increasing the risk of type II error. Participant flow and attrition were not always clearly reported, particularly in older studies and non-randomised designs, raising potential concerns regarding selection and attrition bias. Although most studies reported statistically significant findings where present, clinical significance was rarely addressed, and confidence intervals or precision estimates were inconsistently reported.

Observational and non-randomised studies generally demonstrated appropriate measurement of exposure and outcomes, but often relied on convenience sampling, increasing the risk of selection bias. Cross sectional and case control designs were limited in their ability to account for confounding, although the case control study assessed using CASP demonstrated strong internal consistency across domains. Cohort studies scored highly overall but lacked detailed reporting of precision and effect estimates.

Several important limitations were identified that are not fully captured by CASP or other standard risk of bias tools. These include heterogeneity in intervention content, duration, and delivery personnel; variability in outcome measures across studies; frequent underrepresentation of male participants; and reliance on self-reported outcomes in several domains. In addition, some single session experimental studies lacked a comparator capable of controlling for non-specific effects such as attention, novelty, or participant expectation.

For peer review only

Despite these limitations, higher quality RCTs demonstrated generally good internal validity, clear outcome reporting, and appropriate analytical approaches. Studies rated as high quality were more likely to include blinded outcome assessment, clearer reporting of statistical methods, and better alignment between results and conclusions. Across study designs, conflicts of interest were not identified as a major concern, and authors typically acknowledged at least some methodological limitations.

Overall, the risk of bias profile suggests that while many studies were thoughtfully designed and conducted within the practical constraints of arts-based research, methodological limitations were common, particularly relating to blinding, sample size, and reporting transparency. These factors should be considered when interpreting the strength and consistency of the evidence base.

CASP risk of bias checklists

Part A: RCT checklist

Study	Clear research question?	Randomised?	All participants accounted for?	Participants blinded?	Investigators blinded?	Assessors blinded?	Similar baselines?
Conklyn et al, 2010	Yes	Yes	Yes	No	No	No	Yes
Gatti et al, 2015	Yes	Yes	Yes	No	No	No	Yes
Helminger et al. 2025	Yes	Yes	Yes	No	No	No	Yes
Impellizzeri et al, 2020	Yes	Yes	Yes	Yes	No	No	Yes
Kuhlmann et al, 2025	Yes	Yes	No	No	Yes	No	Yes
Maggio et al. 2021	Yes	No	Yes	No	No	No	Yes
Moore KS. et al. 2008	Yes	Yes	Yes	No	No	No	Yes
Naderi S. Et al. 2022	Yes	No	Yes	No	No	No	Yes
Seebacher et al, 2015	Yes	Yes	Yes	No	No	No	Yes
Seebacher et al, 2017	Yes	Yes	Yes	No	No	No	Yes
Seebacher et al, 2018	Yes	Yes	Yes	No	No	No	Yes
Seebacher et al, 2019	Yes	Yes	Yes	No	No	No	Yes

For peer review only

Seebacher et al, 2024	Yes	Yes	Yes	Yes	Yes	No	Yes
Seighalani et al, 2014	Yes	Yes	Yes	No	No	No	Yes
Shakraki et al, 2017	Yes	Yes	Yes	No	No	No	Yes
Thaut et al, 2008	Yes	yes	Yes	No	No	No	Yes
Thaut et al, 2014	Yes	Yes	Yes	No	Yes	No	Yes
Wiens et al, 1999	Yes	Yes	Yes	No	No	No	Yes
Young et al, 2019	Yes	Yes	Yes	No	No	Yes	Yes

Study	Same level of care?*	Effects of intervention reported comprehensively?	Precision of treatment effect reported?	Benefits outweigh harms and costs?	Applicable to our context?	Greater value than existing treatment?
Conklyn et al, 2010	Yes	Yes	No	Yes	Yes	Can't tell
Gatti et al, 2015	Yes	Yes	Yes	Yes	Yes	Can't tell
Helminger et al. 2025	Yes	Yes	Yes	Yes	Yes	Can't tell
Impellizzeri et al, 2020	Yes	Yes	Yes	Yes	Yes	Can't tell
Kuhlmann et al, 2025	Yes	Yes	Yes	Yes	Yes	Can't tell
Maggio et al. 2021	Yes	Yes	No	Yes	Yes	Can't tell
Moore. et al. 2008	Yes	Yes	No	Yes	Yes	Can't tell
Naderi. et al. 2022	Yes	Yes	No	Yes	Yes	Can't tell
Seebacher et al, 2015	Yes	Yes	Yes	Yes	Yes	Can't tell
Seebacher et al, 2017	Yes	Yes	Yes	Yes	Yes	Can't tell

For peer review only

Seebacher et al, 2018	Yes	Yes	Yes	Yes	Yes	Can't tell
Seebacher et al, 2019	Yes	Yes	Yes	Yes	Yes	Can't tell
Seebacher et al, 2024	Yes	Yes	Yes	Yes	Yes	Can't tell
Seighalani et al, 2014	Yes	Yes	No	Yes	Yes	Can't tell
Shakraki et al, 2017	Yes	Yes	No	Yes	Yes	Can't tell
Thaut et al, 2008	Yes	Yes	No	Yes	Yes	Can't tell
Thaut et al, 2014	Yes	Yes	No	Yes	Yes	Can't tell
Wiens et al, 1999	Yes	Yes	No	Yes	Yes	Can't tell
Young et al, 2019	Yes	Yes	Yes	Yes	Yes	Can't tell

* = apart from intervention

Part B: Cross-sectional checklist

**Study**	**Clear research question?**	**Appropriate method?**	**Acceptable recruitment?**	**Accurate measurement to reduce bias?**	**Data collected in a way that addressed research issue?**	**Enough participants?**	**Clear results presentation?**
Goetschalckx et al, 2021	Yes	Yes	Yes	Yes	Yes	Can't tell	Yes

**Study**	**Rigorous analysis?**	**Clear statement of findings?**	**Applicable to local population?**	**How valuable is the research?**
Goetschalckx et al, 2021	Yes	Yes	Yes	Can't tell

Part C: Case control checklist

For peer review only

Study	Clearly focused issue?	Appropriate method?	Acceptable recruitment?	Acceptable control selection?	Exposure accurately measured to minimise bias?	Groups similar apart from exposure?	Confounding taken into account in design and analysis?
Moumdjian et al, 2019	Yes	Yes	Yes	Yes	Yes	Yes	Yes

Study	Large treatment effect?	Precise estimate of treatment effect?	Do you believe the results?	Applicable to our context?	Results fit with other available evidence?
Moumdjian et al, 2019	Can't tell	Can't tell	Yes	Yes	Yes

Part D: Cohort checklist

Study	Clearly focused issue?	Acceptable recruitment?	Exposure accurately measured to minimise bias?	Outcome accurately measured to minimise bias?	All important confounders identified?	Confounding taken into account in design and analysis?	Follow-up complete enough?
Aldridge et al. 2005	Yes	Yes	Yes	Yes	Yes	Yes	Yes
Schmid et al, 2004	Yes	Yes	Yes	Yes	Yes	Yes	Yes

Study	Clear results presentation?	How precise are the results?	Do you believe the results?	Applicable to local population?	Results fit with other available evidence?	Clear implications for practice?
Aldridge et al. 2005	Yes	Can't tell	Yes	Yes	Yes	Can't tell
Schmid et al, 2004	Can't tell	No	Yes	Yes	Yes	Can't tell

For peer review only

Supplemental file 11: Assessment of reporting bias

**Study**	**Protocol available?**	**Protocol outcomes not reported in journal**
Aldridge et al (2005)	No	Not evaluable
Conklyn et al (2010)	No	Not evaluable
Gatti et al (2015)	No	Not evaluable
Goetschalckx et al (2021)	NCT04142853 and NCT03938558	Primary outcomes from the protocol not reported in journal: Stroop test, Nine-hole peg test, Fatigability index hand, motor fatigability, trait motor fatigue*
Helminger et al (2025)	DRKS00023978 (German trials registry) **	All primary outcomes from the protocol were reported in the journal – some secondary clinical and psychological outcomes were not
Impellizzeri et al (2020)	No	Not evaluable
Kuhlmann et al (2025)	DRKS00024549	All protocol outcomes reported in journal
Maggio et al (2021)	No	Not evaluable
Moore et al (2008)	No	Not evaluable
Moumdjian et al (2019)	NCT03281330	All protocol outcomes reported in journal
Naderi et al (2022)	No	Not evaluable
Schmid et al (2004)	No	Not evaluable
Seebacher et al (2015)	ISRCTN67054113	Primary outcomes from protocol (T25FW + 6MWT) switched to secondary outcomes in the journal, one of the secondary outcomes from the protocol (MFIS) switched to primary outcome in the journal*
Seebacher et al (2017)	ISRCTN67054113	All protocol outcomes reported in journal
Seebacher et al (2018)	ISRCTN92351899***	Some of the primary outcomes from the protocol (T25FW and 6MWT) switched to secondary outcomes in the journal
Seebacher et al (2019)	ISRCTN92351899***	All protocol outcomes reported in journal
Seebacher et al (2024)	DRKS00023978**	All protocol outcomes reported in journal
Seighalani et al (2014)	No	Not evaluable
Shahraki et al (2017)	No	Not evaluable
Thaut et al (2008)	No	Not evaluable
Thaut et al (2014)	No	Not evaluable
Wiens et al (1999)	No	Not evaluable
Young et al (2019)	NCT02533882	Primary outcomes from the protocol not reported in journal: VO2 fitness, pain, fatigue, loneliness, grip strength, balance tests*

For peer review only

All protocols were published prospectively rather than retrospectively, * there were also secondary outcomes from the protocol not reported in the journal (however where primary outcomes from the protocol are not reported in the journal we focus on listing these in the table), ** while these studies share a protocol registration number, they are separate studies with different aims and outcomes (total sample size across the two studies = 147, the sample sizes for the two separate studies are 121 and 31 considering only included participants, suggesting an overlap of 5 participants - 3% of the total sample size across the two studies), *** while these two studies share a protocol registration number, they are separate studies and have no overlap in participants (total sample size across the two studies = 75, the sample sizes for the two separate studies are 15 and 60).

For peer review only

Supplemental file 12. Effect direction plot

Study	Study Design	Motor outcomes	Cognitive outcomes	Psychological outcomes	Quality of life outcomes
Aldridge D. et al. 2005	Matched controlled pilot study		◄►	▲	◄►
Conklyn D. et al. 2010	RCT	▲			◄►
Gatti R. Et al. 2015	RCT	▲			
Goetschalckx M. et al. 2021	Observational cross-sectional comparative study	◄►			
Helminger B. et al. 2025	RCT	◄►			
Impellizzeri et al, 2020	RCT		▲	▲	▲
Kuhlmann J et al. 2025	RCT			▲	▲
Maggio et al. 2021	Quasi-experimental controlled feasibility study	▲	▲	▲	▲
Moore KS. et al. 2008	RCT		◄►		
Moumdjian L. et al. 2019	Observational case control study	▲			▲
Naderi S. Et al. 2022	Quasi-experimental controlled study with repeated measures	▲			
Schmid et al. 2014	Matched control study	◄►	◄►	▲	◄►
Seebacher B. et al. 2015	RCT	▲			▲
Seebacher B. et al. 2017	RCT	▲			▲
Seebacher B. et al. 2018	RCT	▲			▲

For peer review only

Seebacher B. et al. 2019	RCT	▲			▲
Seebacher B. et al. 2024	RCT	▲	◄►	◄►	▲
Seighalani M. et al. 2014	RCT			▲	
Shahraki M et al, 2017	RCT	▲			
Thaut et al. 2008	RCT		▲		
Thaut M. et al. 2014	RCT		▲		
Wiens M E et al. 1999	RCT	◄►			
Young HJ. Et al. 2019	RCT	▲			◄►

Legend:
Study design: RCT: Randomised Controlled Trial. Effect direction: upward arrow ▲= positive health impact, downward arrow ▼= negative health impact, sideways arrow ◄►= no change/mixed effects/conflicting findings. Sample size: Final sample size (individuals) in intervention group Large arrow ▲ >300; medium arrow ▲ 50-300; small arrow ▲ <50. Following Boon and Thomson[82]

For peer review only

Supplemental file 13. Evaluation of minimally clinically important differences

Outcome domain	Measure	MCID	Findings
Motor	Timed 25-foot walk test	17.2% improvement[76]	• Helminger et al[49] – no clinically significant difference in participants receiving actual and/or imagined gait training with rhythmic-auditory cueing from baseline to follow-up. **Within-group**<br>• Seebacher et al[55] – clinically significant difference in participants receiving music cued motor imagery (17.3%) and metronome cued motor imagery (20.0%), but not controls, from baseline to follow-up. **Within-group**<br>• Seebacher et al[56] – no clinically significant difference in any group from baseline to follow-up. **Within-group**<br>• Seebacher et al[57] – no group-level clinically significant difference in any group from baseline to follow-up. One out of five participants in the music- and verbally cued motor imagery group had a clinically significant difference and no participants in other groups. **Within-group**<br>• Seebacher et al [58] – no group-level clinically significant difference in any group from baseline to follow-up. 21.1% of participants in the in the music- and verbally cued motor imagery group, 15.0% in the music-cued motor imagery and none in the motor imagery group had a clinically significant difference (defined as >=20% by the authors of this study). **Within-group**<br>• Seebacher et al[59] – no clinically significant difference in any group from baseline to the final follow-up (week 13). **Within group**<br><br>One study out of six found evidence of a clinically significant difference. All assessed within-group rather than between-group differences, although for some studies information was available for more than one group.

For peer review only

Psychological	Beck depression	17.5% reduction[77]	• Impellizeri et al[32] – clinically significant difference in experimental group (46.4%) but not the control group from baseline to follow-up.<br>**Within-group**<br>• Maggio et al[51] – clinically significant difference in experimental group (56.2%) but not the control group from baseline to follow-up.<br>**Within-group**<br>• Schmid et al[30] - numerical descriptive results not presented, therefore not evaluable<br>Two studies out of two (that were evaluable) found evidence of a clinically significant difference. Both assessed within-group rather than between-group differences, although information was available for more than one group.
Psychological	HADS anxiety	0.80 points (for subscale)[79]	• Kuhlmann et al[50] – no clinically significant difference from baseline to follow-up for the experimental group, although a clinically significant improvement (1.5 points) was found for the control group.<br>**Within-group**<br>• Schmid et al[30] – numerical descriptive results not presented, therefore not evaluable<br>• Seebacher et al[59] – secondary outcome but data not presented, therefore not evaluable<br>No studies out of one (that were evaluable) found evidence of a clinically significant difference. This study assessed within-group rather than between-group differences, although information was available for more than one group.
QoL	MS Impact Scale-29 (MSIS-29)	8 points for physical subscale[81]	• Seebacher et al[58] – clinically significant difference in the music- and verbally-cued motor imagery group (15 points), but not the music-cued motor imagery or motor imagery groups. 79% of participants in the music- and verbally cued motor imagery group, 50% of participants in the music-cued motor imagery group, and 35% of participants in the motor imagery group had a clinically

			significant difference, defined as 7.5 points by the authors of this study.<br>**Within-group**<br>• Seebacher et al[57] – no clinically significant difference in any group from baseline to follow-up (although the music-cued motor imagery group met the more lenient threshold of 7.5 points).<br>**Within-group**<br>• Seebacher et al[56] – no clinically significant difference in any group from baseline to follow-up. 50% of participants in the music- and verbally cued motor imagery group, 41.2% in the music-cued motor imagery group, and 21.2% of those in the motor imagery group had a clinically significant difference, defined as 7.5 points by the authors of this study.<br>**Within-group**<br><br>One study out of three found evidence of a clinically significant difference. All assessed within-group rather than between-group differences, although information was available for more than one group.

Cognitive MCIDs could not be evaluated, as explained in Table 2.

For peer review only

BMJ Open

Potential benefits of music-based therapies on rehabilitation outcomes in people with multiple sclerosis: a systematic review

Journal:	*BMJ Open*
Manuscript ID	bmjopen-2026-116420.R2
Article Type:	Original research
Date Submitted by the Author:	05-Jul-2026
Complete List of Authors:	Ahadi, Nicky ; University of Exeter, University of Exeter Medical School<br>Kim, Jeeyeon ; University of Michigan, Department of Physical Medicine and Rehabilitation<br>Whibley, Daniel; University of Michigan, Department of Physical Medicine and Rehabilitation; University of Michigan, Center for History, Humanities, Arts, Social Sciences, and Ethics in Medicine<br>Barnish, Maxwell; University of Exeter, Peninsula Technology Assessment Group (PenTAG), School of Public Health and Sport Sciences, University of Exeter Medical School
**Primary Subject Heading**:	Public health
Secondary Subject Heading:	Neurology
Keywords:	Multiple sclerosis < NEUROLOGY, COMPLEMENTARY MEDICINE, PUBLIC HEALTH

SCHOLARONE
MANUSCRIPTS

I, the Submitting Author has the right to grant and does grant on behalf of all authors of the Work (as defined in the below author licence), an exclusive licence and/or a non-exclusive licence for contributions from authors who are: i) UK Crown employees; ii) where BMJ has agreed a CC-BY licence shall apply, and/or iii) in accordance with the terms applicable for US Federal Government officers or employees acting as part of their official duties; on a worldwide, perpetual, irrevocable, royalty-free basis to BMJ Publishing Group Ltd ("BMJ") its licensees and where the relevant Journal is co-owned by BMJ to the co-owners of the Journal, to publish the Work in this journal and any other BMJ products and to exploit all rights, as set out in our licence.

The Submitting Author accepts and understands that any supply made under these terms is made by BMJ to the Submitting Author unless you are acting as an employee on behalf of your employer or a postgraduate student of an affiliated institution which is paying any applicable article publishing charge ("APC") for Open Access articles. Where the Submitting Author wishes to make the Work available on an Open Access basis (and intends to pay the relevant APC), the terms of reuse of such Open Access shall be governed by a Creative Commons licence – details of these licences and which Creative Commons licence will apply to this Work are set out in our licence referred to above.

Other than as permitted in any relevant BMJ Author's Self Archiving Policies, I confirm this Work has not been accepted for publication elsewhere, is not being considered for publication elsewhere and does not duplicate material already published. I confirm all authors consent to publication of this Work and authorise the granting of this licence.

For peer review only

Potential benefits of music-based therapies on rehabilitation outcomes in people with multiple sclerosis: a systematic review

Nicky Ahadi[1], Jeeyeon Kim[2], Daniel Whibley[2,3], Maxwell S. Barnish[4]

1 University of Exeter Medical School, University of Exeter, Exeter, UK

2 Department of Physical Medicine and Rehabilitation, University of Michigan, Ann Arbor, USA

3 Center for History, Humanities, Arts, Social Sciences, and Ethics in Medicine, University of Michigan, Ann Arbor, USA

4 Peninsula Technology Assessment Group (PenTAG), School of Public Health and Sport Sciences, University of Exeter Medical School, University of Exeter, Exeter, UK

Correspondence to: Dr Maxwell S. Barnish; m.s.barnish@exeter.ac.uk

Keywords: Multiple sclerosis; Music therapy; Music-based therapies

For peer review only

ABSTRACT

Objectives. To assess the evidence for a benefit of music-based therapies (MBTs) on rehabilitation outcomes in adults with multiple sclerosis (MS).

Setting. Scholarly literature (published in English) from any country. This systematic review received no specific funding and was registered on PROSPERO as CRD420251083656.

Data sources. Six bibliographic databases: AMED (Ebsco), APA PsycINFO (Ovid), CINAHL (Ebsco), Cochrane CENTRAL, EMBASE (Ovid) and MEDLINE (Ovid), plus supplementary searches and citation chasing (search date July 2025).

Primary and secondary outcome measures. Eligible studies used a clinical trial or controlled quantitative observational study design to assess the benefit of MBTs on rehabilitation outcomes in adults with MS. Risk of bias was assessed using CASP checklists. Data were synthesised using narrative synthesis.

Results. 23 studies were included (955 participants, of whom 880 people had MS and 691 received MBTs). The most frequently assessed outcome was motor function (16 studies), followed by quality of life (12 studies). Evidence suggests potential benefits for MBTs, particularly rhythmic auditory stimulation and music-cued motor imagery, on gait and mobility outcomes, including walking speed, walking distance, cadence, stride length, and gait symmetry. Findings for other outcomes were less clear.

Conclusions. MBTs may benefit people with MS, with the most consistent evidence observed for gait and mobility outcomes. However, heterogeneity in intervention type, study design, and outcome measurement limits the strength of conclusions. Higher-quality, adequately powered trials with standardised outcomes and direct comparisons between MBTs are needed to clarify effectiveness and inform clinical implementation.

STRENGTHS AND LIMITATIONS OF THIS STUDY

- Systematic review methods minimised subjectivity and bias.
- A structured outcome set was used.
- An independent dual review was conducted.
- Patient and public involvement was not conducted.
- Only English-language studies could be included.

For peer review only

INTRODUCTION

Multiple sclerosis

Multiple sclerosis (MS) is a chronic inflammatory and neurodegenerative disease of the central nervous system and a leading cause of neurological disability in young adults.[1,2] It affects more than 2.8 million people worldwide and its prevalence has increased substantially in many regions, with the highest rates reported in North America, western Europe and Australasia.[1-3] This rise is attributed to a combination of greater life expectancy, population growth, and better access to diagnostic services.[2,3] Common manifestations include optic neuritis, sensory disturbance, limb weakness and gait ataxia as any site in the central nervous system can be affected.[1-3] MS is usually described as having three main types with Relapsing Remitting MS (RRMS) being the most common, affecting around 85-90% of people with MS. Primary progressive MS (PPMS) accounts for about 10-15% and progresses steadily without relapses. Over time, many with RRMS develop secondary progressive MS (SPMS), where disability gradually increases.[1]

Treatment options for MS

Disease modifying therapies can reduce relapse frequency and delay disability progression, yet they do not fully address the multifactorial physical, cognitive, and emotional consequences of living with a chronic, unpredictable condition. MS places a heavy burden on health systems and carries high economic costs.[2,3] As symptoms often persist despite medication, there is increasing interest in non-pharmacological approaches to support holistic rehabilitation and improve quality of life (QoL).[3,4] These approaches include arts-based interventions, which have been shown to be promising therapeutic approaches in anxiety and depression[5], palliative care[6], stroke[7] and Parkinson's disease[8-10]. Within this broad arts sphere, one focal point of therapeutic interest is the use of music for therapeutic benefit.

The concept of Music-Based Therapies

A range of different approaches have been taken to conceptualising music interventions for therapeutic benefit. One established concept is music therapy (MT). The American Music Therapy Association defines MT as "the clinical and evidence-based use of music interventions to accomplish individualized goals within a therapeutic relationship by a credentialed professional who has completed an approved music therapy program".[11] However, definitions of MT vary greatly internationally, making the concept challenging to interpret in the international literature. The UK, USA and Canada take a highly formalised approach to MT using protected, clinically based legal definitions, requiring specific MT qualifications and registration with a statutory body.[11-13] Meanwhile, the German Music Therapy Society focuses on the "targeted use of music" to restore, maintain and promote mental, physical and cognitive health",[14] the Norwegian approach focuses on "community music therapy", combining individual clinical work with social, community-based approaches,[15-16] and the Chinese approach incorporates theories from traditional Chinese medicine (TCM), such as the 'five tones' model, alongside western MT approaches.[17] Areas of between-country variation include whether statutory registration is required, whether the focus is on active or receptive MT approaches, and the extent to which MT is clinically focused and conducted individually or socially focused and conducted in groups. Therefore, MT does not have the same meaning in different countries - interventions that would be described as MT in some countries would not be permitted to be described as MT in other countries.[14] Pragmatic definitions of MT have been used by some researchers[5,8,9] to overcome this lack of a universal definition of MT, however the misalignment of these definitions with those used by statutory bodies may be problematic.

For peer review only

Noting these issues defining MT in a consistent and meaningful way for international research, broader concepts have been proposed that include interventions that may be beneficial but need not be delivered by a certified music therapist. The broadest alternative conceptualisation of the use of music for health benefit is “musical care” (MC) – a concept developed by Spiro and Sanfilippo.[18] MC is defined as “the role of music—music listening as well as music-making—in supporting any aspect of people's developmental or health needs: for example, physical and mental health, cognitive and behavioral development, and interpersonal relationships”.[18] The authors state[18] that MC encompasses MT but goes beyond MT and includes a wide range of musically based interventions. The concept of MC considers the role of music in its broadest sense and does not only consider structured interventions, nor does it only consider interventions that are therapeutic – MC includes unstructured activities that include music and interventions where music is not the primary therapeutic modality.[18] This may be considered a limitation of the MC concept.

Therefore, the concept of music-based therapies (MBTs) has been proposed, as well as the alternative term music-based interventions (MBIs). As these terms are synonymous, we shall use the term MBTs consistently. The concept of MBTs has been used in several previous systematic reviews in neurological conditions.[19,20,21] Lin et al[19] delineate their review in the full text as assessing MBTs, although the term MT is used in the article title. However, a common limitation in previous reviews using the MBTs concept across different health conditions has been a lack of an explicit definition of MBTs. The key distinction between MBTs and music therapy is that while MBTs are designed to be therapeutic and often to enhance general wellbeing or provide short-term symptom relief, they need not be clinical interventions delivered by a certified music therapist.[22] Based on the work of Raglio,[22] we propose the following explicit operational definition of MBTs for the purposes of our work: “structured musical activities designed to enhance well-being, reduce stress, and improve quality of life, without necessarily focusing on clinical diagnosis, assessment, or long-term psychotherapy”. While professional regulation and licensing remain important for clinical governance, our conceptualisation prioritises the active components of MBTs over jurisdiction-specific labels. Ultimately, understanding which components produce therapeutic effects is most important for advancing the evidence base. Our theoretical and conceptual justification for aggregating diverse music-based activities under the concept of MBTs lies in their shared complexity as interventions with potentially multiple “active ingredients” that promote health, well-being, and neurophysiological change.[23] MBTs are mid-range in their scope – broader than MT but narrower than MC, as MBTs do not include activities that are not therapeutic or do not comprise structured interventions. MT falls within the stated definition of MBTs, but not all aspects of MC are included under MBTs.

Evidence for Music-Based Therapies in MS

We have identified six previous systematic,[20, 24-28] scoping or comprehensive reviews on MBTs for MS, in addition to a broad non-systematic literature review on ‘cognitive re-education’ for QoL and psychological wellbeing in MS.[29] Table 1 summarises the scope, methodological approaches, interventions, outcomes, and included studies of these reviews. Due to clinical, methodological and statistical heterogeneity between studies, no reviews have been able to include a meta-analysis. An early comprehensive review[26] assessed MT in relation to a non-specified set of outcomes and found very limited evidence, with the only full-text peer-reviewed papers besides case reports or case series being two pilot studies.[30,31] Systematic and scoping reviews have typically been limited to specific outcome domains: i) gait and mobility[25,28] and ii) psychological outcomes.[24] Gardener et al[24] conducted a systematic review on MT for psychological outcomes in a broader range of conditions and included only one pilot RCT[32] on MS. Tan et al[28] conducted a recent scoping review of 15 studies on music based and rhythmic

auditory interventions for walking in MS. They focused exclusively on gait and mobility outcomes and reported high acceptability and consistent short-term improvements in walking speed and quality. Kong et al, 2023[25] similarly undertook a systematic review of 12 clinical trials of MT for gait disorders in MS, again concentrating on mobility outcomes and finding that music based rhythmic cueing improved gait performance. Together, these reviews support the role of music-based approaches for gait rehabilitation but do not explore wider motor, cognitive, or psychosocial domains. Lopes et al, 2021[20] provided the first MS specific systematic review of MBTs as a rehabilitative approach. Across 10 clinical trials, they grouped interventions into rhythmic auditory strategies, instrument playing, dance-based strategies and neurological MT. They found that MBTs were superior to conventional therapy or no intervention for several motor and non-motor outcomes, including gait, fatigue, balance, hand dexterity, emotional status and pain, although evidence for memory and mental fatigability was less clear. The search period ended in 2020 and interventions and outcome measures were highly heterogeneous, with several protocols primarily dance-based rather than primarily music focused.[20] Phillips et al, 2025[27] extended the scope of the literature by comparing active and receptive music interventions across 15 studies in people with MS, with outcomes including anxiety, depression, QoL, cognition, gait, balance, fatigue, and hand dexterity. Most interventions were receptive, often involving music listening during walking. While the review showed encouraging effects across motor and non-motor domains, its focus on engagement type and the limited number of studies mean it does not fully represent the breadth of MBTs for MS rehabilitation.[27]

Rationale

Existing systematic reviews are fragmented and limited in scope. Available reviews either focus narrowly on gait and walking-related outcomes,[25,28] only include a small number of heterogeneous studies,[20,27] or provide findings from multiple neurological conditions without MS-specific conclusions.[24] Reviews of related performing arts modalities, e.g. dance, highlight potential motor and psychological benefits for people with MS, yet these interventions do not isolate the therapeutic contribution of the musical component.[4] There is no recent systematic review of the potential benefit of MBTs across a broad range of rehabilitation outcomes relevant to people with MS. No existing review provides a comprehensive synthesis of the impact of MBTs on motor, cognitive, psychological or QoL domains, nor evaluates the diversity of interventions used.

Considering specifically reviews published in 2025, here is a clear statement of how they differ from our systematic review and the advance to knowledge our work offers:

- Gardener et al, 2025[24] - this systematic review has a specific focus on MT and psychological outcomes; our review considers MBTs and mobility, cognitive function and QoL outcomes, therefore offering broader intervention and outcome measure coverage.
- Phillips et al, 2025[27] – this systematic review considers ‘music interventions’, mode of engagement (active versus receptive interventions), and ‘balance’, ‘gait’, ‘anxiety’, ‘depression’, and ‘fatigue’. Our review explicitly uses the MBTs framework to define eligibility, includes mobility and psychological outcomes, and includes comparison of MBTs and control arms.
- Tan et al, 2025[28] – this is a scoping review, while our review is a systematic review. Systematic reviews are considered a higher level of evidence compared to scoping reviews and offer a structured assessment of risk of bias.[33] This review considers ‘music interventions’ but does not use an explicitly defined framework such as MBTs, while our review explicitly uses the MBTs framework to define eligibility. This review

For peer review only

limits its outcomes to gait and mobility, while our review also considers cognitive function, psychological well-being, and QoL.

Therefore, our review provides a clear advance in knowledge in terms of coverage of interventions and outcomes. We provide the first systematic review to provide an assessment of the potential benefit of music in MS using the MBTs framework to ensure appropriately broad intervention coverage and assessment of mobility, cognitive function, psychological outcomes, and QoL outcomes.

Aims

The aim of our work was to provide a systematic review of the evidence for a benefit of MBTs on rehabilitation outcomes in adults with multiple sclerosis. In particular, the primary review question was:

- What is the impact of MBTs on rehabilitation outcomes in people with MS?

Secondary review questions were:

- What types of MBTs have been studied for their rehabilitation potential for people with MS?
- What specific rehabilitation domains (for example motor, cognitive, psychological, and QoL) show the greatest response to MBTs in people with MS?

METHODS

Design

A systematic review was conducted following the Preferred Reporting Items for Systematic Reviews and Meta-Analyses (PRISMA) 2020 guidelines.[34] Completed PRISMA and PRISMA for Abstracts checklists are provided (Supplemental files 1 and 2). Reporting of the narrative synthesis was compliant with the Synthesis without meta-analysis (SWiM) guidelines.[35] This systematic review was registered on the PROSPERO database (CRD420251083656). One protocol amendment was made – to use risk of bias tools from the same suite for all study designs.

Data sources

AMED (Ebsco), APA PsycINFO (Ovid), CINAHL (Ebsco), Cochrane-CENTRAL, EMBASE (Ovid) and MEDLINE (Ovid) were searched from inception until July 2025. Supplementary searches were conducted on Google Scholar and through forward and backward citation chasing on studies identified for full-text review up to November 2025. Searches were designed to retrieve articles on MS and MBTs (strategies for all databases are shown in online supplemental file 3) and were designed by NA and MSB and conducted by the lead author NA. MSB conducted supplementary searches and citation chasing.

Inclusion criteria

Screening was initially conducted based on the title and abstract. Potentially relevant articles were screened at the full-text stage to determine inclusion (online supplemental file 4) or exclusion (online supplemental file 5) in the systematic review. Screening was initially conducted by NA based on titles and abstracts, followed by full text screening of potentially eligible studies. A second reviewer (MSB) independently screened all records to verify consistency, and any disagreements were resolved through discussion.

For peer review only

Eligibility criteria are shown in Box 1. The outcomes of interest were rehabilitation-related outcomes: functional recovery, motor function, gait and balance, cognitive function, psychological well-being, and QoL. Studies with and without control arms were eligible. MBTs were defined using the operational definition proposed in the Introduction: “structured musical activities designed to enhance well-being, reduce stress, and improve quality of life, without necessarily focusing on clinical diagnosis, assessment, or long-term psychotherapy”. As MT falls within this definition of MBTs, studies on MT were eligible for inclusion. There were no specific requirements for what control arms should involve. Therefore, control arms could include alternative populations, such as healthy controls or people with another medical condition rather than MS, in addition to alternative interventions. While no limits were set on the types of control arms that were eligible, based on previous systematic reviews on the performing arts in neurological settings[8-9] and knowledge of the field, it was anticipated that control arms may include exercise, physiotherapy, usual care (or no intervention), conventional cognitive rehabilitation, and other MBTs. Screening was conducted using Rayyan, which uses artificial intelligence, machine learning, and natural language processing to make systematic reviewing more efficient by learning from user decisions to predict article relevance and highlight key PICO terms. All screening was done manually by the two human reviewers.

Data extraction

Information extracted is shown in Box 2. All data extraction processes were conducted by NA, and MSB independently reviewed all records to verify consistency. Any disagreements were resolved through discussion. No automation tools were used. The appendix provides additional information on study characteristics (online supplemental file 6), interventions (online supplemental file 7), controls (online supplemental file 8) and narrative results (online supplemental file 9).

Risk of bias assessment

Risk of bias and methodological quality were assessed using the relevant Critical Appraisal Skills Programme (CASP) checklists for randomised controlled trials, cohort studies, case control studies and cross-sectional studies[36]. The descriptive, qualitative approach to CASP was used, whereby we tabulated and described the patterns of bias both within and between studies but did not use a numerical scoring system or assign overall ‘high’, ‘moderate’ and ‘low’ quality labels to studies. Avoidance of the use of numerical composite scores for risk of bias is in line with recommendations from the Agency for Healthcare Research and Quality (AHRQ)[37] in the USA. Quality appraisal was conducted independently by NA, with MSB reviewing all studies to ensure consistency. Disagreements were resolved through discussion. No automation tools were used. The appendix provides additional information on risk of bias checklists used (supplemental file 10).

Narrative synthesis

A thematic narrative synthesis was used to analyse all studies that met the inclusion criteria. Guidance recommends[38] that the decision whether to undertake a meta-analysis should be pre-specified based on clear a priori justification and conceptual understanding rather than decided post-hoc based on formal evaluation of identified studies. In all previous systematic reviews in the field, meta-analysis was not considered appropriate due to substantial methodological heterogeneity across eligible studies. These sources of heterogeneity included: i) study design (including RCTs and observational studies), ii) differences in type and delivery of MBTs (including active and receptive MBTs), iii) differences in disciplinary backgrounds of intervention leaders (including music therapists, other health professionals,

For peer review only

and professional performing artists), iv) differences in session length, frequency and duration, and v) differences in outcome measurement (both conceptual differences and differences in terms of what assessment tools and scales were used). In previous systematic reviews in the field, these factors were considered to preclude the forming of meaningful meta-analysis sets comprising sufficient studies assessing the same combination of intervention, control, and outcome. This precedent from previous systematic reviews was the key factor in our a priori decision that conducting meta-analysis was not feasible. Additionally, we conducted a qualitative post-hoc assessment of whether these issues were also present in the evidence base for our systematic review to confirm the appropriateness of our a priori decision. This showed that the same issues with conducting meta-analysis were present in the evidence base we identified as in the previous systematic reviews, supporting our decision to conduct exclusively narrative synthesis.

To conduct narrative synthesis, initially the narrative study results extracted during data extraction were reviewed to identify thematic commonalities and patterns in the data. This process identified four key outcome themes in the data: i) motor function, ii) cognitive function, iii) psychological outcomes and iv) QoL. The narrative synthesis was initially stratified by outcome using these four outcome domains. All studies that addressed a particular outcome domain were included in the synthesis for that outcome. Within each outcome domain, the synthesis was further stratified by the type of MBTs assessed. This could include rhythmic auditory stimulation, receptive music listening, active music making and structured MT interventions. However, the types of MBTs were not imposed on the synthesis a priori to allow these to emerge thematically from the data within each outcome domain. As a third level of stratification, within each type of MBTs for each outcome domain, evidence was stratified by interventional and observational study designs, also noting the evidence specifically available from RCTs. Our approach to narrative synthesis enabled comparison across rehabilitation domains and facilitated evaluation of how different MBTs contribute to rehabilitation outcomes in people with MS. For all outcomes, the primary standardised metric for narrative synthesis – based on the SWiM guidelines –[35] was direction of findings to provide an effective overall synthesis of the patterns of findings, while noting considerable differences in how outcomes were measured and analysed across studies. The potential of small-study bias was assessed using an effect direction plot. The potential of outcome reporting bias was assessed by comparing outcomes in published protocols or clinical trials registries with the outcomes in the journal publications. Heterogeneity could not be assessed quantitatively due to the differences in outcome assessment that precluded meta-analysis. However, differences in study designs, study characteristics, interventions and comparators were profiled in Supplemental files 6-8 and reflected upon at appropriate points in the results and discussion. The textual write-up of the narrative synthesis was supplemented by a detailed results table (Supplemental file 9). The table was ordered alphabetically, while the textual write-up was structured according to the stratification factors stated above (outcome, type of MBTs, and study design). The synthesis was initially conducted by one author (NA) and then reviewed, checked and revised by a second author (MSB).

Clinical significance

The approach to assessing clinical significance was pre-specified. Searches were conducted to identify minimally clinically significant differences (MCIDs)[39] for each of the key thematic areas in the narrative synthesis: i) motor function, ii) cognitive function, iii) psychological outcomes and iv) QoL. There was heterogeneity in the outcome measures used to assess these outcomes across studies. In assessing MCIDs,[40] it is considered best practice to evaluate only outcomes used in multiple studies (typically three or more studies) to ensure validity, stability, and consensus. Therefore, we conducted MCID assessment on outcomes

used on at least three studies included in our review. Furthermore, only outcomes that are on measurement scales amenable to MCID assessment and for which a published MCID can be identified could be included.[41] The distinction between within-group and between-group differences was considered in the reporting and in interpretation of MCID results. The MCIDs identified and applied are shown in Table 2.

Certainty assessment

We conducted certainty assessment using GRADE for each outcome domain supported by the narrative synthesis. As GRADE methods are currently being updated, we used the updated GRADE Book[42] for chapters that had been updated and the 2013 GRADE Handbook[43] for the remaining chapters, as recommended in the GRADE BOOK.[42] We refer to both books below as 'the GRADE guidance'. We describe the approach concisely below. In the GRADE guidance, evidence from RCTs initially start as high certainty evidence, while evidence from non-randomised and observational studies start as low certainty evidence. For RCTs, the risk of systematic or random errors may reduce confidence in the findings. Four GRADE domains assess the risk of systematic errors: 1) study limitations (risk of bias), 2) inconsistency, 3) indirectness, and 4) dissemination bias (publication bias). As noted in the limitations section of the discussion, it was not possible to formally assess publication bias in this systematic review. One GRADE domain evaluates the risk of random error: 1) imprecision. An RCT is downgraded one level from high certainty for every domain for which there are serious concerns, or by two levels if there are very serious concerns. Non-randomised and observational studies may be upgraded from low certainty evidence where the risk of confounding variables is relatively low (for example due to large effect sizes or dose-response gradients) or where the presence of a confounding variable strengthens the inference of effect. Detailed operationalisation criteria are found in the GRADE guidance. We followed the GRADE guidance that the starting point for an outcome domain shall be set as high if the body of evidence consists primarily of RCTs and low if it does not consist primarily of RCTs. The evidence is profiled in Table 3 along with explanations for the GRADE scoring decisions – the presentation of GRADE results is consistent with Barnish et al,[8] although we include this information in the main manuscript rather than the supplemental material for clarity. It was determined that the different forms of MBTs in the evidence base did not form sufficiently clear and distinct categories to conduct separate GRADE assessments by type of MBTs.

Patient and public involvement

Patient and public involvement could not be conducted for this systematic review assessing a broad range of MBTs due to a lack of funding. The corresponding author will respond to any reputable media enquiries.

RESULTS

Search results

Database searches returned a total of 5906 records (AMED 17, PsycINFO/APA PsycINFO 1155, EMBASE 4029 and MEDLINE 198, CINAHL 107, Cochrane central 400), plus 13 from supplementary searches. A total of 4918 records proceeded to title and abstract screening (including one duplicate). 47 unique records (including one duplicate) were assessed at full-text screening; 23 studies were included in the systematic review (Figure 1).

Study profile

Of the 23 included studies, there were 20 interventional studies (14 RCTs, two feasibility RCTs, two quasi-experimental controlled feasibility studies, one secondary analysis of an

For peer review only

RCT, and one matched controlled pilot study) and three observational studies (one observational cross-sectional comparative study, one case-control study, and one matched control study, details in Supplemental file 6). Six included studies (26%) described their approach as MT or the session leader as a music therapist (details in Supplemental file 7). Among the remaining 17 studies, six used gait training or motor imagery, five used rhythmic auditory stimulation, three used sung word list tasks, one used instrument playing, one used listening to music, and one used music to movement (M2M).[44] Three studies (13%) had no control arm. A further four studies (17%) had no control arm comprising people with MS – two of these had a 'healthy' control arm who received the MBTs intervention and two had a 'healthy' control arm who received no intervention. Sixteen studies (70%) had a control group with MS; in seven studies the control was no intervention, in three studies it was learning spoken word lists, in three studies it was a 'placebo' or sham intervention (playing a keyboard that was switched off, lying on top of a monochord that is not being played, or doing the same exercises without rhythmic auditory stimulation), in two studies it was conventional cognitive rehabilitation, and in one study it was receptive music activities – listening to music, discussing music, and learning about music and composers (details in Supplemental file 8). No studies compared MBTs to movement interventions (e.g., exercise or physiotherapy).

The included studies comprised 955 participants, of whom 880 had MS and 75 did not. Across all studies, 691 participants received MBT and 264 did not receive MBT and were therefore classified as control participants. Of the 75 participants without MS, 58 received MBT and 17 did not receive MBT. Therefore, the MBT and control totals include both participants with MS and participants without MS, depending on the study design. Sex was incompletely reported; where data were available, 666 participants (75.0%) were female and 222 (25.0%) were male. Reported mean ages across studies generally ranged from the mid-40s to early 50s, with overall participant ages spanning approximately 29 to 68 years. Disease duration was inconsistently reported, but where available typically ranged from approximately 7 to 18 years since diagnosis, with similar durations between intervention and control groups within individual studies. Baseline disability levels were most commonly reported using the Expanded Disability Status Scale (EDSS). Across studies reporting EDSS, participants generally had mild to moderate disability, with median or mean scores most frequently ranging between 2.0 and 4.5. No studies directly compared different music-based intervention types with each other. MBTs assessed covered both active and passive forms of MT (instrumental and vocal) as well as other MBTs such as musically and rhythmically based cueing, and rhythmic auditory stimulation. No studies assessed choral singing or community singing groups. Further details, including on the types of MBTs assessed, are provided in Supplemental file 10.

Narrative synthesis

As there are 23 included studies, an overview of the evidence landscape is provided in Table 4. Several factors appeared to influence intervention effectiveness. Positive motor outcomes were most consistently reported in studies using rhythmic auditory cueing, RAS, and music-cued motor imagery. Benefits were generally observed in walking related outcomes; However, effects on fatigue, QoL, and psychological outcomes were more variable. In terms of contextual moderators, variations in intervention type (active vs. passive), cueing method, intervention duration, baseline disability level, and sample size may have contributed to differences in outcomes across studies.

Motor outcomes

For peer review only

Motor outcomes were assessed in 16 studies (70%) – profiled in Supplemental files 4-6. Fourteen studies were interventional – of which 9 were RCTs,[31,44,46,47,52,56,58,59,61] and 5 were other interventional designs (secondary analysis of RCT, quasi-experimental controlled feasibility study, and feasibility RCT)[49,51,54-56] – and two were observational studies.[48,53]

One study[31] (an RCT) looked at active music therapy and showed that numerically people with MS receiving active music therapy improved in terms of the strength of their respiratory muscles, while those in the passive music engagement group showed a numerical decline, although the between-group difference did not reach statistical significance. Active music making (keyboard playing) – not led by a music therapist – was associated with a statistically significant improvement (p=0.003, Cohen's d = 1.66, large effect) in hand function compared to playing with the keyboard switched off in one study[47] (an RCT). However, there was no between-group difference in hand dexterity or hand strength. Music to movement – which is a specific type[44] of active music intervention that is not led by a music therapist – showed a statistically significant difference compared to no active music intervention or yoga on two mobility outcomes (timed Up and Go, p=0.01, Cohen's d = 0.7, moderate effect; 6-minute walk test (p=0.05, Cohen's d = 0.6, moderate effect) in one study[44] (an RCT), although no statistically significant difference was found for 5 times sit-to-stand.

There were three RCTs[56,58,59] on motor imagery, all conducted by the same research group. All music-based groups showed statistically significant improvements in walking in all three studies, but there were no statistically significant differences between the different music-based groups. One study[59] showed synchronisation improved more with music and verbal motor imagery and music-cued motor imagery and worsened with just motor imagery. While one study[59] showed that physical fatigue only showed a statistically significant improvement after music and verbally cued motor imagery, another study[56] showed that this was found only for music-cued imagery. Studies using other interventional designs partly corroborated these findings – showing improved walking speed and distance after all music interventions[55,57] and reduced physical fatigue after music and verbally-cued motor imagery,[57] although one study only showed improvements in walking tests for those classed as 'responders', while response was associated with changes in brain activation patterns.[49] There were no observational studies for this intervention-outcome combination.

There were two RCTs[46,61] that assessed rhythmic auditory stimulation in relation to motor outcomes, both showing improvements in at some gait parameters. In one study, significant improvements were observed in double-support time, while additional parameters including velocity, cadence, stride length and step length consistently favoured the intervention group and were associated with moderate effect sizes despite not reaching statistical significance.[46] There was one study[54] using a different intervention design, which corroborated the findings from RCTs in terms of improvements in gait parameters versus no rhythmic stimulation. There was one observational study,[48] which showed an effect of frequency of rhythmic auditory stimulation on interlimb coordination (0.75Hz was worse than either 1.00Hz or 1.50Hz) with higher cognitive processing speed and attention scores were associated with better coordination.

Cognitive outcomes

Cognitive outcomes were assessed in 8 studies (35%) – profiled in Supplemental files 4-6. Seven studies were interventional – of which five were RCTs[32,52,59,62,63] and 2 were other interventional designs[30,45] – and one was an observational study.[53]

Specifically using neurologic music therapy methods, one study[32] (an RCT) showed statistically significant improvements in cognitive function ($p<0.001$), long-term retrieval ($p=0.007$) and delayed recall ($p=0.001$) in the music therapy group. One study[51] (using a different interventional design) on passive music listening (not led by a music therapist) showed statistically significant within-group effects in the music group on cognitive function ($p<0.001$) and long-term retrieval ($p<0.007$). There was no statistically significant difference versus conventional cognitive rehabilitation. It should be noted that conventional cognitive rehabilitation was only administered in the control arm for this study.

One study[53] (observational) assessed rhythmic auditory stimulation and found that people with MS perceived statistically significantly lower cognitive fatigue ($p<0.001$) while listening to music.

Psychological outcomes

Psychological outcomes were assessed in 7 studies (30%) – profiled in Supplemental files 4-6. Six studies were interventional – of which 4 were RCTs[32,50,59,60] and two were other interventional designs[45,51] – and one was an observational study.[30]

There was one RCT[60] that assessed active music therapy and showed that – from pre-test to post-test to follow-up, music therapy statistically significantly reduced depression and anxiety and improved self-esteem. One study[45] using a different interventional design did not find statistically significant differences versus control but did show medium effect sizes for improvements in self-esteem (Cohen's $d=0.54$), depression (Cohen's $d=0.64$), and anxiety (Cohen's $d= 0.63$). One observational study reported significant within-group improvements in depression ($p=0.036$), anxiety ($p=0.013$), and self-acceptance ($p=0.012$) following music therapy, however no significant differences were observed compared with the control group.[30] One study[50] (an RCT) assessed passive music therapy (listening to a monochord while lying on it) and showed statistically significant benefits for music therapy versus a silent condition lying on the monochord while it is not being played on psychosocial fatigue ($p=0.029$) and stronger immediate subjective effects ($p<0.001$), although the numerical improvement in anxiety outcomes did not reach statistical significance ($p=0.109$). One study[51] (using a different interventional design) showed statistically significant within-group improvements for passive music listening (not led by a music therapist) on motivation, emotional awareness and depression. There were no statistically significant differences versus conventional cognitive rehabilitation, which was only administered in the control arm.

Quality of life outcomes

QoL-related outcomes were assessed in 12 studies (52%) – profiled in Supplemental files 4-6. Ten studies were interventional – of which 6 were RCTs[32,44,50,56,58,59] and four were other interventional designs[45,51,55,57] – and two were observational.[30,53]

One RCT found that adding neurologic music therapy to conventional cognitive rehabilitation was associated with greater improvement in the mental health component of the MSQoL-54 ($p<0.001$) than conventional cognitive rehabilitation alone.[32] One interventional study[45] (using a different interventional design) and one observational study[30] of active music therapy, reported no significant improvement in QoL compared with a matched control group. Evidence for QoL and wellbeing outcomes was mixed. Studies assessing QoL directly reported variable findings, with significant improvements observed following passive music listening[50] and music-assisted gait training[51], whereas active music therapy studies found no significant improvements in overall QoL.[30,45] Studies assessing related outcomes such as fatigue and pain interference also produced mixed findings, reporting a trend towards reduced fatigue but no significant effect on pain interference.[44]

Three RCTs assessed motor imagery[56,58,59] In two RCTs,[56,58] QoL showed a statistically significant improvement only after music and verbally cued motor imagery. A smaller feasibility study[57] using a different interventional design provided preliminary evidence to support a benefit on QoL.

Summary of synthesis and risk of bias

Overall, current evidence suggests that MBTs may provide benefits across motor, cognitive, psychological, and QoL domains in people with MS. However, heterogeneity in intervention type, outcome measures, control arms, and study design limits the strength of conclusions that can be drawn, and several outcomes were assessed in only a small number of studies. The risk of bias profile as well as the potential impact of risk of bias on the outcomes of the narrative synthesis are presented in online supplemental file 10. Potential outcome reporting bias was not evaluable in 13 studies (57%) as no protocol was available. In five studies (22%), all outcomes from the protocol were reported in the journal article and in one further study all primary outcomes were reported. In two studies (9%), primary outcomes from the protocol were not reported in the journal article, while in two further studies (9%), the primary outcomes from the protocol were reported in the journal article but were switched to secondary outcomes. Further details can be found in online supplemental file 11. The effect direction plot (supplemental file 12) shows that all studies were classed as small or medium (as there were no studies with a sample size more than 300) – however there was no evidence that the results of studies differed materially dependent on sample size.

Clinical significance

Available evidence consistently supported a clinically significant benefit of MBTs on depressive symptoms as assessed by the Beck depression scale. However, less consistent evidence of a clinically significant benefit was found for motor function assessed by the timed 25-foot walk test and QoL assessed by the MSIS-29 physical subscale. Meanwhile, the only evaluable study for anxiety assessed by HADS-anxiety subscale did not find evidence of a clinically significant benefit. Further details can be found in supplemental file 13.

Certainty assessment

Certainty of evidence was assessed as Low for all outcome domains using the GRADE schemata, reflecting potential issues around heterogeneity and the presence of studies where multiple CASP domains were assessed at potential high risk of bias, although most included studies for each outcome domain were RCTs. All studies assessed within-group rather than between-group differences, although for some studies information was available for more than one group.

DISCUSSION

Summary

This systematic review synthesised evidence on MBTs for rehabilitation outcomes in people with MS, identifying 23 eligible studies published between 1999 and 2025. The value of a systematic review comes in the number of studies integrated in the same systematic review rather than how many studies had featured in any previous systematic review.[64] No previous systematic review had included more than 12[26-27] of the 23 studies (52%) that we identified. Indeed, we double the amount of evidence included in the most recent related review by Philips et al.[27] By doubling the amount of evidence on MBTs in MS that has been integrated together compared to any previous systematic review, we offer a clear advance in knowledge. Furthermore, the present review adds an assessment of clinical significance and certainty of findings. Across the 23 studies, outcomes could be mapped to four rehabilitation domains,

For peer review only

with motor outcomes most frequently assessed (16 studies), followed by QoL and related outcomes (12 studies), cognitive outcomes (8 studies), and psychological outcomes (7 studies). The most evidence among motor outcomes was found for gait and mobility, particularly where interventions used rhythmic auditory stimulation and music cued motor imagery, with improvements reported in walking speed, walking distance, cadence, stride length, and gait symmetry. Cognitive findings were mixed, with clearer benefits in music assisted learning paradigms (e.g. sung versus spoken word learning), while broader cognitive rehabilitation comparisons suggested similar rather than superior benefits relative to conventional rehabilitation. Psychological outcomes showed potential improvements in depression, anxiety, self-acceptance and self-esteem, but between group effects were not uniform. QoL and fatigue were frequently co-assessed, with several trials reporting improvements in fatigue and MS-specific QoL measures, though findings were heterogeneous. Importantly, this review incorporated studies that have been less emphasised in prior summaries and were necessary to include for a complete synthesis. Challenges were identified in terms of certainty of evidence as assessed by GRADE and reaching clinical significance thresholds across the sample for some outcomes. These may reflect the emerging nature of the evidence base for MBTs in MS, the arts in health more generally, and the methodological challenges associated therewith.

Interpretation of findings

This review extends and updates the MS rehabilitation literature on MBTs by both increasing the quantity of included evidence and the scope of interventions and outcomes compared with prior syntheses. A key interpretive issue is the distinction between active and receptive MBTs. The evidence base remains weighted toward receptive approaches, particularly music listening during walking or cueing based strategies. Phillips et al[27] concluded that most included interventions were receptive, with fewer active interventions, and highlighted gait parameters and fatigue as the most common outcomes. The findings in the present review corroborate that pattern using a much larger evidence base but also suggest that the active versus receptive categorisation favoured by Phillips et al[27] may be too simplistic for MS rehabilitation. Phillips et al[27] searched from 2002 to June 2024 and included 15 studies,[27] three of which did not meet the inclusion criteria for our review as the interventions were not MBTs (see Table 1 for details). Furthermore, Phillips et al[27] (due to their 2002 search date limit) excluded key earlier work[19] as well as not identifying studies published since their June 2024 search date. The inclusion of Kuhlmann et al[50] in our review provides additional randomised evidence on receptive vibration-based therapy effects on fatigue and pain perception, while Helmlinger et al[49] contributes neuroimaging correlated outcomes that are not represented in reviews with earlier search cut-offs. While group singing-based interventions were shown to be common in Parkinson's disease (PD),[8] no evidence on these types of MBTs was identified in our review on MS. Across outcomes, evidence from our review suggests the greatest benefit for gait and mobility, with more heterogeneous findings for cognition and psychological outcomes. This likely reflects differences in intervention intent and dose, diversity in control arms, and inconsistent outcome measures. While there were multiple studies by the same authors,[49,55-59] they were separate studies, and except for one pair of studies that had a small overlap in sample (3%), it did not appear that there were overlapping participants (Supplemental file 11). However, comparability of findings across studies may be limited by differences in control arms across studies, meaning that MBTs are not always being compared to interventions of equivalent effectiveness. A further limitation of the evidence base was that the follow-up assessment typically took place shortly after the intervention, meaning we lack information about long-term benefit. This is a common limitation with clinical trials and could be addressed through observational registry studies.[65] As sample sizes were typically small, there is a possibility of findings being affected by small-sample bias. Furthermore, arts interventions are difficult to blind and therefore blinding is seldom conducted in research on the arts in health.[5,8,9] Therefore, the possibility of performance bias due to non-blinded

For peer review only

interventions should be considered. Overall, based on the available evidence, MBTs appeared promising across multiple rehabilitation domains in MS, but the evidence remains constrained by intervention heterogeneity, limited head-to-head comparisons of types of MBTs, and variable methodological reporting, which together limit the certainty of any single conclusion.

Strengths and limitations

This systematic review has several strengths. We used a comprehensive search strategy across multiple databases, followed PRISMA reporting standards,[34] and applied predefined eligibility criteria. The final was search conducted in July 2025, within six months of original manuscript submission (January 2026), which strengthens the currency of the evidence base.[66] The review adhered to PRISMA[34] reporting standards and applied predefined eligibility criteria, reducing the risk of selection bias. Data extraction was conducted using standardised forms, and risk of bias was assessed using design-appropriate CASP tools,[36] supporting consistency across heterogeneous study designs. The review also incorporated assessments of clinical significance and certainty of evidence, allowing interpretation beyond statistical significance alone.

However, limitations should be acknowledged. First, meta-analysis was not feasible because of substantial heterogeneity in study design, intervention type, comparator condition, intervention dose, and outcome measurement. As a result, the synthesis relied on narrative methods, which are less precise than quantitative pooling. Second, many included studies had small samples and were exploratory or pilot in nature, limiting the statistical power. Third, although many studies were randomised, blinding of participants and intervention providers was generally not possible because of the nature of MBTs, increasing the potential for performance bias. Also, participant characteristics such as race and ethnicity were not consistently reported, limiting the assessment of generalizability. Finally, only studies published in English were eligible for inclusion – however no studies were excluded on grounds of language of publication, as no studies published in languages other than English that would otherwise be eligible for inclusion were identified. This may reflect limitations[67] in indexing of non-English language journals in major bibliographic databases. It is also noted[68] that subscription coverage of non-English language journals in university libraries in English-speaking countries is limited, which would limit access to full texts of non-English language articles if they had been eligible for inclusion. It was not possible to assess publication bias as the data for most outcomes were not amenable to such aggregation – for many of the same reasons as meta-analysis could not be conducted, including differences in sampling frames and differences in how outcomes were assessed. The groupings in the narrative synthesis were broad and may not reflect granular differences in outcome and intervention types – this was necessary because of the relatively low (n=23) number of total studies.

Implications for research and practice

Future research should prioritise addressing the methodological limitations identified in this review. Larger, adequately powered randomised controlled trials are needed, with clearer reporting[67] of allocation concealment, participant flow, and effect size precision. Greater standardisation of outcome measures, particularly for gait, fatigue, cognition, and QoL, would enhance comparability and enable future quantitative synthesis. Reporting of clinically meaningful change, alongside statistical significance, should be prioritised to support translation into practice. There is a clear need for studies that directly compare different MBTs, including active versus receptive therapies. The relative lack of research on singing-based interventions in MS represents an evidence gap, which contrasts with evidence in Parkinson's disease, where singing is a frequently studied therapeutic modality.[8] Future studies should also expand beyond gait-focused outcomes to include communication, social participation, and patient-prioritised outcomes. From a clinical perspective, the current evidence base

For peer review only

supports cautious integration of MBTs, particularly rhythmic cueing and music-cued motor imagery, within MS rehabilitation programmes. These interventions appear feasible in both facility and home-based settings and may complement conventional physiotherapy and cognitive rehabilitation. However, given heterogeneity and residual risk of bias, MBTs should currently be considered an adjunct rather than a replacement for established rehabilitation approaches.

Conclusion

This systematic review synthesised evidence from 23 studies examining the effects of MBTs on motor, cognitive, psychological, and QoL outcomes in people with multiple sclerosis. The narrative synthesis suggests that MBTs, particularly rhythmic auditory stimulation and music-cued motor imagery, may provide benefits for gait and mobility, with more variable evidence for cognitive, psychological, and QoL outcomes. Evidence for cognitive benefit was found most in music-assisted learning paradigms, while psychological and fatigue-related outcomes showed mixed but promising signals.

This review extends prior rehabilitation-focused syntheses by including a broader range of intervention types, outcome domains, and recent studies, while maintaining methodological consistency through exclusive use of CASP risk of bias tools. Nonetheless, heterogeneity in interventions, outcomes, and study design limits the strength of conclusions. Overall, MBTs appear to be a promising adjunct in MS rehabilitation, but higher-quality, standardised research is required to clarify optimal intervention models and establish clinical effectiveness.

DECLARATIONS

Contributors. MSB provided some initial suggestions of topic areas in which he would be willing to supervise a student project on the Royal Society for Medicine systematic review programme for medical students. NA suggested a specific topic for this review which, through discussion, NA and MSB refined to form the protocol for this systematic review. NA designed the database searches, refined them through discussion with MSB, and ran these searches. MSB designed and conducted the supplementary searches and citation chasing. Screening, data extraction and risk of bias assessment were conducted by NA and MSB. NA and MSB conducted the narrative synthesis and interpreted the findings. MSB conducted the GRADE analysis. NA wrote the first draft of the manuscript, which was initially revised by MSB prior to circulation to all authors. MSB wrote the supplemental materials. DW and JK provided valuable contributions to interpreting the findings and revised the manuscript for substantive intellectual content. All authors read the final version of the manuscript prior to submission and take responsibility for their own work as appropriate. NA is a medical student and her work on this project was supervised by MSB (Senior Research Fellow). JK is a postdoctoral fellow and her work on this project was supervised by DW (Assistant Professor and centre Co-Director). NA was the project manager and MSB was the project director. MSB is the guarantor and takes overall responsibility for the work.

Acknowledgements. This work was done under the auspices of the Royal Society of Medicine systematic review programme for medical students. NA was a co-ordinator of the programme as well as a participant. MSB supervised NA on this programme. The programme did not provide any specific funding for this work. DW is Assistant Professor in Physical Medicine and Rehabilitation and Co-Director (Arts & Medicine), Center for History, Humanities, Arts, Social Sciences & Ethics in Medicine, University of Michigan, Ann Arbor, USA. MSB previously received expenses but not payment for arts promotion activities, however these ceased in 2010 and consequently no longer represent a competing interest.

Funding. The authors have not declared a specific grant for this research from any funding agency in the public, commercial or not-for-profit sectors.

For peer review only

Competing interests. The authors declare no competing interests.

Patient and public involvement. Patients and/or the public were not involved in the design, or conduct, or reporting, or dissemination plans of this research.

Patient consent for publication. Not applicable.

Ethics approval. No ethical approval was required as this is a systematic review. The University contact for research integrity is researchintegrity@exeter.ac.uk.

Conference presentation. This work has not been published or submitted to any other journal. A conference abstract has been accepted an oral presentation at the 2nd International Meeting of Arts Prescribing in HealthCare, Olympus Riviera, Greece, April 2026.

Data availability statement. All data relevant to the study are included in the article or uploaded as supplementary information. The presented work is a systematic review. All relevant information is provided in the manuscript and appendices. This includes the data extraction form completed with the data from all included studies. No meta-analyses were conducted, so there is no analytical code.

REFERENCES

1. Portaccio E, Magyari M, Havrdova EK, et al. Multiple sclerosis: emerging epidemiological trends and redefining the clinical course. *Lancet Reg Health Eur;* 2024;44: 100977.
2. GBD 2016 Multiple Sclerosis Collaborators. Global, regional, and national burden of multiple sclerosis 1990–2016: a systematic analysis for the Global Burden of Disease Study 2016. *Lancet Neurol* 2019;18(3):269-85.
3. Jakimovski D, Bittner S, Zivadinov R, et al. Multiple sclerosis. *Lancet* 2023;403 (10422): 183-202.
4. Davis E, Webster A, Whiteside B, et al. Dance for multiple sclerosis: a systematic review. *Int J MS Care* 2023; 25(4): 176-85.
5. Barnish MS, Nelson-Horne B. Group-based active artistic interventions for adults with primary anxiety and depression: a systematic review. *BMJ Open* 2023;13(6):e069310.
6. Pérez-Eizaguirre M, Vergara-Moragues E. Music therapy interventions in palliative care: a systematic review. *J Palliat Care* 2021;36(3):194–205.
7. Liu Q, Li W, Yin Y, et al. The effect of music therapy on language recovery in patients with aphasia after stroke: a systematic review and meta-analysis. *Neurol Sci* 2022;42(3):863–72.
8. Barnish MS, Reynolds SE, Nelson-Horne RV. Active group-based performing arts interventions in Parkinson's disease: an updated systematic review and meta-analysis. *BMJ Open* 2025;15(4):e089920.
9. Barnish MS, Barran SM. A systematic review of active group-based dance, singing, music therapy and theatrical interventions for quality of life, functional communication, speech, motor function and cognitive status in Parkinson's disease. *BMC Neurol* 2020;20(1):371.
10. Barnish J, Atkinson RAA, Barran SM, et al. Potential benefit of singing for people with Parkinson's disease: a systematic review. *J Parkinsons Dis* 2016;6(3):473–84.
11. American Music Therapy Association. What is music therapy? Available at:// https://www.musictherapy.org/about/musictherapy/ [Accessed 8 May 2026].
12. British Association for Music Therapy. What is a music therapist? Available at :// https://www.bamt.org/music-therapy/what-is-a-music-therapist [Accessed 8 May 2026].
13. Canadian Association of Music Therapists. About music therapy. Available at:// https://www.musictherapy.ca/about-camt-music-therapy/about-music-therapy/ [Accessed 8 May 2026].
14. Mainka S, Weymann E. Music therapy newly defined. Approaches 2025; 17(4): 812-24.

15. Skanland MS. Music therapy and social recovery in flexible assertive community treatment. Nord J Music Ther 2023; 32(4): 290-306.
16. Nebelung I, Kruger V. Norway: country report on professional recognition of music therapy. Approaches 2015; 7(1): 171-2.
17. Xiao H, Chen C. The study of traditional Chinese medicine five-element music therapy for depression: a bibliometric analysis. Digit Chin Med 2025; 8(4): 558-70.
18. Spiro N, Sanfilippo KRM (Eds.). Collaborative insights: interdisciplinary perspectives on musical care throughout the life course. Oxford: Oxford University Press; 2022.
19. Lin TH, Liao YC, Tam KW, et al. Effects of music therapy on cognition, quality of life, and neuropsychiatric symptoms of patients with dementia: A systematic review and meta-analysis of randomized controlled trials. *Psychiatry Res* 2023; 329: 115498.
20. Lopes J, Keppers II. Music-based therapy in rehabilitation of people with multiple sclerosis: a systematic review of clinical trials. *Arq Neuropsiquiatr* 2021;79(6):527–35.
21. Stegemann T, Geretsegger M, Phan QC, et al. Music therapy and other music-based interventions in pediatric health care: an overview. *Medicines (Basel)* 2019; 6(1): 25.
22. Raglio A. A novel music-based therapeutic approach: the Therapeutic Music Listening. Front Hum Neurosci 2023; 17: 1204593.
23. Wolff L, Quan Y, Perry G, et al. Music engagement as a source of cognitive reserve. Am J Alzheimers Dis Other Demen 2023; 38: 15333175231214833.
24. Gardener SH, Mukaetova-Ladinska EB, Perera NA. The effect of music therapy on psychological outcomes for neurological conditions: a systematic review. *Medicina* 2025;61(9):1611.
25. Kong L, Zhang X, Meng L, et al. Effects of music therapy intervention on gait disorders in persons with multiple sclerosis: a systematic review of clinical trials. *Mult Scler Relat Disord* 2023;73:104629.
26. Ostermann T, Schmid W. Music therapy in the treatment of multiple sclerosis: a comprehensive literature review. *Expert Rev Neurother* 2006;6(4):469–77.
27. Phillips CS, Kim J, Ganesh A, et al. Impact of active versus receptive music interventions on psychosocial and neurological outcomes in people with multiple sclerosis: a systematic review. *J Integr Complement Med* 2025;31(10):889–903.
28. Tan Y, Chen X, Wang L, et al. Music interventions for gait and mobility in multiple sclerosis: a scoping review. *Mult Scler Relat Disord* 2025;101: 106563.
29. Manocchio L, Moriano C, D'Amato A, et al. Beyond cognition: cognitive re-education and psychological wellbeing in people with multiple sclerosis: a narrative review. *NeuroSci* 2025; 6(3): 45.
30. Schmid W, Aldridge D. Active music therapy in the treatment of multiple sclerosis patients: a matched control study. *J Music Ther* 2004;41(3):225–40.
31. Wiens ME, Reimer MA, Guyn HL. Music therapy as a treatment method for improving respiratory muscle strength in patients with advanced multiple sclerosis: a pilot study. *Rehabil Nurs* 1999;24(2):74–80.
32. Impellizzeri F, Leonardi S, Latella D, et al. An integrative cognitive rehabilitation using neurologic music therapy in multiple sclerosis: a pilot study. *Medicine (Baltimore)* 2020;99(4):e18866.
33. Munn Z, Peters MDJ, Stern C, et al. Systematic review or scoping review? Guidance for authors when choosing between a systematic or scoping review approach. *BMC Med Res Methodol* 2018; 18(1): 143.
34. Page MJ, McKenzie JE, Bossuyt PM, et al. The PRISMA 2020 statement: an updated guideline for reporting systematic reviews. *BMJ* 2021;372:n71.
35. Campbell M, McKenzie JE, Sowden A, et al. Synthesis without meta-analysis (SWiM) in systematic reviews: reporting guideline. BMJ 2020; 368: l6890.
36. Critical Appraisal Skills Programme. CASP checklists [online]. https://casp-uk.net/casp-tools-checklists/ (accessed 12 December 2025).
37. Viswanathan M, Ansari NT, Berkman ND, et al. Assessing the risk of bias of individual studies in systematic reviews of health care interventions. In: Agency for Healthcare Research and Quality (US). Methods Guide for Effectiveness and Comparative

Effectiveness Reviews. Rockville (MD): Agency for Healthcare Research and Quality; 2008.
38. Johnson BT, Hennessy EA. Systematic reviews and meta-analyses in the health sciences: Best practice methods for research syntheses. *Soc Sci Med* 2019; 233: 237-51.
39. Jaeschke R, Singer J, Guyatt GH. Measurement of health status. Ascertaining the minimal clinically important difference. *Control Clin Trials* 1989;10(4):407-415.
40. Copay AG, Subach BR, Glassman SD, et al. Understanding the minimum clinically important difference (MCID) of patient-reported outcome measures. Spine J 2007; 7(3): 333-43.
41. Engel L, Beaton D E, Touma Z. Minimal clinically important difference: a review of outcome measure score interpretation. *Rheum Dis Clin* 2018*:* 44(2), 177-88.
42. The GRADE Working Group. The GRADE Book. Available at:// https://book.gradepro.org/about [Accessed 10 May 2026].
43. Schunemann H, Brozek J, Guyatt G, et al (Editors). GRADE handbook for grading quality of evidence and strength of recommendations. The GRADE Working Group; 2013
44. Young HJ, Mehta TS, Herman C, et al. The effects of M2M and adapted yoga on physical and psychosocial outcomes in people with multiple sclerosis. *Arch Phys Med Rehabil* 2019;100(3):391–400.
45. Aldridge D, Schmid W, Kaeder M, et al. Functionality or aesthetics? A pilot study of music therapy in the treatment of multiple sclerosis patients. *Complement Ther Med* 2005;13(1):25–33.
46. Conklyn D, Stough D, Novak E, et al. A home-based walking program using rhythmic auditory stimulation improves gait performance in patients with multiple sclerosis: a pilot study. *Neurorehabil Neural Repair* 2010;24(9):835–42.
47. Gatti R, Tettamanti A, Lambiase S, et al. Improving hand functional use in subjects with multiple sclerosis using a musical keyboard: a randomized controlled trial. *Physiother Res Int* 2015;20(2):100–7.
48. Goetschalckx M, Van Geel F, Meesen R, et al. Rhythmic interlimb coordination of the lower limbs in multiple sclerosis during auditory pacing to three different frequencies. *Gait Posture* 2021;86:334–40.
49. Helmlinger B, Seebacher B, Ropele S, et al. Effects of rhythmic-cued gait training on gait-like task related brain activation in people with multiple sclerosis. *J Neurol Sci* 2025;471:123426.
50. Kuhlmann J, Ebner K, Zimmer A, et al. Music therapy with a monochord in multiple sclerosis ("MUTIMS"): a randomized, controlled, rater-blinded trial. *Mult Scler J Exp Transl Clin* 2025;11(3):20552173251352712.
51. Maggio MG, Tripoli D, Porcari B, et al. How may patients with MS benefit from using music assisted therapy? A case-control feasibility study investigating motor outcomes and beyond. *Mult Scler Relat Disord* 2021;48:102713.
52. Moore KS, Peterson DA, O'Shea G, et al. The effectiveness of music as a mnemonic device on recognition memory for people with multiple sclerosis. *J Music Ther* 2008;45(3):307–29.
53. Moumdjian L, Moens B, Maes PJ, et al. Continuous 12 min walking to music, metronomes and in silence: auditory-motor coupling and its effects on perceived fatigue, motivation and gait in persons with multiple sclerosis. *Mult Scler Relat Disord* 2019;35:92–9.
54. Naderi S, Sadeghi H, Amirseyfaddini M. The effects of rhythmic auditory stimulation with different tempos on spatio-temporal parameters and gait symmetry in patients with multiple sclerosis: a quasi-experimental study. *J Res Rehabil Sci* 2022;18(1): 92-9.
55. Seebacher B, Kuisma R, Glynn A, et al. Rhythmic cued motor imagery and walking in people with multiple sclerosis: a randomized controlled feasibility study. *Pilot Feasibility Stud* 2015;1:25.

For peer review only

56. Seebacher B, Kuisma R, Glynn A, et al. The effect of rhythmic-cued motor imagery on walking, fatigue and quality of life in people with multiple sclerosis: a randomized controlled trial. *Mult Scler J* 2017;23(2):286–96.
57. Seebacher B, Kuisma R, Glynn A, et al. Exploring cued and non-cued motor imagery interventions in people with multiple sclerosis: a randomized feasibility trial and reliability study. *Arch Physiother* 2018;8:6.
58. Seebacher B, Kuisma R, Glynn A, et al. Effects and mechanisms of differently cued and non-cued motor imagery in people with multiple sclerosis: a randomised controlled trial. Mult Scler J 2019; 25(12): 1593-1604.
59. Seebacher B, Helmlinger B, Pinter D, et al. Actual and imagined music-cued gait training in people with multiple sclerosis: a double-blind randomized parallel multicenter trial. *Neurorehabil Neural Repair* 2024;38(8):555–69.
60. Seighalani MZ, Ghahari S, Zarbakhsh M. The effectiveness of music therapy on depression, anxiety and self-esteem of patients with multiple sclerosis. *Adv Environ Biol* 2014;8(9):253–60.
61. Shahraki M, Sohrabi M, Taheri Torbati HR, et al. Effect of rhythmic auditory stimulation on gait kinematic parameters of patients with multiple sclerosis. *J Med Life* 2017;10(1):33–7.
62. Thaut MH, Peterson DA, Sena KM, et al. Musical structure facilitates verbal learning in multiple sclerosis. *Music Percept* 2008;25(4):325–30.
63. Thaut MH, Peterson DA, McIntosh GC, et al. Music mnemonics aid verbal memory and induce learning-related brain plasticity in multiple sclerosis. *Front Hum Neurosci* 2014;8:395.
64. Ioannidis JP The mass production of redundant, misleading, and conflicted systematic reviews and meta-analyses. *Millbank Q* 2016; 94(3): 485-514.
65. Barnish MS, Turner S. The value of pragmatic and observational studies in health care and public health. *Pragmat Obs Res*.2017;8:49-55.
66. Higgins JPT, Thomas J, Chandler J, et al (Eds). *Cochrane Handbook for Systematic Reviews of Interventions* version 6.5 (updated August 2024). Available at:// www.cochrane.org/handbook [Last accessed 6 January 2026].
67. Aali G, Shokraneh, F. No limitations to language, date, publication type, and publication status in search step of systematic reviews. *J Clin Epidemiol 2021; 133: 165-7.*
68. Absher LU, Desilets MR. English as the scholarly language: diversity, equity, and inclusion implications for academic reference and instruction librarians. *Libr Q* 2024; 94(3): 296-315.
69. Robb SL, Story KM, Harman E, et al. Reporting Guidelines for Music-based Interventions checklist: explanation and elaboration guide. Front Psychol 2025;16:1552659.
70. Ng A, Bunyan S, Suh J, et al. Ballroom dance for persons with multiple sclerosis: a pilot feasibility study. *Disabil Rehabil* 2020;42(8):1115–21.
71. Van Geel F, Van Asch P, Veldkamp R, et al. Effects of a 10-week multimodal dance and art intervention program leading to a public performance in persons with multiple sclerosis: a controlled pilot trial. *Mult Scler Relat Disord* 2020;44:102256.
72. Basturk S, Ekici G, Kirteke F, et al. Therapeutic effects of line dancing in people with multiple sclerosis: an evaluator-blinded randomized controlled study. *Arts Health* 2024; doi: 10.1080/17533015.2024.2325425 [Online ahead of print].
73. Baram Y, Miller A. Auditory feedback control for improvement of gait in patients with multiple sclerosis. *J Neurol Sci* 2007;254 (1-2):90–4.
74. Moumdjian L, Maes PJ, Dalla Bella S, et al. Detrended fluctuation analysis of gait dynamics when entraining to music and metronomes at different tempi in persons with multiple sclerosis. *Sci Rep* 2020;10(1):12934.
75. Moumdjian L, Moens B, Maes PJ, et al. Walking to music and metronome at various tempi in persons with multiple sclerosis: a basis for rehabilitation. *Neurorehabil Neural Repair* 2019;33(6):464–75.

For peer review only

76. Coleman CI, Sobieraj DM, Marinucci LN. Minimally important clinical difference of the Timed 25-Foot Walk Test: results from a randomized controlled trial in patients with multiple sclerosis. *Curr Med Res Opin*. 2012;28(1):49-56.
77. Button KS, Kounali D, Thomas L, et al. Minimal clinically important difference on the Beck Depression Inventory--II according to the patient's perspective. *Psychol Med*. 2015;45(15):3269-79.
78. Lemay KR, Tulloch HE, Pipe AL, et al. Establishing the minimal clinically important difference for the Hospital Anxiety and Depression Scale in patients with cardiovascular disease. *J Cardiopulm Rehabil Prev* 2019; 39(6): E6-11.
79. Puhan MA, Frey M, Buchi S, et al. The minimal important difference of the hospital anxiety and depression scale in patients with chronic obstructive pulmonary disease. *Health Qual Life Outcomes* 2008: 2(6):46.
80. Canadian Agency for Drugs and Technologies in Health. *Clinical review report: Daclizumab (Zinbryta*). Ottawa (ON): CADTH; 2017.
81. Costelloe L, O'Rourke K, Kearney H, et al. The patient knows best: significant change in the physical component of the Multiple Sclerosis Impact Scale (MSIS-29 physical). *J Neurol Neurosurg Psychiatry* 2007;78(8):841-
82. Boon MH, Thomson H. The effect direction plot revisited: Application of the 2019 Cochrane Handbook guidance on alternative synthesis methods. *Res Synth Methods* 2020; 12(1): 29-33.

For peer review only

TABLES AND FIGURES

Box 1. Inclusion criteria for the systematic review

Eligible studies assessed:

- Population: People with a diagnosis of MS
- Intervention: Studies involving MBTs or music interventions delivered for the purpose of rehabilitation in people with multiple sclerosis. Interventions could include:
    - Active music-making (e.g., instrument playing)
    - Passive listening to music (pre-recorded or live)
    - Rhythmic auditory stimulation (RAS)
    - Structured MT delivered by a trained therapist
    - Group or individual sessions of MBTs
- Control arms: Studies with and without control arms were eligible. There were no specific requirements for what control arms should involve.
- Outcomes: Studies that report rehabilitation-related outcomes: functional recovery, motor function, gait and balance, cognitive function, psychological well-being, QoL.
- Other: Studies available in full-text and published in the English language involving human participants aged 18 and over with a confirmed diagnosis of multiple sclerosis. Studies conducted in any healthcare, community, or rehabilitation setting, provided they meet the control arm and outcome criteria outlined above.

Given the emergent evidence base, we included studies that provided data that were informative for the listed outcome domains but did not cover the full breadth of the domain. For example, the MSFC scale covers cognition but is not exclusively on cognition but was considered relevant. Furthermore, studies addressing pain, general fatigue, and quality of life subscales rather than a formal quality of life scale that covers the full breadth of quality of life were considered relevant.

Box 2. Data Extracted

The following information was extracted for each included study:

- Bibliographic details (authors, year and citation).
- Country of study.
- Study design.
- Participants (sample size, sex profile, mean age or age range, MS type, disease duration, EDSS score, use of walking aids, left/right-handed – as this variable is often considered in neuropsychological studies).
- Inclusion criteria.
- Outcomes.
- Content of intervention.
- Professional background of intervention leader – as work in PD[8] has shown large differences in leader background and expertise within and between studies.
- Location of intervention (eg, health centre or home).
- Frequency and duration of intervention.
- Content of control arm.
- Professional background of leader of intervention delivered to the control arm.
- Location of control arm (eg, health centre or home).
- Frequency and duration of control arm.
- Study results for narrative synthesis for all eligible reported outcomes.

For peer review only - http://bmjopen.bmj.com/site/about/guidelines.xhtml

Table 1. Previous systematic, scoping and comprehensive reviews

Authors	Method	Search date	PROSPERO	Interventions	Outcomes	Included studies
Gardener et al, 2025[24]	Systematic review (without meta-analysis)	January 2025 (limited to literature since 2015)	Not registered, no pre-published protocol	MT	Psychological outcomes	Wider range of neurological conditions including MS. For MS:<br>1 pilot RCT (Impellizeri et al, 2020).[32]
Kong et al, 2023[25]	Systematic review (without meta-analysis)	October 2022	CRD 42022365668	MT	Gait and mobility outcomes	2 case series. 7 RCTs (Conklyn et al, 2010;[46] Seebacher et al, 2015;[55] Seebacher et al, 2017;[56] Seebacher et al, 2018;[57] Seebacher et al, 2019;[58] Shahraki et al, 2017;[61] Young et al, 2019).[44]<br>3 non-RCTs (Maggio et al, 2021;[51] Ng et al, 2020;[70] Van Geel et al, 2020).[71]
Lopes et al, 2021[20]	Systematic review (without meta-analysis)	July 2020	CRD 42020143080	MBTs	Balance, cognition, dexterity, fatigue, gait, pain, QoL	10 clinical trials (Aldridge et al, 2005;[45] Conklyn et al, 2010;[46] Gatti et al, 2015;[47] Van Geel et al, 2020;[71] Impellizeri et al, 2020;[32] Moore et al, 2008;[52] Seebacher et al, 2019;[58] Seebacher et al, 2017;[56] Thaut et al, 2014;[63]* Young et al, 2019).[44]
Ostermann et al, 2006[26]	Comprehensive review	Not stated	Not registered (review pre-dates PROSPERO)	MT	Not specified	8 conference abstracts, chapters or non-peer-reviewed sources.<br>2 case reports or case series.<br>2 pilot studies (Schmid et al 2004;[30] Wiens et al, 1999).[19]

For peer review only

Phillips et al, 2025[27]	Systematic review (without meta-analysis)	June 2024	CRD 42022338291	Music interventions (active or receptive)	Anxiety, balance, cognition, depression, dexterity, fatigue, gait, QoL	10 RCTs (Basturk et al, 2024;[72] Conklyn et al, 2010;[46] Gatti et al, 2015;[47] Impellizzeri et al, 2020;[32] Moore et al, 2008;[52] Seebacher et al, 2017;[56] Seebacher et al, 2019;[58] Thaut et al, 2008;[62] Thaut et al, 2014;[63] Young et al, 2019).[44] 5 quasi-experimental studies (Maggio et al, 2021;[51] Moumdjian et al, 2019;[53] Schmid et al, 2004;[30] Van Geel et al, 2020).[71]
Tan et al, 2025[28]	Scoping review	April 2025	Not registered (but protocol uploaded to Open Science Framework)	Music interventions	Gait and mobility	8 RCTs (Baram & Miller, 2007;[73] Conklyn et al, 2010;[46] Helmlinger et al, 2025;[49] Seebacher et al, 2024;[59] Seebacher et al, 2019;[58] Seebacher et al, 2018;[57] Seebacher et al, 2017;[56] Seebacher et al, 2015;[54] Shahraki et al, 2017).[41]** 3 non-randomised experimental studies (Maggio et al, 2021;[51] Moumdjian et al, 2020;[74] Naderi et al, 2022).[54] One cross-sectional study (Goetschalckx et al, 2021).48 Two case-control studies (Moumdjian et al, 2019a;[53],Moumdjian et al, 2019b).[75] Two case studies.

* The citation for Thaut et al (2014)[63] was incorrect in the bibliography of the cited review but appears to refer to this study. ** The review states 8 RCTs but provides 9 citations – it would appear that 2 papers are being referred to as parts of the same study, but this is not made clear. All included studies from these previous reviews were included in our review with the following exceptions: i) case studies, case reports, case series, conference abstracts, chapters and non-peer-reviewed sources were not eligible publication types for our review; ii) Basturk et al, 2024;[72] Ng et al (2020),[70] and Van Geel et al (2020)[71] were both dance intervention studies which were not eligible for our review because the intervention was not primarily music-based; Baram and Miller (2007)[73] is about auditory feedback control but makes no mention of music and is therefore not eligible for our review; Moumdjian et al. (2019b,[74] 2020)[75] were excluded because they examined movement synchronisation to music or metronome without assessing rehabilitation or clinical outcomes in people with MS. We have checked (May 2026) and the Basturk et al[72] 'online ahead of print' paper from 2024 has not yet been published in its final version.

Table 2. Minimally clinically important differences identified

Outcome domain	Measure	Number of studies	MCID
Motor	Timed 25-foot walk test	6	MCID of a 17.2% improvement in test performance has been proposed based on trial data,[76] which accords well to previous clinical proposals of a 20% improvement
Cognitive	N/A	N/A	No pivotal measures used in at least three studies identified
Psychological	Beck depression	3	No MS-specific MCID identified, however noting clinical significance depends on baseline depression severity, a population-agnostic MCID of a 17.5% reduction from baseline has been proposed[77]

For peer review only

Psychological	HADS anxiety	5	No MS-specific MCID identified, however an MCID of 1.70 points[78] has been found for HADS told in cardiovascular disease and 1.60 points[79] in chronic obstructive pulmonary disease. Halving for the anxiety subscale alone, gives an MCID of 0.80-0.85
QoL	MS Impact Scale-29 (MSIS-29)	3	No overall MCID identified,[80] although an MCID of 8 points has been proposed for the physical subscale.[81]

Table 3. GRADE scoring and explanation

Outcome domain	GRADE	Explanation
Motor	Low	16 studies, of which 10 RCTs (including feasibility RCTs but excluding secondary analysis of RCT data). Starting GRADE High as most of the evidence is randomised (as per GRADE guidance). Downgrade to Moderate because of heterogeneity. Downgrade to Low because the set of studies included evidence considered to be low quality.
Cognitive	Low	8 studies, of which 5 RCTs. Starting GRADE High as most of the evidence is randomised (as per GRADE guidance). Downgrade to Moderate because of heterogeneity. Downgrade to Low because the set of studies included evidence considered to be low quality.
Psychological	Low	7 studies, of which 4 RCTs. Starting GRADE High as most of the evidence is randomised (as per GRADE guidance). Downgrade to Moderate because of heterogeneity. Downgrade to Low because the set of studies included evidence considered to be low quality.
QoL	Low	12 studies, of which 8 RCTs. Starting GRADE High as most of the evidence is randomised (as per GRADE guidance). Downgrade to Moderate because of heterogeneity. Downgrade to Low because the set of studies included evidence considered to be low quality.

Table 4. Distribution of included studies according to rehabilitation outcome domains

First Author, Year	Motor outcomes	Cognitive outcomes	Psychological outcomes	QoL outcomes
Aldridge et al, 2005[45]	–	Global cognitive function	Depression, anxiety, self-acceptance	QoL, functional disability, fatigue
Conklyn et al, 2010[46]	Gait and mobility; muscle strength; spasticity and movement related disability	–	–	Pain; functional disability
Gatti et al, 2015[47]	Upper limb function	–	–	–
Goetschalckx et al, 2021[48]	Balance and stability; coordination and synchronisation	–	–	–
Helminger et al, 2025[49]	Gait and mobility	–	–	–

Impellizzeri et al, 2020[32]	–	Global cognitive function	Mood and emotional processing	MS specific QoL
Kuhlmann et al, 2025[50]	–	–	Anxiety; depression; body perception	Fatigue; pain
Maggio et al, 2021[51]	Gait and mobility; balance and stability	–	Depression; emotional components	MS specific QoL
Moore et al, 2008[52]	–	Learning and memory; executive function and attention	–	–
Moumdjian et al, 2019[53]	Gait and mobility; coordination and synchronisation	Cognitive Fatigue	–	Fatigue
Naderi et al, 2022[54]	Gait and mobility	–	–	–
Schmid et al, 2004[30]	Spasticity and movement related disability	Cognitive function	Depression; anxiety; self-acceptance	QoL; functional disability
Seebacher et al, 2015[55]	Gait and mobility	–	–	Fatigue
Seebacher et al, 2017[56]	Gait and mobility	–	–	QoL; fatigue
Seebacher et al, 2018[57]	Gait and mobility	–	–	QoL; fatigue
Seebacher et al, 2019[58]	Gait and mobility; coordination and synchronisation	–	–	QoL; fatigue
Seebacher et al, 2024[59]	Gait and mobility	Global cognitive function	Anxiety; depression; suicidality	QoL; fatigue
Seighalani et al, 2014[60]	–	–	Depression; anxiety; self esteem	–
Shahraki et al, 2017[61]	Gait and mobility	–	–	–
Thaut et al, 2008[62]	–	Learning and memory	–	–
Thaut et al, 2014[63]	–	Learning and memory; neural synchronisation	–	–
Wiens et al, 1999[31]	Muscle strength (respiratory)	–	–	–
Young et al, 2019[44]	Gait and mobility	–	–	Fatigue; pain

Figure 1. PRISMA 2020 flow diagram. PRISMA, Preferred Reporting Items for Systematic Reviews and Meta-Analyses

For peer review only

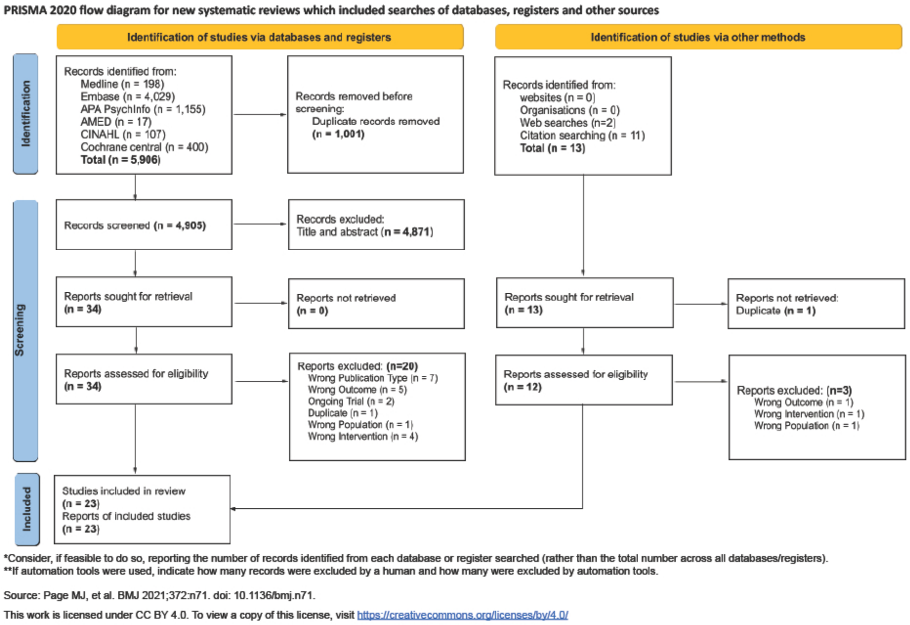

PRISMA 2020 flow diagram. PRISMA, Preferred Reporting Items for Systematic Reviews and Meta-Analyses

361x255mm (72 x 72 DPI)

Ahadi et al. Potential benefits of music-based therapies on rehabilitation outcomes in people with multiple sclerosis: a systematic review. Supplementary files. R1.
Supplemental file 1. Completed PRISMA 2020 checklist

Section and Topic	Item #	Checklist item	Location where item is reported
**TITLE**			
Title	1	Identify the report as a systematic review.	1
**ABSTRACT**			
Abstract	2	See the PRISMA 2020 for Abstracts checklist.	2
**INTRODUCTION**			
Rationale	3	Describe the rationale for the review in the context of existing knowledge.	5-6
Objectives	4	Provide an explicit statement of the objective(s) or question(s) the review addresses.	6
**METHODS**			
Eligibility criteria	5	Specify the inclusion and exclusion criteria for the review and how studies were grouped for the syntheses.	6-8
Information sources	6	Specify all databases, registers, websites, organisations, reference lists and other sources searched or consulted to identify studies. Specify the date when each source was last searched or consulted.	6, Supp file 3
Search strategy	7	Present the full search strategies for all databases, registers and websites, including any filters and limits used.	Supp file 3
Selection process	8	Specify the methods used to decide whether a study met the inclusion criteria of the review, including how many reviewers screened each record and each report retrieved, whether they worked independently, and if applicable, details of automation tools used in the process.	6-7
Data collection process	9	Specify the methods used to collect data from reports, including how many reviewers collected data from each report, whether they worked independently, any processes for obtaining or confirming data from study investigators, and if applicable, details of automation tools used in the process.	7
Data items	10a	List and define all outcomes for which data were sought. Specify whether all results that were compatible with each outcome domain in each study were sought (e.g. for all measures, time points, analyses), and if not, the methods used to decide which results to collect.	7, Table 3
	10b	List and define all other variables for which data were sought (e.g. participant and intervention characteristics, funding sources). Describe any assumptions made about any missing or unclear information.	7, Table 3
Study risk of bias assessment	11	Specify the methods used to assess risk of bias in the included studies, including details of the tool(s) used, how many reviewers assessed each study and whether they worked independently, and if applicable, details of automation tools used in the process.	7
Effect measures	12	Specify for each outcome the effect measure(s) (e.g. risk ratio, mean difference) used in the synthesis or presentation of results.	N/A – no meta-analysis

For peer review only

Section and Topic	Item #	Checklist item	Location where item is reported
Synthesis methods	13a	Describe the processes used to decide which studies were eligible for each synthesis (e.g. tabulating the study intervention characteristics and comparing against the planned groups for each synthesis (item #5)).	6-8
	13b	Describe any methods required to prepare the data for presentation or synthesis, such as handling of missing summary statistics, or data conversions.	N/A – no meta-analysis
	13c	Describe any methods used to tabulate or visually display results of individual studies and syntheses.	N/A – no meta-analysis
	13d	Describe any methods used to synthesize results and provide a rationale for the choice(s). If meta-analysis was performed, describe the model(s), method(s) to identify the presence and extent of statistical heterogeneity, and software package(s) used.	7-8
	13e	Describe any methods used to explore possible causes of heterogeneity among study results (e.g. subgroup analysis, meta-regression).	N/A – no meta-analysis
	13f	Describe any sensitivity analyses conducted to assess robustness of the synthesized results.	N/A – no meta-analysis
Reporting bias assessment	14	Describe any methods used to assess risk of bias due to missing results in a synthesis (arising from reporting biases).	N/A – no meta-analysis
Certainty assessment	15	Describe any methods used to assess certainty (or confidence) in the body of evidence for an outcome.	9
**RESULTS**			
Study selection	16a	Describe the results of the search and selection process, from the number of records identified in the search to the number of studies included in the review, ideally using a flow diagram.	Fig 1
	16b	Cite studies that might appear to meet the inclusion criteria, but which were excluded, and explain why they were excluded.	Supp file 5
Study characteristics	17	Cite each included study and present its characteristics.	Supp file 6
Risk of bias in studies	18	Present assessments of risk of bias for each included study.	Supp file 11

For peer review only

Section and Topic	Item #	Checklist item	Location where item is reported
Results of individual studies	19	For all outcomes, present, for each study: (a) summary statistics for each group (where appropriate) and (b) an effect estimate and its precision (e.g. confidence/credible interval), ideally using structured tables or plots.	No meta-analysis, relevant info in Supp file 9
Results of syntheses	20a	For each synthesis, briefly summarise the characteristics and risk of bias among contributing studies.	9-10, Suppl file 10
	20b	Present results of all statistical syntheses conducted. If meta-analysis was done, present for each the summary estimate and its precision (e.g. confidence/credible interval) and measures of statistical heterogeneity. If comparing groups, describe the direction of the effect.	N/A – no meta-analysis
	20c	Present results of all investigations of possible causes of heterogeneity among study results.	N/A – no meta-analysis
	20d	Present results of all sensitivity analyses conducted to assess the robustness of the synthesized results.	N/A – no meta-analysis
Reporting biases	21	Present assessments of risk of bias due to missing results (arising from reporting biases) for each synthesis assessed.	N/A – no meta-analysis
Certainty of evidence	22	Present assessments of certainty (or confidence) in the body of evidence for each outcome assessed.	13, Table 5
**DISCUSSION**			
Discussion	23a	Provide a general interpretation of the results in the context of other evidence.	14-15
	23b	Discuss any limitations of the evidence included in the review.	14-16
	23c	Discuss any limitations of the review processes used.	15-16
	23d	Discuss implications of the results for practice, policy, and future research.	16
**OTHER INFORMATION**			
Registration and protocol	24a	Provide registration information for the review, including register name and registration number, or state that the review was not registered.	6
	24b	Indicate where the review protocol can be accessed, or state that a protocol was not prepared.	6
	24c	Describe and explain any amendments to information provided at registration or in the protocol.	6

For peer review only

Section and Topic	Item #	Checklist item	Location where item is reported
Support	25	Describe sources of financial or non-financial support for the review, and the role of the funders or sponsors in the review.	17
Competing interests	26	Declare any competing interests of review authors.	17
Availability of data, code and other materials	27	Report which of the following are publicly available and where they can be found: template data collection forms; data extracted from included studies; data used for all analyses; analytic code; any other materials used in the review.	17

From: Page MJ, McKenzie JE, Bossuyt PM, Boutron I, Hoffmann TC, Mulrow CD, et al. The PRISMA 2020 statement: an updated guideline for reporting systematic reviews. BMJ 2021;372:n71. doi: 10.1136/bmj.n71. This work is licensed under CC BY 4.0. To view a copy of this license, visit https://creativecommons.org/licenses/by/4.0/

Synthesis Without Meta-analysis (SWiM) reporting items

The citation for the Synthesis Without Meta-analysis explanation and elaboration article is: Campbell M, McKenzie JE, Sowden A, Katikireddi SV, Brennan SE, Ellis S, Hartmann-Boyce J, Ryan R, Shepperd S, Thomas J, Welch V, Thomson H. Synthesis without meta-analysis (SWiM) in systematic reviews: reporting guideline BMJ 2020;368:l6890 http://dx.doi.org/10.1136/bmj.l6890

**SWiM is intended to complement and be used as an extension to PRISMA**			
**SWiM reporting item**	**Item description**	**Page in manuscript where item is reported**	**Other***
Methods			
**1** Grouping studies for synthesis	1a) Provide a description of, and rationale for, the groups used in the synthesis (e.g., groupings of populations, interventions, outcomes, study design)	8	
	1b) Detail and provide rationale for any changes made subsequent to the protocol in the groups used in the synthesis	N/A (no changes)	
**2** Describe the standardised metric and transformation methods used	Describe the standardised metric for each outcome. Explain why the metric(s) was chosen, and describe any methods used to transform the intervention effects, as reported in the study, to the standardised metric, citing any methodological guidance consulted	8	

For peer review only

**3** Describe the synthesis methods	Describe and justify the methods used to synthesise the effects for each outcome when it was not possible to undertake a meta-analysis of effect estimates	8	
**4** Criteria used to prioritise results for summary and synthesis	Where applicable, provide the criteria used, with supporting justification, to select the particular studies, or a particular study, for the main synthesis or to draw conclusions from the synthesis (e.g., based on study design, risk of bias assessments, directness in relation to the review question)	8	
**SWiM reporting item**	**Item description**	**Page in manuscript where item is reported**	**Other***
**5** Investigation of heterogeneity in reported effects	State the method(s) used to examine heterogeneity in reported effects when it was not possible to undertake a meta-analysis of effect estimates and its extensions to investigate heterogeneity	8	
**6** Certainty of evidence	Describe the methods used to assess certainty of the synthesis findings	8	
**7** Data presentation methods	Describe the graphical and tabular methods used to present the effects (e.g., tables, forest plots, harvest plots).<br>Specify key study characteristics (e.g., study design, risk of bias) used to order the studies, in the text and any tables or graphs, clearly referencing the studies included	8	
Results			
**8** Reporting results	For each comparison and outcome, provide a description of the synthesised findings, and the certainty of the findings. Describe the result in language that is consistent with the question the synthesis addresses, and indicate which studies contribute to the synthesis	10-13	
Discussion			
**9** Limitations of the synthesis	Report the limitations of the synthesis methods used and/or the groupings used in the synthesis, and how these affect the conclusions that can be drawn in relation to the original review question	16	

PRISMA=Preferred Reporting Items for Systematic Reviews and Meta-Analyses.
*If the information is not provided in the systematic review, give details of where this information is available (e.g., protocol, other published papers (provide citation details), or website (provide the URL)).

For peer review only

Supplemental file 2. Completed PRISMA for Abstracts 2020 checklist

Section and Topic	Item #	Checklist item	Reported (Yes/No)
**TITLE**			
Title	1	Identify the report as a systematic review.	Yes
**BACKGROUND**			
Objectives	2	Provide an explicit statement of the main objective(s) or question(s) the review addresses.	Yes
**METHODS**			
Eligibility criteria	3	Specify the inclusion and exclusion criteria for the review.	Yes
Information sources	4	Specify the information sources (e.g. databases, registers) used to identify studies and the date when each was last searched.	Yes
Risk of bias	5	Specify the methods used to assess risk of bias in the included studies.	Yes
Synthesis of results	6	Specify the methods used to present and synthesise results.	Yes
**RESULTS**			
Included studies	7	Give the total number of included studies and participants and summarise relevant characteristics of studies.	Yes
Synthesis of results	8	Present results for main outcomes, preferably indicating the number of included studies and participants for each. If meta-analysis was done, report the summary estimate and confidence/credible interval. If comparing groups, indicate the direction of the effect (i.e. which group is favoured).	Yes
**DISCUSSION**			
Limitations of evidence	9	Provide a brief summary of the limitations of the evidence included in the review (e.g. study risk of bias, inconsistency and imprecision).	Yes
Interpretation	10	Provide a general interpretation of the results and important implications.	Yes
**OTHER**			
Funding	11	Specify the primary source of funding for the review.	Yes
Registration	12	Provide the register name and registration number.	Yes

For peer review only

Supplemental file 3. Detailed search strategies

Medline – via OVID

Search date: Wednesday 16th July 2025

1. exp Multiple Sclerosis/

2. multiple sclerosis.mp.

3. MS.mp.

4. 1 or 2 or 3

5. exp Music Therapy/

6. music therap.mp.*

7. (music-based adj3 therap*).mp.

8. Music-based therapy.mp.

9. music intervention

10. music interventions.mp.

11. musical intervention.mp.

12. musical interventions.mp.

13. rhythmic auditory stimulation.mp.

14. sound therap.mp.*

15. singing therapy.mp.*

16. auditory stimulation.mp.*

17. 5 or 6 or 7 or 8 or 9 or 10 or 11 or 12 or 13 or 14 or 15

18. 4 and 17

19. limit 14 to English language

For peer review only

Results: 198

Search History (19) View Saved

#	Searches	Results	Runtime	Type	Actions	Annotations
1	exp Multiple Sclerosis/	75635	0.62	Advanced	Display Results More	
2	Multiple Sclerosis.mp.	109818	0.98	Advanced	Display Results More	
3	MS.mp.	478942	0.48	Advanced	Display Results More	
4	1 or 2 or 3	531625	0.09	Advanced	Display Results More	
5	exp Music Therapy/	4964	0.09	Advanced	Display Results More	
6	music therap*.mp.	6740	2.61	Advanced	Display Results More	
7	(music-based adj3 therap*).mp. [mp=title, book title, abstract, original title, name of substance word, subject heading word, floating sub-heading word, keyword heading word, organism supplementary concept word, protocol supplementary concept word, rare disease supplementary concept word, unique identifier, synonyms, population supplementary concept word, anatomy supplementary concept word]	103	2.42	Advanced	Display Results More	
8	music-based therapy.mp.	27	0.64	Advanced	Display Results More	
9	music intervention.mp.	700	0.34	Advanced	Display Results More	
10	music interventions.mp.	456	0.32	Advanced	Display Results More	
11	musical intervention.mp.	86	0.28	Advanced	Display Results More	
12	musical interventions.mp.	68	0.27	Advanced	Display Results More	
13	rhythmic auditory stimulation.mp.	215	0.52	Advanced	Display Results More	
14	sound therap*.mp.	458	2.16	Advanced	Display Results More	
15	singing therapy*.mp.	22	1.16	Advanced	Display Results More	
16	auditory stimulation*.mp.	2366	0.63	Advanced	Display Results More	
17	5 or 6 or 7 or 8 or 9 or 10 or 11 or 12 or 13 or 14 or 15 or 16	9799	0.02	Advanced	Display Results More	
18	4 and 17	211	0.05	Advanced	Display Results More	
19	limit 18 to english language	198	0.02	Advanced	Display Results More	

Save Remove Combine with: AND OR Contract

Embase via Ovid

Search date: Wednesday 16th July 2025

1. *exp Multiple Sclerosis/*
2. *multiple sclerosis.mp.*
3. *MS.mp.*
4. *1 or 2 or 3*
5. *exp Music Therapy/*
6. *music therap*.mp.*
7. music-based therap*.mp.

For peer review only - http://bmjopen.bmj.com/site/about/guidelines.xhtml

8. music intervention,mp.
9. music interventions.mp.
10. musical intervention.mp.
11. musical interventions.mp.
12. *rhythmic auditory stimulation.mp.*
13. *sound therap*.mp.*
14. *singing therap*.mp.*
15. *auditory stimulation*.mp.*
16. *5 or 6 or 7 or 8 or 9 or 10 or 11 or 12 or 13 0r 14 or 15*
17. *4 and 16*
18. *limit 17 to English language*

results: 4029

# ▲	Searches	Results	Runtime	Type	Actions		Annotations
1	exp multiple sclerosis/	176930	0.07	Advanced	Display Results	More	
2	multiple sclerosis.mp.	193474	0.77	Advanced	Display Results	More	
3	MS.mp.	664062	0.18	Advanced	Display Results	More	
4	1 or 2 or 3	754065	0.15	Advanced	Display Results	More	
5	exp music therapy/	11246	0.08	Advanced	Display Results	More	
6	music therap*.mp.	11935	4.36	Advanced	Display Results	More	
7	music-based therap*.mp.	61	4.43	Advanced	Display Results	More	
8	music intervention.mp.	1086	0.23	Advanced	Display Results	More	
9	music interventions.mp.	595	0.15	Advanced	Display Results	More	
10	musical intervention.mp.	143	0.22	Advanced	Display Results	More	
11	musical interventions.mp.	98	0.16	Advanced	Display Results	More	
12	rhythmic auditory stimulation.mp.	338	0.65	Advanced	Display Results	More	
13	sound therap*.mp.	625	4.48	Advanced	Display Results	More	
14	singing therap*.mp.	36	4.21	Advanced	Display Results	More	
15	auditory stimulation*.mp.	43773	0.43	Advanced	Display Results	More	
16	5 or 6 or 7 or 8 or 9 or 10 or 11 or 12 or 13 or 14 or 15	56404	0.02	Advanced	Display Results	More	
17	4 and 16	4168	0.07	Advanced	Display Results	More	
18	limit 17 to english language	4029	0.02	Advanced	Display Results	More	

Save Remove Combine with: AND OR Contract

For peer review only

APA PsycInfo via Ovid

Search date: Wednesday 16th July 2025

1. *exp Multiple Sclerosis/*
2. *multiple sclerosis.mp.*
3. *MS.mp.*
4. *1 or 2 or 3*
5. *exp Music Therapy/*
6. *music therap*.mp.*
7. music-based therap*.mp.
8. music intervention.mp.
9. music interventions.mp.
10. musical intervention.mp.
11. musical interventions.mp.
12. *rhythmic auditory stimulation.mp.*
13. *sound therap*.mp.*
14. *singing therap*.mp.*
15. *auditory stimulation*.mp.*
16. *5 or 6 or 7 or 8 or 9 or 10 or 11 or 12 or 13 0r 14 or 15*
17. *4 and 16*
18. *limit 17 to English language*

results: 1155

For peer review only

# ▲	Searches	Results	Runtime	Type	Actions		Annotations
1	exp Multiple Sclerosis/	15247	0.14	Advanced	Display Results	More	
2	Multiple Sclerosis.mp.	19196	1.06	Advanced	Display Results	More	
3	MS.mp.	37720	0.20	Advanced	Display Results	More	
4	1 or 2 or 3	44700	0.01	Advanced	Display Results	More	
5	exp Music Therapy/	6337	0.13	Advanced	Display Results	More	
6	Music therap*.mp.	7695	0.57	Advanced	Display Results	More	
7	music-based therap*.mp.	27	1.03	Advanced	Display Results	More	
8	music intervention.mp.	353	0.29	Advanced	Display Results	More	
9	music interventions.mp.	304	0.24	Advanced	Display Results	More	
10	musical intervention.mp.	59	0.29	Advanced	Display Results	More	
11	musical interventions.mp.	76	0.23	Advanced	Display Results	More	
12	rhythmic auditory stimulation.mp.	83	0.58	Advanced	Display Results	More	
13	sound therap*.mp.	167	0.68	Advanced	Display Results	More	
14	singing therap*.mp.	13	0.73	Advanced	Display Results	More	
15	auditory stimulation*.mp.	24980	0.60	Advanced	Display Results	More	
16	5 or 6 or 7 or 8 or 9 or 10 or 11 or 12 or 13 or 14 or 15	32845	0.01	Advanced	Display Results	More	
17	4 and 16	1175	0.01	Advanced	Display Results	More	
18	limit 17 to english language	1155	0.13	Advanced	Display Results	More	

Save Remove Combine with: AND OR Contract

AMED via EBSCOhost

Search date: Wednesday 16th July 2025

"multiple sclerosis" OR "MS"

AND

"music therap*" OR "music-based therap*" OR "musical intervention" OR "musical interventions" OR “music intervention” OR “music interventions” OR "rhythmic auditory stimulation*" OR "sound therap*" OR "singing therap*" OR "auditory stimulation*"

Limit = English language

Results: 17

For peer review only

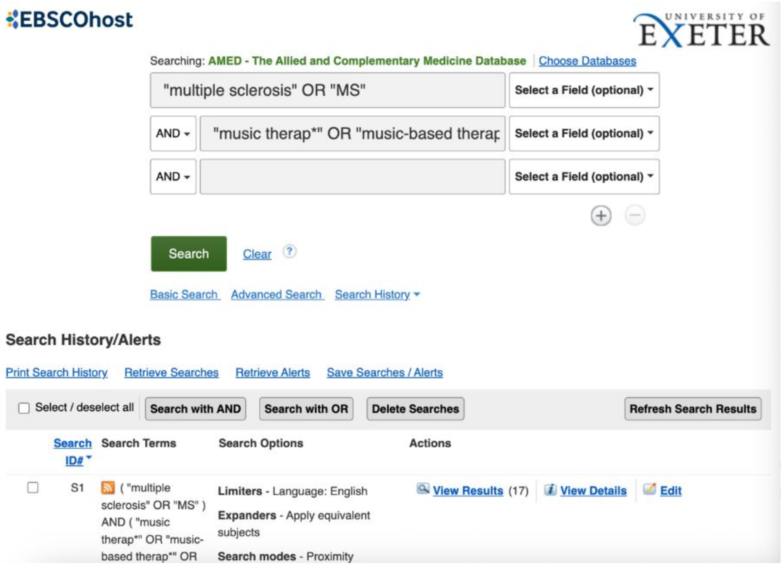

er review only

CINAHL Ultimate via EBSCOhost

Search date: Wednesday 16th July 2025

"multiple sclerosis" OR "MS"

AND

"music therap*" OR "music-based therap*" OR "musical intervention" OR "musical interventions" OR “music intervention” OR “music interventions” OR "rhythmic auditory stimulation*" OR "sound therap*" OR "singing therap*" OR "auditory stimulation*"

Limit = English language

Results: 107

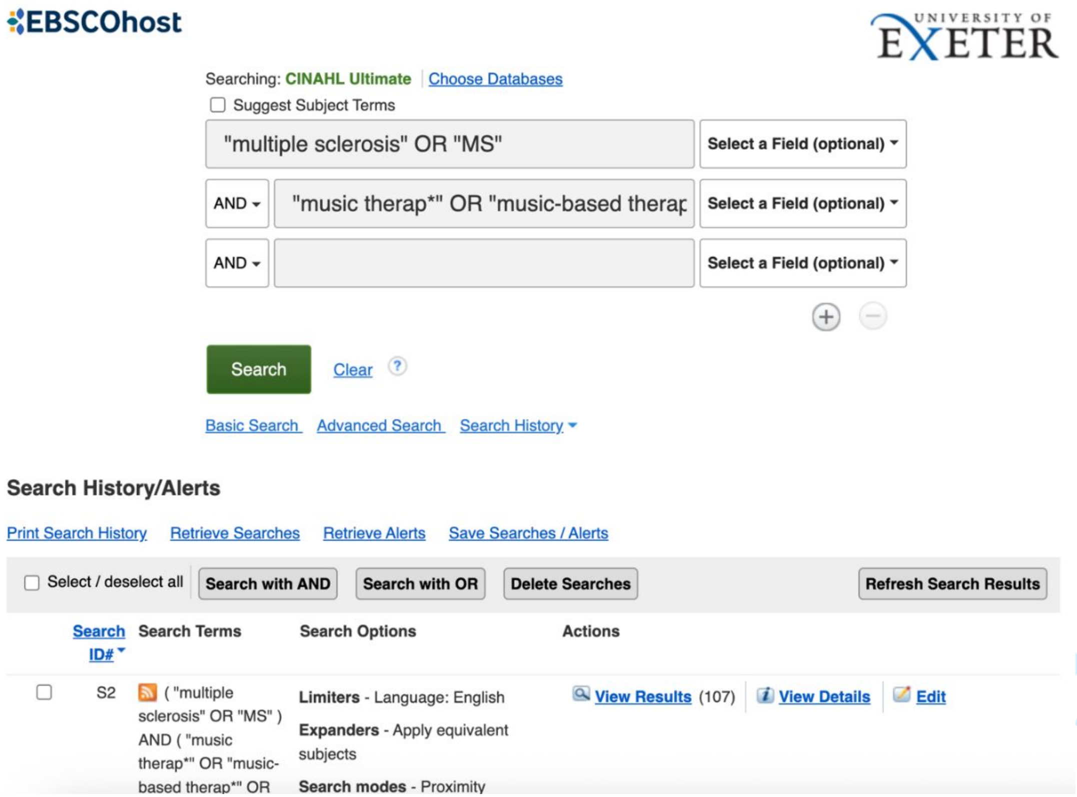

COCHRANE-Central

Search date: Wednesday 16th July 2025

"multiple sclerosis" OR "MS"

AND

(music therapy) OR (music-based therapy) OR (musical intervention) OR (musical interventions) OR (music intervention) OR (music interventions) OR (rhythmic auditory stimulation) OR (sound therapy) OR (singing therapy) OR (auditory stimulation)

Results: 400 trials (Trials = 392 are English and 8 non-English)

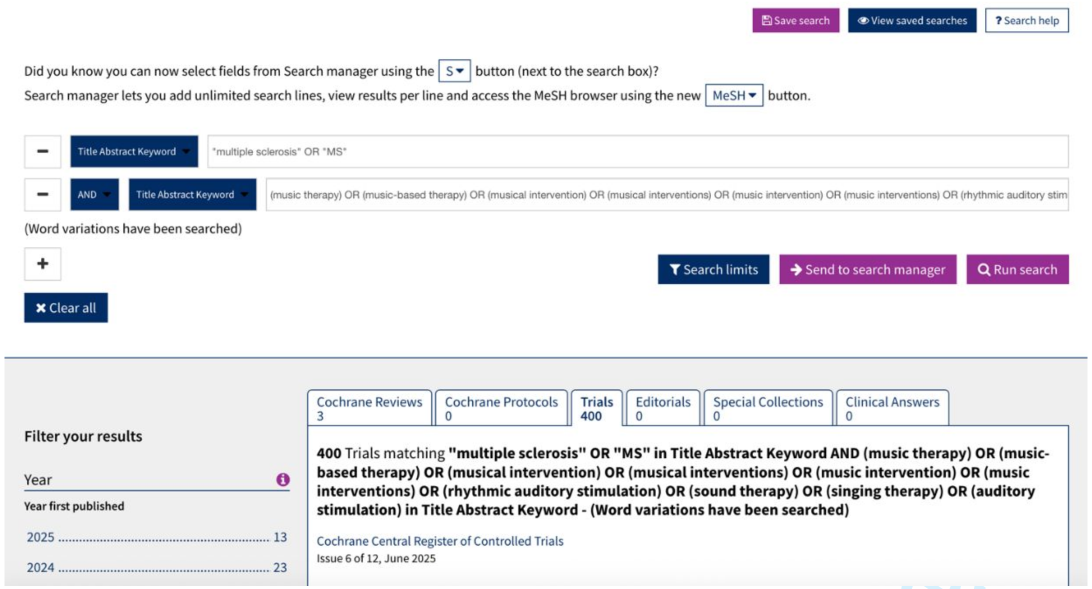
Save search
View saved searches
? Search help
Did you know you can now select fields from Search manager using the S button (next to the search box)?
Search manager lets you add unlimited search lines, view results per line and access the MeSH browser using the new MeSH button.
Title Abstract Keyword
"multiple sclerosis" OR "MS"
AND
Title Abstract Keyword
(music therapy) OR (music-based therapy) OR (musical intervention) OR (musical interventions) OR (music intervention) OR (music interventions) OR (rhythmic auditory stim
(Word variations have been searched)
Search limits
Send to search manager
Run search
Clear all
Filter your results
Year
Year first published
2025 13
2024 23
Cochrane Reviews 3
Cochrane Protocols 0
Trials 400
Editorials 0
Special Collections 0
Clinical Answers 0
400 Trials matching "multiple sclerosis" OR "MS" in Title Abstract Keyword AND (music therapy) OR (music-based therapy) OR (musical intervention) OR (musical interventions) OR (music intervention) OR (music interventions) OR (rhythmic auditory stimulation) OR (sound therapy) OR (singing therapy) OR (auditory stimulation) in Title Abstract Keyword - (Word variations have been searched)
Cochrane Central Register of Controlled Trials
Issue 6 of 12, June 2025

review only

Supplemental file 4. Full list of included publications

1. Aldridge D, Schmid W, Kaeder M, et al. Functionality or aesthetics? A pilot study of music therapy in the treatment of multiple sclerosis patients. *Complement Ther Med* 2005; 13(1): 25–33.
2. Conklyn D, Stough D, Novak E, et al. A home-based walking program using rhythmic auditory stimulation improves gait performance in patients with multiple sclerosis: a pilot study. Neurorehabil Neural Repair 2010; 24(9): 835-42.
3. Gatti R, Tettamanti A, Lambiase S, et al. Improving hand functional use in subjects with multiple sclerosis using a musical keyboard: a randomized controlled trial. Physiother Res Int 2015; 20: 100-7.
4. Goetschalckx M, Van Geel F, Meesen R, et al. Rhythmic interlimb coordination of the lower limbs in multiple sclerosis during auditory pacing to three different frequencies. Gait Posture 2021; 334-40.
5. Helminger B, Seebacher B, Ropele S, et al. Effects of rhythmic-cued gait training on gait-like task related brain activation in people with multiple sclerosis. J Neurol Sci 2025; 471: 123426.
6. Impellizzeri F, Leonardi S, Latella D, et al. An integrative cognitive rehabilitation using neurologic music therapy in multiple sclerosis: a pilot study. Medicine 2020: 99(4): e18866.
7. Kuhlmann J, Ebner K, Zimmer A, et al. Music therapy with a monochord in multiple sclerosis (“MUTIMS”): A randomized, controlled, rater-blinded trial. Mult Scler J Exp Transl Clin 2025; 11(3):20552173251352712.
8. Maggio MG, Tripoli D, Porcari B, et al. How may patients with MS benefit from using music assisted therapy? A case-control feasability study investigating motor outcomes and beyond. Mult Scler Relat Disord 2021; 48: 102713.
9. Moore KS, Peterson DA, O’Shea G, et al. The effectiveness of music as a mnemonic device on recognition memory for people with multiple sclerosis. J Music Ther 2008; 45(3): 307-29.
10. Moumdjian L, Moens B, Maes PJ, et al. Continuous 12 min walking to music, metronomes and in silence: auditory-motor coupling and its effects on perceived fatigue, motivation and gait in persons with multiple sclerosis. Mult Scler Relat Disord 2019; 35: 92-9.
11. Naderi S, Sadeghi H, Amirseyfaddini M. The effects of rhythmic auditory stimulation with different tempos on spatio-temporal parameters and gait symmetry in patients with multiple sclerosis: a quasi-experimental study. J Res Rehabil Sci 2022; 18: doi: 10.48305/jrrs.2023.41771.1041.
12. Schmid W, Aldridge D. Active music therapy in the treatment of multiple sclerosis patients: a matched control study. J Music Ther 2004; 41(3): 225-40.
13. Seebacher B, Helmlinger B, Pinter D, et al. Actual and imagined music-cued gait training in people with multiple sclerosis: a double-blind randomized parallel multicenter trial. Neurorehabil Neural Repair 2024; 38(8): 555-69.
14. Seebacher B, Kuisma R, Glynn A, et al. Effects and mechanisms of differently cued and non-cued motor imagery in people with multiple sclerosis: a randomised controlled trial. Mult Scler J 2019; 25(12): 1593-1604.
15. Seebacher B, Kuisma R, Glynn A, et al. Exploring cued and non-cued motor imagery interventions in people with multiple sclerosis: a randomised feasibility trial and reliability study. Arch Physiother 2018; 8:6.
16. Seebacher B, Kuisma R, Glynn A, et al. The effect of rhythmic-cued motor imagery on walking, fatigue and quality of life in people with multiple sclerosis: a randomised controlled trial. Mult Scler J 2017; 23(2): 286-96.
17. Seebacher B, Kuisma R, Glynn A, et al. Rhythmic cued motor imagery and walking in people with multiple sclerosis: a randomised controlled feasibility study.
Pilot Feasibility Stud 2015; 1: 25.
18. Seighalani MZ, Ghahari S, Zarbakhsh M. The effectiveness of music therapy on depression, anxiety and self-esteem of patients with multiple sclerosis (MS). Adv Environ Biol 2014; 8(9): 253-60.

For peer review only

19. Shahraki M, Sohrabi M, Taheri Torbati HR, et al. Effect of rhythmic auditory stimulation on gait kinematic parameters of patients with multiple sclerosis. J Med Life 2017; 10(1): 33-7.
20. Thaut MH, Peterson DA, Sena KM, et al. Musical structure facilitates verbal learning in multiple sclerosis. *Music Percept* 2008; 25(4): 325–30.
21. Thaut MH, Peterson DA, McIntosh GC, et al. Music mnemonics aid verbal memory and induce learning – related brain plasticity in multiple sclerosis. Front Hum Neurosci 2014; 8: 395.
22. Wiens ME, Reimer MA, Guyn HL. Music therapy as a treatment method for improving respiratory muscle strength in patients with advanced multiple sclerosis: a pilot study. Rehabil Nurs 1999; 24(2): 74-80.
23. Young HJ, Mehta TS, Herman C, et al. The effects of M2M and adapted yoga on physical and psychosocial outcomes in people with multiple sclerosis. Arch Phys Med Rehabil 2019;100(3):391-400.

List of studies for each outcome domain (studies can contribute to multiple outcome domains)

<u>Motor</u>

1. Conklyn et al. 2010
2. Gatti et al. 2015
3. Goetschalckx. et al. 2021
4. Helminger. et al. 2025
5. Maggio et al. 2021
6. Moumdjian et al. 2019
7. Naderi et al 2022
8. Schmid et al, 2004
9. Seebacher et al. 2015
10. Seebacher et al. 2017
11. Seebacher et al. 2018
12. Seebacher et al. 2019
13. Seebacher et al. 2024
14. Shahraki et al, 2017
15. Wiens et al. 1999
16. Young et al. 2019

<u>Cognitive</u>

1. Aldridge et al. 2005
2. Impellizzeri et al. 2020
3. Moore et al. 2008
4. Moumdjian et al. 2019
5. Schmid et al, 2004
6. Seebacher et al. 2024
7. Thaut et al 2008

For peer review only

8. Thaut et al 2014

Psychological

1. Aldridge et al. 2005
2. Impellizzeri et al. 2020
3. Kuhlmann et al. 2025
4. Maggio et al. 2021
5. Schmid et al. 2004
6. Seebacher et al. 2024
7. Seighalani et al. 2014

Quality of life

1. Aldridge et al. 2005
2. Concklyn et al 2010
3. Impellizzeri et al. 2020
4. Kuhlmann et al. 2025
5. Maggio et al. 2021
6. Schmid et al. 2004
7. Seebacher et al. 2015
8. Seebacher et al. 2017
9. Seebacher et al. 2018
10. Seebacher et al. 2019
11. Seebacher et al. 2024
12. Young et al. 2019

For peer review only

Supplemental file 5. Full list of studies excluded at full text review with reasons

Duplicate

1. Lambiase S, Nava C, Tettamanti A, et al. Improving hand functional use in subjects with multiple sclerosis using a musical keyboard: a randomized controlled trial. Physiother Res Int 2015;20: 100-7. [This is Gatti et al, 2015, included study].
2. Moore KS, Peterson DA, O'Shea G, et al. The effectiveness of music as a mnemonic device on recognition memory for people with multiple sclerosis. *J Music Ther* 2008; 45(3): 307–29.

Intervention

3. Baram Y, Miller A. Auditory feedback control for improvement of gait in patients with multiple sclerosis. J Neurol Sci 2007; 254: 90-4.
4. Basturk S, Ekici G, Kirteke F, et al. Therapeutic effects of line dancing in people with multiple sclerosis: an evaluator-blinded, randomized controlled study. Art Health 2024; doi: 10.1080/17533015.2024.2325425.
5. Mandelbaum R, Triche EW, Fasoli SE, et al. A pilot study: examining the effects and tolerability of structured dance intervention for individuals with multiple sclerosis. *Disabil Rehabil* 2016; 38(3): 218–22
6. Trinchillo A, Caliendo D, Nicolella V, et al. Tango classes in people with Multiple Sclerosis (PwMS): Impact on motor and non-motor functions. Clin Neurol Neurosurg 2024; 243: 108380.
7. Van Geel F, Van Asch P, Veldkamp R, et al. Effects of a 10-week multimodal dance and art intervention program leading to a public performance in persons with multiple sclerosis: a controlled pilot-trial. Mult Scler Relat Disord 2020; 44: 102256.

Outcome

8. Fasching B, Mildner S, Fink F, et al. MuSic Moves-co-creating a music-supported exercise programme with and for people with multiple sclerosis: a bicentre participatory mixed methods study. BMJ Open 2024; 14(11): e091168.
9. Moumdjian L, Maes PJ, Dalla Bella S, et al. Detrended fluctuation analysis of gait dynamics when entraining to music and metronomes at different tempi in persons with multiple sclerosis. Sci Rep 2020; 10: 12934.
10. Moumdjian L, Moens B, Maes PJ, et al. Walking to music and metronome at various tempi in persons with multiple sclerosis: a basis for rehabilitation. Neurorehabil Neural Repair 2019; 33(6): 464-75.
11. Seebacher B, Helmlinger B, Hotz I, et al. Actual and imagined music-cued gait training for people with multiple sclerosis: a multicentre qualitative study. BMJ Open 2024; 14: e086555.
12. Seighalani MZ, Cheraghali Gol H, Rostami AM. The effectiveness of music therapy on depression, anxiety and self-esteem of patients with multiple sclerosis. *Adv Environ Biol* 2014; 8(10): 759–64.
13. Vanbilsen N, Moumdjian L, Kinnaert F, et al. Step to the beat: Auditory-motor coupling during walking to higher and lower tempi with music and metronomes in progressive multiple sclerosis: an observational study. Mult Scler Relat Disord 2024; 92: 106152.

Population

14. Srinithiwat P, Foocharoen C, Mahakkanukrauh A, et al. Patient perspectives and satisfaction following a short-term singing intervention in systemic sclerosis patients: a cross-sectional study. *J Med Assoc Thai* 2025; 108: S59–65.

For peer review only

15. Young HJ, Lai B, Wilroy J, et al. Effects of a teleexercise movement-to-music intervention on health outcomes in individuals with mobility disabilities: a randomized controlled trial. Arch Phy Med Rehabil 2025; https://doi.org/10.1016/j.apmr.2025.05.017.

Publication type

16. https://clinicaltrials.gov/study/NCT05792176. Ukulele playing to improve cognition in people with multiple sclerosis: a feasibility study [No results posted].
17. https://clinicaltrials.gov/study/NCT04019912. The novel use of treadmill plus music in MS patients gate rehabilitation (SMUSIC) [No results posted – spelling mistake ‘gate’ for ‘gait’ was made on Clinical Trials.gov].
18. https://clinicaltrials.gov/study/NCT04314076. Rhythmic auditory stimulation & gait training [No results posted].
19. https://clinicaltrials.gov/study/NCT01086371. Rhythmic auditory stimulation and walking performance in multiple sclerosis (MS) patients [No results posted].
20. https://clinicaltrials.gov/study/NCT06800144. Effect of music therapy on pain in people with multiple sclerosis [No results posted].
21. https://trialsearch.who.int/Trial2.aspx?TrialID=IRCT20230219057455N1. The effectiveness of slow and fast music on anxiety and quality of life of people with multiple sclerosis [No results posted].
22. https://trialsearch.who.int/Trial2.aspx?TrialID=DRKS00020469. Effectiveness of playing the instruments „Big Bom“and “Temple Blocks” as a music therapy approach to improve gait function among patients with multiple sclerosis [No results posted].
23. Bethoux F, Awad L, Gallagher L, et al. Safety and acceptability of closed-loop rhythmic cueing for gait training in persons with multiple sclerosis: a pilot single-blind randomized controlled trial. Mult Scler J 2023: 29(3): 645 [Abstract].
24. Pedulla L, Addeo S, Rinaldi S, Aerobic training in multiple sclerosis: a pilot study on the effects of music during group treatment. Mult Scler J 2020; *26* (Suppl 2): 54-55 [Abstract].

For peer review only

Supplemental file 6. Study characteristics for included studies.

Author, year	Country	Design	Participants	Inclusion criteria	Exclusion criteria	Outcomes
Aldridge et al. 2005	Germany	Matched controlled pilot study	20 people with MS (10 per arm). 30% male Age range: 29-47	Diagnosis of multiple sclerosis and within the EDSS range used for matching	Pregnancy and mental disorders requiring medication	Primary: Psychological outcomes such as Depression (Beck, HADS), Anxiety (HADS), Self-acceptance (SESA) Secondary: Quality of life (HAQUAMS) Functional disability (EDSS) Cognitive (MSFC) and motor function (MSFC) Fatigue (IFSS)
Conklyn et al, 2010	USA	RCT	10 people with MS (5 per arm), 30% male, Mean age: intervention 47, control 50	Age 18 or over; medical diagnosis of MS; able to walk 30.48m without physical assistance (assistive devices were allowed); able to walk 7.62m in 8 to 60s [distances were published in imperial feet]	Treatment for MS exacerbation in past 30 days; severe comorbidity precluding participation; severe cognitive deficits precluding informed consent or preventing following study instructions	Primary: Gait parameters<br><br>Secondary: Spasticity (MAS Modified Ashworth Scale); muscle strength (Manual Muscle Testing); Pain (out of 10); Ambulation disability (Ambulation Index); MS-related disability

For peer review only

						(Patient-determined Disease Steps); Treatment efficacy (Subject Global Impression, Clinician Global Impression)
Gatti et al, 2015	Italy	RCT	19 people with MS (9 intervention, 10 control), 37% male, mean age intervention 43, control 48	Adults; primary or secondary progressive MS; no relapse or medication changes in past 3 months; hand strength below normal; Nine-Hole Peg Test >18s; no experience playing a musical instrument; no cognitive or psychiatric disorders; no hearing or visual deficits	No additional criteria	Primary: Perceived hand functional use (ABILHAND)<br><br>Secondary: Hand dexterity (Nine-Hole Peg Test); Hand strength (Jamar and Pinch)
Goetschalckx et al, 2021	Belgium	Observational cross-sectional comparative study	38 people with MS (16% male, mean age 49).13 healthy controls (38% male, mean age 52). Note that the 'controls' were not a comparator group in terms of intervention – they received the same MBTs, the only difference was they did not have MS	MS diagnosis (McDonald criteria); age 18-70; able to walk independently	Other medical conditions influencing walking	Primary: Bilateral gait coordination (Phase Coordination Index)<br><br>Secondary: movement amplitude; movement frequency

For peer review only

Helminger et al, 2025	Austria	Secondary analysis of a randomised controlled trial (longitudinal interventional study)	48 people with MS (52% male, mean age 44). 17 health controls (53% male, mean age 41)	Age at least 18; definite MS (Thompson criteria); mild-to-moderate disability (Expanded Disability Status Scale 2-5);	Relapse or change in disease-modifying treatment or physiotherapy in past 3 months; concomitant diseases; MRI contraindications	Primary: Walking function (2-minute walk test; timed 25-foot walk)<br><br>Secondary: fMRI brain activation changes during movement tasks; correlation of brain activation changes with walking performance
Impellizzeri et al, 2020	Italy	RCT	30 people with MS (15 per arm), 63% male, mean age intervention 52, control 51	MS diagnosis (Lublin criteria); Expanded Disability Status Scale 3-7; enjoy music; no visual or hearing loss; no severe medical and psychiatric illness	No additional criteria	Cognition (Brief Repeatability Battery of Neurophysiology); quality of life (MSQoL-54); depression (Beck); emotions (Emotional Awareness Questionnaire)
Kuhlmann et al, 2025	Switzerland	RCT	57 people with MS (30 intervention, 27 control), 18% male, mean age 50	Aged at least 18; MS diagnosis (McDonald criteria); Expanded Disability Status Scale no more than 6.5	Severe psychiatric disorders; other life-threatening or severely disabling physical disorders; >2 MS relapses in past year or relapse in past 3 months; change in disease-modifying treatment or symptomatic MS medication in past 3	Primary: Anxiety (HADS)<br><br>Secondary: Depression (HADS); fatigue (self-report); pain (quantitative sensory testing); body perception (self-report)

For peer review only

					months; pregnancy; unable to lie supine for 15 min; severely compromised hearing (uncorrected); numbness on the back of both hands	
Maggio et al, 2021	Italy	Quasi-experimental controlled feasibility study	20 people with MS (10 per arm), 35% male	Secondary progressive MS (Lublin criteria); Expanded Disability Status Scale 3-6; enjoy music; no hearing or visual loss; no change in disease-modifying drugs in past 6 months	Age <65; other severe medical conditions or psychiatric illnesses; history of neuroplasms or other neurological conditions; moderate-severe cognitive decline (MoCA <21); neurologic music therapy in past 3 months	Motor evaluation (Berg Balance; Timed Up and Go; 10-metre walk test); neuropsychological battery (Beck depression; MSQOL-54 quality of life; Goal Attainment Scaling); usability (System Usability Scale)
Moore et al, 2008	USA	RCT	38 people with MS (20 intervention, 18 control), 21% male, mean age 53	Right-handed; non-smoker; Extended Disability Status Scale 3.5-7; at least 5 MS brain lesions on MRI; stable on immunomodulatory therapy; less than 2 exacerbations in past 12 months	Active exacerbation phase; treatment with pulse-cortical steroids in past 3 months; history of seizure disorder, traumatic brain injury or substance abuse; taking cognition-enhancing acetylecho-linesterase inhibitors	Verbal learning and memory (adapted Auditory-Visual Learning Test) – sensitivity index; response bias index

For peer review only

Moumdjian et al, 2019	Belgium	Case-control study	31 people with MS (26% male, mean age 53). 30 healthy controls (27% male, mean age 52). Note that only 27 people with MS and 30 healthy controls completed the experimental session. Note that the 'controls' were not a comparator group in terms of intervention – they received the same MBTs, the only difference was they did not have MS	Aged at least 18; diagnosis of MS for over 1 year; no exacerbation in past month; average comfortable walking speed 0.4-1.2 m/s	Pregnancy; hearing impairment; cognitive impairment hindering understanding of instructions	Primary: Auditory-motor coupling and synchronisation<br><br>Secondary: Perceived physical and cognitive fatigue (Modified Fatigue Impact Scale); motivation (Likert); cadence; stride length; velocity
Naderi et al, 2022	Iran	Quasi-experimental controlled feasibility study	13 people with MS (0% male, mean age 38). 14 healthy controls (0% male, mean age 38). Note that the 'controls' were not a comparator group in terms of intervention – they received the same MBTs, the only difference was they did not have MS	MS confirmed by neurologist; Expanded Disability Status Scale 3.5-5.5; no vision or hearing problems; no other active treatment; able to walk without assistive devices	Unable to perform tasks; severe attacks or relapse on day of the test	Primary: Gait (Gait Symmetry Index) – step duration symmetry; stride length symmetry<br><br>Secondary: Additional gait parameters
Schmid et al, 2004	USA	Matched control study	20 people with MS (10 per arm), 30%	Not specifically stated (only exclusion criteria)	Pregnancy; mental disorders requiring medication; previous	Depression (Beck); anxiety (HADS); self-acceptance (Social

For peer review only

			male, age range 29-47		experience of music therapy	and Emotional Skills Assessment); quality of life (Hamburg Quality of Life Questionnaire in MS); cognitive and functional disability (Expanded Disability Status Scale)
Seebacher et al, 2024	Austria	RCT	127 people with MS (split 43, 44, 40 across the 3 music therapy arms), 25% male, mean age per arm range 47-50	Age 18 or over; MS diagnosis (McDonald criteria); Expanded Disability Status Scale 2-5; stable disease	Cognitive impairment (MoCA <26), anxiety or depression (HADS subscales 11 or over); suicidality; relapse, starting new medication, medication adjustment or physiotherapy in past 3 months or during the study	Primary: Walking speed (timed 25-foot walk); change in walking distance (2-minute walk test)<br><br>Secondary: Cognition (MoCA); anxiety and depression (HADS); suicidality (narrative screening); fatigue (Neurological Fatigue Index-MS); quality of life (MS International Quality of Life); motor imagery (Kinesthetic and Visual Imagery Questionnaire-10)
Seebacher et al, 2019	Austria	RCT	59 people with MS (split 19, 20, 20 across the 3 music therapy arms), 20% male, mean age 45	Aged 18 or over; German speaking; clinically definite MS (McDonald criteria); mild-to-moderate disability (Expanded Disability Status Scale 1.5-4.5)	Concomitant diseases affecting interventions or walking; relapse in past 3 months or during the study; change of treatment in past 2 months or	Primary: Walking speed (timed 25-foot walk); change in walking distance (2-minute walk test)<br>Secondary: Fatigue (Modified Fatigue Impact Scale); quality

For peer review only

					during the study; pregnancy; clinical symptoms of depression or cognitive dysfunction	of life (MS Impact Scale-29); motor imagery (Kinesthetic and Visual Imagery Questionnaire-10); sensory motor synchronisation
Seebacher et al, 2018	Austria	Feasibility RCT	15 people with MS (split 5, 5, 5 across the 3 music arms), 13% male, mean age 52	Age 18 or over; MS (McDonald criteria); mild-to-moderate disability (Expanded Disability Status Scale 1.5-4.5); German speaking	Concomitant diseases affecting rhythmic cued motor imagery or walking; relapse in past 3 months or during the study; change of medication or start of physiotherapy in past 2 months; pregnancy; overt symptoms of depression or cognitive dysfunction	Primary: Feasibility; safety and adverse events<br><br>Secondary: Walking speed (timed 25-foot walk); walking distance (6-minute walk test); fatigue (Modified Fatigue Impact Scale); quality of life (MS Impact Scale-29); motor imagery (Kinesthetic and Visual Imagery Questionnaire-10)
Seebacher et al, 2017	Austria and UK	RCT	101 people with MS (34 for each of the 2 music arms, 33 controls), 16% male, mean age 44	Aged 18 or over; clinically definite MS (McDonald criteria); mild-to-moderate disease (Expanded Disability Status Scale 1.5-4.5)	Concomitant diseases affecting interventions or walking; relapse in past 3 months; change of medication or physiotherapy in past 2 months; pregnancy; overt symptoms of depression or cognitive dysfunction	Primary: Walking speed (timed 25-foot walk); change in walking distance (2-minute walk test)<br><br>Secondary: Walking perception (MS Walking Scale-12); fatigue (Modified Fatigue Impact Scale); quality of life

						(MS Impact Scale-29)
Seebacher et al, 2015	Austria	Feasibility RCT	30 people with MS (10 for each of the 2 music arms, 10 controls), 27% male, mean age per arm ranged 42-47	Aged 18 or over; diagnosis of MS (McDonald criteria); mild-to-moderate disability (Expanded Disability Status Scale 1.5-4.5); German speaking	Concomitant diseases affecting rhythmic cued motor imagery or walking; relapse in past 3 months or during the study; medication change in past 2 months; pregnancy; overt symptoms of depression or cognitive dysfunction	Primary: Feasibility; fatigue (Modified Fatigue Impact Scale)<br><br>Secondary: Walking speed (timed 25-foot walk); change in walking distance (2-minute walk test)
Seighalani et al, 2014	Iran	RCT	30 people with MS (15 per arm), gender and age not reported	MS diagnosis by physician; self-esteem below average (Cooper Smith <25)	Cooper Smith self-esteem 25 or above	Depression (Beck); anxiety (Beck); self-esteem (Cooper Smith)
Shahraki et al, 2017	Iran	RCT	18 people with MS (9 per arm), 22% male, mean age intervention 40, control 38	Age 18 or over; Expanded Disability Status Scale 3-6; able to walk 30.48m without assistance; lack of treatment for relapse or MS exacerbation in past 30 days; lack of cardiovascular and rheumatic diseases; lack of severe pain in lower joints; no engagement in regular physical	No additional criteria	Gait parameters

For peer review only

				activity in past 3 months; no hearing impairment		
Thaut et al, 2008	USA	RCT	20 people with MS (10 per arm), 10% male, mean age: intervention 51.7, control 53.8	Right-handed; relapse-remitting MS; normal hearing; no history of other neurological or psychiatric conditions	No additional criteria	Verbal learning and word order memory Recall of higher order word sequences (3–7 words)
Thaut et al, 2014	Germany and USA	RCT	54 people with MS (27 intervention, 27 control), 30% male, mean age intervention 50, control 53	Right-handed; relapse-remitting MS; normal hearing; no history of other neurological or psychiatric conditions; at least 5 brain lesions on MRI; stable on immunomodulatory therapy; <2 exacerbations in past 12 months	In active exacerbation phase; treated with pulse-cortical steroids or cognition-enhancing AChE inhibitors	Learning; memory; brain activity with oscillatory network synchronisation
Wiens et al, 1999	Canada	RCT	19 people with MS (9 intervention, 10 control), gender not reported, mean age 55	Residents in a long-term care centre with MS; able to tolerate 30 minutes of concentrated effort; able to follow verbal instructions; MMSE 20 or above; free from respiratory infection; able to sit in a wheelchair for at least 2 hours	No additional criteria	Strength of respiratory muscles (mouth pressure testing) – inspiratory and expiratory

Young et al, 2019	USA	RCT	81 people with MS (53 intervention across 2 arms, 28 control), 19% male, mean age per arm ranged 47-50	Aged 18-65; self-reported MS diagnosis; Patient-Determined Disease Steps 0-6; able to exercise with arms and/or legs; physician clearance	Participation in similar intervention in past 6 months; use of tobacco in past 6 months; unstable weight; cognitive impairment (MMSE <24); active pressure ulcer; contraindications to exercise (American College of Sports Medicine guidelines)	Primary: Motor evaluation (Timed Up and Go; 6-minute walk test; 5-times sit-to-stand)<br><br>Secondary: Fatigue (Patient-Reported Outcomes Measurement Information System Fatigue); pain (Pain Interference Short Form 8a.)

For peer review only

Supplemental file 7. Intervention profile for included studies.

Author, year	Content	Leader	Location	Duration
Aldridge et al. 2005	Nordoff Robbins approach: Active music therapy - Music-making on instruments, or singing	Therapist	Health facility	3 blocks of music therapy in single sessions over the course of the project (8—10 sessions, respectively)
Conklyn et al, 2010	Rhythmic auditory stimulation	Self	Home	2 sessions of 20 min/day for 4 weeks
Gatti et al, 2015	Playing keyboard (numbered, coloured stickers to guide)	Physiotherapist	Health facility	30 minutes a day, 5 days a week for 3 weeks
Goetschalckx et al, 2021	Rhythmic auditory stimulation with metronome	Facilitator	Health facility	1 single session (each metronome condition lasted 1 minute with a 1-minute rest in between conditions)
Helminger et al, 2025	Actual and/or imagined gait training with rhythmic-auditory cueing	Self	Home	4 sessions of 30 minutes per week for 4 weeks
Impellizzeri et al, 2020	Neurologic music therapy (plus conventional cognitive rehabilitation)	Music therapist	Health facility	3 sessions of 60 minutes per week for 8 weeks
Kuhlmann et al, 2025	Listening to a monochord being played while lying on top of it and feeling the vibration	Music therapist	Health facility	1 session of 45 minutes per week for 6 weeks
Maggio et al, 2021	Listening to music while walking on a treadmill	Physiotherapist	Health facility	3 sessions of 30 minutes per week for 8 weeks
Moore et al, 2008	Hearing word lists in song format and being asked to sing them back	Research team	Health facility	Single session (testing time approximately 60 minutes)

For peer review only

Moumdjian et al, 2019	Rhythmic auditory stimulation (2 groups: music and metronome)	Research team	Health facility	Single session of 12 minutes
Naderi et al, 2022	Rhythmic auditory stimulation	Facilitator	Health facility	Single session (4 walking modes, each 2 minutes walking with 30s rhythm exposure beforehand, and 3-minute rest between modes)
Schmid et al, 2004	Active music therapy (singing or instruments)	Music therapist	Health facility	3 blocks of 8-10 sessions over 1 year
Seebacher et al, 2024	3 groups: cued motor imagery; combined cued motor imagery and cued gait training; cued gait training	Physiotherapist, occupational therapist, psychologist	Home	4 sessions of 30 minutes (including short breaks) per week for 4 weeks
Seebacher et al, 2019	3 groups: music and verbally cued motor imagery; music cued motor imagery; non-cued motor imagery	Self	Home	6 sessions of 17 minutes per week for 4 weeks
Seebacher et al, 2018	3 groups: music and verbally cued motor imagery; music cued motor imagery; non-cued motor imagery	Self	Home	6 sessions of 17 minutes per week for 4 weeks
Seebacher et al, 2017	2 groups: music cued motor imagery; metronome cued motor imagery	Self	Home	6 sessions of 17 minutes per week for 4 weeks
Seebacher et al, 2015	2 groups: music cued motor imagery; metronome cued motor imagery	Self	Home	6 sessions of 17 minutes per week for 4 weeks
Seighalani et al, 2014	Active music therapy (discussion about music; making music with mouth; expressing the effects of music) and passive music therapy (listening to music)	Researcher and music therapist	Health facility	15 sessions of 30 minutes in total (6 active and 9 passive)

For peer review only

Shahraki et al, 2017	Rhythmic auditory stimulation with metronome	Coach	Health facility	3 sessions of 30 minutes per week for 3 weeks
Thaut et al, 2008	Ray's Auditory Verbal Learning Test – sung word lists	Research team	Health facility	Single session
Thaut et al, 2014	Ray's Auditory Verbal Learning Test – sung word lists	Research team	Health facility	Single session
Wiens et al, 1999	Music therapy: focusing on singing: relaxation and diaphragmatic breathing, intonation of syllables, and reading or singing phrases, paragraphs, and simple songs	Music therapist with instrument speciality in voice	Health facility	3 sessions of 30 minutes per week for 12 weeks
Young et al, 2019	2 groups: music to movement; adapted yoga. The yoga group does not meet the inclusion criteria as an intervention for this review and is not presented as a comparator.	Self	Fitness facility	3 sessions of 60 minutes per week for 12 weeks

For peer review only

Supplemental file 8. Control profile for included studies.

**Author, year**	**Synopsis of control arm**
Aldridge et al. 2005	No Intervention
Conklyn et al, 2010	No intervention
Gatti et al, 2015	Playing the keyboard with it turned off
Goetschalckx et al, 2021	Healthy control group who had the same intervention
Helminger et al, 2025	Healthy control group who had no music therapy
Impellizzeri et al, 2020	6 sessions per week for 8 weeks of conventional cognitive rehabilitation
Kuhlmann et al, 2025	Patients laid on top of a monochord and remained relaxed in a supine position in silence
Maggio et al, 2021	Conventional cognitive rehabilitation (same amount of conventional physiotherapy, stretching, muscle strength and occupational therapy as intervention group)
Moore et al, 2008	Spoken word group – learn and recall words without music
Moumdjian et al, 2019	Healthy control group who had no music therapy or metronome
Naderi et al, 2022	Healthy control group who had the same intervention
Schmid et al, 2004	No intervention
Seebacher et al, 2024	[No control arm]
Seebacher et al, 2019	[No control arm]
Seebacher et al, 2018	[No control arm]
Seebacher et al, 2017	No music therapy
Seebacher et al, 2015	No music therapy
Seighalani et al, 2014	No intervention
Shahraki et al, 2017	Exercises as per intervention group but without rhythmic auditory stimulation
Thaut et al, 2008	Spoken word list (same words as sung by intervention group)
Thaut et al, 2014	Spoken 15-word list (same words as sung by intervention group)
Wiens et al, 1999	3 sessions of 30 minutes per week for 12 weeks with a nurse – listened to music, discussed music, learnt about music and composers (but no active music therapy)

For peer review only

Young et al, 2019	No active music therapy or yoga

For peer review only

Supplementary file 9. Narrative results for included studies

Author, year	Results
Aldridge et al. 2005	There were no statistically significant differences between the music therapy group and the control group; however, medium effect sizes were observed for self-esteem (d = 0.54), depression (HAD-D d = 0.63), and anxiety (HAD-A d = 0.63). Within the music therapy group, significant improvements in self-esteem, depression, and anxiety were seen over the treatment period (U1–U4). No statistically significant improvement in quality of life was observed, although a trend towards improvement was reported (HAQUAMS p = 0.066). No differences were found for the functional and physiological values (MSFC, EDSS).
Conklyn et al, 2010	Compared to no intervention, rhythmic auditory stimulation was associated in statistically significant greater improvement in double-support time (left, p = .0176; right, p = .0247). No statistically significant between-group differences were found for the other outcome measures. However, trends with medium to high effect sizes were observed for other gait parameters including walking speed. Within-group analysis showed statistically significant improvement in cadence, stride length, step length, velocity, and normalized velocity. Participant satisfaction level with the rhythmic auditory stimulation intervention was high.
Gatti et al, 2015	Compared to playing with the keyboard turned off, playing with the keyboard turned on was associated with a statistically significant improvement in hand function (p=0.003, Cohen's d = 1.66), while both groups showed a statistically significant improvement in hand dexterity, although between-group differences were not statistically significant. There was also no statistically significant between-group difference in hand strength.
Goetschalckx et al, 2021	Reported results that people with MS performed more poorly than healthy controls were not surprising and outside the scope of the present review. Within people with MS, interlimb coordination was worse at 0.75Hz metronome frequency compared to 1.00Hz and 1.50Hz. Movement amplitude was greatest at 1.00 Hz but reduced at both 0.75Hz and 1.50HZ. Movement frequency followed the metronome tempo. Poorer coordination was statistically significantly correlated with poorer cognitive performance at 0.75Hz and 1.50Hz metronome frequencies, and with reduced walking capacity especially at the 1.50Hz metronome frequency.
Helminger et al, 2025	In people with MS, walking distance in the 2-minute walk test showed a statistically significant improvement only in those classified as responders (>5% improvement), while no statistically significant improvement was shown in the timed 25-foot walk test. Responders showed reductions in brain activation within the premotor cortex, precuneus, and middle frontal gyrus, areas linked to motor planning, spatial guidance, and attention. Across people with MS, greater decreases in brain activation in these regions showed a statistically significant correlation with greater improvements in the 2-minute walk test. There was no observed statistically significant association between walking function and motor imagery-related brain activation changes.
Impellizzeri et al, 2020	The music therapy group showed statistically significant improvements in cognitive function (p < .001), long-term retrieval (p= 0.007), delayed recall (p=0.001), and on multiple subscales of motivation, emotional awareness, depression, and quality of life. The between-group effect versus conventional cognitive rehabilitation did not show any statistically

For peer review only

	significant differences (either better or worse). It should be noted that conventional cognitive rehabilitation was administered to both arms.
Kuhlmann et al, 2025	Compared to the silent condition, monochord music therapy was associated with a statistically significant reduction in psychosocial fatigue (p=0.029), increased heat pain thresholds (p=0.024) and stronger immediate subjective effects (e.g. feeling balanced: p < 0.001, relaxed: p < 0.001, less pain: p < 0.001). However, there was no statistically significant between-group difference in anxiety levels (p=0.109).
Maggio et al, 2021	Participants receiving music-assisted treadmill training demonstrated significant improvements in balance (BBS, p<0.002), walking speed (10mWT, p<0.002), mobility (TUG, p<0.002), mood (BDI-II, p<0.002), physical QoL (MSQOL-PH, p<0.002), mental QoL (MSQOL-MH, p<0.001), and goal attainment (GAS, p<0.002). Compared with conventional gait training, significantly greater improvements were observed in walking speed (p=0.004), mobility (p=0.001–0.003), physical QoL (p=0.004), mental QoL (p<0.001), and goal attainment (p=0.002).
Moore et al, 2008	There were no statistically significant differences between the spoken word and sung groups for recognition accuracy, false alarms, sensitivity or response bias.
Moumdjian et al, 2019	Participants were able to synchronize to the different tempi. People without cognitive impairment showed statistically significantly higher synchronization with the metronome compared with music (p < 0.001). People with MS perceived statistically significantly less physical (p < 0.0001) and cognitive fatigue (p < 0.0001) when walking to music. People with cognitive impairment perceived lower fatigue walking to music compared with metronomes (p < 0.0001).
Naderi et al, 2022	Rhythmic auditory stimulation statistically significantly improved gait symmetry and spatiotemporal parameters in MS patients. At 90% tempo, stride length, stride width, and double support duration increased, while cadence decreased. At 110% tempo, cadence increased while stride length, stride width, and double support duration decreased, indicating improved efficiency and stability. Across all tempi (90%, 100%, 110%), gait symmetry indices improved compared to walking without rhythmic auditory stimulation.
Schmid et al, 2004	No significant between-group differences were observed in functional status (MSFC), self-acceptance, depression, anxiety, or quality of life at the end of treatment. However, medium effect sizes favoured music therapy for self-esteem (d=0.54), depression (d=0.63), and anxiety (d=0.63). Within the music therapy group, significant improvements were observed in self-acceptance (p=0.012), depression (p=0.035–0.036), and anxiety (p=0.013), while no significant changes were found in functional status or quality of life.
Seebacher et al, 2024	Music-cued motor imagery, combined motor imagery and gait training, and conventional gait training (CGT) all improved walking performance, fatigue, and several domains of health-related quality of life. However, no significant between-group differences or interaction effects were observed for quality of life outcomes. Reductions in physical, cognitive, and total fatigue were reported across all groups, with a significant between-group difference favouring CGT for cognitive fatigue (p=0.048). No significant effects were reported for anxiety, depression, or cognitive function.

For peer review only

Seebacher et al, 2019	All music interventions statistically significantly improved walking speed and distance. Motor outcomes, fatigue and quality of life significantly improved only after music and verbally cued motor imagery. Synchronisation improved more with music and verbal motor imagery and music motor imagery and worsened with just motor imagery.
Seebacher et al, 2018	Feasibility was demonstrated. There were no safety-related or adverse events related to the study. Improvements in all groups for walking speed and distance, with greatest improvement in the music and verbally cued motor imagery group. Improvements in fatigue, quality of life and motor imagery ability were observed. Only one participant (in the music and verbally cued motor imagery group) showed a clinically significant improvement (defined as at least 20%) in the timed 25-foot walk test. 60% of participants in the music and verbally cued motor imagery group and 40% of participants in other groups showed a clinically significant improvement (defined as at least 20%) in the 6-minute walk test. Fatigue decreased and QoL improved following music-cued and non-cued motor imagery interventions, although findings were exploratory as this is a feasibility study.
Seebacher et al, 2017	Music-cued and metronome-cued motor imagery significantly improved walking speed and walking distance compared with controls (both $p<0.0001$). Cognitive and total fatigue improved in both intervention groups, while physical fatigue, vitality, and several QoL domains improved only after music-cued motor imagery.
Seebacher et al, 2015	Motor imagery with instrumental music and metronome cues statistically significantly improved walking speed and distance in intervention groups compared to no music.
Seighalani et al, 2014	Compared to no intervention, music therapy significantly reduced depression and anxiety from pretest to post-test and follow-up, with effects persisting over time. Depression scores decreased markedly (39.13 to ~17), and anxiety scores halved (41.07 to ~21). Self-esteem scores improved substantially, rising from 16.00 at pretest to ~37–38 at post-test and follow-up. While differences between post-test and follow-up were minimal, all outcomes showed significant improvements compared to pretest.
Shahraki et al, 2017	Statistically significant improvements in the gait parameters stride length, time, double support time, cadence, and gait speed were found in the rhythmic auditory stimulation group compared to the control group doing the same exercises without rhythmic auditory stimulation.
Thaut et al, 2008	Music-based learning was associated with superior word order recall compared with spoken learning,($F(1,18) = 4.51$, $p = 0.038$). Musical learning led to greater improvements in early and late learning phases, while spoken learning showed only transient gains that reduced toward the end of the task.
Thaut et al, 2014	The sung word list group showed statistically significantly more improvement in word order memory and recall ($p= 0.038$) and bilateral frontal alpha learning related synchronization ($p= 0.05$) than the spoken word group.
Wiens et al, 1999	Compared to passive music engagement, active music therapy was not shown to be associated with statistically significant improvements in the strength of respiratory muscles in people with advanced MS.
Young et al, 2019	Statistically significant improvements for music to movement versus no active music therapy or yoga were shown for in Timed Up and Go (least square mean difference [95% confidence interval] = −1.9s [−3.3 to −0.5], P=.01, d=0.7 and 6-minute walk test (41.0m [2.2-80.0], P=.04, d=0.6; controlled for Patient-Determined Disease Steps). No significant group

	differences were found in 5-times sit-to-stand, fatigue, and pain interference. Tests of music to movement versus control were post-hoc as the study also contained an active yoga intervention and the primary comparisons were made across music to movement, active yoga and control.

For peer review only

Supplementary file 10. Risk of bias results

Risk of bias and methodological quality were assessed using the Critical Appraisal Skills Programme (CASP) checklists, selected according to study design (randomised controlled trials, cohort studies, case control studies, and cross-sectional studies). CASP was chosen to provide a structured, domain-based assessment of internal validity, applicability, and reporting quality, rather than a single summary score. Interpretation focused on domain level patterns of bias, consistent with recommendations to avoid reliance on composite quality scores.
Across the 23 included studies, the RCT checklist was the most frequently applied, reflecting that most studies were experimental in design. Where previous reviews had inconsistently labelled some studies as non-randomised, quasi experimental, or clinical trials, the present review applied CASP checklists based on the actual methodological features reported, rather than labels used in earlier syntheses. This resulted in three intentional mismatches between study labels used in prior reviews and the CASP checklist applied here. For example, Maggio et al (2021) and Naderi et al (2022) were assessed using the RCT checklist despite being described as quasi experimental in some summaries, as both involved prospective intervention allocation and comparator groups. Conversely, Aldridge et al (2005) and Schmid et al (2004) were assessed using the cohort checklist, as allocation was matched rather than randomised. These decisions were made to ensure the most appropriate appraisal tool was applied to each study's actual design.

Overall, most studies addressed a clearly focused research question, reported outcomes comprehensively, and used validated outcome measures. Baseline characteristics were generally comparable between intervention and control groups where applicable. However, several recurring sources of bias were identified across studies. Blinding of participants and intervention providers was rarely possible, reflecting the nature of music-based interventions. While some studies reported assessor blinding, this was inconsistently applied or insufficiently described. Allocation concealment was rarely reported and may represent either a reporting limitation or a true methodological weakness.

Sample size was a common concern. Many studies were small, exploratory, or pilot in nature, limiting statistical power and increasing the risk of type II error. Participant flow and attrition were not always clearly reported, particularly in older studies and non-randomised designs, raising potential concerns regarding selection and attrition bias. Although most studies reported statistically significant findings where present, clinical significance was rarely addressed, and confidence intervals or precision estimates were inconsistently reported.

Observational and non-randomised studies generally demonstrated appropriate measurement of exposure and outcomes, but often relied on convenience sampling, increasing the risk of selection bias. Cross sectional and case control designs were limited in their ability to account for confounding, although the case control study assessed using CASP demonstrated strong internal consistency across domains. Cohort studies scored highly overall but lacked detailed reporting of precision and effect estimates.

Several important limitations were identified that are not fully captured by CASP or other standard risk of bias tools. These include heterogeneity in intervention content, duration, and delivery personnel; variability in outcome measures across studies; frequent underrepresentation of male participants; and reliance on self-reported outcomes in several domains. In addition, some single session experimental studies lacked a comparator capable of controlling for non-specific effects such as attention, novelty, or participant expectation.

For peer review only

Despite these limitations, higher quality RCTs demonstrated generally good internal validity, clear outcome reporting, and appropriate analytical approaches. Studies rated as high quality were more likely to include blinded outcome assessment, clearer reporting of statistical methods, and better alignment between results and conclusions. Across study designs, conflicts of interest were not identified as a major concern, and authors typically acknowledged at least some methodological limitations.

Overall, the risk of bias profile suggests that while many studies were thoughtfully designed and conducted within the practical constraints of arts-based research, methodological limitations were common, particularly relating to blinding, sample size, and reporting transparency. These factors should be considered when interpreting the strength and consistency of the evidence base.

CASP risk of bias checklists

Part A: RCT checklist

**Study**	**Clear research question?**	**Randomised?**	**All participants accounted for?**	**Participants blinded?**	**Investigators blinded?**	**Assessors blinded?**	**Similar baselines?**
Conklyn et al, 2010	Yes	Yes	Yes	No	No	No	Yes
Gatti et al, 2015	Yes	Yes	Yes	No	No	No	Yes
Helminger et al. 2025	Yes	Yes	Yes	No	No	No	Yes
Impellizzeri et al, 2020	Yes	Yes	Yes	Yes	No	No	Yes
Kuhlmann et al, 2025	Yes	Yes	No	No	Yes	No	Yes
Maggio et al. 2021	Yes	No	Yes	No	No	No	Yes
Moore KS. et al. 2008	Yes	Yes	Yes	No	No	No	Yes
Naderi S. Et al. 2022	Yes	No	Yes	No	No	No	Yes
Seebacher et al, 2015	Yes	Yes	Yes	No	No	No	Yes
Seebacher et al, 2017	Yes	Yes	Yes	No	No	No	Yes
Seebacher et al, 2018	Yes	Yes	Yes	No	No	No	Yes
Seebacher et al, 2019	Yes	Yes	Yes	No	No	No	Yes

For peer review only

Seebacher et al, 2024	Yes	Yes	Yes	Yes	Yes	No	Yes
Seighalani et al, 2014	Yes	Yes	Yes	No	No	No	Yes
Shakraki et al, 2017	Yes	Yes	Yes	No	No	No	Yes
Thaut et al, 2008	Yes	yes	Yes	No	No	No	Yes
Thaut et al, 2014	Yes	Yes	Yes	No	Yes	No	Yes
Wiens et al, 1999	Yes	Yes	Yes	No	No	No	Yes
Young et al, 2019	Yes	Yes	Yes	No	No	Yes	Yes

**Study**	**Same level of care?***	**Effects of intervention reported comprehensively?**	**Precision of treatment effect reported?**	**Benefits outweigh harms and costs?**	**Applicable to our context?**	**Greater value than existing treatment?**
Conklyn et al, 2010	Yes	Yes	No	Yes	Yes	Can't tell
Gatti et al, 2015	Yes	Yes	Yes	Yes	Yes	Can't tell
Helminger et al. 2025	Yes	Yes	Yes	Yes	Yes	Can't tell
Impellizzeri et al, 2020	Yes	Yes	Yes	Yes	Yes	Can't tell
Kuhlmann et al, 2025	Yes	Yes	Yes	Yes	Yes	Can't tell
Maggio et al. 2021	Yes	Yes	No	Yes	Yes	Can't tell
Moore. et al. 2008	Yes	Yes	No	Yes	Yes	Can't tell
Naderi. et al. 2022	Yes	Yes	No	Yes	Yes	Can't tell
Seebacher et al, 2015	Yes	Yes	Yes	Yes	Yes	Can't tell
Seebacher et al, 2017	Yes	Yes	Yes	Yes	Yes	Can't tell

For peer review only

Seebacher et al, 2018	Yes	Yes	Yes	Yes	Yes	Can't tell
Seebacher et al, 2019	Yes	Yes	Yes	Yes	Yes	Can't tell
Seebacher et al, 2024	Yes	Yes	Yes	Yes	Yes	Can't tell
Seighalani et al, 2014	Yes	Yes	No	Yes	Yes	Can't tell
Shakraki et al, 2017	Yes	Yes	No	Yes	Yes	Can't tell
Thaut et al, 2008	Yes	Yes	No	Yes	Yes	Can't tell
Thaut et al, 2014	Yes	Yes	No	Yes	Yes	Can't tell
Wiens et al, 1999	Yes	Yes	No	Yes	Yes	Can't tell
Young et al, 2019	Yes	Yes	Yes	Yes	Yes	Can't tell

* = apart from intervention

Part B: Cross-sectional checklist

**Study**	**Clear research question?**	**Appropriate method?**	**Acceptable recruitment?**	**Accurate measurement to reduce bias?**	**Data collected in a way that addressed research issue?**	**Enough participants?**	**Clear results presentation?**
Goetschalckx et al, 2021	Yes	Yes	Yes	Yes	Yes	Can't tell	Yes

**Study**	**Rigorous analysis?**	**Clear statement of findings?**	**Applicable to local population?**	**How valuable is the research?**
Goetschalckx et al, 2021	Yes	Yes	Yes	Can't tell

Part C: Case control checklist

For peer review only

Study	Clearly focused issue?	Appropriate method?	Acceptable recruitment?	Acceptable control selection?	Exposure accurately measured to minimise bias?	Groups similar apart from exposure?	Confounding taken into account in design and analysis?
Moumdjian et al, 2019	Yes	Yes	Yes	Yes	Yes	Yes	Yes

Study	Large treatment effect?	Precise estimate of treatment effect?	Do you believe the results?	Applicable to our context?	Results fit with other available evidence?
Moumdjian et al, 2019	Can't tell	Can't tell	Yes	Yes	Yes

Part D: Cohort checklist

Study	Clearly focused issue?	Acceptable recruitment?	Exposure accurately measured to minimise bias?	Outcome accurately measured to minimise bias?	All important confounders identified?	Confounding taken into account in design and analysis?	Follow-up complete enough?
Aldridge et al. 2005	Yes	Yes	Yes	Yes	Yes	Yes	Yes
Schmid et al, 2004	Yes	Yes	Yes	Yes	Yes	Yes	Yes

Study	Clear results presentation?	How precise are the results?	Do you believe the results?	Applicable to local population?	Results fit with other available evidence?	Clear implications for practice?
Aldridge et al. 2005	Yes	Can't tell	Yes	Yes	Yes	Can't tell
Schmid et al, 2004	Can't tell	No	Yes	Yes	Yes	Can't tell

For peer review only

Supplemental file 11: Assessment of reporting bias

Study	Protocol available?	Protocol outcomes not reported in journal
Aldridge et al (2005)	No	Not evaluable
Conklyn et al (2010)	No	Not evaluable
Gatti et al (2015)	No	Not evaluable
Goetschalckx et al (2021)	NCT04142853 and NCT03938558	Primary outcomes from the protocol not reported in journal: Stroop test, Nine-hole peg test, Fatigability index hand, motor fatigability, trait motor fatigue*
Helminger et al (2025)	DRKS00023978 (German trials registry) **	All primary outcomes from the protocol were reported in the journal – some secondary clinical and psychological outcomes were not
Impellizzeri et al (2020)	No	Not evaluable
Kuhlmann et al (2025)	DRKS00024549	All protocol outcomes reported in journal
Maggio et al (2021)	No	Not evaluable
Moore et al (2008)	No	Not evaluable
Moumdjian et al (2019)	NCT03281330	All protocol outcomes reported in journal
Naderi et al (2022)	No	Not evaluable
Schmid et al (2004)	No	Not evaluable
Seebacher et al (2015)	ISRCTN67054113	Primary outcomes from protocol (T25FW + 6MWT) switched to secondary outcomes in the journal, one of the secondary outcomes from the protocol (MFIS) switched to primary outcome in the journal*
Seebacher et al (2017)	ISRCTN67054113	All protocol outcomes reported in journal
Seebacher et al (2018)	ISRCTN92351899***	Some of the primary outcomes from the protocol (T25FW and 6MWT) switched to secondary outcomes in the journal
Seebacher et al (2019)	ISRCTN92351899***	All protocol outcomes reported in journal
Seebacher et al (2024)	DRKS00023978**	All protocol outcomes reported in journal
Seighalani et al (2014)	No	Not evaluable
Shahraki et al (2017)	No	Not evaluable
Thaut et al (2008)	No	Not evaluable
Thaut et al (2014)	No	Not evaluable
Wiens et al (1999)	No	Not evaluable
Young et al (2019)	NCT02533882	Primary outcomes from the protocol not reported in journal: VO2 fitness, pain, fatigue, loneliness, grip strength, balance tests*

For peer review only

All protocols were published prospectively rather than retrospectively, * there were also secondary outcomes from the protocol not reported in the journal (however where primary outcomes from the protocol are not reported in the journal we focus on listing these in the table), ** while these studies share a protocol registration number, they are separate studies with different aims and outcomes (total sample size across the two studies = 147, the sample sizes for the two separate studies are 121 and 31 considering only included participants, suggesting an overlap of 5 participants - 3% of the total sample size across the two studies), *** while these two studies share a protocol registration number, they are separate studies and have no overlap in participants (total sample size across the two studies = 75, the sample sizes for the two separate studies are 15 and 60).

For peer review only

Supplemental file 12. Effect direction plot

Study	Study Design	Motor outcomes	Cognitive outcomes	Psychological outcomes	Quality of life outcomes
Aldridge D. et al. 2005	Matched controlled pilot study		◀▶	▲	◀▶
Conklyn D. et al. 2010	RCT	▲			◀▶
Gatti R. Et al. 2015	RCT	▲			
Goetschalckx M. et al. 2021	Observational cross-sectional comparative study	◀▶			
Helminger B. et al. 2025	RCT	◀▶			
Impellizzeri et al, 2020	RCT		▲	▲	▲
Kuhlmann J et al. 2025	RCT			▲	▲
Maggio et al. 2021	Quasi-experimental controlled feasibility study	▲	▲	▲	▲
Moore KS. et al. 2008	RCT		◀▶		
Moumdjian L. et al. 2019	Observational case control study	▲			▲
Naderi S. Et al. 2022	Quasi-experimental controlled study with repeated measures	▲			
Schmid et al. 2014	Matched control study	◀▶	◀▶	▲	◀▶
Seebacher B. et al. 2015	RCT	▲			▲
Seebacher B. et al. 2017	RCT	▲			▲
Seebacher B. et al. 2018	RCT	▲			▲

For peer review only

Seebacher B. et al. 2019	RCT	▲			▲
Seebacher B. et al. 2024	RCT	▲	◄►	◄►	▲
Seighalani M. et al. 2014	RCT			▴	
Shahraki M et al, 2017	RCT	▴			
Thaut et al. 2008	RCT		▴		
Thaut M. et al. 2014	RCT		▲		
Wiens M E et al. 1999	RCT	◂▸			
Young HJ. Et al. 2019	RCT	▲			◄►

Legend:
Study design: RCT: Randomised Controlled Trial. Effect direction: upward arrow ▲= positive health impact, downward arrow ▼= negative health impact, sideways arrow ◄►= no change/mixed effects/conflicting findings. Sample size: Final sample size (individuals) in intervention group Large arrow ▲ >300; medium arrow ▲ 50-300; small arrow ▴ <50. Following Boon and Thomson[82]

For peer review only

Supplemental file 13. Evaluation of minimally clinically important differences

Outcome domain	Measure	MCID	Findings
Motor	Timed 25-foot walk test	17.2% improvement[76]	• Helminger et al[49] – no clinically significant difference in participants receiving actual and/or imagined gait training with rhythmic-auditory cueing from baseline to follow-up. **Within-group**<br>• Seebacher et al[55] – clinically significant difference in participants receiving music cued motor imagery (17.3%) and metronome cued motor imagery (20.0%), but not controls, from baseline to follow-up. **Within-group**<br>• Seebacher et al[56] – no clinically significant difference in any group from baseline to follow-up. **Within-group**<br>• Seebacher et al[57] – no group-level clinically significant difference in any group from baseline to follow-up. One out of five participants in the music- and verbally cued motor imagery group had a clinically significant difference and no participants in other groups. **Within-group**<br>• Seebacher et al [58] – no group-level clinically significant difference in any group from baseline to follow-up. 21.1% of participants in the in the music- and verbally cued motor imagery group, 15.0% in the music-cued motor imagery and none in the motor imagery group had a clinically significant difference (defined as >=20% by the authors of this study). **Within-group**<br>• Seebacher et al[59] – no clinically significant difference in any group from baseline to the final follow-up (week 13). **Within group**<br><br>One study out of six found evidence of a clinically significant difference. All assessed within-group rather than between-group differences, although for some studies information was available for more than one group.

For peer review only

Psychological	Beck depression	17.5% reduction[77]	• Impellizeri et al[32] – clinically significant difference in experimental group (46.4%) but not the control group from baseline to follow-up. **Within-group**<br>• Maggio et al[51] – clinically significant difference in experimental group (56.2%) but not the control group from baseline to follow-up. **Within-group**<br>• Schmid et al[30] - numerical descriptive results not presented, therefore not evaluable<br>Two studies out of two (that were evaluable) found evidence of a clinically significant difference. Both assessed within-group rather than between-group differences, although information was available for more than one group.
Psychological	HADS anxiety	0.80 points (for subscale)[79]	• Kuhlmann et al[50] – no clinically significant difference from baseline to follow-up for the experimental group, although a clinically significant improvement (1.5 points) was found for the control group. **Within-group**<br>• Schmid et al[30] – numerical descriptive results not presented, therefore not evaluable<br>• Seebacher et al[59] – secondary outcome but data not presented, therefore not evaluable<br>No studies out of one (that were evaluable) found evidence of a clinically significant difference. This study assessed within-group rather than between-group differences, although information was available for more than one group.
QoL	MS Impact Scale-29 (MSIS-29)	8 points for physical subscale[81]	• Seebacher et al[58] – clinically significant difference in the music- and verbally-cued motor imagery group (15 points), but not the music-cued motor imagery or motor imagery groups. 79% of participants in the music- and verbally cued motor imagery group, 50% of participants in the music-cued motor imagery group, and 35% of participants in the motor imagery group had a clinically

			significant difference, defined as 7.5 points by the authors of this study.<br>**Within-group**<br>• Seebacher et al[57] – no clinically significant difference in any group from baseline to follow-up (although the music-cued motor imagery group met the more lenient threshold of 7.5 points).<br>**Within-group**<br>• Seebacher et al[56] – no clinically significant difference in any group from baseline to follow-up. 50% of participants in the music- and verbally cued motor imagery group, 41.2% in the music-cued motor imagery group, and 21.2% of those in the motor imagery group had a clinically significant difference, defined as 7.5 points by the authors of this study.<br>**Within-group**<br>One study out of three found evidence of a clinically significant difference. All assessed within-group rather than between-group differences, although information was available for more than one group.

Cognitive MCIDs could not be evaluated, as explained in Table 2.

For peer review only
